# Transcriptomic Analysis of Porcine Endometrium during Implantation after In Vitro Stimulation by Adiponectin

**DOI:** 10.3390/ijms20061335

**Published:** 2019-03-16

**Authors:** Nina Smolinska, Karol Szeszko, Kamil Dobrzyn, Marta Kiezun, Edyta Rytelewska, Katarzyna Kisielewska, Marlena Gudelska, Kinga Bors, Joanna Wyrebek, Grzegorz Kopij, Barbara Kaminska, Tadeusz Kaminski

**Affiliations:** Department of Animal Anatomy and Physiology, Faculty of Biology and Biotechnology, University of Warmia and Mazury in Olsztyn, Oczapowskiego 1A, 10-719 Olsztyn-Kortowo, Poland; karol.szeszko@uwm.edu.pl (K.S.); kamil.dobrzyn@uwm.edu.pl (K.D.); marta.kiezun@uwm.edu.pl (M.K.); edyta.rytelewska@uwm.edu.pl (E.R.); katarzyna.kisielewska@uwm.edu.pl (K.K.); marlena.gudelska@uwm.edu.pl (M.G.); kinga.bors@uwm.edu.pl (K.B.); joanna.wyrebek@uwm.edu.pl (J.W.); grzegorz.kopij@student.uwm.edu.pl (G.K.); barbara.kaminska@uwm.edu.pl (B.K.); tkam@uwm.edu.pl (T.K.)

**Keywords:** endometrium, implantation, transcriptome, microarray, adiponectin, pig

## Abstract

Comprehensive understanding of the regulatory mechanism of the implantation process in pigs is crucial for reproductive success. The endometrium plays an important role in regulating the establishment and maintenance of gestation. The goal of the current study was to determine the effect of adiponectin on the global expression pattern of genes and relationships among differentially expressed genes (DE-genes) in the porcine endometrium during implantation using microarrays. Diverse transcriptome analyses including gene ontology (GO), biological pathway, networks, and DE-gene analyses were performed. Adiponectin altered the expression of 1286 genes with fold-change (FC) values greater than 1.2 (*p* < 0.05). The expression of 560 genes were upregulated and 726 downregulated in the endometrium treated with adiponectin. Thirteen genes were selected for real-time PCR validation of differential expression based on a known role in metabolism, steroid and prostaglandin synthesis, interleukin and growth factor action, and embryo implantation. Functional analysis of the relationship between DE-genes indicated that adiponectin interacts with genes that are involved in the processes of cell proliferation, programmed cell death, steroid and prostaglandin synthesis/metabolism, cytokine production, and cell adhesion that are critical for reproductive success. The presented results suggest that adiponectin signalling may play a key role in the implantation of pig.

## 1. Introduction

Understanding the mechanisms controlling energy homeostasis and reproduction creates the foundation and opens a way towards future effective modification of these processes in farm animals. Most authors agree that 20% to 30% of porcine embryos are lost between days 12 to 30 of pregnancy (reviewed by Reference [1]). This critical period for embryos is not yet fully understood. Establishment and maintenance of pregnancy require reciprocal interactions between the conceptus and endometrium. The embryos migrating within the uterus about days 10 to 11 of pregnancy synthesize and secrete oestradiol (E2) and prostaglandin E2 (PGE2), the key hormonal components of the process of maternal recognition of pregnancy (days 12 to 13). On the other hand, the endometrium undergoes hormonally regulated (mainly by progesterone (P4) and E2) alteration of receptivity in order to attach and implant the embryo, which occurs around days 15 to 16 of gestation. The period of uterine receptivity, called the “window of implantation”, is also related to the presence of growth factors, multiple adhesion molecules, such as integrins, or proteolytic enzymes, such as metalloproteinases, which are essential for embryo implantation. During this period, the endometrium produces prostaglandins, chemokines, and cytokines, which are responsible for the proper course of the implantation process [2,3,4]. During the peri-implantation period, properly synchronized expression of genes for many factors, e.g., steroid hormones, prostaglandins, cytokines, angiogenic factors, apoptosis-related substances, integrins, and metalloproteinases in the uteri, conceptuses, and trophoblasts are important for effective maternal recognition of pregnancy and implantation, and consequently it is essential for embryo survival and development. Adiponectin, which is a member of adipokine family, could belong to the group of hormones that affect the processes/mechanisms related to the peri-implantation period directly or indirectly by controlling the synthesis of the above factors.

Adiponectin is a 30 kDa cytokine, predominantly synthesized and secreted by the adipose tissue and circulating at high levels in the bloodstream [5,6]. In the plasma, adiponectin can be found in the form of a trimer (low molecular weight, LMW), a combination of two trimers (middle molecular weight, MMW) or a structure of six trimers (high molecular weight, HMW) [7]. Adiponectin levels in the blood and in the adipose tissue are negatively associated with obesity [6,8]. Adiponectin exerts its effect by binding to two transmembrane receptors, adiponectin receptor type 1 (AdipoR1) and adiponectin receptor type 2 (AdipoR2). Adiponectin receptor type 1 demonstrates a higher affinity for the trimeric form of adiponectin, and it is found at the highest concentrations in skeletal muscles, whereas AdipoR2, found mainly in the liver, shows greater affinity for MMW and HMW forms. Adiponectin regulates energy homeostasis by fatty-acid oxidation, stimulation of glucose uptake, and inhibition of gluconeogenesis. The above leads to intensified thermogenesis, weight loss, and increased insulin sensitivity in tissues [9]. This adipokine possesses anti-atherogenic, anti-inflammatory, anti-proliferative, anti-angiogenic, and pro-apoptotic properties (for review see Reference [10]).

Accumulating evidence suggests the involvement of adiponectin in reproduction control (for review see References [11,12]). Adiponectin and its receptors (the adiponectin system) have been found in the female reproductive tract of different species such as human, rat, mouse, and pig [13,14,15,16,17,18]. Our previous findings show that the concentrations of adiponectin system transcripts and proteins in the porcine endometrium and myometrium fluctuated during different phases of the oestrous cycle and pregnancy, which indicates that females’ hormonal status influences the expression of adiponectin system components and the sensitivity of the examined structures to the adipokine [19,20]. Our study revealed that adiponectin regulates the expression of steroidogenic enzymes genes and the secretion of steroid hormones by *in vitro* incubated porcine endometrial and myometrial explants during early pregnancy and on days 10 to 11 of the oestrous cycle [21]. Based on our observations, we hypothesized that adiponectin may be an important factor in the regulation of processes taking place in the uterus. Thus, the aim of the present study was to investigate the comprehensive effect of adiponectin on the transcriptome of the porcine endometrial cells during the implantation period, on days 15 to 16 of pregnancy. The proposed study will help to identify genes and the processes modified by adiponectin, and thereby provide further evidence that adiponectin plays a role in reproduction.

## 2. Results 

### 2.1. Microarray Data Analysis

#### 2.1.1. Identification of Differentially Expressed (DE) Genes

Transcriptome analyses were carried out to compare the gene expression between the control endometrial tissue explants and endometrial tissue explants treated with 10 μg/mL of adiponectin. A complete list of DE-genes recognized during this study is presented in table format in the Table S1. The table includes a probe set name, gene ID, fold change, *p*-value, the direction of change (up or down), as well as function and accession number. The whole number of DE-genes generated by GeneSpring was 1494, among which 1286 genes had a FC values greater than 1.2 (*p* < 0.05). The expression of 560 genes was upregulated and 726 downregulated in the endometrium treated with adiponectin.

#### 2.1.2. Gene Ontology Analysis

Gene ontology enrichment analysis was used to perform a functional categorisation of the DE-genes. As a result of the analysis prepared in DAVID (Database for Annotation, Visualization and Integrated Discovery), three GO categories were specified: biological process (BP), metabolic function (MF), and cell component (CC). The results for the entire DE-gene list (up- and downregulated; modified Fisher’s exact test *p* < 0.1) are summarized in the Table S2. The most significantly enriched genes ontologies were obtained under the BP category. Furthermore, two additional BP lists were generated from upregulated and downregulated DE-genes (Supplementary Materials Table S3). These results allowed us to choose 102 of the most important processes for endometrium functioning, including 35 and 67 processes connected to up- and downregulated DE-genes, respectively (Supplementary Materials Table S4). In the analysis conducted on the basis of the upregulated DE-gene list, we observed a group of 47 gene products associated with GO terms related to *gene expression* (GO:0010467). Most of these gene products (26) were involved in *transcription*, *DNA-templated* (GO:0006351), which suggests that adiponectin induces transcription in the endometrial cells. The next group of 39 gene products was involved in the *RNA metabolic processes* (GO:0016070), including 30 engaged in *RNA biosynthetic processes* (GO:0032774) and *nucleic acid-templated transcription* (GO:0097659). Adiponectin also had an effect on the *regulation of gene expression* (GO:0010468), *regulation of transcription*, *DNA-templated* (GO:0006355), and the *positive regulation of pri-miRNA transcription from RNA polymerase II promoter* (GO:1902895) by increasing the expression of 33, 24, and 2 DE-genes, respectively. The next group of 33 gene products was involved in the *regulation of nitrogen compound metabolic processes* (GO:0051171): 27 of these gene products were connected with the *regulation of nucleobase-containing compound metabolic processes* (GO:0019219), 25 with the *regulation of RNA metabolic processes* (GO:0051252), 24 with the *regulation of RNA biosynthetic processes* (GO:2001141), and also 24 with the *regulation of nucleic acid-templated transcription* (GO:1903506).

Another biological process modulated by the studied hormone were the *regulation of macromolecule biosynthetic processes* (GO:0010556) and *regulation of cellular macromolecule biosynthetic processes* (GO:2000112). We identified 31 and 29 upregulated DE-genes related to these GO terms, respectively. The adipokine increased the expression of 15 genes, whose products were involved in the *cellular catabolic processes* (GO:0044248). Another 14 gene products were associated with the *vesicle-mediated transport* (GO:0016192). The next group of 12 gene products was involved in the *DNA metabolic processes* (GO:0006259) including seven and five engaged in *DNA repair* (GO:0006281) and *DNA recombination* (GO:0006310), respectively. Another biological process modulated by the adiponectin was the *protein modification by small protein conjugation or removal* (GO:0070647). Moreover, nine DE-genes were involved in the regulation of the *response to abiotic stimulus* (GO:0009628) and three of these in the *cellular response to light stimulus* (GO:0071482). The next group of eight gene products was involved in the *ribonucleoprotein complex biogenesis* (GO:0022613) and three in *ribosome biogenesis* (GO:0042254). Another biological process modulated by the studied hormone was the *nucleoside metabolic processes* (GO:0009116; 6 DE-genes). The same group of six gene products was also involved in *purine nucleoside metabolic process* (GO:0042278) and *purine ribonucleotide metabolic process* (GO:0046128). Another group of six gene products was involved in *glycosyl compound metabolic processes* (GO:1901657). The last five significant results were related to *nucleoside triphosphate metabolic processes* (GO:0009141; 5 DE-genes), *positive regulation of cell projection organization* (GO:0031346; 5 DE-genes), *cell–matrix adhesion* (GO:0007160; 4 DE-genes), *translational initiation* (GO:0006413; 4 DE-genes), and *regulation of mRNA 3’-end processing* (GO:0031440; 2 DE-genes). In the group of genes connected to *positive regulation of cell projection organization*, four gene products were also involved in *positive regulation of neuron projection development* (GO:0010976).

In the case of GO analysis of downregulated DE-genes, the most significant group consisted of 85 genes whose products were involved in the *regulation of gene expression* (GO:0010468); 41 of these gene products were connected with the *positive regulation of gene expression* (GO:0010628), 36 with the *negative regulation of gene expression* (GO:0010629) and six with *gene silencing* (GO:0016458). Another 56 gene products were associated with *the negative regulation of metabolic processes* (GO:0009892), including 53 involved in the *negative regulation of cellular metabolic processes* (GO:0031324), and 23 in the *negative regulation of cellular protein metabolic processes* (GO:0032269). The next group of 41 gene products was involved in 19 GO terms related to the *regulation of immune system processes* (GO:0002682), including *positive regulation of immune system processes* (GO:0002684; 27 DE-genes), *regulation of immune response* (GO:0050776; 15 DE-genes), *positive regulation of immune response* (GO:0050778; 13 DE-genes), *activation of immune response* (GO:0002253; 10 DE-genes), *regulation of hemopoiesis* (GO:1903706; 13 DE-genes), *positive regulation of hemopoiesis* (GO:1903708; 7 DE-genes), *negative regulation of hemopoiesis* (GO:1903707; 7 DE-genes), *regulation of myeloid cell differentiation* (GO:0045637; 10 DE-genes), *positive regulation of myeloid cell differentiation* (GO:0045639; 5 DE-genes), *negative regulation of myeloid cell differentiation* (GO:0045638; 5 DE-genes), *regulation of erythrocyte differentiation* (GO:0045646; 6 DE-genes), *negative regulation of erythrocyte differentiation* (GO:0045647; 3 DE-genes), *regulation of innate immune response* (GO:0045088; 8 DE-genes), *positive regulation of innate immune response* (GO:0045089; 7 DE-genes), *regulation of leukocyte migration* (GO:0002685; 8 DE-genes), *leukocyte tethering or rolling* (GO:0050901; 3 DE-genes), *regulation of neutrophil migration* (GO:1902622; 3 DE-genes), and *regulation of neutrophil chemotaxis* (GO:0090022; 3 DE-genes). Another biological process modulated by the studied adipokine were the *regulation of cell proliferation* (GO:0042127; 38 DE-genes) and *negative regulation of cell proliferation* (GO:0008285; 17 DE-genes). The next group of 36 gene products was involved in *cell proliferation* (GO:0008283). The studied hormone also had an effect on *programmed cell death* (GO:0012501) by decreasing the expression of 35 DE-genes. Additionally, we identified 29 downregulated DE-genes related to *biological adhesion* (GO:0022610), 29 of these DE-genes affect *cell adhesion* (GO:0007155), 24 with *single organism cell adhesion* (GO:0098602), 23 with *single organismal cell–cell adhesion* (GO:0016337), 23 with *cell–cell adhesion* (GO:0098609), 17 with *leukocyte cell–cell adhesion* (GO:0007159), four with *leukocyte adhesion to vascular endothelial cell* (GO:0061756), and seven affect *cell–matrix adhesion* (GO:0007160). Moreover, 28 DE-genes were involved in *immune system development* (GO:0002520) and *hematopoietic or lymphoid organ development* (GO:0048534), 26 of these DE-genes in the *hemopoiesis* (GO:0030097) including the *myeloid cell differentiation* (GO:0030099; 17 DE-genes), *erythrocyte differentiation* (GO:0030218; 8 DE-genes), and *myeloid cell development* (GO:0061515; 4 DE-genes).

Adiponectin also downregulated 18 DE-genes associated with the *multicellular organism reproduction* (GO:0032504) and *multicellular organismal reproductive processes* (GO:0048609). The group of 17 of these genes was related to *sexual reproduction* (GO:0019953), 14 to *gamete generation* (GO:0007276), three to the *ovulation* (GO:0030728) and four to *ovulation cycle processes* (GO:0022602). The next group of 17 gene products was involved in *developmental processes involved in reproduction* (GO:0003006) including eight engaged in *development of primary sexual characteristics* (GO:0045137) and *development of primary female sexual characteristics* (GO:0046545), five DE-genes in the *female sex differentiation* (GO:0046660) processes. Other biological processes modulated by the studied adipokine were *cytokine production* (GO:0001816; 16 DE-genes), *cytokine metabolic processes* (GO:0042107; 5 DE-genes), and *cytokine biosynthetic processes* (GO:0042089; 5 DE-genes). Next, the biological process worth emphasizing was the *regulation of cell adhesion* (GO:0030155), which was represented by 16 DE-genes. Moreover, 13 of these DE-genes affected the *regulation of cell–cell adhesion* (GO:0022407), 10 affected the *regulation of leukocyte cell–cell adhesion* (GO:1903037), and 11 affected the *positive regulation of cell adhesion* (GO:0045785). Adiponectin had also an effect on the *reproductive system development* (GO:0061458) by decreasing the expression of 12 DE-genes. Most of these were involved in *reproductive structure development* (GO:0048608), *gonad development* (GO:0008406), and *female gonad development* (GO:0008585). The next group of 12 gene products was involved in the *negative regulation of cell cycle* (GO:0045786). The last two processes affected by adiponectin were the *regulation of haemostasis* (GO:1900046; 5 DE-genes) and *positive regulation of cytokine biosynthetic processes* (GO:0042108; 4 DE-genes).

#### 2.1.3. Biological Pathway Analysis

Forty-four biological pathways were generated using the KEGG (Kyoto Encyclopedia of Genes) database (Table 1). The pathways with the largest number of involved genes were *pathways in cancer* (21 DE-genes). The other pathways indicated by the DAVID tool that were affected by adiponectin treatment were as follows: *cytokine–cytokine receptor interaction* (18 DE-genes), *Jak–STAT signalling pathway* (16 DE-genes), *regulation of actin cytoskeleton* (15 DE-genes), *HTLV-I infection* (15 DE-genes), *transcriptional misregulation in cancer* (14 DE-genes), *herpes simplex infection* (14 DE-genes), *viral carcinogenesis* (14 DE-genes), *insulin signalling pathway* (13 DE-genes), *chemokine signalling pathway* (13 DE-genes), *Epstein–Barr virus infection* (12 DE-genes), *measles* (11 DE-genes), *ubiquitin mediated proteolysis* (11 DE-genes), *prolactin signalling pathway* (10 DE-genes), *Salmonella infection* (10 DE-genes), *NF-kappa B signalling pathway* (10 DE-genes), *TNF signalling pathway* (10 DE-genes), *hepatitis C* (10 DE-genes), and *osteoclast differentiation* (10 DE-genes). Less significant pathways included the i.a. *ErbB signalling pathway (9 DE-genes)*, *toll-like receptor signalling pathway* (9 DE-genes), *insulin resistance (9 DE-genes)*, *ribosome biogenesis in eukaryotes* (8 DE-genes), *bacterial invasion of epithelial cells* (7 DE-genes), *peroxisome* (7 DE-genes), *small-cell lung cancer* (7 DE-genes), *phosphatidylinositol signalling system* (7 DE-genes), *systemic lupus erythematosus* (7 DE-genes), *NOD-like receptor signalling pathway* (5 DE-genes), *ovarian steroidogenesis* (5 DE-genes), *pentose phosphate pathway* (4 DE-genes), *intestinal immune network for IgA production* (4 DE-genes), *fatty-acid biosynthesis* (3 DE-genes), *glycosaminoglycan biosynthesis–chondroitin sulfate/dermatan sulfate* (3 DE-genes), *nicotinate*, *and nicotinamide metabolism* (3 DE-genes).

#### 2.1.4. Network between Differentially Expressed Genes

**Figure 1:**
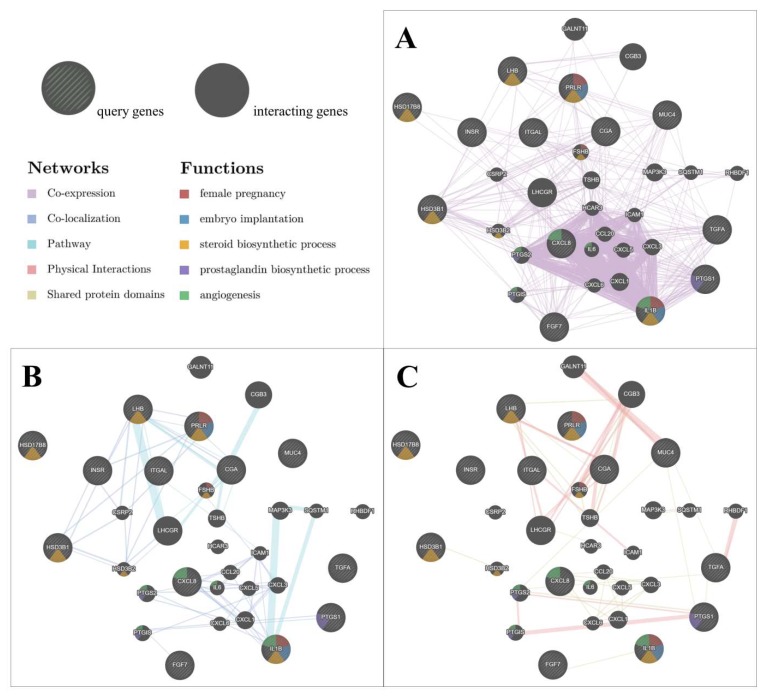
Gene interaction networks created in GeneMania for selected genes. The relations between the chosen genes based on their known participation in female reproduction and metabolism (query genes, striped circles) and additionally automatically generated genes mediating in the biological processes (interacting genes, non-striped circles). Colours on the circles indicate the contribution of the genes in the specific biological functions. The size of the circle indicates the importance of the gene in the specific interactions. The colour of the lines connecting the genes denotes the type of interaction: co-expression (**A**), co-localization and pathways (**B**), as well as physical interactions and shared protein domains (**C**), while the width of lines indicates the weight of interaction between genes (refer to the key).

Analysis of the interaction network was performed between 13 selected genes involved in metabolism, steroid and prostaglandin synthesis, interleukin and growth factor action, and embryo implantation. GeneMania was used to predict the relations between the chosen genes (query genes) in three different types of interaction as follows: co-expression (Figure 1A), co-localization and pathways (Figure 1B), as well as physical interactions and shared protein domains (Figure 1C). The analysis considered an additional 19 automatically generated genes, which were necessary to indicate the observed networks (interacting genes). Moreover, using different colours, we indicated the contribution of genes in specific biological functions related to female reproduction: female pregnancy (red), embryo implantation (blue), steroid biosynthetic process (yellow), prostaglandin biosynthetic process (purple), and angiogenesis (green; refer to the key in the Figure 1). Co-expression of the genes was found in 57 interactions and co-localization was observed in 41 interactions. In 21 cases, the genes participated in common pathways. Physical interactions were present in 37 cases, and in 19 cases interactions were based on the shared protein domains. The complete list of gene interactions is presented in Table S5.

### 2.2. Validation of the Microarray Results by Real-Time PCR (qPCR).

**Figure 2:**
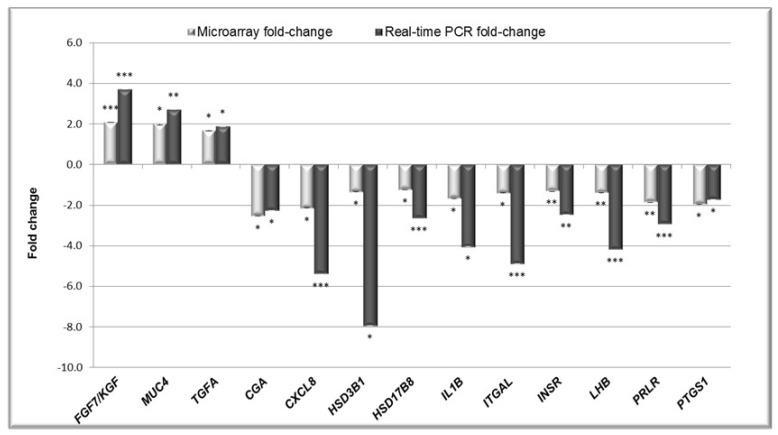
Real-time PCR validation of the microarray experiment. Light-grey bars represent fold changes for microarray data; dark-grey bars represent fold changes for qPCR data. Data are presented as means ± SEM from four different observations; * *p* < 0.05; ** *p* < 0.01; *** *p* < 0.001.

Real-time PCR method was used to verify the differential expression of 13 genes that were detected by the 4 × 44 Porcine (V2) Two-Colour Gene Expression Microarray kit (Agilent, Santa Clara, CA, USA). Only genes with an FC greater than 1.2 and *p*-values under 0.05 were selected. The protein products of selected genes were involved in processes important for endometrium functions during early pregnancy, such as metabolism of lipids, carbohydrates, and proteins (insulin receptor—*INSR*), gonadotropin metabolism and action (alpha polypeptide of glycoprotein hormones—*CGA*, luteinizing hormone beta polypeptide—*LHB*, prolactin receptor—*PRLR*), steroid hormone and prostaglandin synthesis and metabolism (hydroxy-delta-5-steroid dehydrogenase—*HSD3B1*, hydroxysteroid (17-beta) dehydrogenase 8—*HSD17B8*, prostaglandin-endoperoxide synthase 1—*PTGS1*), interleukin and growth factor action (C-X-C motif chemokine ligand 8—*CXCL8*, fibroblast growth factor 7—*FGF7*, interleukin 1 beta—*IL1B*, transforming growth factor alpha—*TGFA*), and embryo implantation (integrin, alpha L—*ITGAL*, mucin 4—*MUC4*). All changes in the gene expression determined by the microarray analysis were confirmed by qPCR (Figure 2).

## 3. Discussion

In mammals, conceptus implantation in the endometrium is of critical importance for reproductive success; in pigs this process takes place on days 15 to 16 of gestation. Consequently, physiological mechanisms and cellular signal pathways related to implantation have long been a research interest. The highest conceptus mortality in pigs occurs between days 12 to 30 of pregnancy (reviewed by Reference [1]). Establishing the interface between the developing embryos, appropriate remodelling of maternal endometrium, and uterine receptivity involves a number of complex signalling networks. Periodic expression of numerous genes essential for embryo survival and development occurs during implantation. In the present study, in-depth genomic analysis demonstrates for the first time, the effect of adiponectin on global gene expression in the porcine endometrium during implantation period. The obtained list of DE-genes was used to analyse specific genes ontologies, biological pathways, and possible interaction networks. In our present study, 1494 DE-genes were identified among which 1286 genes had FC values greater than 1.2. The expression of 560 genes was upregulated and 726 downregulated in the endometrium treated with adiponectin relative to the control group. It is important to note that, of the 13 genes selected for interaction analysis, six genes were directly involved in the steroid hormone biosynthesis or embryo implantation, two genes were engaged in the angiogenesis, and one gene was involved in the prostaglandin biosynthetic process.

The adiponectin system (adiponectin and its receptors) has been found in the uterus of many species, including pig, human, mouse, and rabbit [14,19,20,22]. Adiponectin concentrations in the porcine serum and uterine luminal fluid were the highest on days 15 to 16 and 27 to 28 of gestation, i.e., the beginning and the end of implantation period [23]. Only a few previous studies indicated adiponectin’s effect on endometrial gene expression; however, these concerned pre-determined genes and did not analyse the whole transcriptome. According to the study by Brochu-Gaudreau et al. [24], adiponectin regulates the endometrial expression of genes associated with placental formation, cyclooxygenase-2 (*COX-2*), vascular endothelial growth factor (*VEGF*), and peroxisome proliferator-activated receptor gamma (*PPARγ*). Our earlier study revealed that adiponectin modulates the expression of key enzymes in the synthesis of the steroids: steroidogenic acute regulatory protein (*StAR*), P450 side-chain cleavage enzyme (*CYP11A1*), and 3β-hydroxysteroid dehydrogenase (*HSD3B1*), as well as P4 and androstenedione (A4) secretion by the porcine uterus during early pregnancy [21]. Although the above studies have significantly helped us understand the role of adiponectin in the uterus, a genome-wide approach using a microarray analysis allows us to more efficiently investigate the effect of adiponectin on global gene expression in the endometrium during critical period for embryo survival and development. Understanding the processes/mechanisms that occur in the endometrium during the peri-implantation period will provide more avenues to influence conceptus growth and litter size. Moreover, the domestic pig is not only an economically important species but also a good experimental model for understanding human health and diseases. It is much more similar to humans than the more frequently-used laboratory rodents because of profound changes in rodents and other small mammals caused by redistribution of purifying selection [25,26]. Thus, results from the present study will also significantly contribute to a better understanding of human physiology.

In the constructed network of interactions, the gene encoding interleukin 1 beta (*IL1B*) was the node with the largest number of co-expression, co-localization, pathways, and functions. We demonstrated that *IL1B* gene expression was significantly downregulated in the porcine endometrium treated with adiponectin during implantation. This pro-inflammatory cytokine has been reported to play important roles in the implantation process, mediating conceptus–endometrial interactions in a number of mammalian species (for review see Reference [27]). During the period of implantation, the porcine embryos (and endometrium) produced IL1B [28,29]. Conceptus IL1B mRNA and protein expression is rapidly increased during porcine trophoblast elongation but rapidly declines immediately following the completion of the elongation process [29]. Therefore, IL1B was proposed as a candidate for initiating the cellular signalling pathway for the remodelling of conceptus and might be involved in the successful establishment of pregnancy in pigs. The result of the gene ontology analysis showed that adiponectin affects the expression of genes involved in the regulation of *cell proliferation* (GO:0042127), *negative regulation of cell proliferation* (GO:0008285), *cell proliferation* (GO:0008283), and *programmed cell death* (GO:0012501). Adiponectin decreased human endometrial stromal cell proliferation in dose- and time-dependent manners and caused cell death. Therefore, it is suggested that the adipokine is an anti-endometriosis factor [30]. This finding has been confirmed by subsequent studies by the same group of authors indicating the inhibitory effect of adiponectin on the proliferation of endometriotic stromal cells [31]. Evidence has revealed that adiponectin concentrations were decreased in the plasma and peritoneal fluid of women with endometriosis [32,33]. Plasma adiponectin concentrations were also decreased in genital cancers, such as oestrogen-related cancer as well as endometrial cancer [34,35]. Furthermore, high levels of circulating adiponectin are associated with reduced endometrial cancer risk [36]. The above studies correspond with findings reported by Cong et al. [37] implying the direct anti-proliferative impact of adiponectin on human endometrial cancer cell lines by inducing cell cycle arrest and apoptosis. On the other hand, the latest studies revealed that adiponectin significantly stimulated proliferation and suppressed apoptosis of porcine uterine luminal epithelial cells (LEc) which would enhance uterine receptivity for embryo implantation. The mentioned adiponectin-stimulated proliferation of LEc was related to activation of the phosphatidylinositol 3-kinase (PI3K) and mitogen-activated protein kinase signal transduction pathways [38]. This finding supports the hypothesis that adiponectin signalling is necessary for the establishment and maintenance of conceptus–uterine cross-talk during early pregnancy. However, further research is required to support the suggestion of adiponectin involvement in the implantation process and its utilization as prognostic markers and/or therapeutic targets in endometrial cancer.

The analysis of DE-genes indicated that adiponectin stimulated *MUC4* gene expression. Mucin 4 is a member of the family of membrane mucins and it is expressed at the apical surface of most epithelia including the endometrium [39,40]. Mucins are able to form gels and they are proposed to protect the surface of most epithelia [41]. Studies carried out in various species imply that MUC4 is involved in embryo implantation. In the normal cycling rodent, uterine luminal epithelium (LE) expression of MUC4 is high during oestrous stages corresponding with high oestrogen concentrations and low during stages characterised by high P4 levels [42]. In rats with the invasive type of implantation, loss of MUC4 in the uterine LE occurs at the beginning of the period of receptivity for implantation. Mucin 4 has anti-adhesive properties; therefore, it is assumed to make a barrier in the uterus to block blastocyst implantation during the pre-receptive period. The absence of MUC4 during implantation implies that this glycoprotein must be lost from the apical surface of the rat uterine LE to create the receptive state for uterine implantation [43]. However, in gilts with the non-invasive, epitheliochorial implantation, endometrial MUC4 expression increased during the period of trophoblastic attachment to the uterine luminal surface. It is suggested that maintenance of MUC4 on the uterine surface epithelium could play a role in modulating the proteolytic activity of porcine conceptuses to prevent erosion of the glandular and surface epithelia [44]. It would be interesting to check whether the expression of MUC4 in the porcine uterine LE, similar to the regulation proposed in the rodents, is regulated by steroid hormones. It would also be interesting to explain the mechanism of adiponectin action in this regulation. Our previous studies indicated the effect of adiponectin on steroids production and steroid hormones on adiponectin expression in the porcine uterus during early pregnancy [21,45,46]. In the current studies, treatment with adiponectin inhibited the expression of *HSD3B1* which is responsible for P4 and A4 production, and *HSD17B8* which selectively catalyses the conversion of E2 to biologically less-active oestrone [47]. Therefore, it cannot be excluded that adiponectin affects luminal uterine expression of MUC4 through steroid hormones or the other way around.

Adiponectin also affects the following groups of genes that encode *biological adhesion* (GO:0022610), *cell adhesion* (GO:0007155), *cell–cell adhesion* (GO:0098609), and *cell–matrix adhesion* (GO:0007160). In all of these processes, the *ITGAL* gene is involved. The results from the analysis of DE-genes revealed that adiponectin inhibited the expression of *ITGAL* coding integrin subunit alpha. In pigs, implantation follows an extended pre-attachment period of 8 to 15 days [48]. Adhesion and signal transduction events that occur during prolonged periods of apposition and attachment include the modulation of number adhesion molecules. Conceptus attachment to the uterine LE does not take place until hormonally regulated events change the non-adhesive (pre-receptive) uterine wall to mutually adhesive receptive epithelial surface [49]. Integrins are a family of glycoconjugates that are heterodimeric intrinsic membrane proteins composed of non-covalently linked α and β subunits that interact with various extracellular matrix (ECM) components and cell adhesion molecules [50]. Integrins play a dominant role in the attachment and implantation of the blastocyst to the uterine LE. They have been recognised as critical molecules involved in the implantation adhesion cascade that possess the ability to bind ECM and other ligands to mediate adhesion, migration, invasion, and cause cytoskeletal reorganization and transduce cellular signals [51]. In the pig, the constitutive and cycle-dependent expression of integrin subunits including α1, α3, α4, α5, αv, β1, β3, and β5 has been found on the surface of uterine LE and conceptuses. Of these, the α4, α5, αv, β1, β3, and β5 subunits were indicated at sites of initial attachment between uterine LE and trophectoderm on days 12 to 15 of gestation. During the peri-implantation period, P4 stimulated the expression of integrins that may partially define “the window of implantation” in this species [49]. It seems that the endometrium is the only tissue known to exhibit hormone-dependent integrin expression. In humans, three integrins are considered as markers of uterine receptivity for implantation and occur when the uterus is influenced by P4. The expression of αvβ3 correlates with conceptus attachment and loss of the α4 integrin subunits is coincident with closure of the window of receptivity. Furthermore, the presence of both αvβ3 and α4β1 on the apical surface of uterine LE suggests a role of these integrins in initial trophectoderm–LE interaction during implantation [52]. It is possible that adiponectin also participates in the implantation adhesion cascade. As a continuation of the present work, we intend to investigate the expression patterns of integrins in the porcine endometrium during the peri-implantation period under the influence of adiponectin.

## 4. Materials and Methods

### 4.1. Experimental Animals and Tissue Collection

Four mature gilts (Large White × Polish Landrace; 7 to 8 months of age, body weight of 120 to 130 kg) descended from a private breeding farm were used in the study. The gilts used in the study were on days 15 to 16 of pregnancy. Females were monitored daily for oestrus behaviour in the presence of an intact boar. The day of onset of the second oestrus was designated as day 0 of the oestrous cycle. Insemination was performed on days 1 to 2 of the oestrous cycle. Uteri collected after slaughter from pregnant gilts were immediately placed in ice-cold PBS supplemented with 100 IU/mL penicillin and 100 µg/mL streptomycin and transported to the laboratory on ice within 1 h for in vitro explant tissue culture. Pregnancy was confirmed by the presence of conceptuses. All slices of the endometrium on days 15 to 16 of gestation were collected at the implantation sites. The experiments were carried out in accordance with the ethical standards of the Animal Ethics Committee at the University of Warmia and Mazury in Olsztyn (Ethical approval: 113/2011/DTN; 14.12.2011).

### 4.2. Endometrial Explant Culture

Uteri collected from gilts on days 15 to 16 of pregnancy were washed three times in sterile PBS. Endometrial explant cultures were performed according to Smolinska et al. [21]. The endometrial tissues from the uterine horns were dissected and cut into small, irregular slices (3-mm thick, 100 mg ± 10%) and then washed three times in medium M199 (Sigma–Aldrich Co., Saint Louis, MO, USA). Individual endometrial explants were placed into culture glass vials with 2-mL medium M199 containing 0.1% BSA (MP Biomedicals, Santa Ana, CA, USA), 5% dextran/charcoal-stripped new-born calf serum (Sigma–Aldrich Co.), penicillin (100 IU/mL), and streptomycin (100 μg/mL). The tissue cultures were preincubated in a shaking water bath for 2 h at 37 °C in an atmosphere of 95% O_2_ and 5% CO_2_. After preincubation, to examine the impact of adiponectin on the global gene expression in the endometrium, the slices were treated for 24 h with recombinant human adiponectin (10 μg/mL, BioVendor, Brno, Czech Republic). The doses of adiponectin were established based on Ledoux et al. [53] and Maleszka et al. [17,18]. Control slices were incubated without any treatment. All cultures were performed in duplicates in four independent experiments (*n* = 4). The viability of tissue explants was monitored by measuring lactate dehydrogenase (LDH) activity in medium at 2 h of preincubation as well as at the end of the treatment period. The release of LDH was performed using a Liquick Cor-LDH kit (Cormay, Lomianki, Poland) following the manufacturer’s instructions. The activity of LDH during the culture of tissue explants was compared to its activity in medium obtained after destruction of endometrial cells by homogenization (positive control for causing cell death and the maximal release of LDH). Mean activity of LDH in cultured slices after treatment period was 55.1 ± 4.5 U/L (1.8% of maximal release of LDH after total endometrial cell destruction).

### 4.3. Total RNA Isolation and Quality Control

Total RNA was isolated from 8 endometrium samples (4 endometrium slices treated with adiponectin and 4 control endometrium) using the RNeasy Mini Kit (Qiagen, Germantown, MD, USA). The DNA was removed by on-column DNase I digestion as recommended by Qiagen (Germantown, MD, USA). The quality of the RNA was checked using an Agilent 2100 Bioanalyzer (Agilent Technologies, Santa Clara, CA, USA) and an RNA 6000 Nano Assay Kit (Agilent Technologies, Santa Clara, CA, USA). The RNA quantity was determined spectrophotometrically (Infinite 200 PRO plate reader with NanoQuant plates, Tecan Group, Mannedorf, Switzerland). In order to ensure the most reliable results from the microarray and quantitative real-time PCR (qPCR) validation experiments, only samples with an RNA integrity number (RIN) above 8 were used. The RNA samples were stored at −80 °C.

### 4.4. Microarray Hybridization

The porcine genome microarrays were purchased from Agilent Technologies (Porcine (V2) Gene Expression Microarray 4 × 44; Agilent Technologies, Santa Clara, CA, USA). The microarray experiment was performed as previously described by Szeszko et al. [54] and Dobrzyn et al. [55]. The RNA from the four endometrium slices treated with adiponectin and four control endometria was used to generate Cyanine-3 (Cy3) and Cyanine-5 (Cy5) labelled cRNA with a Low Input Quick Amp, Two-Colour kit (Agilent Technologies, Santa Clara, CA, USA) according to the manufacturer’s instructions. Quantification of cRNA and cyanine dye incorporation were measured with Infinite 200 PRO plate reader with NanoQuant plates (Tecan Group, Mannedorf, Switzerland). For each microarray, 825 ng of the Cy3 and 825 ng of the Cy5 labelled cRNA were mixed together. The dual-labelled cRNA samples (obtained from treated and control samples) were fragmented and placed on each array (*n* = 4, one slide) in a balanced block design with dye swaps (Figure S1). To discount the dye bias effect observed in the dual-colour experiments, the study design included the alternate use of both dyes (dye swap), namely, in two microarrays the control probes were dyed by Cy-3 and the adiponectin-treated samples with Cy-5, whereas in another two microarrays, the control probes were dyed with Cy-5 and the adiponectin-treated with Cy-3. Hybridization was carried out according to the manufacturer’s instructions at 60 °C for 17 h in an Agilent hybridization oven. After hybridization, slides were washed and scanned on an Agilent’s High-Resolution C Microarray Scanner at a 5 μm resolution. In the next step, the images’ raw data was acquired by Feature Extraction Software (Agilent Technologies, Santa Clara, CA, USA) for filtering of outlier spots, background subtraction from features, dye normalizations (linear and LOWESS), and expression data extraction and detailed analysis. All the microarray raw data files were uploaded on the National Centre for Biotechnology Information Gene Expression Omnibus (GEO) server (https://www.ncbi.nlm.nih.gov/geo/; accession number: GSE122400).

### 4.5. Bioinformatic Analysis

In order to identify DE-genes between control and treated by adiponectin endometrial explants, FC rule was used. The FC of DE-genes was obtained by analysis of previously generated expression data using the GeneSpring GX 12 software (Agilent Technologies, Santa Clara, CA, USA). The results obtained through the normalization of fluorescence intense were log-transformed, and then analysed using Student’s *t*-test. The threshold set for up- and downregulated genes was if they had an FC greater than 1.2 and a *p*-value ≤ 0.05. The 1.2 FC cut-off was selected based on previous studies, which used the microarray technology [54,55,56,57]. To compare the normalized fluorescence intense for adiponectin treated versus control samples Student’s *t*-test was applied. The FC was estimated based on the mean values of the treated/control gene expression levels of four biological replicates. Additionally, the Basic Local Alignment Search Tool (BLAST) was used to align the unknown gene probe sequences with the whole porcine transcriptome deposited in the database, which resulted in manual enrichment of the DE-genes list. For DE-genes represented on the Agilent’s Porcine V2 microarray by multiple probes, the FC mean value for all the probes was calculated.

#### 4.5.1. Gene Ontology Analysis

The functional analysis of these DE-genes was performed by utilizing the DAVID tool (http://david.abcc.ncifcrf.gov) to explore functional class scoring in the resulting gene list by means of GO term enrichment analysis [58]. Performed gene ontology analysis was limited to *Sus scrofa*, furthermore the genome of this species was used as a background. The level of significance was determined by the modified Fisher’s exact test, incorporating gene-enrichment analysis, which modifies the original *p*-value by the threshold of maximum probability (EASE Score Threshold, *p* ≤ 0.1).

#### 4.5.2. Biological Pathways Analysis

The biological pathways analysis was also conducted based on the DE-genes resulting list by the DAVID tool. The software was able to categorize DE-transcripts into different biological functions and pathways, using information from each individual gene and computing a total over-representation value for each pathway represented in the KEGG. Like in the case of GO analysis, this analysis was limited to *Sus scrofa*, and also a genome of this species was used as a background. Values at *p* ≤ 0.05 were considered statistically significant.

#### 4.5.3. Interaction Network of Differentially Expressed Genes

An interaction network between DE-genes was performed with the GeneMania Prediction Server [59]. From the DE-genes list, there were 13 genes selected with fold change > 1.2: *CGA*, *FGF7*, *HSD3B1*, *HSD17B8*, *IL1B*, *CXCL8*, *INSR*, *LHB*, *PRLR*, *PTGS1*, *TGFA*, *ITGAL*, and *MUC4*. Products of the selected genes are known to be involved in the regulation of female reproduction and metabolism. GeneMania was used to predict the functions of selected genes and define possible interaction networks between them based on known interplay such as co-expression, co-localization, genetic interactions, signalling pathways, physical interactions, and shared protein domains.

### 4.6. Real-Time PCR Validations

Complementary DNA (cDNA) synthesis and qPCR were conducted by using the same total RNA (*n* = 4, for both, the treated and the control samples) as for the microarray experiment, as described previously by Szeszko et al. [54] and Dobrzyn et al. [55]. One microgram of total RNA was reverse transcribed into cDNA using the Omniscript RT Kit (Qiagen, Germantown, MD, USA) in a total volume of 20 μL with 0.5 μg oligo(dt)15 Primer (Roche, Basel, Switzerland). The reverse transcription reaction was carried out at 37 °C for 1 h and finally were heat-inactivated by incubation at 93 °C for 5 min. Real-time PCR analyses were performed in two technical repeats for each sample using a 7300 Real-Time PCR system and Power SYBR^®^ Green PCR Master Mix (Life Technologies, Carlsbad, CA, USA). Real-Time PCR reaction mix included: 20 ng cDNA, 1 of 13 primer pairs (forward and reverse) at various concentrations, 12.5 μL SYBR^®^ Green PCR Master Mix (Applied Biosystems, Waltham, MA, USA), and RNase free water to a final volume of 25 μL. The specificity of amplification was tested at the end of the qPCR by melting-curve analysis. Product purity was confirmed by agarose gel electrophoresis. The negative controls were performed in which cDNA was substituted by water, or reverse transcription was not performed before qPCR. The negative controls gave non-detectable signals in all samples, confirming the high specificity of the assay. To validate microarray results by qPCR, we chose the same thirteen genes as in the interaction network analysis. Selected forward and reverse primers sequences, qPCR conditions, and concentrations of primers are presented in Table 2. Calculation of the relative expression levels of the genes was conducted based on the comparative cycle threshold method (ΔΔCT) and normalised using the geometrical means of reference gene expression levels: β-actin (*ACTB*) and glyceraldehyde 3-phosphate dehydrogenase (*GAPDH*). During the preliminary studies it was found that the expression of both constitutively expressed genes did not differ significantly between the treated and control sample; therefore, these genes were suitable housekeeping genes for this experiment. Presented data are means ± SEM from four different biological replicates. Differences between treated samples and controls were analysed by one-way ANOVA followed by the least significant differences (LSD) post-hoc test using Statistica Software (StatSoft Inc., Tulsa, OK, USA). Values of *p* < 0.05 were considered as statistically significant. The Ct values for all non-template controls were under the detection threshold.

## 5. Conclusions

Conceptuses loss during early gestation can be a critical determinant of litter size. Identification of genes/proteins of many factors involved in different molecular pathways may contribute to successful pregnancy establishment and embryo development. This is the first global microarray-based study analysing differentially regulated genes by adiponectin in the endometrium of pregnant gilts during the implantation period. The present work indicates that adiponectin affects a number of genes and processes that are engaged in i.a. cell proliferation, programmed cell death, immune system response, metabolism of lipids, carbohydrates, and proteins, steroid hormone and prostaglandin synthesis and metabolism, interleukin and growth factor action, and cell adhesion. The above processes are critical for reproductive success; therefore, adiponectin may be an important regulator of implantation in pigs. Nevertheless, understanding the relationship between transcriptome and proteome, and further functional studies are required to explain the role and mechanism of adiponectin action in the porcine endometrium during peri-implantation period.

## Figures and Tables

**Table 1:** Analysis of pathways significantly enriched in the list of differentially expressed genes.

KEGG Pathway Analysis			
Analysis Name	Gene Number	*p*-Value	Altered Genes
Pathways in cancer	21	1.00 × 10^−1^	*CEBPA*, *STAT5A*, *CBL*, *CXCL8*, *NFKBIA*, *GNG12*, *APPL1*, *CBLB*, *CDKN1A*, *EP300*, *GNAQ*, *CXCR4*, *NCOA4*, *TGFA*, *PIK3R5*, *LAMC2*, *RARB*, *NOS2*, *CRK*, *CHUK*, *TRAF3*
Cytokine–cytokine receptor interaction	18	3.60 × 10^−3^	*CSF3*, *IL9*, *CXCL8*, *CCL28*, *TNFRSF4*, *CCL4*, *IFNAR1*, *TNFRSF1B*, *TNFRSF11B*, *PRLR*, *TNFSF13B*, *IL10RB*, *CXCR4*, *IL4R*, *IL13RA1*, *IFNGR2*, *BMPR1A*
Jak–STAT signalling pathway	16	1.60 × 10^−4^	*CSF3*, *STAT5A*, *SOCS1*, *IL9*, *SOCS4*, *IL24*, *IFNAR1*, *EP300*, *PRLR*, *IL10RB*, *IL4R*, *PIK3R5*, *JAK3*, *IL13RA1*, *IFNGR2*
Regulation of actin cytoskeleton	15	1.40 × 10^−2^	*ITGAL*, *SSH1*, *DIAPH1*, *SSH2*, *ARPC5*, *GNG12*, *PPP1CC*, *NCKAP1*, *ARPC1B*, *ITGB7*, *PIK3R5*, *MSN*, *PIP4K2A*, *CRK*, *MYLK*
HTLV-I infection	15	8.30 × 10^−2^	*ITGAL*, *TLN1*, *KAT2B*, *STAT5A*, *NFKBIA*, *MYBL2*, *ATM*, *SLA-8*, *CDKN1A*, *EP300*, *ETS1*, *PIK3R5*, *JAK3*, *NFATC3*, *CHUK*
Transcriptional misregulation in cancer	14	3.80 × 10^−3^	*CEBPA*, *CEBPB*, *KMT2A*, *CEBPE*, *LDB1*, *CCNT1*, *ELANE*, *CXCL8*, *ATM*, *HHEX*, *CDKN1A*, *ITGB7*, *GOLPH3L*
Herpes simplex infection	14	1.60 × 10^−2^	*CSNK2A2*, *CFP*, *EP300*, *HNRNPK*, *TAF5*, *EIF2S1*, *NFKBIA*, *HCFC2*, *PPP1CC*, *IFNGR2*, *CHUK*, *IFNAR1*, *SLA-8*, *TRAF3*
Viral carcinogenesis	14	1.90 × 10^−2^	*LOC100156127*, *KAT2B*, *UBE3A*, *STAT5A*, *NFKBIA*, *PMAIP1*, *SLA-8*, *CDKN1A*, *HNRNPK*, *EP300*, *PIK3R5*, *JAK3*, *LOC100621389*, *TRAF3*
Insulin signalling pathway	13	2.40 × 10^−3^	*SOCS1*, *CBL*, *ACACA*, *FBP1*, *RPS6KB1*, *SOCS4*, *PPP1CC*, *CBLB*, *PYGM*, *PYGL*, *PIK3R5*, *CRK*, *INSR*
Chemokine signalling pathway	13	2.30 × 10^−2^	*AMCF-II*, *CXCR4*, *CXCL2*, *CXCL8*, *NFKBIA*, *PIK3R5*, *FOXO3*, *GNG12*, *JAK3*, *CCL4*, *CCL28*, *CRK*, *CHUK*
Epstein–Barr virus infection	12	7.90 × 10^−2^	*CSNK2A2*, *ITGAL*, *CDKN1A*, *EP300*, *IL10RB*, *NFKBIA*, *PIK3R5*, *JAK3*, *TNFAIP3*, *CHUK*, *SLA-8*, *TRAF3*
Measles	11	1.80 × 10^−2^	*CSNK2A2*, *EIF2S1*, *STAT5A*, *NFKBIA*, *PIK3R5*, *JAK3*, *MSN*, *TNFAIP3*, *IFNGR2*, *CHUK*, *IFNAR1*
Ubiquitin-mediated proteolysis	11	2.00 × 10^−2^	*CUL5*, *CBLB*, *UBE3A*, *UBR5*, *WWP1*, *SOCS1*, *CBL*, *RHOBTB1*, *KEAP1*, *LOC780419*, *TRIP12*
Prolactin signalling pathway	10	2.90 × 10^−4^	*CGA*, *PRLR*, *STAT5A*, *SOCS1*, *IRF1*, *SOCS4*, *PIK3R5*, *FOXO3*, *LHB*, *CSN2*
Salmonella infection	10	1.50 × 10^−3^	*ARPC1B*, *CXCL2*, *PKN2*, *CXCL8*, *ARPC5*, *NOS2*, *DYNC1H1*, *CASP1*, *CCL4*, *IFNGR2*
NF-kappa B signalling pathway	10	3.10 × 10^−3^	*CSNK2A2*, *TNFSF13B*, *LY96*, *CXCL8*, *NFKBIA*, *TNFAIP3*, *CCL4*, *ATM*, *CHUK*, *TRAF3*
TNF signalling pathway	10	1.20 × 10^−2^	*LOC100736836*, *TNFRSF1B*, *CEBPB*, *CXCL2*, *NFKBIA*, *PIK3R5*, *TNFAIP3*, *CHUK*, *TRAF3*
Hepatitis C	10	3.30 × 10^−2^	*CDKN1A*, *EIF2S1*, *IRF1*, *CXCL8*, *NFKBIA*, *PIK3R5*, *CHUK*, *IFNAR1*, *PPP2R2A*, *TRAF3*
Osteoclast differentiation	10	4.30 × 10^−2^	*CYLD*, *TNFRSF11B*, *CTSK*, *SOCS1*, *NFKBIA*, *PIK3R5*, *IFNGR2*, *SIRPA*, *CHUK*, *IFNAR1*
ErbB signalling pathway	9	7.20 × 10^−3^	*CBLB*, *CDKN1A*, *STAT5A*, *CBL*, *TGFA*, *PIK3R5*, *RPS6KB1*, *ABL2*, *CRK*
Toll-like receptor signalling pathway	9	2.10 × 10^−2^	*CTSK*, *LY96*, *CXCL8*, *NFKBIA*, *PIK3R5*, *CCL4*, *CHUK*, *IFNAR1*, *TRAF3*
Chagas disease (American trypanosomiasis)	9	2.90 × 10^−2^	*GNAQ*, *CD247*, *CXCL8*, *NFKBIA*, *PIK3R5*, *NOS2*, *IFNGR2*, *CHUK*, *PPP2R2A*
Insulin resistance	9	3.80 × 10^−2^	*RPS6KA3*, *PYGM*, *PYGL*, *NFKBIA*, *PIK3R5*, *RPS6KB1*, *PPP1CC*, *SLC27A2*, *INSR*
Toxoplasmosis	9	4.10 × 10^−2^	*LY96*, *IL10RB*, *SOCS1*, *NFKBIA*, *LAMC2*, *PIK3R5*, *NOS2*, *IFNGR2*, *CHUK*
AMPK signalling pathway	9	4.70 × 10^−2^	*EEF2K*, *ACACA*, *FBP1*, *RAB14*, *PIK3R5*, *RPS6KB1*, *FOXO3*, *INSR*, *PPP2R2A*
Chronic myeloid leukemia	8	6.50 × 10^−3^	*CBLB*, *CDKN1A*, *STAT5A*, *CBL*, *NFKBIA*, *PIK3R5*, *CRK*, *CHUK*
Ribosome biogenesis in eukaryotes	8	1.80 × 10^−2^	*CSNK2A2*, *RN18S*, *MPHOSPH10*, *NOP58*, *WDR3*, *NOP56*, *BMS1*, *GNL3*
HIF-1 signalling pathway	8	5.20 × 10^−2^	*PDK1*, *CDKN1A*, *EP300*, *PIK3R5*, *RPS6KB1*, *NOS2*, *IFNGR2*, *INSR*
Bacterial invasion of epithelial cells	7	3.40 × 10^−2^	*ARPC1B*, *CBLB*, *CBL*, *PIK3R5*, *ARPC5*, *CRK*, *ARHGAP10*
Peroxisome	7	4.80 × 10^−2^	*ACSL1*, *NUDT12*, *NOS2*, *SLC27A2*, *ACSL3*, *CROT*, *SOD2*
Small cell lung cancer	7	5.90 × 10^−2^	*NFKBIA*, *LAMC2*, *PIK3R5*, *NOS2*, *RARB*, *CHUK*, *TRAF3*
Phosphatidylinositol signalling system	7	9.40 × 10^−2^	*MTMR14*, *PI4KA*, *PIK3R5*, *DGKH*, *INPP4A*, *PIP4K2A*, *INPP5B*
Systemic lupus erythematosus	7	9.80 × 10^−2^	*LOC100156127*, *LOC100157763*, *LOC100158121*, *ELANE*, *LOC100154071*, *LOC100153329*, *LOC100621389*
Pertussis	6	7.50 × 10^−2^	*AMCF-II*, *LY96*, *IRF1*, *CXCL8*, *NOS2*, *CASP1*
NOD-like receptor signalling pathway	5	5.10 × 10^−2^	*CXCL8*, *NFKBIA*, *TNFAIP3*, *CASP1*, *CHUK*
Ovarian steroidogenesis	5	5.40 × 10^−2^	*CGA*, *PLA2G4A*, *HSD3B1*, *LHB*, *INSR*
Malaria	5	5.80 × 10^−2^	*CSF3*, *ITGAL*, *SELP*, *CXCL8*
Acute myeloid leukemia	5	6.70 × 10^−2^	*CEBPA*, *STAT5A*, *PIK3R5*, *RPS6KB1*, *CHUK*
Pentose phosphate pathway	4	1.10 × 10^−2^	*FBP1*, *TKT*, *RPIA*, *PRPS2*
Intestinal immune network for IgA production	4	9.50 × 10^−2^	*TNFSF13B*, *CXCR4*, *ITGB7*, *CCL28*
Fatty acid biosynthesis	3	1.40 × 10^−2^	*ACSL1*, *ACACA*, *ACSL3*
Glycosaminoglycan biosynthesis-chondroitin sulphate/dermatan sulphate	3	5.10 × 10^−2^	*CSGALNACT2*, *CHST3*, *CHSY1*
Nicotinate and nicotinamide metabolism	3	9.40 × 10^−2^	*ENPP1*, *NUDT12*, *NMRK1*
Dorso-ventral axis formation	3	9.40 × 10^−2^	*CPEB2*, *ETS1*, *CPEB4*

**Table 2:** Primers used for the validation of microarray results.

Gene Symbol	Primers Sequences	Reaction Conditions		Primer (nM)	Target Sequence Accession Number	References
*CGA*	F: 5′-CTCCAGAGCGTACCCAACTC-3′ R: 5′-ACTGTGGCCTTGGTAAATGC-3′	Activation: 50 °C, 30 min;	40 cycles	500 nM	XM_005659277.1	[60]
		95 °C 15 min,				
		1. Denaturation: 94 °C, 15 s				
		2. Annealing: 55 °C, 30 s				
		3. Extension: 72 °C, 30 s				
		77 °C, 15 s				
*CXCL8*	F: 5′-GGCAGTTTTCCTGCTTTCT-3′ R: 5′-CAGTGGGGTCCACTCTCAAT-3′	Activation: 95 °C, 15 min	40 cycles	400 nM	X61151.1	[61]
		1. Denaturation: 94 °C, 15 s				
		2. Annealing: 58 °C, 30 s				
		3. Extension: 72 °C, 30 s				
*FGF7/KGF*	F: 5′-GCTTCCACATTATCTGTCTGGTG-3′ R: 5′-GTCCCTTTGACTTTGCCTCG-3′	Activation: 95 °C, 10 min	40 cycles	500 nM	AF217463.1	This study
		1. Denaturation: 95 °C, 15 s				
		2. Annealing: 60 °C, 1 min				
		3. Extension: 72 °C, 1 min				
*HSD3B1*	F: 5′-AGGTTCGCCCGCTCATC-3′ R: 5′-CTGGGCACCGAGAAATACTTG-3′	Activation: 95 °C, 10 min	40 cycles	300 nM	NM_001004049.1	[62]
		1. Denaturation: 95 °C, 15 s				
		2. Annealing: 61 °C, 1 min				
		3. Extension: 72 °C, 1 min				
*HSD17B8*	F: 5′-TTCTGCTCCGCATGTCTGAAG-3′ R: 5′-CCATGTTTCCCACCTTCCCTA-3′	Activation: 95 °C, 10 min	40 cycles	500 nM	NM_001130730.1	This study
		1. Denaturation: 95 °C, 15 s				
		2. Annealing: 60 °C, 1 min				
		3. Extension: 72 °C, 1 min				
*IL1B*	F: 5′-TGCCAACGTGCAGTCTATGG-3′ R: 5′-TGGGCCAGCCAGCACTAG-3′	Activation: 95 °C, 10 min	40 cycles	100 nM	NM_214055	[29]
		1. Denaturation: 95 °C, 15 s				
		2. Annealing: 60 °C, 1 min				
		3. Extension: 72 °C, 1 min				
*INSR*	F: 5′-AAACGCCAGGGACATCGTCAAGG-3′ R: 5′-CCGCAGGGAACGCAGGTAACTCT-3′	Activation: 95 °C-10 min	40 cycles	200 nM	XM_005654749.1	[63]
		1. Denaturation: 95 °C, 15 s				
		2. Annealing: 60 °C, 1 min				
		3. Extension: 72 °C, 1 min				
*ITGAL*	F: 5′-CTTGTCGAGCTGAAGGCTGA-3′ R: 5′-TTCCTGGTCCTTGGTGAGGA-3′	Activation: 95 °C, 10 min	40 cycles	500 nM	NM_001044608.1	This study
		1. Denaturation: 95 °C, 15 s				
		2. Annealing: 60 °C, 1 min				
		3. Extension: 72 °C, 1 min				
*LHB*	F: 5′-TTCACCACCAGCATCTGTGC-3′ R: 5′-AAGAGGAGGCCTGGGAGTAG-3′	Activation: 95 °C, 10 min	40 cycles	500 nM	XM_005664700.1	This study
		1. Denaturation: 95 °C, 15 s				
		2. Annealing: 60 °C, 1 min				
		3. Extension: 72 °C, 1 min				
*MUC4*	F: 5′-GATGCCCTGGCCACAGAA-3′ R: 5′-TGATTCAAGGTAGCATTCATTTGC-3′	Activation: 95 °C, 10 min	40 cycles	500 nM	NM_001206344.2	[64]
		1. Denaturation: 95 °C, 15 s				
		2. Annealing: 60 °C, 1 min				
*PRLR*	F: 5′-CCAGATACCTAATGACTTCTCAATG-3′ R: 5′-TCCAACAGATGGGTGTCAAA-3′	Activation: 50 °C, 30 min;	40 cycles	500 nM	NM_214084	[63]
		95 °C, 15 min				
		1. Denaturation: 94 °C, 15 s				
		2. Annealing: 55 °C, 30 s				
		3. Extension: 72 °C, 30 s				
		77 °C, 15 s				
*PTGS1*	F: 5′-CAACACTTCACCCACCAGTTCTTC-3′ R: 5′-TCCATAAATGTGGCCGAGGTCTAC-3′	Activation: 95 °C, 10 min	40 cycles	500 nM	AF207823.1	[65]
		1. Denaturation: 95 °C, 15 s				
		2. Annealing: 60 °C, 1 min				
		3. Extension: 72 °C, 1 min				
*TGFA*	F: 5′-CGCGCTGGGTATCTTGTTG-3′ R: 5′-GTGGGAATCTGGGCAGTCAT-3′	Activation: 50 °C, 2 min;	40 cycles	200 nM	NM_214251.1	[66]
		95 °C, 10 min				
		1. Denaturation: 95 °C, 3 s				
		2. Annealing: 60 °C, 30 s				
		3. Extension: 72 °C, 1 min				
*ACTB*	F: 5′-ACATCAAGGAGAAGCTCTGCTACG-3′ R: 5′-GAGGGGCGATGATCTTGATCTTCA-3′	Activation: 95 °C, 10 min	40 cycles	500 nM	U07786	[67]
		1. Denaturation: 95 °C, 15 s				
		2. Annealing: 61 °C, 1 min				
		3. Extension: 72 °C, 1 min				
*GAPDH*	F: 5′-CCTTCATTGACCTCCACTACATGG-3′ R: 5′-CCACAACATACGTAGCACCAGCATC-3′	Activation: 95 °C, 10 min	40 cycles	500 nM	U48832	[68]
		1. Denaturation: 95 °C, 15 s				
		2. Annealing: 59 °C, 1 min				

## Supplementary Materials

Supplementary materials can be found at https://www.mdpi.com/1422-0067/20/6/1335/s1.
