# Supplementary material for: Transcriptomic Analysis of Porcine Endometrium during Implantation after In Vitro Stimulation by Adiponectin
Source: Int J Mol Sci. 2019 Mar 16;20(6):1335. doi: 10.3390/ijms20061335 (PMC6470965; doi:10.3390/ijms20061335)
Supplement: Supplementary file 1 [file ijms-20-01335-s001.zip › SF.1..pdf]

Supplementary Table 1

List of differentially expressed genes

ProbeName	Official Gene Symbol	Fold-change	P-value	Regulation	Description
A_72_P196792		3,9660	0,0441	up	354318 MARC 1PIG Sus scrofa cDNA 5', mRNA sequence
A_72_P222917		3,2637	0,0138	up	1140898 MARC 4PIG Sus scrofa cDNA 3', mRNA sequence
A_72_P358918		3,2505	0,0453	up	MI-P-AY0-ncm-b-03-0-UI.s1 MI-P-AY0 Sus scrofa cDNA clone MI-P-AY0-ncm-b-03-0-UI 3', mRNA sequence
A_72_P350158		2,8920	0,0423	up	Pig DNA sequence from clone CH242-171P20 on chromosome X, complete sequence
A_72_P337643		2,8861	0,0078	up	gdtca_Cluster4852 Developing mandibular deciduous molars of the miniature pig cDNA library Sus scrofa cDNA, mRNA sequence
A_72_P408928		2,8554	0,0332	up	Sus scrofa mRNA, clone:LNG010043B04, expressed in lung.
A_72_P401428		2,8475	0,0269	up	MI-P-A2-aey-d-11-1-UM.s1 MI-P-A2 Sus scrofa cDNA clone MI-P-A2-aey-d-11-1-UM 3', mRNA sequence
A_72_P104946		2,8184	0,0456	up	Pig DNA sequence from clone XX-BE284E13 on chromosome 11, complete sequence
A_72_P271794		2,7767	0,0375	up	AJ683600 CSEQRAN04 Sus scrofa cDNA clone C0001802_I21, mRNA
A_72_P324303		2,6549	0,0373	up	AJ957011 KN404_2 Sus scrofa cDNA clone C0007750m18 3', mRNA
A_72_P373223	EEA1	2,6245	0,0409	up	Sus scrofa early endosome antigen 1 (EEA1), mRNA
A_72_P320608		2,5613	0,0348	up	1103584 MARC 4PIG Sus scrofa cDNA 5', mRNA sequence
A_72_P111001		2,5387	0,0129	up	UMC-p6mm3-008-b02 6mm ovarian follicle p6mm Sus scrofa cDNA 3', mRNA sequence
A_72_P338613		2,5273	0,0453	up	346982 MARC 1PIG Sus scrofa cDNA 5', mRNA sequence
A_72_P005621		2,5069	0,0376	up	1000485 MARC 4PIG Sus scrofa cDNA 3', mRNA sequence
A_72_P081726		2,4986	0,0402	up	Sus scrofa mRNA, clone: UTR010040A09, expressed in uterus
A_72_P159361		2,4869	0,0473	up	Rep: DSPP600 - Sus scrofa (Pig), partial (3%) [TC572246]
A_72_P218717		2,4406	0,0208	up	Rnca19b_m21.y1 nca Sus scrofa cDNA 5', mRNA sequence
A_72_P415663		2,4166	0,0295	up	Pig DNA sequence from clone XX-BE284E13 on chromosome 11, complete sequence
A_72_P279359		2,4045	0,0309	up	AJ659296 KN277 Sus scrofa cDNA clone C0005216_M17, mRNA sequence
A_72_P099131		2,3998	0,0343	up	AJ964668 KN404_2 Sus scrofa cDNA clone C0007758n19 5', mRNA sequence
A_72_P267581	LOC100516932	2,3904	0,0321	up	Sus scrofa keratin-associated protein 4-12-like (LOC100516932), mRNA
A_72_P316698	LOC100626841	2,3579	0,0487	up	Sus scrofa uncharacterized LOC100626841 (LOC100626841), transcript
A_72_P162201		2,3578	0,0275	up	Pig DNA sequence from clone CH242-145J20 on chromosome X, complete sequence
A_72_P723538		2,3433	0,0439	up	Sus scrofa mRNA, clone:LVR010051G01, expressed in liver
A_72_P116056	HECTD1	2,3426	0,0269	up	Sus scrofa HECT domain containing E3 ubiquitin protein ligase 1 (HECTD1), mRNA
A_72_P414873	DHX36	2,3378	0,0491	up	Sus scrofa DEAH (Asp-Glu-Ala-His) box polypeptide 36 (DHX36), mRNA
A_72_P269579		2,3304	0,0355	up	AJ604906 Sus scrofa skeletal muscle, biceps femoris adult Sus scrofa cDNA clone 10B12Ft7, mRNA sequence
A_72_P006561		2,2975	0,0146	up	1541517 MARC 3PIG Sus scrofa cDNA 3', mRNA sequence
A_72_P304669		2,2909	0,0408	up	AJ959173 KN404_2 Sus scrofa cDNA clone C0007752b12 3', mRNA sequence
A_72_P132286		2,2758	0,0461	up	1116614 MARC 4PIG Sus scrofa cDNA 3', mRNA sequence
A_72_P371598		2,2683	0,0259	up	AJ960829 KN404_2 Sus scrofa cDNA clone C0007755p17 5', mRNA
A_72_P407148		2,2667	0,0346	up	Pig DNA sequence from clone CH242-344D21 on chromosome 4, complete sequence
A_72_P065806		2,2623	0,0127	up	UMC-pcrybst1-012-e10 Blastocyst after cryopreservation pcrybst Sus scrofa cDNA 3', mRNA sequence
A_72_P372433		2,2276	0,0238	up	MI-P-CP0-nvs-e-12-0-UI.s1 MI-P-CP0 Sus scrofa cDNA clone MI-P-CP0-nvs-e-12-0-UI 3', mRNA sequence
A_72_P183056		2,2176	0,0004	up	rret07_b23.y1 ret Sus scrofa cDNA 5', mRNA sequence
A_72_P398673		2,2153	0,0376	up	Pig DNA sequence from clone CH242-253A6 on chromosome X, complete sequence
A_72_P338428		2,2067	0,0399	up	BP155763 full-length enriched swine cDNA library, adult peripheral blood lymphocyte Sus scrofa cDNA clone PBL010002D09 5', mRNA sequence
A_72_P031491		2,1988	0,0048	up	AJ686753 CSEQRAN04 Sus scrofa cDNA clone C0001812_L22, mRNA sequence
A_72_P378973		2,1946	0,0320	up	1527088 MARC 3PIG Sus scrofa cDNA 3', mRNA sequence
A_72_P174606		2,1869	0,0212	up	BP153436 full-length enriched swine cDNA library, adult ovary Sus scrofa cDNA clone OVRM10137G10 5', mRNA sequence
A_72_P386458		2,1865	0,0167	up	CJ029091 full-length enriched swine cDNA library, adult trachea Sus scrofa cDNA clone TCH01F100091 5', mRNA sequence
A_72_P289424		2,1765	0,0169	up	DB803436 full-length enriched swine cDNA library, adult skin Sus scrofa cDNA clone SKNB10057H02 5', mRNA sequence
A_72_P489896	HELLS	2,1745	0,0461	up	Sus scrofa helicase, lymphoid-specific (HELLS), mRNA
A_72_P015821		2,1720	0,0400	up	AJ681382 CSEQRAN04 Sus scrofa cDNA clone C0001795_P06, mRNA
A_72_P014726	ACACA	2,1463	0,0349	up	Sus scrofa clone CH242-27L18 acetyl-coenzyme A carboxylase alpha (ACACA) gene, complete cds
A_72_P286604		2,1394	0,0340	up	Sus scrofa mRNA, clone:OVRM10063H09, expressed in ovary
A_72_P148551		2,1363	0,0161	up	BP170869 full-length enriched swine cDNA library, adult uterus Sus scrofa cDNA clone UTR010004C11 5', mRNA sequence
A_72_P382663		2,1342	0,0074	up	1545338 MARC 3PIG Sus scrofa cDNA 5', mRNA sequence
A_72_P417804		2,1333	0,0388	up	1496210 MARC 3PIG Sus scrofa cDNA 3', mRNA sequence
A_72_P034516		2,1270	0,0176	up	Pig DNA sequence from clone CH242-404E7 on chromosome 7, complete
A_72_P289919		2,1085	0,0023	up	Sus scrofa mRNA, clone:ADR010096H12, expressed in adrenal gland
A_72_P706082	LOC100739480	2,1004	0,0497	up	Sus scrofa zinc finger protein with KRAB and SCAN domains 7-like (LOC100739480), mRNA
A_72_P773793		2,0966	0,0296	up	Sus scrofa BAC clone KNP_1798G2
A_72_P305719	FGF7	2,0775	0,0007	up	Sus scrofa domestica fibroblast growth factor (FGF7) gene, partial cds
A_72_P273784		2,0771	0,0475	up	1535916 MARC 3PIG Sus scrofa cDNA 5', mRNA sequence
A_72_P214197		2,0559	0,0403	up	893457 MARC 4PIG Sus scrofa cDNA 5', mRNA sequence
A_72_P116011		2,0528	0,0471	up	AJ958155 KN404_2 Sus scrofa cDNA clone C0007753e15 5', mRNA sequence
A_72_P390363		2,0230	0,0151	up	BP448011 full-length enriched swine cDNA library, adult liver Sus scrofa cDNA clone LVR010030H11 5', mRNA sequence
A_72_P066786		2,0227	0,0141	up	CJ030293 full-length enriched swine cDNA library, adult testis Sus scrofa cDNA clone TES01A070005 5', mRNA sequence
A_72_P309553		2,0151	0,0453	up	AJ954378 KN404_1 Sus scrofa cDNA clone C0007747o12 5', mRNA sequence

A_72_P317763	MUC4	2,0067	0,0495	up	Sus scrofa mucin 4, cell surface associated (MUC4), mRNA
A_72_P241982		1,9996	0,0158	up	AJ962209 KN404_2 Sus scrofa cDNA clone C0007748e09 5', mRNA
A_72_P239107		1,9977	0,0476	up	Sus scrofa contig sequence, clone KNP_240H2_Contig7
A_72_P128521		1,9868	0,0327	up	Pig DNA sequence from clone CH242-35J8 on chromosome 7, complete
A_72_P177001		1,9856	0,0413	up	Sus scrofa mRNA, clone:LNG010023A05, expressed in lung
A_72_P293279	SMC2	1,9723	0,0085	up	Sus scrofa structural maintenance of chromosomes 2 (SMC2), mRNA
A_72_P285084		1,9682	0,0388	up	1139826 MARC 4PIG Sus scrofa cDNA 3', mRNA sequence
A_72_P125231		1,9662	0,0241	up	370839 MARC 2PIG Sus scrofa cDNA 5', mRNA sequence
A_72_P362148		1,9605	0,0117	up	Pig DNA sequence from clone CH242-195A3 on chromosome 14, complete sequence
A_72_P694466	SLC10A5	1,9588	0,0222	up	Sus scrofa solute carrier family 10, member 5 (SLC10A5), mRNA
A_72_P400423		1,9534	0,0463	up	1571693 MARC 3PIG Sus scrofa cDNA 3', mRNA sequence
A_72_P368353		1,9463	0,0135	up	949737 MARC 4PIG Sus scrofa cDNA 3', mRNA sequence
A_72_P330213		1,9395	0,0414	up	BW972589 full-length enriched swine cDNA library, adult periphral blood mononuclear cell Sus scrofa cDNA clone PBL010072F08 5', mRNA sequence
A_72_P299614		1,9365	0,0448	up	PDUts1077E10 Porcine testis cDNA library I Sus scrofa cDNA clone PDUts1077E10 5' similar to homologue to emb|X64127.1| S.scrofa DNA for SINE sequence SSPRE, mRNA sequence
A_72_P322838		1,9355	0,0267	up	Sus scrofa mRNA, clone:OVRM10106F11, expressed in ovary
A_72_P265212		1,9311	0,0212	up	1572528 MARC 3PIG Sus scrofa cDNA 3', mRNA sequence
A_72_P082996		1,9283	0,0328	up	rret19_h20.y1 ret Sus scrofa cDNA 5', mRNA sequence
A_72_P153351		1,9279	0,0240	up	UNL-P-FN-bo-h-07-0-UNL.s1 UNL-P-FN Sus scrofa cDNA clone UNL-P-FN-
A_72_P155186		1,9261	0,0272	up	CJ010054 full-length enriched swine cDNA library, adult mesenteric lymph
A_72_P047056		1,9249	0,0124	up	AJ958139 KN404_2 Sus scrofa cDNA clone C0007760h04 5', mRNA
A_72_P083691	LOC100519726	1,9105	0,0374	up	Sus scrofa uncharacterized LOC100519726 (LOC100519726), misc_RNA
A_72_P099066		1,9092	0,0362	up	AJ964783 KN404_2 Sus scrofa cDNA clone C0007754a13 5', mRNA sequence
A_72_P182821		1,9052	0,0008	up	rpgl01_f3.y1 pgl Sus scrofa cDNA 5', mRNA sequence
A_72_P167956		1,9017	0,0274	up	43860 MARC 2PIG Sus scrofa cDNA 5', mRNA sequence
A_72_P162191		1,9005	0,0461	up	AJ959837 KN404_2 Sus scrofa cDNA clone C0007749m12 5', mRNA sequence
A_72_P379393		1,8983	0,0385	up	365252 MARC 2PIG Sus scrofa cDNA 5', mRNA sequence
A_72_P022576		1,8973	0,0009	up	953025 MARC 4PIG Sus scrofa cDNA 3', mRNA sequence
A_72_P105391		1,8971	0,0274	up	Sus scrofa mRNA, clone: LVRM10150H08, expressed in liver
A_72_P103866		1,8720	0,0198	up	857804 MARC 3PIG Sus scrofa cDNA 5', mRNA sequence
A_72_P380558		1,8693	0,0183	up	944344 MARC 4PIG Sus scrofa cDNA 5', mRNA sequence
A_72_P423289		1,8641	0,0344	up	129880 MARC 1PIG Sus scrofa cDNA 5', mRNA sequence
A_72_P117971		1,8638	0,0303	up	rjej34b_p3.y1 jej Sus scrofa cDNA 5', mRNA sequence
A_72_P214712		1,8631	0,0448	up	AJ943845 CSEQRAN17 Sus scrofa cDNA clone C0007319n08 5', mRNA sequence
A_72_P216672	MEP1B	1,8587	0,0463	up	Sus scrofa meprin A, beta (MEP1B), mRNA
A_72_P083261		1,8585	0,0268	up	110742 MARC 1PIG Sus scrofa cDNA 5', mRNA sequence
A_72_P403888		1,8521	0,0355	up	AJ959081 KN404_2 Sus scrofa cDNA clone C0007748d07 3', mRNA sequence
A_72_P329343	CCDC99	1,8518	0,0272	up	Sus scrofa spindle apparatus coiled-coil protein 1 (CCDC99), mRNA
A_72_P088431	DIRAS3	1,8438	0,0129	up	Sus scrofa DIRAS family, GTP-binding RAS-like 3 (DIRAS3), mRNA
A_72_P411118		1,8434	0,0214	up	Sus scrofa mRNA, clone: MLTL10048G04, expressed in longissimus muscle
A_72_P280994		1,8409	0,0219	up	Sus scrofa mRNA, clone:OVR010041D02, expressed in ovary
A_72_P027051		1,8405	0,0055	up	1123279 MARC 4PIG Sus scrofa cDNA 3', mRNA sequence
A_72_P219732		1,8401	0,0281	up	Pig DNA sequence from clone CH242-467E16 on chromosome 4, complete sequence
A_72_P316953		1,8381	0,0113	up	Sus scrofa mRNA, clone:TCH010047E01, expressed in trachea
A_72_P035226		1,8317	0,0465	up	Sus scrofa mRNA, clone:OVR010018F09, expressed in ovary
A_72_P301609		1,8298	0,0457	up	1100700 MARC 4PIG Sus scrofa cDNA 3', mRNA sequence
A_72_P267901		1,8272	0,0263	up	Sus scrofa mRNA, clone: DCI010062B03, expressed in dendritic cells (immature)
A_72_P091006		1,8255	0,0272	up	CMD48_G12_88 UMNMPM3 Sus scrofa cDNA clone PPSUBLIB_33G12 5', mRNA sequence
A_72_P309868		1,8135	0,0475	up	Sus scrofa mRNA, clone:SKNB10097G04, expressed in skin
A_72_P294399		1,8083	0,0094	up	Sus scrofa mRNA, clone: OVRT10073F12, expressed in ovary
A_72_P340623	SNED1	1,8065	0,0360	up	PREDICTED: Sus scrofa sushi, nidogen and EGF-like domains 1 (SNED1), transcript variant X1, mRNA
A_72_P090881		1,8007	0,0428	up	Sus scrofa mRNA, clone:LVRM10154G06, expressed in liver
A_72_P056971	LOC100739021	1,7920	0,0005	up	Sus scrofa PC-esterase domain-containing protein 1B-like (LOC100739021), misc_RNA
A_72_P412828		1,7873	0,0322	up	adipo-223 Porcine adipogenesis cDNA library Sus scrofa cDNA, mRNA sequence
A_72_P584121	NOP58	1,7869	0,0264	up	Sus scrofa NOP58 ribonucleoprotein (NOP58), mRNA
A_72_P471611	PPIG	1,7700	0,0155	up	Sus scrofa peptidylprolyl isomerase G (cyclophilin G) (PPIG), mRNA
A_72_P426779		1,7634	0,0324	up	AJ962205 KN404_2 Sus scrofa cDNA clone C0007750j18 5', mRNA sequence
A_72_P145501		1,7628	0,0065	up	1533095 MARC 3PIG Sus scrofa cDNA 5', mRNA sequence
A_72_P305439		1,7585	0,0316	up	1149764 MARC 4PIG Sus scrofa cDNA 3', mRNA sequence
A_72_P366598		1,7569	0,0246	up	CJ025828 full-length enriched swine cDNA library, adult trachea Sus scrofa cDNA clone TCH01H010045 5', mRNA sequence
A_72_P057326		1,7551	0,0319	up	MI-P-CP1-nzc-o-07-0-UI.s1 MI-P-CP1 Sus scrofa cDNA clone MI-P-CP1-nzc-o-07-0-UI 3', mRNA sequence
A_72_P190976		1,7534	0,0373	up	CJ024501 full-length enriched swine cDNA library, adult trachea Sus scrofa cDNA clone TCH01C050028 5', mRNA sequence
A_72_P363403		1,7481	0,0214	up	866514 MARC 4PIG Sus scrofa cDNA 3', mRNA sequence
A_72_P329348		1,7399	0,0490	up	BX917575 Sus Scrofa library (scan) Sus scrofa cDNA clone scan0035.b.12 5prim, mRNA sequence
A_72_P309068		1,7382	0,0435	up	BX914361 Sus Scrofa library (scan) Sus scrofa cDNA clone scan0001.o.15 5prim, mRNA sequence
A_72_P193392		1,7381	0,0375	up	AJ652903 KN277 Sus scrofa cDNA clone C0005182_D17, mRNA sequence
A_72_P301349	PARPBP	1,7342	0,0206	up	Sus scrofa PARP1 binding protein (PARPBP), partial mRNA
A_72_P404513		1,7267	0,0486	up	904399 MARC 4PIG Sus scrofa cDNA 5', mRNA sequence
A_72_P375123		1,7196	0,0230	up	Pig DNA sequence from clone CH242-12K2 on chromosome 2, complete sequence
A_72_P078786		1,7177	0,0297	up	AJ954368 KN404_1 Sus scrofa cDNA clone C0007735l05 5', mRNA sequence
A_72_P374903		1,7166	0,0026	up	DB787348 full-length enriched swine cDNA library, adult adrenal gland Sus scrofa cDNA clone ADR010066B08 5', mRNA sequence

A_72_P101066		1,7152	0,0281	up	rnca28b_i3.y1 nca Sus scrofa cDNA 5', mRNA sequence
A_72_P167521		1,7128	0,0228	up	UMC-pd6end2-008-d07 Endometrium gilt D6 of estrous cycle pd6end Sus scrofa cDNA 3', mRNA sequence
A_72_P366798		1,7127	0,0277	up	AJ687157 CSEQRAN04 Sus scrofa cDNA clone C0001813_N17, mRNA sequence
A_72_P113191		1,7119	0,0410	up	
A_72_P320418		1,7101	0,0352	up	BP149557 full-length enriched swine cDNA library, adult ovary Sus scrofa cDNA clone OVRM10097D09 5', mRNA sequence
A_72_P073376	BCL9	1,7082	0,0495	up	Sus scrofa B-cell CLL/lymphoma 9 (BCL9), mRNA
A_72_P221462		1,7033	0,0078	up	BW983903 full-length enriched swine cDNA library, adult intestine Sus scrofa cDNA clone ITT010089D09 5', mRNA sequence
A_72_P307413		1,6991	0,0467	up	Sus scrofa mRNA, clone:SKNB10050H04, expressed in skin
A_72_P555216	MPHOSPH10	1,6978	0,0313	up	Sus scrofa M-phase phosphoprotein 10 (U3 small nucleolar ribonucleoprotein) (MPHOSPH10), mRNA
A_72_P016536		1,6966	0,0419	up	1143188 MARC 4PIG Sus scrofa cDNA 3', mRNA sequence
A_72_P167716		1,6966	0,0450	up	UMC-pd6end1-008-e09 Endometrium gilt D6 of estrous cycle pd6end Sus scrofa cDNA 3', mRNA sequence
A_72_P187171	SLC37A2	1,6918	0,0458	up	Sus scrofa solute carrier family 37 (glucose-6-phosphate transporter), member 2 (SLC37A2), mRNA
A_72_P250707		1,6870	0,0332	up	Pig DNA sequence from clone CH242-97B16 on chromosome 8, complete sequence
A_72_P069486		1,6853	0,0433	up	Sus scrofa mRNA, clone: KDN010054G03, expressed in kidney
A_72_P441434	TGFA	1,6835	0,0185	up	Sus scrofa transforming growth factor, alpha (TGFA), mRNA
A_72_P246442		1,6826	0,0454	up	Sus scrofa ataxia-telangiectasia mutated protein gene, exons 1 through 64 and complete cds
A_72_P227172		1,6824	0,0376	up	AJ669039 CSEQRAN09 Sus scrofa cDNA clone C0000048_L08, mRNA sequence
A_72_P279074		1,6806	0,0406	up	rhyp01c_m24.y1 hyp Sus scrofa cDNA 5', mRNA sequence
A_72_P395818		1,6779	0,0214	up	1571911 MARC 3PIG Sus scrofa cDNA 3', mRNA sequence
A_72_P389178		1,6754	0,0221	up	1144288 MARC 4PIG Sus scrofa cDNA 3', mRNA sequence
A_72_P436879		1,6744	0,0475	up	AJ959932 KN404_2 Sus scrofa cDNA clone C0007752b10 3', mRNA sequence
A_72_P426064		1,6734	0,0468	up	BX918411 Sus Scrofa library (scan) Sus scrofa cDNA clone scan0038.i.08 5prim, mRNA sequence
A_72_P380223		1,6731	0,0280	up	UMC-pfeto1-013-a06 Fetal ovary pfeto Sus scrofa cDNA 3', mRNA sequence
A_72_P369458		1,6720	0,0446	up	UMC-p6mm2-002-a01 6mm ovarian follicle p6mm Sus scrofa cDNA 3', mRNA sequence
A_72_P443016	CCT6A	1,6703	0,0229	up	Sus scrofa chaperonin containing TCP1, subunit 6A (zeta 1) (CCT6A), mRNA
A_72_P099426		1,6656	0,0186	up	AJ964241 KN404_2 Sus scrofa cDNA clone C0007754e02 5', mRNA sequence
A_72_P521584		1,6628	0,0159	up	EST-AR111H04 Infected Porcine Spleen cDNA library Sus scrofa cDNA clone AR111H04, mRNA sequence
A_72_P186811	SGSM1	1,6613	0,0014	up	Sus scrofa small G protein signaling modulator 1 (SGSM1), mRNA
A_72_P616219	DLD	1,6597	0,0304	up	Sus scrofa dihydrolipoamide dehydrogenase (DLD), mRNA
A_72_P054756		1,6597	0,0252	up	Select seq gb|CV101857.1|
A_72_P321353	CHD2	1,6569	0,0084	up	Sus scrofa chromodomain helicase DNA binding protein 2 (CHD2), mRNA
A_72_P666425	HSPD1	1,6552	0,0032	up	Sus scrofa heat shock 60kDa protein 1 (chaperonin) (HSPD1), mRNA
A_72_P297029	MBOAT1	1,6534	0,0452	up	Sus scrofa membrane bound O-acyltransferase domain containing 1 (MBOAT1), mRNA
A_72_P268639	CEP68	1,6500	0,0463	up	Sus scrofa centrosomal protein 68kDa (CEP68), mRNA
A_72_P231457		1,6493	0,0258	up	CJ024558 full-length enriched swine cDNA library, adult trachea Sus scrofa cDNA clone TCH01A110029 5', mRNA sequence
A_72_P665246		1,6475	0,0067	up	Sus scrofa mRNA, clone:AMP010012A11, expressed in alveolar macrophage
A_72_P442176		1,6472	0,0229	up	Sus scrofa domestica mitochondrial DNA, complete genome
A_72_P379763		1,6469	0,0332	up	937362 MARC 4PIG Sus scrofa cDNA 3', mRNA sequence
A_72_P309623		1,6466	0,0193	up	Sus scrofa mRNA, clone:SKNB10045G09, expressed in skin
A_72_P258692		1,6454	0,0291	up	46042 MARC 2PIG Sus scrofa cDNA 5', mRNA sequence
A_72_P212877		1,6451	0,0372	up	rp11179 Normalized porcine adipose tissues cDNA library Sus scrofa cDNA, mRNA sequence
A_72_P349653	CRYBB3	1,6440	0,0491	up	Sus scrofa crystallin, beta B3 (CRYBB3), mRNA
A_72_P283189		1,6439	0,0367	up	904556 MARC 4PIG Sus scrofa cDNA 5', mRNA sequence
A_72_P760603	ATM	1,6426	0,0268	up	Sus scrofa ataxia telangiectasia mutated (ATM), mRNA
A_72_P310108		1,6415	0,0132	up	886329 MARC 4PIG Sus scrofa cDNA 3', mRNA sequence
A_72_P090681		1,6409	0,0192	up	BW978330 full-length enriched swine cDNA library, adult intestine Sus scrofa cDNA clone ITT010026B11 5', mRNA sequence
A_72_P095956		1,6394	0,0385	up	rgul0118_o6.y1 gul Sus scrofa cDNA 5', mRNA sequence
A_72_P280314	ANKRD50	1,6392	0,0165	up	PREDICTED: Sus scrofa ankyrin repeat domain 50 (ANKRD50), mRNA
A_72_P306079		1,6373	0,0326	up	Sus scrofa mRNA, clone:LVRM10174E09, expressed in liver
A_72_P223093		1,6361	0,0409	up	Pig DNA sequence from clone CH242-194K16 on chromosome X, complete sequence
A_72_P295819		1,6360	0,0381	up	1527065 MARC 3PIG Sus scrofa cDNA 3', mRNA sequence
A_72_P156836		1,6359	0,0471	up	AJ938613 CSEQRAN17 Sus scrofa cDNA clone C0007318g05 3', mRNA sequence
A_72_P273094		1,6344	0,0280	up	AJ941555 CSEQRAN17 Sus scrofa cDNA clone C0007319d12 3', mRNA sequence
A_72_P003726		1,6323	0,0429	up	1115068 MARC 4PIG Sus scrofa cDNA 3', mRNA sequence
A_72_P120251		1,6305	0,0372	up	Sus scrofa clone Clu_28644.scr.msk.p1.Contig1, mRNA sequence
A_72_P104551		1,6254	0,0150	up	FS681524 full-length enriched swine cDNA library, adult longissimus Sus scrofa cDNA clone MLTL10036C10 5', mRNA sequence
A_72_P251257		1,6230	0,0244	up	AR031C10LN05EE02S Porcine Lymph node cDNA library, Cot 5 Sus scrofa cDNA, mRNA sequence
A_72_P052696		1,6181	0,0016	up	Sus scrofa mRNA, clone:MLN010086G12, expressed in mesenteric lymph nodes
A_72_P378758		1,6157	0,0064	up	UMC-pblivv1-003-h11 Blastocycst-(invivo) pblivv Sus scrofa cDNA 3', mRNA sequence
A_72_P248327		1,6147	0,0451	up	CJ007980 full-length enriched swine cDNA library, adult mesenteric lymph node Sus scrofa cDNA clone MLN01C090004 5', mRNA sequence
A_72_P349398	ATL2	1,6143	0,0242	up	Sus scrofa atlastin GTPase 2 (ATL2), mRNA
A_72_P219767		1,6132	0,0477	up	944285 MARC 4PIG Sus scrofa cDNA 5', mRNA sequence
A_72_P147736	LEO1	1,6131	0,0299	up	Sus scrofa Leo1, Paf1/RNA polymerase II complex component, homolog (S. cerevisiae) (LEO1), mRNA
A_72_P509075	LOC100525988	1,6115	0,0075	up	Sus scrofa aspartyl/asparaginyl beta-hydroxylase-like (LOC100525988), mRNA
A_72_P347933		1,6090	0,0373	up	Sus scrofa mRNA, clone:TCH010062H06, expressed in trachea
A_72_P013686	TRPV2	1,6047	0,0077	up	Sus scrofa transient receptor potential cation channel, subfamily V, member 2 (TRPV2), mRNA
A_72_P186731		1,6042	0,0003	up	Select seq gb|EW340903.2|

A_72_P205117		1,6028	0,0273	up	Sus scrofa mRNA, clone:OVRM10074F04, expressed in ovary.
A_72_P190801	CCDC91	1,6019	0,0035	up	Sus scrofa coiled-coil domain containing 91 (CCDC91), mRNA
A_72_P006091	PLA2G4A	1,6015	0,0172	up	Sus scrofa phospholipase A2, group IVA (cytosolic, calcium-dependent) (PLA2G4A), mRNA
A_72_P269194		1,6006	0,0014	up	rplac_11608.y1 pla Sus scrofa cDNA 5', mRNA sequence
A_72_P189856	NEO1	1,6005	0,0364	up	Sus scrofa neogenin 1 (NEO1), partial mRNA
A_72_P732228		1,5994	0,0171	up	Sus scrofa mitochondrion, complete genome
A_72_P308473		1,5981	0,0249	up	AJ955916 KN404_2 Sus scrofa cDNA clone C0007760m04 3', mRNA sequence
A_72_P118151		1,5977	0,0211	up	244810 MARC 2PIG Sus scrofa cDNA 5', mRNA sequence
A_72_P608427		1,5952	0,0059	up	Sus scrofa mRNA, clone:AMP010012A11, expressed in alveolar macrophage
A_72_P348868		1,5906	0,0221	up	UNL-P-FN-bo-f-05-0-UNL.s1 UNL-P-FN Sus scrofa cDNA clone UNL-P-FN-bo-f-05-0-UNL 3', mRNA sequence
A_72_P065641		1,5888	0,0027	up	Pig DNA sequence from clone CH242-28F9 on chromosome 7, complete sequence
A_72_P519181	LOC100738992	1,5859	0,0408	up	Sus scrofa zinc finger transcription factor Trps1-like (LOC100738992), mRNA
A_72_P309683		1,5850	0,0345	up	BP146922 full-length enriched swine cDNA library, adult ovary Sus scrofa cDNA clone OVRM10081B08 5', mRNA sequence
A_72_P611693		1,5846	0,0090	up	Sus scrofa mRNA, clone:AMP010012A11, expressed in alveolar macrophage
A_72_P261027		1,5836	0,0229	up	Sus scrofa mRNA, clone:AMP010012A11, expressed in alveolar macrophage
A_72_P719508		1,5817	0,0226	up	Sus scrofa mitochondrion, complete genome
A_72_P361018		1,5811	0,0040	up	998248 MARC 4PIG Sus scrofa cDNA 3', mRNA sequence
A_72_P384273		1,5805	0,0190	up	AJ667988 CSEQRAN09 Sus scrofa cDNA clone C0000044_C01, mRNA sequence
A_72_P088506	RYR1	1,5804	0,0095	up	Sus scrofa ryanodine receptor 1 (skeletal) (RYR1), mRNA
A_72_P023781	LOC100524682	1,5790	0,0300	up	Sus scrofa leukocyte receptor cluster member 8 homolog (LOC100524682), mRNA
A_72_P724458		1,5733	0,0225	up	Sus scrofa mitochondrion, complete genome
A_72_P431379		1,5726	0,0346	up	1513985 MARC 3PIG Sus scrofa cDNA 3', mRNA sequence
A_72_P332538		1,5712	0,0186	up	Sus scrofa mRNA, clone:ITT010037H11, expressed in small intestine
A_72_P058141		1,5696	0,0112	up	BX926768 Sus Scrofa library (scan) Sus scrofa cDNA clone scan0015.a.16 5prim, mRNA sequence
A_72_P230897		1,5665	0,0297	up	BP169889 full-length enriched swine cDNA library, adult uterus Sus scrofa cDNA clone UTR010005B01 5', mRNA sequence
A_72_P080318	ACSL3	1,5661	0,0196	up	Sus scrofa acyl-CoA synthetase long-chain family member 3 (ACSL3), mRNA
A_72_P155906		1,5653	0,0094	up	875820 MARC 4PIG Sus scrofa cDNA 3', mRNA sequence
A_72_P259962		1,5579	0,0004	up	885854 MARC 4PIG Sus scrofa cDNA 3', mRNA sequence
A_72_P134961		1,5551	0,0352	up	Pig DNA sequence from clone CH242-225C1 on chromosome X, complete sequence
A_72_P231247		1,5551	0,0134	up	Sus scrofa mRNA, clone:BKFL10107G11, expressed in back fat
A_72_P289774	SERPINB8	1,5537	0,0198	up	Sus scrofa serpin peptidase inhibitor, clade B (ovalbumin), member 8 (SERPINB8), mRNA
A_72_P489297	LOC100737162	1,5520	0,0328	up	Sus scrofa uncharacterized LOC100737162 (LOC100737162), mRNA
A_72_P257367	CUL5	1,5504	0,0157	up	Sus scrofa cullin 5 (CUL5), mRNA
A_72_P322803	MCMBP	1,5491	0,0342	up	Sus scrofa minichromosome maintenance complex binding protein (MCMBP), mRNA
A_72_P741589		1,5466	0,0259	up	Sus scrofa mitochondrion, complete genome
A_72_P217282	TOPAZ1	1,5452	0,0489	up	PREDICTED: Sus scrofa testis and ovary specific PAZ domain containing 1 (TOPAZ1), partial mRNA
A_72_P178331		1,5446	0,0211	up	AJ961763 KN404_2 Sus scrofa cDNA clone C0007750d02 5', mRNA sequence
A_72_P390863		1,5431	0,0217	up	539412 MARC 2PIG Sus scrofa cDNA 5', mRNA sequence
A_72_P393898		1,5416	0,0306	up	Pig DNA sequence from clone CH242-235H24 on chromosome 14, complete sequence
A_72_P350353		1,5397	0,0474	up	835587 MARC 3PIG Sus scrofa cDNA 3', mRNA sequence
A_72_P589919		1,5387	0,0372	up	Sus scrofa mRNA, clone:AMP010075C06, expressed in alveolar macrophage
A_72_P108091		1,5379	0,0476	up	Sus scrofa mRNA, clone:MLN010100H02, expressed in mesenteric lymph nodes
A_72_P364728	DDHD2	1,5365	0,0018	up	Sus scrofa DDHD domain containing 2 (DDHD2), transcript variant X1, mRNA
A_72_P226722	SEC24A	1,5362	0,0382	up	Sus scrofa SEC24 family, member A (S. cerevisiae) (SEC24A), mRNA
A_72_P606508	GOLGA4	1,5355	0,0387	up	Sus scrofa golgin A4 (GOLGA4), mRNA
A_72_P322948		1,5353	0,0153	up	Sus scrofa mRNA, clone:OVRM10092G02, expressed in ovary
A_72_P211412		1,5352	0,0240	up	AJ956694 KN404_2 Sus scrofa cDNA clone C0007748n08 5', mRNA sequence
A_72_P003031		1,5320	0,0002	up	1144601 MARC 4PIG Sus scrofa cDNA 3', mRNA sequence
A_72_P049891		1,5320	0,0250	up	938707 MARC 4PIG Sus scrofa cDNA 5', mRNA sequence
A_72_P631310	FAM214A	1,5318	0,0251	up	Sus scrofa family with sequence similarity 214, member A (FAM214A), mRNA
A_72_P049111		1,5306	0,0239	up	Sus scrofa mRNA, clone:SPL010048D03, expressed in spleen
A_72_P208272	LOC414428	1,5305	0,0444	up	Sus scrofa muskelin 1 (LOC414428), mRNA
A_72_P249247		1,5286	0,0034	up	rpgl27_f8.y1 pgl Sus scrofa cDNA 5', mRNA sequence
A_72_P403523		1,5280	0,0448	up	AJ960020 KN404_2 Sus scrofa cDNA clone C0007758k22 3', mRNA sequence
A_72_P211902		1,5232	0,0431	up	MI-P-AY1-nrs-c-10-0-UI.s1 MI-P-AY1 Sus scrofa cDNA clone MI-P-AY1-nrs-c-10-0-UI 3', mRNA sequence
A_72_P489945	ZNF518A	1,5222	0,0385	up	Sus scrofa zinc finger protein 518A (ZNF518A), mRNA
A_72_P227292		1,5221	0,0254	up	Sus scrofa mRNA, clone: AMP010074D07, expressed in alveolar macrophage
A_72_P063271	LOC106510391	1,5206	0,0275	up	PREDICTED: Sus scrofa uncharacterized LOC106510391 (LOC106510391), ncRNA
A_72_P055816		1,5200	0,0146	up	Sus scrofa mRNA, clone:LVRM10114E03, expressed in liver
A_72_P148516		1,5178	0,0444	up	BP155797 full-length enriched swine cDNA library, adult peripheral blood lymphocyte Sus scrofa cDNA clone PBL010032D11 5', mRNA sequence
A_72_P113531		1,5175	0,0397	up	Sus scrofa mRNA, clone: MLTL10083F11, expressed in longissimus muscle
A_72_P279964		1,5164	0,0443	up	rgul0117_f9.y1 gul Sus scrofa cDNA 5', mRNA sequence
A_72_P404188		1,5163	0,0222	up	Pig DNA sequence from clone CH242-171J7 on chromosome X, complete sequence
A_72_P189346	LSMEM1	1,5156	0,0140	up	Sus scrofa leucine-rich single-pass membrane protein 1 (LSMEM1), mRNA
A_72_P243822	SNAPC5	1,5153	0,0412	up	Sus scrofa small nuclear RNA activating complex, polypeptide 5, 19kDa (SNAPC5), mRNA
A_72_P130541		1,5122	0,0167	up	Sus scrofa mRNA, clone:OVRM10091G02, expressed in ovary
A_72_P109886		1,5114	0,0416	up	AJ658972 KN277 Sus scrofa cDNA clone C0005215_F03, mRNA sequence

A_72_P046946		1,5078	0,0238	up	AJ957767 KN404_2 Sus scrofa cDNA clone C0007756j13 5', mRNA sequence
A_72_P060276		1,5070	0,0223	up	rhyp30c_a7.y1 hyp Sus scrofa cDNA 5', mRNA sequence
A_72_P429234	SCN2B	1,5046	0,0319	up	PREDICTED: Sus scrofa sodium channel, voltage gated, type II beta subunit (SCN2B), transcript variant X1, mRNA
A_72_P121166		1,5013	0,0440	up	Sus scrofa mRNA, clone:THY010098E11, expressed in thymus
A_72_P383818		1,4988	0,0035	up	1570255 MARC 3PIG Sus scrofa cDNA 3', mRNA sequence
A_72_P085681		1,4978	0,0415	up	Sus scrofa mRNA, clone:AMP010019C01, expressed in alveolar macrophage
A_72_P239382		1,4966	0,0434	up	Sus scrofa mRNA, clone:PTG010089B03, expressed in pituitary gland
A_72_P015111		1,4963	0,0479	up	916813 MARC 4PIG Sus scrofa cDNA 3', mRNA sequence
A_72_P099856		1,4950	0,0481	up	AJ963321 KN404_2 Sus scrofa cDNA clone C0007758j07 5', mRNA sequence
A_72_P704201	UGGT1	1,4945	0,0229	up	Sus scrofa UDP-glucose glycoprotein glucosyltransferase 1 (UGGT1), mRNA
A_72_P373148		1,4943	0,0402	up	1539590 MARC 3PIG Sus scrofa cDNA 3', mRNA sequence
A_72_P301044		1,4898	0,0269	up	1106092 MARC 4PIG Sus scrofa cDNA 5', mRNA sequence
A_72_P387143	SYNCRIP	1,4881	0,0095	up	Sus scrofa synaptotagmin binding, cytoplasmic RNA interacting protein (SYNCRIP), mRNA
A_72_P194712		1,4850	0,0318	up	AJ958745 KN404_2 Sus scrofa cDNA clone C0007748g13 3', mRNA sequence
A_72_P260222		1,4848	0,0429	up	rtra16_e21r1.y1 tra Sus scrofa cDNA 5', mRNA sequence
A_72_P386738		1,4840	0,0308	up	AJ958173 KN404_2 Sus scrofa cDNA clone C0007759e20 5', mRNA sequence
A_72_P611673	TTC14	1,4824	0,0224	up	Sus scrofa tetratricopeptide repeat domain 14 (TTC14), mRNA
A_72_P704046	TMEM177	1,4816	0,0275	up	Sus scrofa transmembrane protein 177 (TMEM177), mRNA
A_72_P170426		1,4799	0,0123	up	AJ957369 KN404_2 Sus scrofa cDNA clone C0007759e20 3', mRNA sequence
A_72_P328018		1,4776	0,0357	up	rspl03_g22.y1 spl Sus scrofa cDNA 5', mRNA sequence
A_72_P425174		1,4761	0,0383	up	Sus scrofa mRNA, clone:AMP010075C06, expressed in alveolar macrophage
A_72_P363858		1,4746	0,0368	up	Sus scrofa mRNA, clone:OVRM10151D05, expressed in ovary
A_72_P081126		1,4741	0,0379	up	Sus scrofa mRNA, clone: DCI010065A07, expressed in dendritic cells (immature)
A_72_P145676	LOC100620089	1,4730	0,0477	up	Sus scrofa zinc finger protein 732-like (LOC100620089), mRNA
A_72_P167056		1,4728	0,0118	up	Pig DNA sequence from clone CH242-289H21 on chromosome 4, complete sequence
A_72_P707992	CSN2	1,4719	0,0169	up	Sus scrofa casein beta (CSN2), mRNA
A_72_P009421		1,4678	0,0027	up	AJ956544 KN404_2 Sus scrofa cDNA clone C0007749l16 5', mRNA sequence
A_72_P400943		1,4677	0,0422	up	ESTPBL133a differential display RT-PCR clones Sus scrofa cDNA clone BL133a, mRNA sequence
A_72_P288389		1,4672	0,0429	up	AJ655810 KN277 Sus scrofa cDNA clone C0005191_M19, mRNA sequence
A_72_P172196		1,4668	0,0408	up	1122050 MARC 4PIG Sus scrofa cDNA 3', mRNA sequence
A_72_P397858		1,4668	0,0271	up	137451 MARC 1PIG Sus scrofa cDNA 5', mRNA sequence
A_72_P726683	MT-ND2	1,4663	0,0238	up	Sus scrofa domesticus breed Mangalica mitochondrion, complete genome
A_72_P340923	SV2B	1,4623	0,0416	up	Sus scrofa synaptic vesicle glycoprotein 2B (SV2B), mRNA
A_72_P240787		1,4557	0,0365	up	860845 MARC 4PIG Sus scrofa cDNA 5', mRNA sequence
A_72_P369213		1,4557	0,0163	up	AJ664028 CSEQRAN09 Sus scrofa cDNA clone C0000028_D11, mRNA sequence
A_72_P321488		1,4548	0,0362	up	807179 MARC 3PIG Sus scrofa cDNA 5', mRNA sequence
A_72_P257407	ZBTB11	1,4545	0,0356	up	Sus scrofa zinc finger and BTB domain containing 11 (ZBTB11), mRNA
A_72_P154396		1,4539	0,0308	up	AJ938463 CSEQRAN17 Sus scrofa cDNA clone C0007307i22 3', mRNA sequence
A_72_P380793		1,4535	0,0061	up	917123 MARC 4PIG Sus scrofa cDNA 3', mRNA sequence
A_72_P094966		1,4535	0,0431	up	AJ947512 KN404_1 Sus scrofa cDNA clone C0007746l03 5', mRNA sequence
A_72_P473686	DPY19L4	1,4526	0,0481	up	Sus scrofa dpy-19-like 4 (C. elegans) (DPY19L4), mRNA
A_72_P257517	SAMD8	1,4517	0,0363	up	Sus scrofa sterile alpha motif domain containing 8 (SAMD8), mRNA
A_72_P326458	ENPP1	1,4517	0,0332	up	Sus scrofa ectonucleotide pyrophosphatase/phosphodiesterase 1 (ENPP1), mRNA
A_72_P731193	GPM6B	1,4507	0,0084	up	Sus scrofa glycoprotein M6B (GPM6B), mRNA
A_72_P313248		1,4505	0,0440	up	AJ659592 KN277 Sus scrofa cDNA clone C0005217_H21, mRNA sequence
A_72_P066526	KIF21A	1,4501	0,0339	up	Sus scrofa kinesin family member 21A (KIF21A), mRNA
A_72_P396763		1,4470	0,0003	up	1139004 MARC 4PIG Sus scrofa cDNA 3', mRNA sequence
A_72_P213142	RBM12	1,4464	0,0465	up	PREDICTED: Sus scrofa RNA binding motif protein 12 (RBM12), transcript variant X1, mRNA
A_72_P315593	NAA15	1,4447	0,0295	up	Sus scrofa N(alpha)-acetyltransferase 15, NatA auxiliary subunit (NAA15), mRNA
A_72_P081286		1,4432	0,0158	up	Sus scrofa mRNA, clone: DCI010005C02, expressed in dendritic cells (immature)
A_72_P285104		1,4422	0,0034	up	Sus scrofa BAC clone CHORI242_375P3_Contig114
A_72_P025536		1,4420	0,0180	up	AJ956685 KN404_2 Sus scrofa cDNA clone C0007748g21 5', mRNA sequence
A_72_P006641		1,4419	0,0365	up	Sus scrofa serum paraoxonase/arylesterase 2-like (LOC100620687), mRNA
A_72_P382353		1,4411	0,0389	up	853660 MARC 3PIG Sus scrofa cDNA 3', mRNA sequence
A_72_P310633		1,4377	0,0226	up	891871 MARC 4PIG Sus scrofa cDNA 3', mRNA sequence
A_72_P570469		1,4360	0,0466	up	BX925554 Sus Scrofa library (scan) Sus scrofa cDNA clone scan0021.k.05 5prim, mRNA sequence
A_72_P416923	ALG11	1,4339	0,0470	up	Sus scrofa ALG11, alpha-1,2-mannosyltransferase (ALG11), mRNA
A_72_P113766		1,4335	0,0151	up	1538809 MARC 3PIG Sus scrofa cDNA 3', mRNA sequence
A_72_P442187	ATP8	1,4322	0,0376	up	Sus scrofa domesticus breed Mangalica mitochondrion, complete genome
A_72_P216937		1,4307	0,0055	up	1114907 MARC 4PIG Sus scrofa cDNA 3', mRNA sequence
A_72_P646798	FKBP3	1,4302	0,0392	up	Sus scrofa FK506 binding protein 3, 25kDa (FKBP3), mRNA
A_72_P015186		1,4274	0,0350	up	1543733 MARC 3PIG Sus scrofa cDNA 5', mRNA sequence
A_72_P429784	UBE3A	1,4274	0,0220	up	Sus scrofa ubiquitin protein ligase E3A (UBE3A), mRNA
A_72_P279984		1,4264	0,0468	up	HX221364 full-length enriched swine cDNA library, adult colon Sus scrofa cDNA clone CLNT10129C04, mRNA sequence
A_72_P707113	GFRA2	1,4244	0,0036	up	Sus scrofa GDNF family receptor alpha 2 (GFRA2), mRNA
A_72_P434289		1,4240	0,0392	up	1142416 MARC 4PIG Sus scrofa cDNA 3', mRNA sequence [DN128347]
A_72_P433354		1,4172	0,0458	up	Pig DNA sequence from clone CH242-424M7 on chromosome X, complete sequence
A_72_P544737	KIAA0020	1,4171	0,0317	up	Sus scrofa KIAA0020 ortholog (KIAA0020), mRNA
A_72_P507958	FOCAD	1,4130	0,0063	up	Sus scrofa focadhesin (FOCAD), mRNA
A_72_P102906		1,4114	0,0373	up	140981 MARC 1PIG Sus scrofa cDNA 5', mRNA sequence

A_72_P636667	CCDC152	1,4108	0,0418	up	Sus scrofa coiled-coil domain containing 152 (CCDC152), mRNA
A_72_P402578		1,4106	0,0309	up	EST-AO234H12 Spleen from PRRSV-infected pig Sus scrofa cDNA clone AO234H12, mRNA sequence
A_72_P362958	VPS26A	1,4097	0,0442	up	Sus scrofa vacuolar protein sorting 26 homolog A (S. pombe) (VPS26A), mRNA
A_72_P033936	GNL3	1,4079	0,0189	up	Sus scrofa guanine nucleotide binding protein-like 3 (nucleolar) (GNL3), transcript variant X4, mRNA
A_72_P249337	ABCA3	1,4077	0,0054	up	Sus scrofa ATP-binding cassette sub-family A member 3-like (LOC102166493), mRNA
A_72_P132686	CUX2	1,4073	0,0475	up	Sus scrofa cut-like homeobox 2 (CUX2), mRNA
A_72_P373553		1,4069	0,0287	up	821882 MARC 3PIG Sus scrofa cDNA 3', mRNA sequence
A_72_P177476	RABGGTB	1,4065	0,0260	up	Sus scrofa Rab geranylgeranyltransferase, beta subunit (RABGGTB), mRNA
A_72_P149441	LOC100517984	1,4036	0,0430	up	Sus scrofa UPF0415 protein C7orf25 homolog (LOC100517984), mRNA
A_72_P199317	UCHL5	1,4012	0,0484	up	Sus scrofa ubiquitin carboxyl-terminal hydrolase L5 (UCHL5), mRNA
A_72_P588316		1,3984	0,0322	up	Sus scrofa mRNA, clone:AMP010075C06, expressed in alveolar macrophage
A_72_P356563	LOC102162987	1,3981	0,0104	up	Sus scrofa multidrug resistance-associated protein 6-like (LOC102162987), mRNA
A_72_P264577		1,3974	0,0262	up	1119645 MARC 4PIG Sus scrofa cDNA 3', mRNA sequence
A_72_P240007		1,3974	0,0350	up	866550 MARC 4PIG Sus scrofa cDNA 3', mRNA sequence
A_72_P278029		1,3960	0,0064	up	rcst33_i6.y1 cst Sus scrofa cDNA 5', mRNA sequence
A_72_P253967		1,3949	0,0181	up	BP166821 full-length enriched swine cDNA library, adult thymus Sus scrofa cDNA clone THY010001G01 5', mRNA sequence
A_72_P373903		1,3912	0,0244	up	Sus scrofa mRNA, clone:LVRM10134E01, expressed in liver
A_72_P314838		1,3900	0,0253	up	Sus scrofa mRNA, clone:ITT010065D10, expressed in small intestine
A_72_P365448	KPNA5	1,3892	0,0164	up	PREDICTED: Sus scrofa karyopherin alpha 5 (importin alpha 6) (KPNA5), transcript variant X1, mRNA
A_72_P443162	SMARCA1	1,3887	0,0221	up	Sus scrofa SWI/SNF related, matrix associated, actin dependent regulator of chromatin, subfamily a, member 1 (SMARCA1), mRNA
A_72_P436809		1,3879	0,0479	up	
A_72_P298164	NUDT12	1,3873	0,0426	up	Sus scrofa nudix (nucleoside diphosphate linked moiety X)-type motif 12 (NUDT12), mRNA
A_72_P658461		1,3871	0,0367	up	Sus scrofa mRNA, clone:AMP010025B11, expressed in alveolar macrophages
A_72_P472373	RQCD1	1,3863	0,0085	up	Sus scrofa RCD1 required for cell differentiation1 homolog (S. pombe) (RQCD1), mRNA.
A_72_P305429	DGKH	1,3862	0,0467	up	PREDICTED: Sus scrofa diacylglycerol kinase, eta (DGKH), transcript variant X1, mRNA
A_72_P409258		1,3850	0,0488	up	941956 MARC 4PIG Sus scrofa cDNA 3', mRNA sequence
A_72_P163136	UBR1	1,3841	0,0187	up	PREDICTED: Sus scrofa ubiquitin protein ligase E3 component n-recognin 1 (UBR1), mRNA
A_72_P083301		1,3841	0,0277	up	1499884 MARC 3PIG Sus scrofa cDNA 3', mRNA sequence
A_72_P084146		1,3834	0,0452	up	938891 MARC 4PIG Sus scrofa cDNA 5', mRNA sequence
A_72_P239357	NOP56	1,3824	0,0025	up	Sus scrofa NOP56 ribonucleoprotein homolog (yeast) (NOP56), mRNA
A_72_P179031		1,3793	0,0401	up	AJ957635 KN404_2 Sus scrofa cDNA clone C0007750p03 3', mRNA sequence
A_72_P283234	KIAA1429	1,3769	0,0261	up	PREDICTED: Sus scrofa KIAA1429 ortholog (KIAA1429), mRNA
A_72_P216802		1,3756	0,0394	up	949746 MARC 4PIG Sus scrofa cDNA 3', mRNA sequence
A_72_P340503		1,3738	0,0203	up	FS646627 full-length enriched swine cDNA library, adult brain (frontal lobe) Sus scrofa cDNA clone BFLT10084C05 5', mRNA sequence
A_72_P348018	LOC100626209	1,3723	0,0072	up	Sus scrofa uncharacterized LOC100626209 (LOC100626209), mRNA
A_72_P361613		1,3720	0,0127	up	998224 MARC 4PIG Sus scrofa cDNA 3', mRNA sequence
A_72_P394048	TRMT11	1,3703	0,0176	up	Sus scrofa tRNA methyltransferase 11 homolog (S. cerevisiae) (TRMT11), mRNA
A_72_P418334		1,3690	0,0390	up	AJ658526 KN277 Sus scrofa cDNA clone C0005212_K17, mRNA sequence
A_72_P109306	ZNF667	1,3689	0,0228	up	Sus scrofa zinc finger protein 667 (ZNF667), mRNA
A_72_P230722		1,3684	0,0271	up	Sus scrofa mRNA, clone:TCH010100C01, expressed in trachea
A_72_P003201		1,3664	0,0405	up	Sus scrofa mRNA, clone: MLN010096B09, expressed in mesenteric lymph node
A_72_P087836		1,3654	0,0190	up	999930 MARC 4PIG Sus scrofa cDNA 3', mRNA sequence
A_72_P121356		1,3653	0,0487	up	1118251 MARC 4PIG Sus scrofa cDNA 3', mRNA sequence
A_72_P257027	SEPP1	1,3651	0,0229	up	Sus scrofa selenoprotein P, plasma, 1 (SEPP1), mRNA
A_72_P351699		1,3646	0,0359	up	Sus scrofa mRNA, clone:LVRM10080C04, expressed in liver
A_72_P489239	RTKN2	1,3634	0,0311	up	Sus scrofa rhotekin 2 (RTKN2), mRNA
A_72_P150986		1,3633	0,0400	up	355673 MARC 1PIG Sus scrofa cDNA 5', mRNA sequence
A_72_P254887		1,3631	0,0291	up	891989 MARC 4PIG Sus scrofa cDNA 3', mRNA sequence
A_72_P098371	DCLRE1A	1,3613	0,0062	up	Sus scrofa DNA cross-link repair 1A (DCLRE1A), mRNA
A_72_P106626	SLC35A5	1,3609	0,0321	up	Sus scrofa solute carrier family 35, member A5 (SLC35A5), mRNA
A_72_P258182		1,3603	0,0321	up	PBL_19_G08 Porcine Brain Library Sus scrofa cDNA clone PBL 5', mRNA sequence
A_72_P188496		1,3577	0,0460	up	1116417 MARC 4PIG Sus scrofa cDNA 3', mRNA sequence
A_72_P342313		1,3577	0,0398	up	1118906 MARC 4PIG Sus scrofa cDNA 3', mRNA sequence
A_72_P396023		1,3573	0,0483	up	1543431 MARC 3PIG Sus scrofa cDNA 5', mRNA sequence
A_72_P179526	KDM1B	1,3548	0,0327	up	Sus scrofa lysine (K)-specific demethylase 1B (KDM1B), mRNA
A_72_P034166		1,3531	0,0020	up	AJ953749 KN404_1 Sus scrofa cDNA clone C0007740o02 5', mRNA sequence
A_72_P663732	RABGGTB	1,3527	0,0130	up	Sus scrofa rab geranylgeranyltransferase, beta subunit (RABGGTB) mRNA, complete cds
A_72_P051791	TMEM68	1,3514	0,0280	up	Sus scrofa transmembrane protein 68 (TMEM68), mRNA
A_72_P180541	RPS6KB1	1,3494	0,0429	up	Sus scrofa ribosomal protein S6 kinase, 70kDa, polypeptide 1 (RPS6KB1), mRNA
A_72_P403818		1,3488	0,0285	up	999216 MARC 4PIG Sus scrofa cDNA 3', mRNA sequence
A_72_P355333		1,3486	0,0375	up	1512280 MARC 3PIG Sus scrofa cDNA 3', mRNA sequence
A_72_P162911		1,3476	0,0308	up	rspc030_b8.y1 spc Sus scrofa cDNA 5', mRNA sequence
A_72_P250332	LOC100739232	1,3452	0,0286	up	PREDICTED: Sus scrofa transmembrane protein 236 (LOC100739232), mRNA
A_72_P494665	TMEM8B	1,3443	0,0053	up	Sus scrofa transmembrane protein 8B (TMEM8B), mRNA
A_72_P173881		1,3437	0,0048	up	UMC-pd10en3-021-e05 Endometrium gilt D10 of estrous cycle pd10en Sus scrofa cDNA 3', mRNA sequence
A_72_P004571		1,3432	0,0363	up	Sus scrofa mRNA, clone:TCH010070H12, expressed in trachea
A_72_P235667		1,3429	0,0337	up	CJ015393 full-length enriched swine cDNA library, adult mesenteric lymph node Sus scrofa cDNA clone MLN01H010096 5', mRNA sequence
A_72_P371093	FAM81B	1,3419	0,0437	up	Sus scrofa family with sequence similarity 81, member B (FAM81B), mRNA
A_72_P564269	RABGGTB	1,3417	0,0452	up	Sus scrofa Rab geranylgeranyltransferase, beta subunit (RABGGTB), mRNA
A_72_P371913	MRS2	1,3412	0,0041	up	Sus scrofa MRS2 magnesium transporter (MRS2), mRNA
A_72_P034096		1,3402	0,0215	up	1512978 MARC 3PIG Sus scrofa cDNA 3', mRNA sequence

A_72_P722813	SOCS4	1,3380	0,0274	up	Sus scrofa suppressor of cytokine signaling 4 (SOCS4), mRNA
A_72_P007776		1,3376	0,0316	up	1512978 MARC 3PIG Sus scrofa cDNA 3', mRNA sequence
A_72_P491350		1,3361	0,0312	up	PREDICTED: Sus scrofa HBS1-like translational GTPase (HBS1L), transcript variant X1, mRNA
A_72_P103976		1,3352	0,0349	up	1107432 MARC 4PIG Sus scrofa cDNA 5', mRNA sequence
A_72_P296474		1,3345	0,0336	up	CJ029106 full-length enriched swine cDNA library, adult trachea Sus scrofa cDNA clone TCH01H040091 5', mRNA sequence
A_72_P560239	SEPP1	1,3338	0,0232	up	Sus scrofa selenoprotein P, plasma, 1 (SEPP1), mRNA
A_72_P076386		1,3333	0,0290	up	MI-P-E6-ahk-f-05-1-UM.s1 MI-P-E6 Sus scrofa cDNA clone MI-P-E6-ahk-f-05-1-UM 3', mRNA sequence
A_72_P277474	HOOK1	1,3320	0,0269	up	Sus scrofa hook microtubule-tethering protein 1 (HOOK1), mRNA
A_72_P675941	COPS5	1,3316	0,0474	up	Sus scrofa COP9 constitutive photomorphogenic homolog subunit 5 (Arabidopsis) (COPS5), mRNA.
A_72_P198062		1,3314	0,0061	up	1512141 MARC 3PIG Sus scrofa cDNA 3', mRNA sequence
A_72_P156176		1,3307	0,0445	up	BP171071 full-length enriched swine cDNA library, adult uterus Sus scrofa cDNA clone UTR010020D03 5', mRNA sequence
A_72_P562789	LOC733678	1,3305	0,0382	up	Sus scrofa ATP synthase H+-transporting mitochondrial F1 complex O subunit (LOC733678), mRNA
A_72_P606738	BMPR1A	1,3300	0,0369	up	Sus scrofa bone morphogenetic protein receptor, type IA (BMPR1A), mRNA
A_72_P358358	ATP9A	1,3294	0,0453	up	Sus scrofa ATPase, class II, type 9A (ATP9A), mRNA
A_72_P198057	ATP5O	1,3282	0,0240	up	Sus scrofa mitochondrial ATP5O (ATP5O) mRNA, complete cds; nuclear gene for mitochondrial product
A_72_P471711	OLA1	1,3269	0,0247	up	Sus scrofa Obg-like ATPase 1 (OLA1), mRNA
A_72_P051831	KPNA5	1,3268	0,0252	up	PREDICTED: Sus scrofa karyopherin alpha 5 (importin alpha 6) (KPNA5), transcript variant X1, mRNA
A_72_P369743		1,3265	0,0119	up	Pig DNA sequence from clone CH242-127N16 on chromosome X, complete sequence
A_72_P043451		1,3262	0,0262	up	MI-P-E6-ace-a-02-1-UM.s1 MI-P-E6 Sus scrofa cDNA clone MI-P-E6-ace-a-02-1-UM 3', mRNA sequence
A_72_P118076		1,3246	0,0445	up	Sus scrofa mRNA, clone:TCH010015G11, expressed in trachea
A_72_P307728		1,3243	0,0428	up	1148873 MARC 4PIG Sus scrofa cDNA 3', mRNA sequence
A_72_P405318		1,3242	0,0412	up	CJ038202 full-length enriched swine cDNA library, adult uterus Sus scrofa cDNA clone UTR01C070089 5', mRNA sequence
A_72_P099561		1,3231	0,0133	up	AJ964528 KN404_2 Sus scrofa cDNA clone C0007755i23 5', mRNA sequence
A_72_P163921		1,3226	0,0214	up	FS663198 full-length enriched swine cDNA library, adult colon Sus scrofa cDNA clone CLNT10085G03 5', mRNA sequence
A_72_P383443	LOC100517362	1,3224	0,0375	up	Sus scrofa protein regulator of cytokinesis 1-like (LOC100517362), mRNA
A_72_P027181	PRC1	1,3223	0,0302	up	PREDICTED: Sus scrofa protein regulator of cytokinesis 1 (PRC1), mRNA
A_72_P288544		1,3219	0,0455	up	1519450 MARC 3PIG Sus scrofa cDNA 3', mRNA sequence
A_72_P288734	CCDC62	1,3205	0,0145	up	Sus scrofa coiled-coil domain containing 62 (CCDC62), mRNA
A_72_P294684	TMEM161B	1,3195	0,0341	up	Sus scrofa transmembrane protein 161B (TMEM161B), mRNA
A_72_P445078	CHUK	1,3190	0,0475	up	Sus scrofa conserved helix-loop-helix ubiquitous kinase (CHUK), mRNA
A_72_P421779		1,3190	0,0455	up	AJ947061 KN404_1 Sus scrofa cDNA clone C0007736j08 3', mRNA sequence
A_72_P015221	LAMC2	1,3176	0,0300	up	Sus scrofa laminin, gamma 2 (LAMC2), partial mRNA
A_72_P443129	PHEX	1,3162	0,0399	up	Sus scrofa phosphate regulating endopeptidase homolog, X-linked (PHEX), mRNA
A_72_P231717		1,3139	0,0024	up	AJ947061 KN404_1 Sus scrofa cDNA clone C0007736j08 3', mRNA sequence
A_72_P074131		1,3130	0,0452	up	324735 MARC 1PIG Sus scrofa cDNA 5', mRNA sequence
A_72_P345073		1,3111	0,0277	up	Sus scrofa mRNA, clone: OVRT10007C12, expressed in ovary
A_72_P102136		1,3111	0,0137	up	322379 MARC 1PIG Sus scrofa cDNA 5', mRNA sequence
A_72_P291904		1,3096	0,0036	up	817918 MARC 3PIG Sus scrofa cDNA 5', mRNA sequence
A_72_P035116	CDC45	1,3092	0,0197	up	Sus scrofa cell division cycle 45 (CDC45), mRNA
A_72_P693595	TMEM30A	1,3073	0,0144	up	Sus scrofa transmembrane protein 30A (TMEM30A), mRNA
A_72_P377733	LOC102160866	1,3072	0,0183	up	Sus scrofa uncharacterized LOC102160866 (LOC102160866), misc_RNA
A_72_P230777		1,3063	0,0495	up	Sus scrofa mRNA, clone:OVRM10109G02, expressed in ovary
A_72_P391593		1,3044	0,0385	up	866309 MARC 4PIG Sus scrofa cDNA 3', mRNA sequence
A_72_P712688	SLC35A3	1,3038	0,0313	up	Sus scrofa solute carrier family 35 (UDP-N-acetylglucosamine (UDP-GlcNAc) transporter), member A3 (SLC35A3), mRNA
A_72_P540403	S100A16	1,3035	0,0449	up	Sus scrofa S100 calcium binding protein A16 (S100A16), mRNA
A_72_P087651		1,3016	0,0407	up	1572153 MARC 3PIG Sus scrofa cDNA 3', mRNA sequence
A_72_P227182		1,3015	0,0113	up	BP461490 full-length enriched swine cDNA library, adult ovary Sus scrofa cDNA clone OVRM10190H02 5', mRNA sequence
A_72_P272449		1,3015	0,0466	up	AJ665946 CSEQRAN09 Sus scrofa cDNA clone C0000032_O16, mRNA sequence
A_72_P074001		1,3007	0,0160	up	BX925554 Sus Scrofa library (scan) Sus scrofa cDNA clone scan0021.k.05 5prim, mRNA sequence
A_72_P434394		1,2981	0,0393	up	BX925554 Sus Scrofa library (scan) Sus scrofa cDNA clone scan0021.k.05 5prim, mRNA sequence
A_72_P108176		1,2966	0,0109	up	Sus scrofa mRNA, clone:OVR010040E04, expressed in ovary
A_72_P305714	TFAM	1,2965	0,0393	up	Sus scrofa transcription factor A, mitochondrial (TFAM), mRNA
A_72_P125216	NCKAP1	1,2961	0,0342	up	Sus scrofa NCK-associated protein 1 (NCKAP1), mRNA
A_72_P163046	STX1A	1,2946	0,0307	up	Sus scrofa syntaxin 1A (brain) (STX1A), mRNA
A_72_P361888		1,2911	0,0139	up	234109 MARC 2PIG Sus scrofa cDNA 5', mRNA sequence
A_72_P570109	MTDH	1,2900	0,0442	up	Sus scrofa metadherin (MTDH), mRNA
A_72_P257229	RBM26	1,2899	0,0130	up	Sus scrofa RNA binding motif protein 26 (RBM26), mRNA
A_72_P641204	TDRD7	1,2888	0,0097	up	Sus scrofa tudor domain containing 7 (TDRD7), mRNA
A_72_P602603	ARHGAP11A	1,2884	0,0009	up	Sus scrofa Rho GTPase activating protein 11A (ARHGAP11A), mRNA
A_72_P742361		1,2863	0,0210	up	Sus scrofa complete mitochondrial DNA
A_72_P444255	PPP2R2A	1,2858	0,0479	up	Sus scrofa protein phosphatase 2, regulatory subunit B, alpha (PPP2R2A), mRNA
A_72_P752715		1,2854	0,0494	up	Sus scrofa complete mitochondrial DNA
A_72_P738029		1,2842	0,0408	up	Sus scrofa mitochondrion, complete genome
A_72_P313668	CRK	1,2835	0,0400	up	Sus scrofa v-crk sarcoma virus CT10 oncogene homolog (avian) (CRK), mRNA
A_72_P179751	RDH11	1,2826	0,0411	up	Sus scrofa retinol dehydrogenase 11 (all-trans/9-cis/11-cis) (RDH11), mRNA
A_72_P149281		1,2822	0,0094	up	943157 MARC 4PIG Sus scrofa cDNA 5', mRNA sequence
A_72_P071441	HPCAL4	1,2806	0,0210	up	Sus scrofa hippocalcin like 4 (HPCAL4), mRNA
A_72_P154136	ZNF445	1,2790	0,0022	up	Sus scrofa zinc finger protein 445 (ZNF445), mRNA
A_72_P580007	C5H12orf11	1,2774	0,0340	up	Sus scrofa chromosome 12 open reading frame 11 ortholog (C5H12orf11), mRNA
A_72_P035326	ENPEP	1,2766	0,0084	up	Sus scrofa glutamyl aminopeptidase (aminopeptidase A) (ENPEP), mRNA
A_72_P132466	NFXL1	1,2762	0,0236	up	Sus scrofa nuclear transcription factor, X-box binding-like 1 (NFXL1), mRNA

A_72_P055721		1,2748	0,0190	up	Pig DNA sequence from clone CH242-454E1 on chromosome 4, complete sequence
A_72_P543767	EIF2S1	1,2729	0,0426	up	Sus scrofa eukaryotic translation initiation factor 2, subunit 1 alpha, 35kDa (EIF2S1), mRNA
A_72_P725283		1,2726	0,0256	up	Sus scrofa mitochondrion, complete genome
A_72_P095841		1,2724	0,0203	up	ramn3510b_m9.y1 amn Sus scrofa cDNA 5', mRNA sequence
A_72_P273124		1,2711	0,0288	up	1572764 MARC 3PIG Sus scrofa cDNA 3', mRNA sequence
A_72_P366478	TRDMT1	1,2705	0,0003	up	Sus scrofa tRNA aspartic acid methyltransferase 1 (TRDMT1), mRNA
A_72_P221152		1,2702	0,0227	up	1512348 MARC 3PIG Sus scrofa cDNA 3', mRNA sequence
A_72_P016366	WDR3	1,2664	0,0345	up	Sus scrofa WD repeat domain 3 (WDR3), mRNA
A_72_P345358	ETFA	1,2654	0,0468	up	Sus scrofa electron-transfer-flavoprotein, alpha polypeptide (ETFA), mRNA
A_72_P042086		1,2650	0,0172	up	Sus scrofa mRNA, clone:UTR010043B06, expressed in uterus
A_72_P102016	ARHGAP29	1,2649	0,0237	up	Sus scrofa Rho GTPase activating protein 29 (ARHGAP29), mRNA
A_72_P356953	ZBTB1	1,2642	0,0010	up	Sus scrofa zinc finger and BTB domain containing 1 (ZBTB1), mRNA
A_72_P207947	ABL2	1,2640	0,0484	up	PREDICTED: Sus scrofa ABL proto-oncogene 2, non-receptor tyrosine kinase (ABL2), transcript variant X8, mRNA
A_72_P092336		1,2625	0,0434	up	998973 MARC 4PIG Sus scrofa cDNA 3', mRNA sequence
A_72_P337793		1,2624	0,0382	up	892055 MARC 4PIG Sus scrofa cDNA 3', mRNA sequence
A_72_P304814	UGGT1	1,2610	0,0448	up	Sus scrofa UDP-glucose glycoprotein glucosyltransferase 1 (UGGT1), mRNA
A_72_P122311		1,2603	0,0124	up	AJ956967 KN404_2 Sus scrofa cDNA clone C0007756j13 3', mRNA sequence
A_72_P316923	FSD1L	1,2600	0,0167	up	Sus scrofa fibronectin type III and SPRY domain containing 1-like (FSD1L), mRNA
A_72_P216227	TPRKB	1,2600	0,0298	up	Sus scrofa TP53RK binding protein (TPRKB), mRNA
A_72_P364388		1,2580	0,0359	up	Sus scrofa clone KVL15634 microsatellite sequence
A_72_P210362		1,2578	0,0476	up	UMC-pmpIlg_0-008-e06 Pig Metaphase II Gilt/MU pmpIlg Sus scrofa cDNA 3', mRNA sequence
A_72_P216212		1,2567	0,0448	up	rthg14_p5.y1 thg Sus scrofa cDNA 5', mRNA sequence
A_72_P128586	BMS1	1,2554	0,0464	up	Sus scrofa BMS1 ribosome biogenesis factor (BMS1), mRNA
A_72_P386443		1,2548	0,0126	up	AJ941456 CSEQRAN17 Sus scrofa cDNA clone C0007305a16 3', mRNA sequence
A_72_P004991		1,2547	0,0085	up	Pig DNA sequence from clone CH242-239D10 on chromosome X, complete sequence
A_72_P006016	MYBL2	1,2531	0,0134	up	Sus scrofa v-myb myeloblastosis viral oncogene homolog (avian)-like 2 (MYBL2), mRNA
A_72_P219647	FAM83C	1,2502	0,0203	up	Sus scrofa family with sequence similarity 83, member C (FAM83C), mRNA
A_72_P020486		1,2502	0,0329	up	
A_72_P125516	FAM196B	1,2480	0,0326	up	Sus scrofa family with sequence similarity 196, member B (FAM196B), mRNA
A_72_P172681	XKR5	1,2467	0,0280	up	Sus scrofa XK, Kell blood group complex subunit-related family, member 5 (XKR5), mRNA
A_72_P135131		1,2447	0,0319	up	Sus scrofa mRNA, clone: BFLT10037B05, expressed in brain (frontal lobe)
A_72_P455672	HCFC2	1,2441	0,0176	up	Sus scrofa host cell factor C2 (HCFC2), mRNA
A_72_P422559		1,2429	0,0441	up	Sus scrofa mRNA, clone:OVRM10168A06, expressed in ovary
A_72_P003481	CROT	1,2419	0,0086	up	Sus scrofa carnitine O-octanoyltransferase (CROT), mRNA
A_72_P002476	C7H15orf29	1,2417	0,0442	up	Sus scrofa chromosome 15 open reading frame 29 (C7H15orf29), mRNA
A_72_P252987		1,2416	0,0111	up	
A_72_P354143	PAPOLA	1,2396	0,0390	up	Sus scrofa poly(A) polymerase alpha (PAPOLA), mRNA
A_72_P553436	RUSC1	1,2380	0,0290	up	Sus scrofa RUN and SH3 domain containing 1 (RUSC1), mRNA
A_72_P374203		1,2378	0,0345	up	
A_72_P663877	MED23	1,2351	0,0225	up	Sus scrofa mediator complex subunit 23 (MED23), partial mRNA
A_72_P300114	FANCA	1,2336	0,0177	up	Sus scrofa Fanconi anemia, complementation group A (FANCA), mRNA
A_72_P225637	LOC102164478	1,2329	0,0168	up	Sus scrofa uncharacterized LOC102164478 (LOC102164478), misc_RNA
A_72_P443065		1,2327	0,0396	up	Sus scrofa mRNA, clone:OVRM10182E03, expressed in ovary
A_72_P741664		1,2319	0,0106	up	Sus scrofa mitochondrion, complete genome
A_72_P041866	CCDC90B	1,2318	0,0442	up	Sus scrofa coiled-coil domain containing 90B (CCDC90B), mRNA
A_72_P242357	TAF2	1,2305	0,0050	up	Sus scrofa TAF2 RNA polymerase II, TATA box binding protein (TBP)-associated factor, 150kDa (TAF2), mRNA
A_72_P743581	DENR	1,2302	0,0398	up	Sus scrofa density-regulated protein (DENR), mRNA
A_72_P337983		1,2292	0,0310	up	Pig DNA sequence from clone CH242-198K1 on chromosome X, complete sequence
A_72_P069516		1,2285	0,0040	up	Sus scrofa mRNA, clone: PBL010084A03, expressed in peripheral blood lymphocytes
A_72_P429429		1,2279	0,0478	up	AJ958574 KN404_2 Sus scrofa cDNA clone C0007758i04 5', mRNA sequence
A_72_P408073	CLPX	1,2275	0,0288	up	Sus scrofa caseinolytic mitochondrial matrix peptidase chaperone subunit (CLPX), mRNA
A_72_P080626		1,2270	0,0210	up	HX233885 full-length enriched swine cDNA library, adult inguinal lymph node Sus scrofa cDNA clone ILNT10089F05, mRNA sequence
A_72_P490208	TAF5	1,2267	0,0072	up	Sus scrofa TAF5 RNA polymerase II, TATA box binding protein (TBP)-associated factor, 100kDa (TAF5), mRNA
A_72_P753106		1,2255	0,0467	up	Sus scrofa mitochondrion, complete genome
A_72_P264912		1,2255	0,0072	up	258058 MARC 2PIG Sus scrofa cDNA 5', mRNA sequence
A_72_P772081		1,2254	0,0233	up	Sus scrofa mitochondrion, complete genome
A_72_P213257	MYCBP	1,2253	0,0073	up	Sus scrofa c-myc binding protein (MYCBP), mRNA
A_72_P536495		1,2240	0,0236	up	Sus scrofa mRNA, clone: BFLT10095E07, expressed in brain (frontal lobe)
A_72_P070096	ABCE1	1,2236	0,0181	up	Sus scrofa ATP-binding cassette, sub-family E (OABP), member 1 (ABCE1), mRNA
A_72_P492748	AP4E1	1,2236	0,0257	up	Sus scrofa adaptor-related protein complex 4, epsilon 1 subunit (AP4E1), mRNA
A_72_P152056		1,2234	0,0175	up	Sus scrofa mRNA, clone:LNG010057A05, expressed in lung
A_72_P497594	SF3B1	1,2213	0,0193	up	PREDICTED: Sus scrofa splicing factor 3b, subunit 1, 155kDa (SF3B1), transcript variant X1, mRNA
A_72_P179281	ASCC3	1,2200	0,0114	up	Sus scrofa activating signal cointegrator 1 complex subunit 3 (ASCC3), mRNA
A_72_P130971		1,2193	0,0443	up	1572748 MARC 3PIG Sus scrofa cDNA 3', mRNA sequence
A_72_P365378	CPEB2	1,2190	0,0152	up	Sus scrofa cytoplasmic polyadenylation element binding protein 2 (CPEB2), mRNA
A_72_P212087	LOC100522644	1,2189	0,0221	up	Sus scrofa uncharacterized LOC100522644 (LOC100522644), misc_RNA
A_72_P158086	ARFIP1	1,2183	0,0059	up	Sus scrofa ADP-ribosylation factor interacting protein 1 (ARFIP1), mRNA
A_72_P277109		1,2175	0,0361	up	rcst12_h13.y1 cst Sus scrofa cDNA 5', mRNA sequence
A_72_P004791		1,2168	0,0491	up	MI-P-H3-adl-a-05-1-UM.s1 MI-P-H3 Sus scrofa cDNA clone MI-P-H3-adl-a-05-1-UM 3', mRNA sequence
A_72_P502256		1,2156	0,0421	up	rnbm09b_a20.y1 nbm Sus scrofa cDNA 5', mRNA sequence

A_72_P106921	WDR35	1,2126	0,0498	up	Sus scrofa WD repeat domain 35 (WDR35), mRNA
A_72_P180726	VARS	1,2126	0,0224	up	Sus scrofa valyl-tRNA synthetase (VARS), mRNA
A_72_P032746		1,2125	0,0410	up	Sus scrofa mRNA, clone:OVR010040B12, expressed in ovary
A_72_P679684		1,2120	0,0380	up	Sus scrofa mRNA, clone: SPL010090H05, expressed in spleen
A_72_P015071		1,2113	0,0238	up	1555810 MARC 3PIG Sus scrofa cDNA 3', mRNA sequence
A_72_P252022		1,2110	0,0310	up	1520836 MARC 3PIG Sus scrofa cDNA 3', mRNA sequence
A_72_P388463		1,2107	0,0454	up	Pig DNA sequence from clone CH242-227A13 on chromosome 14, complete sequence
A_72_P114136		1,2098	0,0351	up	Pig DNA sequence from clone CH242-391F13 on chromosome 4, complete sequence
A_72_P544487	DBF4	1,2060	0,0084	up	Sus scrofa DBF4 homolog (S. cerevisiae) (DBF4), mRNA
A_72_P510905		1,2053	0,0459	up	Sus scrofa mRNA, clone:UTR010069C12, expressed in uterus
A_72_P227347		1,2037	0,0212	up	815526 MARC 3PIG Sus scrofa cDNA 3', mRNA sequence
A_72_P404478	LARP4	1,2015	0,0327	up	Sus scrofa La ribonucleoprotein domain family, member 4 (LARP4), mRNA
A_72_P217922		1,2008	0,0457	up	815526 MARC 3PIG Sus scrofa cDNA 3', mRNA sequence
A_72_P729778	ND1	1,1999	0,0314	up	NADH-ubiquinone oxidoreductase chain 1 [Source:UniProtKB/Swiss-Prot;Acc:O79874] [ENSSSCT00000019660]
A_72_P384403	DCAF13	1,1994	0,0092	up	DDB1 and CUL4 associated factor 13 [Source:HGNC Symbol;Acc:24535] [ENSSSCT00000006637]
A_72_P406428	SERINC2	1,1984	0,0500	up	Sus scrofa serine incorporator 2 (SERINC2), mRNA [NM_001244148]
A_72_P725783	CYTB	1,1975	0,0478	up	cytochrome b [Source:RefSeq peptide;Acc:NP_008646] [ENSSSCT00000019689]
A_72_P643304	HRSP12	1,1961	0,0439	up	heat-responsive protein 12 [Source:HGNC Symbol;Acc:16897] [ENSSSCT00000006670]
A_72_P288029		1,1922	0,0300	up	Sus scrofa mRNA, clone:LVRM10003A02, expressed in liver. [AK232675]
A_72_P171441		1,1921	0,0498	up	
A_72_P024821		1,1918	0,0289	up	family with sequence similarity 133, member B [Source:HGNC Symbol;Acc:28629] [ENSSSCT00000016690]
A_72_P363218	CA5B	1,1897	0,0363	up	carbonic anhydrase VB, mitochondrial [Source:HGNC Symbol;Acc:1378] [ENSSSCT00000034853]
A_72_P228577		1,1892	0,0375	up	Rep: Mitochondrial-processing peptidase subunit alpha, mitochondrial precursor - Homo sapiens (Human), partial (20%) [TC564485]
A_72_P020856		1,1882	0,0194	up	Rep: 3'-5' exonuclease component of the exosome - Chlamydomonas reinhardtii, partial (7%) [TC544894]
A_72_P441439	ERO1L	1,1881	0,0446	up	Sus scrofa ERO1-like (S. cerevisiae) (ERO1L), mRNA [NM_001137627]
A_72_P340148		1,1866	0,0355	up	
A_72_P050301	IREB2	1,1846	0,0141	up	Sus scrofa iron-responsive element binding protein 2 (IREB2), mRNA [NM_001167781]
A_72_P587836	VCL	1,1825	0,0479	up	Sus scrofa vinculin (VCL), mRNA [NM_213934]
A_72_P601633	PGRMC1	1,1815	0,0188	up	Sus scrofa progesterone receptor membrane component 1 (PGRMC1), mRNA [NM_213911]
A_72_P346132		1,1813	0,0362	up	MI-P-AY1-nqw-a-12-0-UI.s1 MI-P-AY1 Sus scrofa cDNA clone MI-P-AY1-nqw-a-12-0-UI 3', mRNA sequence [BI400387]
A_72_P493667	TIPIN	1,1802	0,0280	up	TIMELESS interacting protein [Source:HGNC Symbol;Acc:30750] [ENSSSCT00000005454]
A_72_P584606	ETFA	1,1801	0,0133	up	Sus scrofa electron-transfer-flavoprotein, alpha polypeptide (ETFA), mRNA [NM_001244403]
A_72_P312938		1,1799	0,0385	up	Rep: Zinc transporter 9 - Pongo pygmaeus (Orangutan), partial (27%) [TC560316]
A_72_P269114		1,1791	0,0466	up	Sus scrofa mRNA, clone:MLN010084D05, expressed in mesenteric lymph node. [AK233976]
A_72_P315823		1,1787	0,0056	up	Rep: VACUOLAR ATP SYNTHASE SUBUNIT C - Encephalitozoon cuniculi, partial (5%) [TC609086]
A_72_P189881		1,1785	0,0329	up	Sus scrofa mRNA, clone:PTG010054A01, expressed in pituitary gland. [AK349352]
A_72_P083366		1,1782	0,0458	up	48036 MARC 1PIG Sus scrofa cDNA 5', mRNA sequence [AW482851]
A_72_P315768		1,1767	0,0417	up	
A_72_P362643		1,1762	0,0262	up	Sus scrofa mRNA, clone:OVRM10182B05, expressed in ovary. [AK348490]
A_72_P027711		1,1738	0,0430	up	Rep: nucleoporin 133kDa - Bos Taurus, partial (29%) [TC594340]
A_72_P569064		1,1709	0,0179	up	Rep: Conserved membrane protein, multidrug efflux associated - Synechococcus sp. RS9916, partial (6%) [TC549730]
A_72_P587986	EBAG9	1,1699	0,0450	up	estrogen receptor binding site associated, antigen, 9 [Source:HGNC Symbol;Acc:3123] [ENSSSCT00000006611]
A_72_P187213		1,1691	0,0272	up	POSM0605011_G22R porcine skeletal muscle cDNA library (PoSM) Sus scrofa cDNA 3', mRNA sequence [DV899440]
A_72_P273319		1,1688	0,0480	up	cleavage and polyadenylation specific factor 1, 160kDa [Source:HGNC Symbol;Acc:2324] [ENSSSCT00000033818]
A_72_P502685	MT-2B	1,1665	0,0373	up	rcki012_d23.y1 cki Sus scrofa cDNA 5', mRNA sequence [EV949314]
A_72_P491744	SYNCRIP	1,1657	0,0373	up	Sus scrofa mRNA, clone:MLN010075A08, expressed in mesenteric lymph nodes. [AK346917]
A_72_P478846	DCAF4	1,1654	0,0175	up	Uncharacterized protein [Source:UniProtKB/TrEMBL;Acc:F1S3L1] [ENSSSCT00000025390]
A_72_P122066		1,1654	0,0417	up	Rep: Peptidase M56, BlaR1 precursor - Caulobacter sp. K31, partial (4%) [TC560927]
A_72_P402023		1,1644	0,0136	up	
A_72_P132526	NAA35	1,1636	0,0340	up	N(alpha)-acetyltransferase 35, NatC auxiliary subunit [Source:HGNC Symbol;Acc:24340] [ENSSSCT00000011992]
A_72_P379728		1,1631	0,0294	up	Rep: Cytochrome c-type biogenesis protein ccmE - Pelagibacter ubique, partial (14%) [TC528085]
A_72_P199712		1,1599	0,0000	up	MI-P-H3-adl-e-03-1-UM.s1 MI-P-H3 Sus scrofa cDNA clone MI-P-H3-adl-e-03-1-UM 3', mRNA sequence [BF712745]
A_72_P007551		1,1577	0,0175	up	rthg19_l3.y1 thg Sus scrofa cDNA 5', mRNA sequence [EW643975]
A_72_P187966		1,1563	0,0213	up	AJ948602 KN404_1 Sus scrofa cDNA clone C0007746b05 3', mRNA sequence [AJ948602]
A_72_P158496	NUDCD1	1,1558	0,0004	up	Sus scrofa mRNA, clone:AMP010014H05, expressed in alveolar macrophage. [AK230599]
A_72_P364318		1,1555	0,0120	up	Rep: Tubulin beta-2B chain - Homo sapiens (Human), partial (47%) [TC553963]
A_72_P092506		1,1521	0,0207	up	Rep: PREDICTED: chromosome transmission fidelity factor 8 protein isoform 2 - Pan troglodytes, complete [TC543210]
A_72_P363303		1,1497	0,0394	up	Rep: Glutamate--cysteine ligase GSHA - Frankia alni (strain ACN14a), partial (4%) [TC585473]
A_72_P207257		1,1491	0,0287	up	
A_72_P086886		1,1488	0,0136	up	Sus scrofa mRNA, clone: LVRM10140A02, expressed in liver. [AK394281]
A_72_P312388		1,1471	0,0066	up	AJ961381 KN404_2 Sus scrofa cDNA clone C0007751g04 3', mRNA sequence [AJ961381]
A_72_P406188	MTRR	1,1458	0,0379	up	5-methyltetrahydrofolate-homocysteine methyltransferase reductase [Source:HGNC Symbol;Acc:7473] [ENSSSCT00000018618]

A_72_P493552	KIAA1468	1,1449	0,0322	up	PREDICTED: Sus scrofa lisH domain and HEAT repeat-containing protein KIAA1468-like (LOC100521354), mRNA [XM_003121702]
A_72_P293389		1,1438	0,0221	up	BX918737 Sus Scrofa library (scan) Sus scrofa cDNA clone scan0035.c.18 5prim, mRNA sequence [BX918737]
A_72_P742844		1,1435	0,0459	up	Histone H3.3 [Source:UniProtKB/Swiss-Prot;Acc:Q71LE2] [ENSSSCT00000018727]
A_72_P396473		1,1406	0,0295	up	
A_72_P020069	MMP11	1,1405	0,0109	up	matrix metallopeptidase 11 (stromelysin 3) [Source:HGNC Symbol;Acc:7157] [ENSSSCT00000011032]
A_72_P565069	IREB2	1,1395	0,0116	up	Sus scrofa iron-responsive element binding protein 2 (IREB2), mRNA [NM_001167781]
A_72_P265942		1,1392	0,0406	up	1101965 MARC 4PIG Sus scrofa cDNA 5', mRNA sequence [DN105690]
A_72_P544799	SET	1,1390	0,0188	up	Sus scrofa SET nuclear oncogene (SET), mRNA [NM_001244090]
A_72_P259617		1,1349	0,0271	up	
A_72_P077581	NGB	1,1325	0,0170	up	Sus scrofa neuroglobin (NGB), mRNA [NM_001001647]
A_72_P729538	RPL6	1,1318	0,0182	up	Sus scrofa ribosomal protein L6 (RPL6), mRNA [NM_001044542]
A_72_P030316		1,1302	0,0155	up	Sus scrofa mRNA, clone:UTR010013D09, expressed in uterus. [AK352150]
A_72_P433539	ASB3	1,1300	0,0120	up	Sus scrofa ankyrin repeat and SOCS box containing 3 (ASB3), mRNA [NM_001205042]
A_72_P632636	LOC100153343	1,1282	0,0193	up	RWD domain containing 1 [Source:HGNC Symbol;Acc:20993] [ENSSSCT00000004911]
A_72_P774083		1,1259	0,0317	up	
A_72_P147946	FEM1B	1,1245	0,0433	up	fem-1 homolog b (C. elegans) [Source:HGNC Symbol;Acc:3649] [ENSSSCT00000031923]
A_72_P774331		1,1243	0,0452	up	
A_72_P253667		1,1224	0,0149	up	
A_72_P679310	PITPNB	1,1189	0,0295	up	phosphatidylinositol transfer protein, beta [Source:HGNC Symbol;Acc:9002] [ENSSSCT00000010923]
A_72_P263042		1,1159	0,0262	up	Rep: Sarcoplasmic/endoplasmic reticulum calcium ATPase 1 (EC 3.6.3.8) (Calcium pump 1) (SERCA1) (SR Ca(2+)-ATPase 1) (Calcium-transporting ATPase sarcoplasmic reticulum type, fast twitch skeletal muscle isoform) (Endoplasmic reticulum class 1/2 Ca(2+) ATPase). - Canis familiaris, partial (13%) [TC555343]
A_72_P005001		1,1136	0,0327	up	ZFP28 zinc finger protein [Source:HGNC Symbol;Acc:17801] [ENSSSCT00000025504]
A_72_P362428		1,1123	0,0146	up	Rep: Chromosome undetermined SCAF10386, whole genome shotgun sequence - Tetraodon nigroviridis (Green puffer), partial (7%) [TC559328]
A_72_P323743	KIAA1522	1,1103	0,0266	up	KIAA1522 [Source:HGNC Symbol;Acc:29301] [ENSSSCT00000022969]
A_72_P386428		1,1100	0,0382	up	Rep: Thioredoxin domain-containing protein 1 precursor - Bos taurus (Bovine), partial (31%) [TC546228]
A_72_P661966	NKAPL	1,1075	0,0248	up	NFKB activating protein-like [Source:HGNC Symbol;Acc:21584] [ENSSSCT00000001303]
A_72_P029901		1,1009	0,0372	up	Rep: Oncogene tlm - Mus musculus (Mouse), partial (10%) [TC539787]
A_72_P706492		1,1001	0,0328	up	
A_72_P290034	HSPB3	3,3826	0,0122	down	Sus scrofa heat shock 27kDa protein 3 (HSPB3), mRNA
A_72_P144946	LOC100525680	2,8864	0,0061	down	Sus scrofa serum amyloid A-2 protein-like (LOC100525680), mRNA
A_72_P164846		2,5949	0,0253	down	PBL_3_A09 Porcine Brain Library Sus scrofa cDNA clone PBL 5', mRNA sequence
A_72_P630178	CGA	2,5578	0,0428	down	Sus scrofa glycoprotein hormones, alpha polypeptide (CGA), transcript variant X1, mRNA
A_72_P077636	PDYN	2,5462	0,0161	down	Sus scrofa prodynorphin (PDYN), mRNA
A_72_P181711		2,5116	0,0333	down	MI-P-CP1-nwf-d-09-0-UI.s1 MI-P-CP1 Sus scrofa cDNA clone MI-P-CP1-nwf-d-09-0-UI 3', mRNA sequence
A_72_P413428	LOC100738180	2,4598	0,0458	down	Sus scrofa uncharacterized LOC100738180 (LOC100738180), mRNA
A_72_P465435	PTCHD1	2,3707	0,0247	down	Sus scrofa patched domain containing 1 (PTCHD1), mRNA
A_72_P403313		2,3622	0,0467	down	rjej26b_o7.y1 jej Sus scrofa cDNA 5', mRNA sequence
A_72_P077321	CCL28	2,3436	0,0314	down	Sus scrofa chemokine (C-C motif) ligand 28 (CCL28), mRNA
A_72_P577553	RLN	2,3069	0,0361	down	Sus scrofa relaxin (RLN), mRNA
A_72_P223367	RHCG	2,2921	0,0347	down	Sus scrofa Rh family, C glycoprotein (RHCG), mRNA
A_72_P320373		2,2882	0,0264	down	BP435556 full-length enriched swine cDNA library, adult lung Sus scrofa cDNA clone LNG010031C08 5', mRNA sequence
A_72_P760975	SAA3	2,2733	0,0079	down	Sus scrofa serum amyloid A-3 protein (SAA3), mRNA
A_72_P165616	SAA1P	2,2615	0,0090	down	Sus scrofa serum serum amyloid A-1 pseudogene (SAA1P) on chromosome 2
A_72_P275444	F13A1	2,2590	0,0369	down	Sus scrofa coagulation factor XIII, A1 polypeptide (F13A1), mRNA
A_72_P766152	LOC733603	2,2470	0,0078	down	Sus scrofa serum amyloid A2 (LOC733603), mRNA
A_72_P060076	APCS	2,2331	0,0184	down	PREDICTED: Sus scrofa amyloid P component, serum (APCS), mRNA
A_72_P441669	LOC733603	2,2204	0,0046	down	Sus scrofa serum amyloid A2 (LOC733603), mRNA
A_72_P195572	ALDH1L2	2,2146	0,0282	down	Sus scrofa aldehyde dehydrogenase 1 family, member L2 (ALDH1L2), mRNA
A_72_P489219	RHOBTB1	2,1773	0,0311	down	Sus scrofa Rho-related BTB domain containing 1 (RHOBTB1), mRNA
A_72_P232367	CXCL8	2,1587	0,0251	down	Sus scrofa chemokine (C-X-C motif) ligand 8 (CXCL8), mRNA
A_72_P322704	CYP3A46	2,1181	0,0043	down	Sus scrofa cytochrome P450 3A46 (CYP3A46), mRNA
A_72_P164366		2,0886	0,0008	down	MI-P-CP1-nwr-c-12-0-UI.s1 MI-P-CP1 Sus scrofa cDNA clone MI-P-CP1-nwr-c-12-0-UI 3', mRNA sequence
A_72_P404993	RFTN1	2,0875	0,0080	down	Sus scrofa raftlin, lipid raft linker 1 (RFTN1), mRNA
A_72_P251027		2,0839	0,0153	down	1520831 MARC 3PIG Sus scrofa cDNA 3', mRNA sequence
A_72_P222402		2,0837	0,0265	down	Sus scrofa mRNA, clone: HTMT10025H08, expressed in hypothalamus
A_72_P444532	P14ARF	2,0700	0,0401	down	Sus scrofa p14ARF protein (P14ARF), mRNA
A_72_P070401	CD79A	2,0508	0,0301	down	Sus scrofa CD79a molecule, immunoglobulin-associated alpha (CD79A), mRNA
A_72_P478841	DPF3	2,0312	0,0027	down	Sus scrofa D4, zinc and double PHD fingers, family 3 (DPF3), mRNA
A_72_P614721	SDR16C5	2,0254	0,0079	down	Sus scrofa short chain dehydrogenase/reductase family 16C, member 5 (SDR16C5), mRNA
A_72_P381283	MFAP2	2,0230	0,0022	down	Sus scrofa microfibrillar-associated protein 2 (MFAP2), mRNA
A_72_P285314	POPDC3	2,0139	0,0433	down	Sus scrofa popeye domain containing 3 (POPDC3), mRNA
A_72_P413648	NOS2	2,0080	0,0010	down	Sus scrofa nitric oxide synthase 2, inducible (NOS2), mRNA
A_72_P194302		1,9974	0,0108	down	UMC-pgvo2-010-h01 pgvo Sus scrofa cDNA 3', mRNA sequence
A_72_P153356	SLC22A5	1,9965	0,0168	down	Sus scrofa solute carrier family 22 (organic cation/carnitine transporter), member 5 (SLC22A5), mRNA
A_72_P107401	ALG12	1,9958	0,0399	down	Sus scrofa ALG12, alpha-1,6-mannosyltransferase (ALG12), mRNA
A_72_P511075	CHI3L1	1,9883	0,0094	down	Sus scrofa chitinase 3-like 1 (cartilage glycoprotein-39) (CHI3L1), mRNA
A_72_P443413	TNFRSF11B	1,9866	0,0063	down	Sus scrofa tumor necrosis factor receptor superfamily, member 11b (TNFRSF11B), mRNA
A_72_P360403	PTGS1	1,9821	0,0134	down	Sus scrofa prostaglandin-endoperoxide synthase 1 (prostaglandin G/H synthase and cyclooxygenase) (PTGS1), mRNA

A_72_P065401		1,9778	0,0244	down	AJ648907 CSEQRAN19 Sus scrofa cDNA clone C0003265_P16, mRNA sequence
A_72_P016761	C6H1orf87	1,9765	0,0035	down	Sus scrofa chromosome 6 open reading frame, human C1orf87 (C6H1orf87), transcript variant X5, mRNA
A_72_P225652	BATF3	1,9737	0,0022	down	Sus scrofa basic leucine zipper transcription factor, ATF-like 3 (BATF3), mRNA
A_72_P602303	PDLIM3	1,9732	0,0170	down	Sus scrofa PDZ and LIM domain 3 (PDLIM3), mRNA
A_72_P075046	IL9	1,9718	0,0334	down	Sus scrofa interleukin 9 (IL9), mRNA
A_72_P379423		1,9572	0,0246	down	UMC-p4mm3-021-b02 4mm ovarian follicle p4mm Sus scrofa cDNA 3', mRNA sequence
A_72_P326128	AMCF-II	1,9478	0,0448	down	Sus scrofa alveolar macrophage-derived chemotactic factor-II (AMCF-II), mRNA
A_72_P032606	SERPINB10	1,9249	0,0200	down	Sus scrofa serpin peptidase inhibitor, clade B (ovalbumin), member 10 (SERPINB10), mRNA
A_72_P212987		1,9140	0,0330	down	909449 MARC 4PIG Sus scrofa cDNA 5', mRNA sequence
A_72_P059201		1,9123	0,0186	down	MI-P-CP1-nwx-g-03-0-UI.s1 MI-P-CP1 Sus scrofa cDNA clone MI-P-CP1-nwx-g-03-0-UI 3', mRNA sequence
A_72_P162741	PTPLA	1,9098	0,0093	down	Sus scrofa protein tyrosine phosphatase-like (proline instead of catalytic arginine), member A (PTPLA), mRNA
A_72_P080101	TLE4	1,9041	0,0339	down	Sus scrofa transducin-like enhancer of split 4 (E(sp1) homolog, Drosophila) (TLE4), mRNA
A_72_P424114	CCNT1	1,9029	0,0484	down	Sus scrofa cyclin T1 (CCNT1), mRNA
A_72_P169901		1,9009	0,0036	down	AJ950876 KN404_1 Sus scrofa cDNA clone C0007744f20 5', mRNA sequence
A_72_P088641	AQP3	1,8861	0,0330	down	Sus scrofa aquaporin 3 (Gill blood group) (AQP3), mRNA
A_72_P075861	LOC100739146	1,8793	0,0201	down	Sus scrofa probable tubulin polyglutamylase TTLL9-like (LOC100739146), partial mRNA
A_72_P490025	SFRP5	1,8759	0,0359	down	Sus scrofa secreted frizzled-related protein 5 (SFRP5), mRNA
A_72_P106541	YPEL1	1,8752	0,0180	down	Sus scrofa yippee-like 1 (Drosophila) (YPEL1), mRNA
A_72_P260457		1,8723	0,0447	down	CJ017662 full-length enriched swine cDNA library, adult spleen Sus scrofa cDNA clone SPL01A070052 5', mRNA sequence
A_72_P077336	PRLR	1,8713	0,0056	down	Sus scrofa prolactin receptor (PRLR), mRNA
A_72_P232692	FBP1	1,8688	0,0319	down	Sus scrofa fructose-1,6-bisphosphatase 1 (FBP1), mRNA
A_72_P078151		1,8674	0,0289	down	BX926728 Sus Scrofa library (scan) Sus scrofa cDNA clone scan0014.o.15 5prim, mRNA sequence
A_72_P329118	C3H2orf40	1,8667	0,0302	down	Sus scrofa chromosome 2 open reading frame 40 ortholog (C3H2orf40), mRNA
A_72_P176971	TNFRSF4	1,8630	0,0463	down	Sus scrofa tumor necrosis factor receptor superfamily, member 4 (TNFRSF4), mRNA
A_72_P220382		1,8588	0,0349	down	rhlv15b_i20.y1 hlv Sus scrofa cDNA 5', mRNA sequence
A_72_P124566	GDAP1	1,8499	0,0162	down	Sus scrofa ganglioside induced differentiation associated protein 1 (GDAP1), mRNA
A_72_P124881		1,8473	0,0243	down	BW967587 full-length enriched swine cDNA library, adult ovary Sus scrofa cDNA clone OVR010093B02 5', mRNA sequence
A_72_P354828		1,8441	0,0161	down	jns43_D11.f jns Sus scrofa cDNA 5', mRNA sequence
A_72_P008261		1,8365	0,0138	down	230306 MARC 2PIG Sus scrofa cDNA 5', mRNA sequence
A_72_P248472	DRAM1	1,8363	0,0484	down	Sus scrofa DNA-damage regulated autophagy modulator 1 (DRAM1), mRNA
A_72_P194247	LOC102167657	1,8333	0,0436	down	Sus scrofa uncharacterized LOC102167657 (LOC102167657), misc_RNA
A_72_P078756	ZNF652	1,8296	0,0276	down	Sus scrofa zinc finger protein 652 (ZNF652), mRNA
A_72_P385148		1,8277	0,0086	down	PADT08110O7_A13R porcine adipose tissue cDNA library (PADT) Sus scrofa cDNA 3', mRNA sequence
A_72_P290279		1,8236	0,0373	down	UMC-pgvo2-010-h01 pgvo Sus scrofa cDNA 3', mRNA sequence
A_72_P442067	OCT1	1,8164	0,0036	down	Sus scrofa organic cation transporter 1 (OCT1), mRNA
A_72_P444306	LOC100152327	1,8104	0,0224	down	Sus scrofa immunoglobulin lambda-like polypeptide 5 (LOC100152327), mRNA
A_72_P706576	FAM53B	1,8092	0,0386	down	Sus scrofa family with sequence similarity 53, member B (FAM53B), mRNA
A_72_P306173	LOC100621891	1,8086	0,0475	down	Sus scrofa uncharacterized LOC100621891 (LOC100621891), misc_RNA
A_72_P271869		1,7970	0,0386	down	800075 MARC 3PIG Sus scrofa cDNA 3', mRNA sequence
A_72_P333748		1,7951	0,0023	down	1512897 MARC 3PIG Sus scrofa cDNA 3', mRNA sequence
A_72_P381653	CHD3	1,7946	0,0174	down	Sus scrofa chromodomain helicase DNA binding protein 3 (CHD3), mRNA
A_72_P440611	ANGPTL7	1,7945	0,0436	down	Sus scrofa angiopoietin-like 7 (ANGPTL7), mRNA
A_72_P275359		1,7905	0,0303	down	Sus scrofa mRNA, clone:THY010074F05, expressed in thymus
A_72_P442989	SLC35D1	1,7866	0,0205	down	Sus scrofa solute carrier family 35 (UDP-glucuronic acid/UDP-N-acetylgalactosamine dual transporter), member D1 (SLC35D1), mRNA
A_72_P067721		1,7854	0,0296	down	AJ649189 CSEQRAN19 Sus scrofa cDNA clone C0003271_M21, mRNA sequence
A_72_P442714	TXNRD1	1,7844	0,0143	down	Sus scrofa thioredoxin reductase 1 (TXNRD1), mRNA
A_72_P078426	LOC102166799	1,7801	0,0164	down	Sus scrofa uncharacterized LOC102166799 (LOC102166799), misc_RNA
A_72_P008421		1,7731	0,0385	down	Sus scrofa mRNA, clone:THY010065B08, expressed in thymus
A_72_P516390		1,7693	0,0035	down	rcad05b_d10.y1 cag Sus scrofa cDNA 5', mRNA sequence
A_72_P232192	TNFR2	1,7691	0,0107	down	Sus scrofa tumor necrosis factor receptor 2 (TNFR2) mRNA, complete cds
A_72_P317938	SLC4A9	1,7660	0,0161	down	Sus scrofa solute carrier family 4, sodium bicarbonate cotransporter, member 9 (SLC4A9), mRNA
A_72_P271704	FREM1	1,7626	0,0016	down	Sus scrofa FRAS1 related extracellular matrix 1 (FREM1), mRNA
A_72_P736997	ACTA1	1,7597	0,0478	down	Sus scrofa actin, alpha 1, skeletal muscle (ACTA1), mRNA
A_72_P101791		1,7580	0,0073	down	Sus scrofa mRNA, clone:SKNB10031H12, expressed in skin
A_72_P364398	DIAPH1	1,7574	0,0038	down	Sus scrofa diaphanous homolog 1 (Drosophila) (DIAPH1), mRNA
A_72_P373988		1,7563	0,0449	down	998373 MARC 4PIG Sus scrofa cDNA 3', mRNA sequence
A_72_P516452	PYGM	1,7512	0,0001	down	Sus scrofa phosphorylase, glycogen, muscle (PYGM), mRNA
A_72_P470999	CLASP1	1,7511	0,0455	down	Sus scrofa cytoplasmic linker associated protein 1 (CLASP1), mRNA
A_72_P204422	KPNA7	1,7489	0,0087	down	Sus scrofa karyopherin alpha 7 (importin alpha 8) (KPNA7), mRNA
A_72_P300259		1,7301	0,0328	down	BX923227 Sus Scrofa library (scan) Sus scrofa cDNA clone scan0024.n.16 5prim, mRNA sequence
A_72_P608487	HIPK1	1,7288	0,0371	down	Sus scrofa homeodomain interacting protein kinase 1 (HIPK1), tmRNA
A_72_P425089	LOC100621782	1,7271	0,0421	down	Sus scrofa tripartite motif-containing protein 60-like (LOC100621782), mRNA
A_72_P132711	LOC100523712	1,7248	0,0492	down	Sus scrofa E3 ubiquitin-protein ligase TRIM32-like (LOC100523712), misc_RNA
A_72_P336828	ABCG8	1,7246	0,0256	down	Sus scrofa ATP-binding cassette, sub-family G (WHITE), member 8 (ABCG8), mRNA
A_72_P443272	ACSL1	1,7218	0,0218	down	Sus scrofa acyl-CoA synthetase long-chain family member 1 (ACSL1), mRNA
A_72_P352143	C3H16orf54	1,7178	0,0133	down	Sus scrofa chromosome 3 open reading frame, human C16orf54 (C3H16orf54), mRNA

A_72_P339448		1,7174	0,0052	down	AJ939468 CSEQRAN17 Sus scrofa cDNA clone C0007316g13 3', mRNA sequence
A_72_P201752	RSPH3	1,7073	0,0350	down	Sus scrofa radial spoke 3 homolog (Chlamydomonas) (RSPH3), partial mRNA
A_72_P444398	SLC22A3	1,7061	0,0341	down	Sus scrofa solute carrier family 22 (extraneuronal monoamine transporter), member 3 (SLC22A3), mRNA
A_72_P392133	FBXL4	1,7058	0,0106	down	Sus scrofa F-box and leucine-rich repeat protein 4 (FBXL4), mRNA
A_72_P274354	SYNM	1,7050	0,0076	down	Sus scrofa synemin, intermediate filament protein (SYNM), mRNA
A_72_P281634	JAM3	1,7029	0,0112	down	Sus scrofa junctional adhesion molecule 3 (JAM3), mRNA
A_72_P031841		1,7026	0,0040	down	Pig DNA sequence from clone CH242-196N13 on chromosome 7, complete sequence
A_72_P463511	IL1b	1,7012	0,0313	down	Sus scrofa interleukin-1 beta (IL-1beta), mRNA
A_72_P411833	SAMD4A	1,7004	0,0322	down	PREDICTED: Sus scrofa sterile alpha motif domain containing 4A (SAMD4A), transcript variant X3, mRNA
A_72_P010351	KLHL5	1,6960	0,0351	down	Sus scrofa kelch-like family member 5 (KLHL5), mRNA
A_72_P357108		1,6947	0,0402	down	Sus scrofa mRNA, clone: KDN010079F01, expressed in kidney.
A_72_P414318	HHEX	1,6892	0,0143	down	Sus scrofa hematopoietically expressed homeobox (HHEX), mRNA
A_72_P488884	KLHL22	1,6868	0,0367	down	Sus scrofa kelch-like family member 22 (KLHL22), mRNA
A_72_P272274		1,6866	0,0398	down	BP441774 full-length enriched swine cDNA library, adult liver Sus scrofa cDNA clone LVR010081A11 5', mRNA sequence
A_72_P076086	LOC100524097	1,6865	0,0098	down	Sus scrofa uncharacterized LOC100524097 (LOC100524097), mRNA
A_72_P021631	CD226	1,6844	0,0332	down	PREDICTED: Sus scrofa CD226 molecule (CD226), mRNA
A_72_P088816	PAG6	1,6838	0,0257	down	Sus scrofa pregnancy-associated glycoprotein 6 (PAG6), mRNA
A_72_P240922		1,6837	0,0381	down	UMC-puuo5-003-b05 5' Library puuo Sus scrofa cDNA 5', mRNA sequence
A_72_P063896	LOC100625296	1,6829	0,0444	down	Sus scrofa neurofilament heavy polypeptide-like (LOC100625296), misc_RNA
A_72_P472481	EPHA4	1,6818	0,0170	down	Sus scrofa EPH receptor A4 (EPHA4), mRNA
A_72_P418639		1,6785	0,0392	down	1138874 MARC 4PIG Sus scrofa cDNA 3', mRNA sequence
A_72_P078706	FKBP5	1,6740	0,0398	down	Sus scrofa FK506 binding protein 5 (FKBP5), mRNA
A_72_P241652	DPF3	1,6714	0,0240	down	PREDICTED: Sus scrofa D4, zinc and double PHD fingers, family 3 (DPF3), mRNA
A_72_P312773		1,6685	0,0202	down	122997 MARC 1PIG Sus scrofa cDNA 5', mRNA sequence
A_72_P145816		1,6649	0,0115	down	1516043 MARC 3PIG Sus scrofa cDNA 5', mRNA sequence
A_72_P286759	EIF2C1	1,6648	0,0164	down	Sus scrofa eukaryotic translation initiation factor 2C, 1 (EIF2C1), mRNA
A_72_P536730	NQO1	1,6641	0,0295	down	Sus scrofa NAD(P)H dehydrogenase, quinone 1 (NQO1), mRNA
A_72_P053626		1,6633	0,0173	down	risp33_g5.y1 isp Sus scrofa cDNA 5', mRNA sequence
A_72_P318948		1,6613	0,0440	down	rmed06c_k2.y1 med Sus scrofa cDNA 5', mRNA sequence
A_72_P178976	MARCKS	1,6601	0,0184	down	Sus scrofa myristoylated alanine-rich protein kinase C substrate (MARCKS), mRNA
A_72_P232632	PROC	1,6574	0,0103	down	Sus scrofa protein C (inactivator of coagulation factors Va and VIIIa) (PROC), mRNA
A_72_P055166	TMEM67	1,6546	0,0199	down	Sus scrofa transmembrane protein 67 (TMEM67), mRNA
A_72_P189551	DDX46	1,6508	0,0395	down	Sus scrofa DEAD (Asp-Glu-Ala-Asp) box polypeptide 46 (DDX46), mRNA
A_72_P425299		1,6500	0,0499	down	UMC-p4civv1-010-g01 4-Cell-Embryo-(invivo) p4civv Sus scrofa cDNA 3', mRNA sequence
A_72_P035541	CASP1	1,6476	0,0067	down	Sus scrofa caspase 1, apoptosis-related cysteine peptidase (CASP1), mRNA
A_72_P223332	CCL4	1,6475	0,0144	down	Sus scrofa chemokine (C-C motif) ligand 4 (CCL4), mRNA
A_72_P597409		1,6463	0,0463	down	Sus scrofa mRNA, clone: OVR010040D04, expressed in ovary.
A_72_P172528	FAM65B	1,6459	0,0042	down	Sus scrofa family with sequence similarity 65, member B (FAM65B), mRNA
A_72_P610794		1,6447	0,0467	down	Sus scrofa mRNA, clone:OVR010066H07, expressed in ovary
A_72_P140581	RGS14	1,6425	0,0296	down	Sus scrofa regulator of G-protein signaling 14 (RGS14), mRNA
A_72_P302739	ADRA2B	1,6425	0,0351	down	Sus scrofa adrenoceptor alpha 2B (ADRA2B), mRNA
A_72_P335903		1,6424	0,0228	down	MI-P-NA-aet-h-10-1-UM.s1 MI-P-NA Sus scrofa cDNA clone MI-P-NA-aet-h-10-1-UM 3', mRNA sequence
A_72_P061851		1,6392	0,0066	down	1515849 MARC 3PIG Sus scrofa cDNA 5', mRNA sequence
A_72_P698600	RNF111	1,6388	0,0460	down	Sus scrofa ring finger protein 111 (RNF111), mRNA
A_72_P674232	NCOA4	1,6385	0,0052	down	Sus scrofa nuclear receptor coactivator 4 (NCOA4), mRNA
A_72_P403188	RPS27	1,6368	0,0403	down	PREDICTED: Sus scrofa ribosomal protein S27 (RPS27), mRNA
A_72_P761755		1,6363	0,0137	down	rhyp09c_f20.y1 hyp Sus scrofa cDNA 5', mRNA sequence
A_72_P232232	PIK3R5	1,6348	0,0161	down	Sus scrofa phosphoinositide-3-kinase, regulatory subunit 5 (PIK3R5), mRNA
A_72_P257987	LOC100517569	1,6347	0,0478	down	Sus scrofa lysine-specific demethylase 4C-like (LOC100517569), misc_RNA
A_72_P633564	RAI14	1,6344	0,0156	down	Sus scrofa retinoic acid induced 14 (RAI14), mRNA
A_72_P706415	LOC100739291	1,6316	0,0233	down	Sus scrofa myosin light chain kinase, smooth muscle-like (LOC100739291), mRNA
A_72_P518459	RN18S	1,6307	0,0316	down	Sus scrofa 18S ribosomal RNA gene, complete sequence
A_72_P099401		1,6287	0,0345	down	AJ963622 KN404_2 Sus scrofa cDNA clone C0007756e22 3', mRNA sequence
A_72_P304694		1,6274	0,0393	down	1121923 MARC 4PIG Sus scrofa cDNA 3', mRNA sequence
A_72_P379243		1,6257	0,0477	down	FS657729 full-length enriched swine cDNA library, adult colon Sus scrofa cDNA clone CLNT10009A06 5', mRNA sequence
A_72_P057471	DNAJA4	1,6245	0,0185	down	Sus scrofa DnaJ (Hsp40) homolog, subfamily A, member 4 (DNAJA4), mRNA
A_72_P632736	LOC100294636	1,6240	0,0418	down	Sus scrofa RIIa domain-containing protein (LOC100294636), mRNA
A_72_P197812		1,6237	0,0100	down	AJ943557 CSEQRAN17 Sus scrofa cDNA clone C0007303g06 5', mRNA sequence
A_72_P442262	INHBA	1,6208	0,0496	down	Sus scrofa inhibin, beta A (INHBA), mRNA
A_72_P407208	DYNC1H1	1,6192	0,0055	down	Sus scrofa dynein, cytoplasmic 1, heavy chain 1 (DYNC1H1), mRNA
A_72_P237312	ADAM-9	1,6188	0,0202	down	Sus scrofa cellular disintegrin precursor (ADAM-9), mRNA
A_72_P178081	IFNGR2	1,6183	0,0229	down	Sus scrofa interferon gamma receptor 2 (interferon gamma transducer 1) (IFNGR2), mRNA
A_72_P372243	EPHA4	1,6169	0,0129	down	Sus scrofa EPH receptor A4 (EPHA4), mRNA
A_72_P169416		1,6149	0,0134	down	AJ947875 KN404_1 Sus scrofa cDNA clone C0007735e23 5', mRNA sequence
A_72_P176206	KLKB1	1,6121	0,0499	down	Sus scrofa kallikrein B, plasma (Fletcher factor) 1 (KLKB1), mRNA
A_72_P274979		1,6119	0,0179	down	1553931 MARC 3PIG Sus scrofa cDNA 3', mRNA sequence
A_72_P000071		1,6098	0,0350	down	806105 MARC 3PIG Sus scrofa cDNA 5', mRNA sequence
A_72_P499175		1,6097	0,0246	down	AR037D10PBC30BD10S Porcine Peripheral Blood Cell cDNA library, Cot 30 Sus scrofa cDNA, mRNA sequence
A_72_P179546	KLF11	1,6097	0,0136	down	Sus scrofa Kruppel-like factor 11 (KLF11), mRNA
A_72_P567124	POLG	1,6075	0,0427	down	Sus scrofa polymerase (DNA directed), gamma (POLG), mRNA
A_72_P219427	PIH1D3	1,6072	0,0004	down	Sus scrofa PIH1 domain containing 3 (PIH1D3), mRNA
A_72_P414548	LYVE1	1,6072	0,0468	down	Sus scrofa lymphatic vessel endothelial hyaluronan receptor 1 (LYVE1), mRNA
A_72_P327498	STXBP1	1,6067	0,0393	down	Sus scrofa syntaxin binding protein 1 (STXBP1), mRNA
A_72_P263047	DZIP1	1,6047	0,0202	down	Sus scrofa DAZ interacting zinc finger protein 1 (DZIP1), mRNA

A_72_P271269		1,6033	0,0113	down	rpigcf0_016227.y1 ctl Sus scrofa cDNA 5', mRNA sequence
A_72_P381078	SSH1	1,6029	0,0028	down	Sus scrofa slingshot protein phosphatase 1 (SSH1), mRNA
A_72_P170361	ZO1	1,6024	0,0139	down	Sus scrofa zonula occludens 1 (ZO1), mRNA
A_72_P165846	NFKBIA	1,5980	0,0425	down	Sus scrofa nuclear factor of kappa light polypeptide gene enhancer in B-cells inhibitor, alpha (NFKBIA), mRNA
A_72_P295144		1,5976	0,0032	down	893832 MARC 4PIG Sus scrofa cDNA 5', mRNA sequence
A_72_P010651	PMFBP1	1,5933	0,0273	down	Sus scrofa polyamine modulated factor 1 binding protein 1 (PMFBP1), mRNA
A_72_P026476	TPK1	1,5932	0,0358	down	Sus scrofa thiamin pyrophosphokinase 1 (TPK1), mRNA
A_72_P121686	SCFD2	1,5927	0,0444	down	Sus scrofa sec1 family domain containing 2 (SCFD2), mRNA
A_72_P218312	SOCS1	1,5919	0,0133	down	Sus scrofa suppressor of cytokine signaling 1 (SOCS1), mRNA
A_72_P251567	TCP11L2	1,5912	0,0039	down	Sus scrofa t-complex 11, testis-specific-like 2 (TCP11L2), mRNA
A_72_P478123	CEBPE	1,5911	0,0202	down	Sus scrofa CCAAT/enhancer binding protein (C/EBP), epsilon (CEBPE), mRNA
A_72_P423619	SLC6A1	1,5909	0,0461	down	Sus scrofa solute carrier family 6 (neurotransmitter transporter), member 1 (SLC6A1), mRNA
A_72_P316818	FADS2	1,5908	0,0264	down	Sus scrofa fatty acid desaturase 2 (FADS2), mRNA
A_72_P108641		1,5902	0,0427	down	BP463763 full-length enriched swine cDNA library, adult uterus Sus scrofa cDNA clone UTR010058D10 5', mRNA sequence
A_72_P117871		1,5886	0,0251	down	jns39_A04.f jns Sus scrofa cDNA 5', mRNA sequence
A_72_P224942		1,5881	0,0416	down	BX665628 Sus Scrofa library (scag) Sus scrofa cDNA clone scag0007.g.07 5prim, mRNA sequence
A_72_P612772	PHLPP1	1,5872	0,0089	down	Sus scrofa PH domain and leucine rich repeat protein phosphatase 1 (PHLPP1), mRNA
A_72_P710798	LOC100158011	1,5871	0,0024	down	Sus scrofa pancreatic trypsin inhibitor-like (LOC100158011), transcript variant 1, mRNA
A_72_P034196	SMAP2	1,5854	0,0034	down	Sus scrofa small ArfGAP2 (SMAP2), mRNA
A_72_P034506		1,5850	0,0317	down	Sus scrofa mRNA, clone:ADR010069G10, expressed in adrenal gland.
A_72_P444408	IGF2R	1,5848	0,0337	down	Sus scrofa insulin-like growth factor 2 receptor (IGF2R), mRNA
A_72_P427111	APLP2	1,5840	0,0034	down	Sus scrofa amyloid beta (A4) precursor-like protein 2 (APLP2), mRNA
A_72_P762749	MBP	1,5836	0,0162	down	Sus scrofa myelin basic protein (MBP), mRNA
A_72_P006351	SSH2	1,5835	0,0430	down	Sus scrofa slingshot protein phosphatase 2 (SSH2), partial mRNA
A_72_P362608		1,5822	0,0239	down	376287 MARC 2PIG Sus scrofa cDNA 5', mRNA sequence
A_72_P056591		1,5817	0,0464	down	856551 MARC 4PIG Sus scrofa cDNA 3', mRNA sequence
A_72_P320813	TNFAIP3	1,5813	0,0404	down	Sus scrofa tumor necrosis factor, alpha-induced protein 3 (TNFAIP3), mRNA
A_72_P237542	ATL3	1,5805	0,0039	down	Sus scrofa atlastin GTPase 3 (ATL3), mRNA
A_72_P730317		1,5790	0,0040	down	Sus scrofa mRNA, clone:LNG010007F06, expressed in lung.
A_72_P114516		1,5786	0,0222	down	Sus scrofa pSNORD3 cluster snoRNA, complete sequence
A_72_P336133	CDC25C	1,5777	0,0124	down	Sus scrofa cell division cycle 25 homolog C (S. pombe) (CDC25C), mRNA
A_72_P404923		1,5756	0,0460	down	921266 MARC 4PIG Sus scrofa cDNA 3', mRNA sequence
A_72_P478646	MCTP2	1,5733	0,0045	down	Sus scrofa multiple C2 domains, transmembrane 2 (MCTP2), mRNA
A_72_P040846		1,5718	0,0333	down	805259 MARC 3PIG Sus scrofa cDNA 5', mRNA sequence
A_72_P300294	ZNF576	1,5713	0,0112	down	Sus scrofa zinc finger protein 576 (ZNF576), mRNA
A_72_P453249	ATP4B	1,5710	0,0412	down	Sus scrofa ATPase, H+/K+ exchanging, beta polypeptide (ATP4B), mRNA
A_72_P106471	LOC100518000	1,5671	0,0170	down	PREDICTED: Sus scrofa nuclear envelope pore membrane protein POM 121-like (LOC100518000), mRNA
A_72_P034901		1,5649	0,0331	down	Sus scrofa mRNA, clone:PTG010071D08, expressed in pituitary gland
A_72_P477135	LOC100738047	1,5639	0,0375	down	Sus scrofa trem-like transcript 2 protein-like (LOC100738047), mRNA
A_72_P394248	TFAP2A	1,5634	0,0488	down	Sus scrofa transcription factor AP-2 alpha (activating enhancer binding protein 2 alpha) (TFAP2A), mRNA
A_72_P322783		1,5633	0,0094	down	Sus scrofa mRNA, clone:OVRM10116D09, expressed in ovary
A_72_P367488		1,5592	0,0499	down	1562924 MARC 3PIG Sus scrofa cDNA 5', mRNA sequence
A_72_P486627	MASP1	1,5569	0,0385	down	Sus scrofa mannan-binding lectin serine peptidase 1 (C4/C2 activating component of Ra-reactive factor) (MASP1), mRNA
A_72_P083911	KANSL1L	1,5558	0,0473	down	Sus scrofa KAT8 regulatory NSL complex subunit 1-like (KANSL1L), mRNA
A_72_P382248	LOC100156775	1,5554	0,0450	down	Sus scrofa solute carrier family 7 member 13-like (LOC100156775), mRNA
A_72_P075961	MTMR9	1,5548	0,0394	down	Sus scrofa myotubularin related protein 9 (MTMR9), mRNA
A_72_P405188		1,5542	0,0053	down	AJ669547 CSEQRAN09 Sus scrofa cDNA clone C000001059_N02, mRNA sequence
A_72_P401243	TMEM106C	1,5529	0,0260	down	Sus scrofa transmembrane protein 106C (TMEM106C), mRNA
A_72_P567564	CKM	1,5518	0,0365	down	Sus scrofa creatine kinase, muscle (CKM), mRNA
A_72_P304274	CLIC5	1,5513	0,0222	down	Sus scrofa chloride intracellular channel 5 (CLIC5), misc_RNA
A_72_P411188	ENPP4	1,5513	0,0297	down	Sus scrofa ectonucleotide pyrophosphatase/phosphodiesterase 4 (putative) (ENPP4), mRNA
A_72_P327188		1,5504	0,0418	down	Select seq gb|EW502890.2|
A_72_P327028	DYRK3	1,5503	0,0168	down	PREDICTED: Sus scrofa dual-specificity tyrosine-(Y)-phosphorylation regulated kinase 3 (DYRK3), partial mRNA
A_72_P763028		1,5502	0,0137	down	Chain 5, Structure Of The Idle Mammalian Ribosome-sec61 Complex. This Entry Contains The Large Subunit Ribosomal Rna
A_72_P083666		1,5482	0,0432	down	1497603 MARC 3PIG Sus scrofa cDNA 5', mRNA sequence
A_72_P077231	CDC42BPA	1,5476	0,0020	down	Sus scrofa CDC42 binding protein kinase alpha (DMPK-like) (CDC42BPA), mRNA
A_72_P364933		1,5457	0,0119	down	Sus scrofa mRNA, clone:THY010051H11, expressed in thymus
A_72_P115851	EIF4G3	1,5453	0,0050	down	PREDICTED: Sus scrofa eukaryotic translation initiation factor 4 gamma, 3 (EIF4G3), mRNA
A_72_P165956	PTPRU	1,5449	0,0328	down	Sus scrofa protein tyrosine phosphatase, receptor type, U (PTPRU), mRNA
A_72_P200472	LYZL1	1,5446	0,0429	down	Sus scrofa lysozyme-like 1 (LYZL1), mRNA
A_72_P374828		1,5439	0,0133	down	1539113 MARC 3PIG Sus scrofa cDNA 3', mRNA sequence
A_72_P265902		1,5418	0,0115	down	UMC-p4mm3-019-e10 4mm ovarian follicle p4mm Sus scrofa cDNA 3', mRNA sequence
A_72_P264457	SPATA18	1,5409	0,0066	down	Sus scrofa spermatogenesis associated 18 (SPATA18), mRNA
A_72_P062611		1,5404	0,0227	down	rcki09_g11.y1 cki Sus scrofa cDNA 5', mRNA sequence
A_72_P069541	RASGEF1B	1,5399	0,0015	down	RasGEF domain family, member 1B [Source:HGNC Symbol;Acc:24881] [ENSSSCT00000026377]
A_72_P705754		1,5394	0,0494	down	
A_72_P258467	IL24	1,5388	0,0373	down	Sus scrofa interleukin 24 (IL24), mRNA
A_72_P429264	KIF1B	1,5366	0,0122	down	Sus scrofa kinesin family member 1B (KIF1B), mRNA
A_72_P013161		1,5301	0,0249	down	PDUts2021G04 Porcine testis cDNA library II Sus scrofa cDNA clone PDUts2021G04 5', mRNA sequence
A_72_P426224	MED17	1,5295	0,0003	down	Sus scrofa mediator complex subunit 17 (MED17), mRNA
A_72_P311618		1,5265	0,0416	down	BX674626 Sus Scrofa library (scac) Sus scrofa cDNA clone scac0044.i.21 5prim, mRNA sequence
A_72_P078796		1,5250	0,0444	down	1541586 MARC 3PIG Sus scrofa cDNA 3', mRNA sequence
A_72_P443584	ATXN1	1,5239	0,0347	down	Sus scrofa ataxin 1 (ATXN1), mRNA

A_72_P151466		1,5239	0,0160	down	BX923970 Sus Scrofa library (scan) Sus scrofa cDNA clone scan0015.o.10 5prim, mRNA sequence
A_72_P368448	CFP	1,5232	0,0394	down	Sus scrofa complement factor properdin (CFP), mRNA
A_72_P407843	AMPD3	1,5230	0,0368	down	Sus scrofa adenosine monophosphate deaminase 3 (AMPD3), mRNA
A_72_P636073	CTSK	1,5215	0,0118	down	Sus scrofa cathepsin K (CTSK), mRNA
A_72_P673137	BCCIP	1,5188	0,0244	down	Sus scrofa BRCA2 and CDKN1A interacting protein (BCCIP), mRNA
A_72_P168901		1,5182	0,0475	down	1110540 MARC 4PIG Sus scrofa cDNA 3', mRNA sequence
A_72_P260252	SIT1	1,5180	0,0389	down	Sus scrofa signaling threshold regulating transmembrane adaptor 1 (SIT1), mRNA
A_72_P225962	APTX	1,5164	0,0292	down	Sus scrofa aprataxin (APTX), mRNA
A_72_P486262	ZIC1	1,5159	0,0190	down	Sus scrofa Zic family member 1 (ZIC1), mRNA
A_72_P010366	LOC100736836	1,5150	0,0283	down	Sus scrofa mixed lineage kinase domain-like protein-like (LOC100736836), mRNA
A_72_P516237	LOC106505672	1,5143	0,0041	down	PREDICTED: Sus scrofa interleukin-1 receptor-associated kinase-like 2 (LOC106505672), mRNA
A_72_P107786	TLN1	1,5142	0,0313	down	Sus scrofa talin 1 (TLN1), mRNA
A_72_P320963	LOC100738358	1,5140	0,0492	down	Sus scrofa V-set and immunoglobulin domain-containing protein 4-like (LOC100738358), mRNA
A_72_P478994	C7H14orf118	1,5114	0,0135	down	Sus scrofa uncharacterized protein C14orf118-like (C7H14orf118), mRNA
A_72_P443556	LRRC8C	1,5108	0,0256	down	Sus scrofa leucine rich repeat containing 8 family, member C (LRRC8C), mRNA
A_72_P381373	RPS6KA3	1,5076	0,0241	down	Sus scrofa ribosomal protein S6 kinase, 90kDa, polypeptide 3 (RPS6KA3), mRNA
A_72_P655258	CHSY1	1,5072	0,0210	down	Sus scrofa chondroitin sulfate synthase 1 (CHSY1), mRNA
A_72_P231642		1,5059	0,0130	down	AJ960631 KN404_2 Sus scrofa cDNA clone C0007759n14 5', mRNA sequence
A_72_P140936		1,5052	0,0060	down	rcje12_a8.y1 cje Sus scrofa cDNA 5', mRNA sequence
A_72_P600573	CA3	1,5047	0,0443	down	Sus scrofa carbonic anhydrase III, muscle specific (CA3), mRNA
A_72_P476416	LOC100154071	1,5022	0,0472	down	Sus scrofa uncharacterized LOC100154071 (LOC100154071), mRNA
A_72_P426394	MID1IP1	1,5016	0,0152	down	PREDICTED: Sus scrofa MID1 interacting protein 1 (MID1IP1), transcript variant X1, mRNA
A_72_P256402	LOC100624199	1,5014	0,0412	down	Sus scrofa solute carrier family 40 member 1-like (LOC100624199), mRNA
A_72_P387818		1,5010	0,0105	down	324651 MARC 1PIG Sus scrofa cDNA 5', mRNA sequence
A_72_P398118	LOC100515779	1,4970	0,0093	down	Sus scrofa dual specificity tyrosine-phosphorylation-regulated kinase 2-like (LOC100515779), mRNA
A_72_P306433		1,4970	0,0278	down	MI-P-CP1-nwn-c-11-0-UI.s1 MI-P-CP1 Sus scrofa cDNA clone MI-P-CP1-nwn-c-11-0-UI 3', mRNA sequence
A_72_P058806	IL17D	1,4964	0,0087	down	Sus scrofa interleukin 17D (IL17D), mRNA
A_72_P359548	TMX4	1,4948	0,0220	down	Sus scrofa thioredoxin-related transmembrane protein 4 (TMX4), mRNA
A_72_P359358		1,4936	0,0446	down	Sus scrofa mRNA, clone:SMG010050A07, expressed in submaxillary gland
A_72_P317438	LOC100624467	1,4935	0,0268	down	Sus scrofa zinc finger protein 347-like (LOC100624467), mRNA
A_72_P366538		1,4932	0,0171	down	899950 MARC 4PIG Sus scrofa cDNA 3', mRNA sequence
A_72_P082626		1,4926	0,0322	down	BX922545 Sus Scrofa library (scan) Sus scrofa cDNA clone scan0018.j.11 5prim, mRNA sequence
A_72_P491087	ZBTB2	1,4910	0,0436	down	Sus scrofa zinc finger and BTB domain containing 2 (ZBTB2), mRNA
A_72_P434019	LOC100737758	1,4901	0,0432	down	Sus scrofa protein transport protein Sec31A-like (LOC100737758), mRNA
A_72_P275124	LOC100738337	1,4899	0,0260	down	Sus scrofa myosin light chain kinase, smooth muscle-like (LOC100738337), partial mRNA
A_72_P023796		1,4889	0,0034	down	BW965580 full-length enriched swine cDNA library, adult ovary Sus scrofa cDNA clone OVR010067D06 5', mRNA sequence
A_72_P410548		1,4878	0,0494	down	susfleck_FC_N_5_E12 SUSFLECK Fat Cell Normalized Sus scrofa cDNA clone 5_E12, mRNA sequence
A_72_P453504	PPPDE2	1,4875	0,0345	down	Sus scrofa desumoylating isopeptidase 1 (PPPDE2), mRNA
A_72_P370453	FAM114A1	1,4867	0,0024	down	Sus scrofa family with sequence similarity 114, member A1 (FAM114A1), mRNA
A_72_P314243	ELOVL7	1,4843	0,0005	down	Sus scrofa ELOVL fatty acid elongase 7 (ELOVL7), mRNA
A_72_P585956	MTMR14	1,4828	0,0154	down	Sus scrofa myotubularin related protein 14 (MTMR14), transcript variant 3, mRNA
A_72_P226242		1,4819	0,0082	down	952597 MARC 4PIG Sus scrofa cDNA 3', mRNA sequence
A_72_P141986	MAP1A	1,4809	0,0192	down	Sus scrofa microtubule-associated protein 1A (MAP1A), mRNA
A_72_P681440		1,4789	0,0408	down	PDUts1073G07 Porcine testis cDNA library I Sus scrofa cDNA clone PDUts1073G07 5' similar to homologue to ref|NM_014477.2| Homo sapiens chromosome 20 open reading frame 10 (C20orf10), mRNA, mRNA sequence
A_72_P281869		1,4778	0,0198	down	Sus scrofa sex comb on midleg-like protein 4-like (LOC100738974), partial mRNA
A_72_P378713		1,4778	0,0145	down	Pig DNA sequence from clone CH242-106L21 on chromosome X, complete sequence
A_72_P017296	EIF4G1	1,4777	0,0481	down	Sus scrofa eukaryotic translation initiation factor 4 gamma, 1 (EIF4G1), mRNA
A_72_P232527	SELP	1,4776	0,0479	down	Sus scrofa selectin P (granule membrane protein 140kDa, antigen CD62) (SELP), mRNA
A_72_P109831	RRAD	1,4769	0,0401	down	Sus scrofa Ras-related associated with diabetes (RRAD), partial mRNA
A_72_P602644	CKM	1,4767	0,0455	down	Sus scrofa creatine kinase, muscle (CKM), mRNA
A_72_P455657	TXNRD1	1,4752	0,0105	down	Sus scrofa thioredoxin reductase 1 (TXNRD1), mRNA
A_72_P201852		1,4746	0,0279	down	BX669346 Sus Scrofa library (scac) Sus scrofa cDNA clone scac0029.f.20 3prim, mRNA sequence
A_72_P131261	RAPH1	1,4739	0,0496	down	Sus scrofa Ras association (RalGDS/AF-6) and pleckstrin homology domains 1 (RAPH1), mRNA
A_72_P186641	CHST3	1,4732	0,0003	down	Sus scrofa carbohydrate (chondroitin 6) sulfotransferase 3 (CHST3), mRNA
A_72_P125276	REEP3	1,4723	0,0017	down	Sus scrofa receptor accessory protein 3 (REEP3), mRNA
A_72_P299444	SNRK	1,4721	0,0473	down	Sus scrofa SNF related kinase (SNRK), mRNA
A_72_P206762		1,4719	0,0353	down	
A_72_P392563		1,4713	0,0215	down	BX914598 Sus Scrofa library (scan) Sus scrofa cDNA clone scan0027.d.14 5prim, mRNA sequence
A_72_P389453	NFATC3	1,4712	0,0018	down	Sus scrofa nuclear factor of activated T-cells, cytoplasmic, calcineurin-dependent 3 (NFATC3), partial mRNA
A_72_P217742	LOC100521749	1,4707	0,0010	down	PREDICTED: Sus scrofa breakpoint cluster region protein (LOC100521749), mRNA
A_72_P128021		1,4701	0,0038	down	1106573 MARC 4PIG Sus scrofa cDNA 5', mRNA sequence
A_72_P117351		1,4697	0,0210	down	1554020 MARC 3PIG Sus scrofa cDNA 3', mRNA sequence
A_72_P124116		1,4694	0,0061	down	AJ649612 CSEQRAN19 Sus scrofa cDNA clone C0003272_P12, mRNA sequence
A_72_P119551	KDM5B	1,4691	0,0034	down	Sus scrofa lysine (K)-specific demethylase 5B (KDM5B), mRNA
A_72_P327348	LOC106510427	1,4683	0,0158	down	PREDICTED: Sus scrofa uncharacterized LOC106510427 (LOC106510427), ncRNA
A_72_P412888	CEBPA	1,4661	0,0304	down	Sus scrofa CCAAT/enhancer binding protein (C/EBP), alpha (CEBPA), mRNA

A_72_P178056	TMEM57	1,4658	0,0129	down	Sus scrofa transmembrane protein 57 (TMEM57), mRNA
A_72_P186881	LOC100520636	1,4652	0,0186	down	Sus scrofa very long-chain specific acyl-CoA dehydrogenase, mitochondrial-like (LOC100520636), mRNA
A_72_P388418	CABIN1	1,4650	0,0263	down	Sus scrofa calcineurin binding protein 1 (CABIN1), mRNA
A_72_P239292		1,4650	0,0286	down	262953 MARC 2PIG Sus scrofa cDNA 5', mRNA sequence
A_72_P555835	CITED2	1,4646	0,0440	down	Sus scrofa Cbp/p300-interacting transactivator, with Glu/Asp-rich carboxy-terminal domain, 2 (CITED2), mRNA
A_72_P013036	FANCD2	1,4644	0,0472	down	Sus scrofa Fanconi anemia, complementation group D2 (FANCD2), mRNA
A_72_P225872	LOC102164714	1,4629	0,0097	down	Sus scrofa uncharacterized LOC102164714 (LOC102164714), misc_RNA
A_72_P394148	LOC102166027	1,4616	0,0096	down	Sus scrofa TBC1 domain family member 10B-like (LOC102166027), mRNA
A_72_P177961	RENBP	1,4611	0,0114	down	Sus scrofa renin binding protein (RENBP), mRNA
A_72_P385113	SLC16A8	1,4611	0,0026	down	Sus scrofa solute carrier family 16 (monocarboxylate transporter), member 8 (SLC16A8), mRNA
A_72_P101771	FTSJ3	1,4611	0,0399	down	Sus scrofa FtsJ homolog 3 (E. coli) (FTSJ3), mRNA
A_72_P493740	TLE3	1,4602	0,0244	down	Sus scrofa transducin-like enhancer of split 3 (E(sp1) homolog, Drosophila) (TLE3), partial mRNA
A_72_P444471	ME1	1,4589	0,0281	down	Sus scrofa malic enzyme 1, NADP(+)-dependent, cytosolic (ME1), mRNA
A_72_P442534	PIP4K2A	1,4575	0,0373	down	Sus scrofa phosphatidylinositol-5-phosphate 4-kinase, type II, alpha (PIP4K2A), mRNA
A_72_P318233		1,4575	0,0471	down	364283 MARC 2PIG Sus scrofa cDNA 5', mRNA sequence
A_72_P214892	ZNF236	1,4570	0,0144	down	Sus scrofa zinc finger protein 236 (ZNF236), partial mRNA
A_72_P672137	CEBPB	1,4567	0,0292	down	PREDICTED: Sus scrofa CCAAT/enhancer binding protein (C/EBP), beta (CEBPB), mRNA
A_72_P245572	HBZ	1,4551	0,0270	down	Sus scofa hemoglobin, zeta (HBZ), mRNA
A_72_P391818		1,4517	0,0072	down	Sus scrofa mRNA, clone: PST010025D12, expressed in prostate
A_72_P306084	MYLK	1,4501	0,0315	down	Sus scrofa myosin light chain kinase (MYLK), mRNA
A_72_P062126		1,4484	0,0086	down	rcki24_j23.y1 cki Sus scrofa cDNA 5', mRNA sequence
A_72_P369043	SMAD7	1,4482	0,0419	down	Sus scrofa SMAD family member 7 (SMAD7), mRNA
A_72_P046786		1,4474	0,0373	down	AJ958093 KN404_2 Sus scrofa cDNA clone C0007754h01 5', mRNA sequence
A_72_P642520	CEBPB	1,4472	0,0399	down	Sus scrofa CCAAT/enhancer-binding protein beta (C/EBP beta) mRNA, 3' UTR
A_72_P405563	SH3D19	1,4470	0,0198	down	Sus scrofa SH3 domain containing 19 (SH3D19), mRNA
A_72_P144826	GPX2	1,4448	0,0411	down	Sus scrofa glutathione peroxidase 2 (gastrointestinal) (GPX2), mRNA
A_72_P706296	DCLK3	1,4433	0,0269	down	Sus scrofa doublecortin-like kinase 3 (DCLK3), mRNA
A_72_P744064		1,4429	0,0144	down	
A_72_P264607		1,4427	0,0364	down	Sus scrofa mRNA, clone: OVRM10154F10, expressed in ovary
A_72_P030756	AZIN1	1,4426	0,0477	down	Sus scrofa antizyme inhibitor 1 (AZIN1), mRNA
A_72_P023201	LOC100155528	1,4395	0,0077	down	Sus scrofa uncharacterized LOC100155528 (LOC100155528), mRNA
A_72_P599748	CXCR4	1,4393	0,0363	down	Sus scrofa mRNA, clone: AMP010061D05, expressed in alveolar macrophage
A_72_P322718	RARB	1,4393	0,0485	down	PREDICTED: Sus scrofa retinoic acid receptor, beta (RARB), transcript variant X3, mRNA
A_72_P669017	RPIA	1,4385	0,0080	down	Sus scrofa ribose 5-phosphate isomerase A (RPIA), mRNA
A_72_P773975		1,4378	0,0498	down	HX239372 full-length enriched swine cDNA library, adult spleen Sus scrofa cDNA clone SPLT10002C08, mRNA sequence
A_72_P184296		1,4367	0,0315	down	BW966806 full-length enriched swine cDNA library, adult ovary Sus scrofa cDNA clone OVR010082B12 5', mRNA sequence
A_72_P308648	MSN	1,4350	0,0205	down	Sus scrofa moesin (MSN), mRNA
A_72_P235462	KAT2B	1,4332	0,0267	down	Sus scrofa K(lysine) acetyltransferase 2B (KAT2B), mRNA
A_72_P606653	CRELD1	1,4329	0,0424	down	Sus scrofa cysteine-rich with EGF-like domains 1 (CRELD1), mRNA
A_72_P425104		1,4327	0,0037	down	UMC-p4civv1-021-e02 4-Cell-Embryo-(invivo) p4civv Sus scrofa cDNA 3', mRNA sequence
A_72_P359033	MESDC1	1,4327	0,0095	down	Sus scrofa mesoderm development candidate 1 (MESDC1), mRNA
A_72_P443759	GLTSCR1L	1,4316	0,0015	down	PREDICTED: Sus scrofa GLTSCR1-like (GLTSCR1L), transcript variant X1, mRNA
A_72_P323148		1,4293	0,0299	down	Sus scrofa mRNA, clone:OVRM10055D01, expressed in ovary
A_72_P544532	IGF2	1,4281	0,0386	down	Sus scrofa insulin-like growth factor 2 precursor transcript variant 8 (IGF2) mRNA
A_72_P222532	SACM1L	1,4272	0,0244	down	PREDICTED: Sus scrofa SAC1 suppressor of actin mutations 1-like (yeast) (SACM1L), transcript variant X1, mRNA
A_72_P106356	C4H1orf189	1,4270	0,0119	down	Sus scrofa chromosome 4 open reading frame, human C1orf189 (C4H1orf189), mRNA
A_72_P023541	SLC12A2	1,4265	0,0228	down	Sus scrofa solute carrier family 12 (sodium/potassium/chloride transporter), member 2 (SLC12A2), mRNA
A_72_P179566	ARHGAP28	1,4259	0,0336	down	Sus scrofa Rho GTPase activating protein 28 (ARHGAP28), mRNA
A_72_P303349	LOC780419	1,4253	0,0203	down	Sus scrofa UB2D1 (LOC780419), mRNA
A_72_P259537	NUAK2	1,4252	0,0282	down	Sus scrofa NUAK family, SNF1-like kinase, 2 (NUAK2), mRNA
A_72_P487898	STX2	1,4239	0,0264	down	Sus scrofa syntaxin 2 (STX2), mRNA
A_72_P383133	ABCF1	1,4228	0,0166	down	Sus scrofa ATP-binding cassette, sub-family F (GCN20), member 1 (ABCF1), mRNA
A_72_P245047	IFNAR1, IFNGR2, TMEM50B	1,4227	0,0265	down	Sus scrofa IFNAR1 gene, IFNGR2 gene and TMEM50B gene
A_72_P140041	GTF3C1	1,4226	0,0113	down	Sus scrofa general transcription factor IIIC, polypeptide 1, alpha 220kDa (GTF3C1), mRNA
A_72_P361938		1,4220	0,0150	down	Sus scrofa uncharacterized LOC100517767 (LOC100517767), misc_RNA
A_72_P077381	ITGAL	1,4215	0,0291	down	Sus scrofa integrin, alpha L (antigen CD11A (p180), lymphocyte function-associated antigen 1; alpha polypeptide) (ITGAL), mRNA
A_72_P299809		1,4201	0,0293	down	326271 MARC 1PIG Sus scrofa cDNA 5', mRNA sequence
A_72_P204112	DNAJB1	1,4179	0,0301	down	Sus scrofa dnaJ homolog subfamily B member 1-like (LOC100627777), mRNA
A_72_P076926		1,4176	0,0495	down	239016 MARC 2PIG Sus scrofa cDNA 5', mRNA sequence
A_72_P365638	INPP5B	1,4175	0,0248	down	Sus scrofa inositol polyphosphate-5-phosphatase, 75kDa (INPP5B), mRNA
A_72_P021606	TRIM4	1,4158	0,0149	down	Sus scrofa tripartite motif containing 4 (TRIM4), mRNA
A_72_P511680		1,4154	0,0466	down	not found
A_72_P383898	APPL1	1,4123	0,0040	down	Sus scrofa adaptor protein, phosphotyrosine interaction, PH domain and leucine zipper containing 1 (APPL1), mRNA
A_72_P016721	MYOF	1,4117	0,0270	down	Sus scrofa myoferlin (MYOF), mRNA
A_72_P188801	CHD8	1,4115	0,0268	down	Sus scrofa chromodomain helicase DNA binding protein 8 (CHD8), partial mRNA
A_72_P490818	CALY	1,4108	0,0259	down	Sus scrofa calcyon neuron-specific vesicular protein (CALY), mRNA
A_72_P411143	PHF20L1	1,4096	0,0175	down	Sus scrofa PHD finger protein 20-like 1 (PHF20L1), mRNA
A_72_P450017	PABPC1L	1,4083	0,0045	down	Sus scrofa poly(A) binding protein, cytoplasmic 1-like (PABPC1L), mRNA
A_72_P077316	BDKRB1	1,4079	0,0398	down	Sus scrofa bradykinin receptor B1 (BDKRB1), mRNA

A_72_P431514	ARHGAP10	1,4077	0,0271	down	PREDICTED: Sus scrofa Rho GTPase activating protein 10 (ARHGAP10), mRNA
A_72_P631250	ETS1	1,4074	0,0200	down	Sus scrofa v-ets erythroblastosis virus E26 oncogene homolog 1 (avian) (ETS1), mRNA
A_72_P646436	PGA	1,4070	0,0384	down	Sus scrofa pepsinogen A (PGA), mRNA
A_72_P005561	TP63	1,4064	0,0140	down	Sus scrofa tumor protein p63 (TP63), mRNA
A_72_P391903	PIGV	1,4046	0,0185	down	PREDICTED: Sus scrofa phosphatidylinositol glycan anchor biosynthesis, class V (PIGV), transcript variant X3, mRNA
A_72_P166706	GRB7	1,4038	0,0126	down	Sus scrofa growth factor receptor-bound protein 7 (GRB7), mRNA
A_72_P189831	EP300	1,4034	0,0220	down	Sus scrofa E1A binding protein p300 (EP300), mRNA
A_72_P186391	CD247	1,4026	0,0068	down	Sus scrofa CD247 molecule (CD247), mRNA
A_72_P540123	LDB1	1,4015	0,0435	down	Sus scrofa LIM domain binding 1 (LDB1), mRNA
A_72_P651922	LHB	1,4011	0,0061	down	Sus scrofa luteinizing hormone beta polypeptide (LHB), mRNA
A_72_P724643	TPM1	1,4000	0,0271	down	Sus scrofa tropomyosin 1 (alpha) (TPM1), mRNA
A_72_P642425		1,3994	0,0488	down	rcki09_g11.y1 cki Sus scrofa cDNA 5', mRNA sequence
A_72_P317973	KMT2A	1,3977	0,0063	down	Sus scrofa lysine (K)-specific methyltransferase 2A (KMT2A), mRNA
A_72_P732383	CXCL2	1,3973	0,0299	down	Sus scrofa chemokine (C-X-C motif) ligand 2 (CXCL2), mRNA
A_72_P753775	SAV1	1,3970	0,0175	down	Sus scrofa salvador homolog 1 (Drosophila) (SAV1), mRNA
A_72_P532951	FAM21C	1,3969	0,0314	down	Sus scrofa family with sequence similarity 21, member C (FAM21C), mRNA
A_72_P222767	TRIP12	1,3966	0,0123	down	Sus scrofa thyroid hormone receptor interactor 12 (TRIP12), mRNA
A_72_P058991	KLF13	1,3964	0,0183	down	Sus scrofa Kruppel-like factor 13 (KLF13) mRNA, complete cds
A_72_P246162	WWP1	1,3959	0,0458	down	Sus scrofa WW domain containing E3 ubiquitin protein ligase 1 (WWP1), mRNA
A_72_P264392	LOC102161272	1,3957	0,0216	down	Sus scrofa uncharacterized LOC102161272 (LOC102161272), misc_RNA
A_72_P518348	LOC100155473	1,3945	0,0131	down	Sus scrofa dedicator of cytokinesis protein 1-like (LOC100155473), mRNA
A_72_P329198		1,3943	0,0000	down	rlin19_c15.y1 lin Sus scrofa cDNA 5', mRNA sequence
A_72_P555381	MIC-2	1,3943	0,0222	down	Sus scrofa MHC class I related antigen 2 (MIC-2), mRNA
A_72_P200087	LOC100153182	1,3920	0,0258	down	Sus scrofa EF-hand calcium-binding domain-containing protein 1-like (LOC100153182), mRNA
A_72_P475675	FAM102B	1,3913	0,0234	down	PREDICTED: Sus scrofa family with sequence similarity 102, member B (FAM102B), mRNA
A_72_P084881	ELANE	1,3913	0,0480	down	Sus scrofa elastase, neutrophil expressed (ELANE), mRNA
A_72_P003386	PYGL	1,3872	0,0049	down	Sus scrofa phosphorylase, glycogen, liver (PYGL), mRNA
A_72_P117276		1,3868	0,0167	down	1570154 MARC 3PIG Sus scrofa cDNA 3', mRNA sequence
A_72_P441514	CSF3	1,3865	0,0263	down	Sus scrofa colony stimulating factor 3 (granulocyte) (CSF3), mRNA
A_72_P584851	LY96	1,3842	0,0291	down	Sus scrofa lymphocyte antigen 96 (LY96), mRNA
A_72_P406183	CBLB	1,3841	0,0235	down	Sus scrofa Cbl proto-oncogene B, E3 ubiquitin protein ligase (CBLB), mRNA
A_72_P005506	IKZF4	1,3834	0,0342	down	Sus scrofa IKAROS family zinc finger 4 (Eos) (IKZF4), mRNA
A_72_P271999	MYCT1	1,3826	0,0393	down	PREDICTED: Sus scrofa myc target 1 (MYCT1), mRNA
A_72_P243407	GOLPH3L	1,3810	0,0448	down	Sus scrofa golgi phosphoprotein 3-like (GOLPH3L), mRNA
A_72_P660698	RAB11FIP2	1,3810	0,0046	down	Sus scrofa RAB11 family interacting protein 2 (class I) (RAB11FIP2), mRNA
A_72_P476273	LOC100153329	1,3803	0,0427	down	Sus scrofa histone H2A type 1-A-like (LOC100153329), transcript variant X1, mRNA
A_72_P041636	ZCCHC2	1,3799	0,0094	down	PREDICTED: Sus scrofa zinc finger, CCHC domain containing 2 (ZCCHC2), partial mRNA
A_72_P068256		1,3797	0,0359	down	recc2825c_o18.y1 ecc Sus scrofa cDNA 5', mRNA sequence
A_72_P609999	LY96	1,3775	0,0406	down	Sus scrofa lymphocyte antigen 96 (LY96), mRNA
A_72_P474341	DDR2	1,3775	0,0363	down	Sus scrofa discoidin domain receptor tyrosine kinase 2 (DDR2), mRNA
A_72_P443520	LOC100621389	1,3751	0,0169	down	Sus scrofa histone H4-like (LOC100621389), mRNA
A_72_P572519	CEP44	1,3741	0,0239	down	Sus scrofa centrosomal protein 44kDa (CEP44), mRNA
A_72_P098116	DNAAF1	1,3733	0,0362	down	Sus scrofa dynein, axonemal, assembly factor 1 (DNAAF1), mRNA
A_72_P078126		1,3730	0,0029	down	Sus scrofa mRNA, clone:PBL010030D02, expressed in peripheral blood mononuclear cells
A_72_P709589	APP	1,3725	0,0465	down	Sus scrofa amyloid beta (A4) precursor protein (APP), mRNA
A_72_P406648		1,3721	0,0331	down	rcst09_b2E11.y1 cst Sus scrofa cDNA 5', mRNA sequence
A_72_P088596	PLIN2	1,3720	0,0166	down	Sus scrofa perilipin 2 (PLIN2), mRNA
A_72_P108371	HSD3B1	1,3706	0,0415	down	Sus scrofa hydroxy-delta-5-steroid dehydrogenase, 3 beta- and steroid delta-isomerase 1 (HSD3B1), mRNA
A_72_P087296	MDFIC	1,3706	0,0398	down	Sus scrofa MyoD family inhibitor domain containing (MDFIC), mRNA
A_72_P153441		1,3691	0,0075	down	reye1613b_e22r1.y1 eye Sus scrofa cDNA 5', mRNA sequence
A_72_P405313	CYLD	1,3678	0,0052	down	PREDICTED: Sus scrofa cylindromatosis (turban tumor syndrome) (CYLD), mRNA
A_72_P165221	CLN3	1,3675	0,0216	down	Sus scrofa ceroid-lipofuscinosis, neuronal 3 (CLN3), mRNA
A_72_P264357		1,3672	0,0267	down	BX919483 Sus Scrofa library (scan) Sus scrofa cDNA clone scan0004.j.09 5prim, mRNA sequence
A_72_P453774	PRDM4	1,3668	0,0150	down	Sus scrofa PR domain containing 4 (PRDM4), mRNA
A_72_P709040		1,3666	0,0358	down	Sus scrofa chromosome 6 open reading frame 106 ortholog (C7H6orf106), mRNA
A_72_P261657	CBL	1,3657	0,0337	down	Sus scrofa Cbl proto-oncogene, E3 ubiquitin protein ligase (CBL), mRNA
A_72_P313608	SYN1	1,3652	0,0406	down	Sus scrofa synapsin I (SYN1), mRNA
A_72_P035886	APOA1	1,3652	0,0438	down	Sus scrofa apolipoprotein A-I (APOA1), mRNA
A_72_P474927		1,3649	0,0245	down	
A_72_P405233	TM7SF2	1,3648	0,0401	down	Sus scrofa transmembrane 7 superfamily member 2 (TM7SF2), mRNA
A_72_P372203	PCIF1	1,3638	0,0310	down	Sus scrofa PDX1 C-terminal inhibiting factor 1 (PCIF1), mRNA
A_72_P019266		1,3635	0,0342	down	
A_72_P198192		1,3634	0,0486	down	rcst09_b2E11.y1 cst Sus scrofa cDNA 5', mRNA sequence
A_72_P323168		1,3632	0,0134	down	Sus scrofa mRNA, clone:OVRM10064C11, expressed in ovary
A_72_P402618		1,3617	0,0294	down	rese25c_k5.y1 ese Sus scrofa cDNA 5', mRNA sequence
A_72_P628192	INPP4A	1,3602	0,0220	down	Sus scrofa inositol polyphosphate-4-phosphatase, type I, 107kDa (INPP4A), mRNA
A_72_P077366	DTNBP1	1,3598	0,0181	down	Sus scrofa dystrobrevin binding protein 1 (DTNBP1), mRNA
A_72_P006376	MRAP2	1,3597	0,0148	down	Sus scrofa melanocortin 2 receptor accessory protein 2 (MRAP2), mRNA
A_72_P226142		1,3584	0,0440	down	1000732 MARC 4PIG Sus scrofa cDNA 3', mRNA sequence
A_72_P233662		1,3581	0,0092	down	Sus scrofa mRNA, clone: MLTL10071B08, expressed in longissimus muscle
A_72_P374898		1,3572	0,0316	down	BX922043 Sus Scrofa library (scan) Sus scrofa cDNA clone scan0023.e.07 5prim, mRNA sequence
A_72_P443877	LOC100620346	1,3567	0,0442	down	Sus scrofa uncharacterized LOC100620346 (LOC100620346), misc_RNA
A_72_P065171	ZBED5	1,3566	0,0117	down	Sus scrofa zinc finger, BED-type containing 5 (ZBED5), mRNA
A_72_P676396	ARID5B	1,3559	0,0131	down	Sus scrofa AT rich interactive domain 5B (MRF1-like) (ARID5B), mRNA
A_72_P015121		1,3529	0,0183	down	1117432 MARC 4PIG Sus scrofa cDNA 3', mRNA sequence
A_72_P291569	LOC100513756	1,3514	0,0197	down	Sus scrofa TPR and ankyrin repeat-containing protein 1-like (LOC100513756), mRNA

A_72_P432954	CDKL5	1,3510	0,0236	down	Sus scrofa cyclin-dependent kinase-like 5 (CDKL5), mRNA
A_72_P479357	CCNK	1,3502	0,0349	down	Sus scrofa cyclin K (CCNK), mRNA
A_72_P407903		1,3500	0,0376	down	Sus scrofa mRNA, clone: PST010052E12, expressed in prostate
A_72_P088186	OPRL1	1,3477	0,0010	down	Sus scrofa opiate receptor-like 1 (OPRL1), mRNA
A_72_P000256		1,3476	0,0020	down	HX204550 full-length enriched swine cDNA library, adult brain (frontal lobe) Sus scrofa cDNA clone BFLT10135E03, mRNA sequence
A_72_P444413	SOD2	1,3473	0,0232	down	Sus scrofa superoxide dismutase 2, mitochondrial (SOD2), transcript variant X1, mRNA
A_72_P443854	ZFP36L1	1,3467	0,0466	down	Sus scrofa ZFP36 ring finger protein-like 1 (ZFP36L1), mRNA
A_72_P232372	ZFAND5	1,3460	0,0315	down	Sus scrofa zinc finger, AN1-type domain 5 (ZFAND5), mRNA
A_72_P555755	RAD23B	1,3441	0,0217	down	Sus scrofa RAD23 homolog B (S. cerevisiae) (RAD23B), mRNA
A_72_P708845	CDC42SE1	1,3408	0,0154	down	Sus scrofa CDC42 small effector 1 (CDC42SE1), mRNA
A_72_P362778	HNRNPK	1,3404	0,0185	down	Sus scrofa heterogeneous nuclear ribonucleoprotein K (HNRNPK), mRNA
A_72_P703926	LOC100513756	1,3389	0,0383	down	PREDICTED: Sus scrofa TPR and ankyrin repeat-containing protein 1-like (LOC100513756), mRNA
A_72_P443643	LOC100158121	1,3382	0,0400	down	Sus scrofa histone H2A type 1-like (LOC100158121), mRNA
A_72_P475146	PLEKHO1	1,3381	0,0173	down	PREDICTED: Sus scrofa pleckstrin homology domain containing, family O member 1 (PLEKHO1), transcript variant X1, mRNA
A_72_P383743	BRD2	1,3377	0,0301	down	Sus scrofa bromodomain containing 2 (BRD2), mRNA
A_72_P688621	SIRPA	1,3374	0,0272	down	Sus scrofa signal-regulatory protein alpha (SIRPA), mRNA
A_72_P553926		1,3356	0,0091	down	
A_72_P230187	KANSL1	1,3355	0,0101	down	Sus scrofa KAT8 regulatory NSL complex subunit 1 (KANSL1), mRNA
A_72_P474357	UHMK1	1,3344	0,0071	down	PREDICTED: Sus scrofa U2AF homology motif (UHM) kinase 1 (UHMK1), mRNA
A_72_P441858	IL10RB	1,3340	0,0230	down	Sus scrofa interleukin 10 receptor, beta (IL10RB), mRNA
A_72_P437009	CSNK2A2	1,3338	0,0179	down	Sus scrofa casein kinase 2, alpha prime polypeptide (CSNK2A2), mRNA
A_72_P477646	ABHD2	1,3336	0,0221	down	Sus scrofa abhydrolase domain containing 2 (ABHD2), mRNA
A_72_P641264		1,3335	0,0195	down	
A_72_P273434	LOC100737341	1,3325	0,0323	down	Sus scrofa synapse differentiation-inducing gene protein 1-like (LOC100737341), partial mRNA
A_72_P338323		1,3325	0,0080	down	1556591 MARC 3PIG Sus scrofa cDNA 3', mRNA sequence
A_72_P106136	HIPK3	1,3322	0,0283	down	PREDICTED: Sus scrofa homeodomain interacting protein kinase 3 (HIPK3), transcript variant X4, mRNA
A_72_P170956		1,3319	0,0473	down	230281 MARC 2PIG Sus scrofa cDNA 5', mRNA sequence
A_72_P053766	LOC106506662	1,3317	0,0001	down	PREDICTED: Sus scrofa cilia- and flagella-associated protein 53-like (LOC106506662), mRNA
A_72_P084236		1,3311	0,0269	down	Sus scrofa clone KVL2011 microsatellite sequence
A_72_P517672	SLA-8	1,3310	0,0448	down	Sus scrofa MHC class I antigen 8 (SLA-8), mRNA
A_72_P286854	NCEH1	1,3296	0,0321	down	Sus scrofa neutral cholesterol ester hydrolase 1 (NCEH1), mRNA
A_72_P692777		1,3291	0,0156	down	Sus scrofa mRNA, clone:OVRM10096D11, expressed in ovary
A_72_P279714	CCDC85A	1,3290	0,0236	down	Sus scrofa coiled-coil domain containing 85A (CCDC85A), mRNA
A_72_P193702	LOC106504487	1,3287	0,0482	down	PREDICTED: Sus scrofa uncharacterized LOC106504487 (LOC106504487), ncRNA
A_72_P633177	TMCO1	1,3281	0,0281	down	Sus scrofa transmembrane and coiled-coil domains 1 (TMCO1), mRNA
A_72_P093506		1,3277	0,0052	down	AJ648384 CSEQRAN19 Sus scrofa cDNA clone C0003264_I04, mRNA sequence
A_72_P223937	ICAM-1	1,3274	0,0288	down	Sus scrofa intercellular adhesion molecule-1 (ICAM-1), mRNA
A_72_P584626	PLN	1,3267	0,0473	down	Sus scrofa phospholamban (PLN), mRNA
A_72_P058776	LOC100620661	1,3260	0,0005	down	PREDICTED: Sus scrofa ubiquitin carboxyl-terminal hydrolase 10-like (LOC100620661), mRNA
A_72_P176331		1,3257	0,0231	down	Sus scrofa mRNA for hypothetical protein (5'; clone 2C4)
A_72_P185136	MAT2B	1,3254	0,0269	down	Sus scrofa methionine adenosyltransferase II, beta (MAT2B), mRNA
A_72_P168911	PAN3	1,3253	0,0389	down	Sus scrofa PAN3 poly(A) specific ribonuclease subunit homolog (S. cerevisiae) (PAN3), mRNA
A_72_P452702	FAM124A	1,3236	0,0212	down	Sus scrofa family with sequence similarity 124A (FAM124A), mRNA
A_72_P453839	CCNT1	1,3234	0,0478	down	PREDICTED: Sus scrofa cyclin T1 (CCNT1), transcript variant X1, mRNA
A_72_P169991	LOC102158880	1,3229	0,0252	down	Sus scrofa uncharacterized LOC102158880 (LOC102158880), misc_RNA
A_72_P530528	TACC2	1,3224	0,0020	down	Sus scrofa transforming, acidic coiled-coil containing protein 2 (TACC2), mRNA
A_72_P235247	LOC102163992	1,3223	0,0188	down	Sus scrofa uncharacterized LOC102163992 (LOC102163992), misc_RNA
A_72_P247297	DAPK1	1,3212	0,0100	down	Sus scrofa death-associated protein kinase 1 (DAPK1), mRNA
A_72_P431954	RBMS2	1,3209	0,0033	down	Sus scrofa RNA binding motif, single stranded interacting protein 2 (RBMS2), partial mRNA
A_72_P210287	ST3GAL1	1,3207	0,0216	down	Sus scrofa ST3 beta-galactoside alpha-2,3-sialyltransferase 1 (ST3GAL1), mRNA
A_72_P540858	DCAF8	1,3201	0,0474	down	Sus scrofa DDB1 and CUL4 associated factor 8 (DCAF8), mRNA
A_72_P202212		1,3188	0,0031	down	UNL-P-FN-ct-g-02-0-UNL.s2 UNL-P-FN Sus scrofa cDNA clone UNL-P-FN-ct-g-02-0-UNL 3', mRNA sequence
A_72_P429584	IQSEC2	1,3185	0,0446	down	Sus scrofa IQ motif and Sec7 domain 2 (IQSEC2), mRNA
A_72_P299589	DNAJB12	1,3183	0,0497	down	Sus scrofa DnaJ (Hsp40) homolog, subfamily B, member 12 (DNAJB12), mRNA
A_72_P476411	LOC100157763	1,3182	0,0137	down	Sus scrofa histone H2A type 1-like (LOC100157763), mRNA
A_72_P055626		1,3182	0,0315	down	AJ947164 KN404_1 Sus scrofa cDNA clone C0007745I10 3', mRNA sequence
A_72_P156796	ZNF256	1,3181	0,0245	down	Sus scrofa zinc finger protein 256 (ZNF256), mRNA
A_72_P301704	CPEB4	1,3181	0,0191	down	Sus scrofa cytoplasmic polyadenylation element binding protein 4 (CPEB4), mRNA
A_72_P594119	GCLM	1,3175	0,0458	down	Sus scrofa glutamate-cysteine ligase, modifier subunit (GCLM), mRNA
A_72_P033766		1,3175	0,0149	down	936656 MARC 4PIG Sus scrofa cDNA 3', mRNA sequence
A_72_P372868		1,3172	0,0181	down	856260 MARC 4PIG Sus scrofa cDNA 3', mRNA sequence
A_72_P413618	INSR	1,3167	0,0033	down	Sus scrofa insulin receptor (INSR), partial mRNA
A_72_P247177	SLCO2A1	1,3162	0,0263	down	Sus scrofa solute carrier organic anion transporter family, member 2A1 (SLCO2A1), mRNA
A_72_P382313	ARPC5	1,3160	0,0156	down	Sus scrofa actin related protein 2/3 complex, subunit 5, 16kDa (ARPC5), mRNA
A_72_P417964		1,3157	0,0265	down	Sus scrofa mRNA, clone:OVRM10137D04, expressed in ovary
A_72_P367038	TBL1XR1	1,3148	0,0083	down	Sus scrofa transducin (beta)-like 1 X-linked receptor 1 (TBL1XR1), mRNA
A_72_P117191	RAB14	1,3144	0,0184	down	Sus scrofa RAB14, member RAS oncogene family (RAB14), mRNA
A_72_P303424	FBXO9	1,3134	0,0468	down	Sus scrofa F-box protein 9 (FBXO9), mRNA
A_72_P014366		1,3133	0,0115	down	Sus scrofa mRNA, clone: OVRM10027E08, expressed in ovary
A_72_P034171		1,3126	0,0146	down	Sus scrofa mRNA, clone:THY010038E04, expressed in thymus
A_72_P510691	LOC106504487	1,3120	0,0146	down	PREDICTED: Sus scrofa uncharacterized LOC106504487 (LOC106504487), ncRNA
A_72_P188526	YLPM1	1,3110	0,0319	down	Sus scrofa YLP motif containing 1 (YLPM1), misc_RNA
A_72_P606928	TACC2	1,3101	0,0012	down	Sus scrofa transforming, acidic coiled-coil containing protein 2 (TACC2), mRNA

A_72_P316323	SLC27A2	1,3099	0,0324	down	Sus scrofa solute carrier family 27 (fatty acid transporter), member 2 (SLC27A2), mRNA
A_72_P070256	PEA15	1,3099	0,0483	down	Sus scrofa phosphoprotein enriched in astrocytes 15 (PEA15), mRNA
A_72_P313423	RAB13	1,3098	0,0427	down	Sus scrofa RAB13, member RAS oncogene family (RAB13), mRNA
A_72_P047556	PKN2	1,3091	0,0290	down	Sus scrofa protein kinase N2 (PKN2), mRNA
A_72_P035891	NR3C1	1,3090	0,0110	down	Sus scrofa nuclear receptor subfamily 3, group C, member 1 (glucocorticoid receptor) (NR3C1), mRNA
A_72_P577099	LGALS8	1,3087	0,0180	down	Sus scrofa lectin, galactoside-binding, soluble, 8 (LGALS8), mRNA
A_72_P281539		1,3080	0,0143	down	rese25c_k5.y1 ese Sus scrofa cDNA 5', mRNA sequence
A_72_P251407		1,3076	0,0221	down	BX670500 Sus Scrofa library (scac) Sus scrofa cDNA clone scac0032.f.19 5prim, mRNA sequence
A_72_P474362	C4H1orf111	1,3071	0,0423	down	Sus scrofa chromosome 4 open reading frame, human C1orf111 (C4H1orf111), mRNA
A_72_P077531	KLK1	1,3069	0,0091	down	Sus scrofa kallikrein 1 (KLK1), mRNA
A_72_P131871	CLIC4	1,3067	0,0416	down	PREDICTED: Sus scrofa chloride intracellular channel 4 (CLIC4), mRNA
A_72_P416543	ITGB7	1,3065	0,0033	down	Sus scrofa integrin, beta 7 (ITGB7), mRNA
A_72_P443294	GSR	1,3064	0,0241	down	Sus scrofa glutathione reductase (GSR), mRNA
A_72_P073381	SERPINB1	1,3053	0,0139	down	Sus scrofa serpin peptidase inhibitor, clade B (ovalbumin), member 1 (SERPINB1), mRNA
A_72_P302874		1,3044	0,0246	down	Sus scrofa mRNA for swine workshop cluster 3 antigen precursor (swc3 gene)
A_72_P230337	TNIP1	1,3037	0,0379	down	Sus scrofa TNFAIP3 interacting protein 1 (TNIP1), mRNA
A_72_P127081		1,3032	0,0352	down	DB820117 full-length enriched swine cDNA library, adult uterus Sus scrofa cDNA clone UTR010016D01 3', mRNA sequence
A_72_P182426	TBC1D16	1,3028	0,0195	down	Sus scrofa TBC1 domain family, member 16 (TBC1D16), mRNA
A_72_P296064	MMAB	1,3024	0,0483	down	Sus scrofa methylmalonic aciduria (cobalamin deficiency) cblB type (MMAB), mRNA
A_72_P302944	PPP1CC	1,3018	0,0385	down	Sus scrofa protein phosphatase 1, catalytic subunit, gamma isozyme (PPP1CC), mRNA
A_72_P033181	CAMSAP2	1,3010	0,0475	down	Sus scrofa calmodulin regulated spectrin-associated protein family, member 2 (CAMSAP2), mRNA
A_72_P232462	PLDN	1,3009	0,0431	down	Sus scrofa biogenesis of lysosomal organelles complex-1, subunit 6, pallidin (PLDN), mRNA
A_72_P555510	CWF19L1	1,3007	0,0483	down	Sus scrofa CWF19-like 1, cell cycle control (S. pombe) (CWF19L1), mRNA
A_72_P529595		1,3004	0,0280	down	Sus scrofa mRNA, clone: BMWN10069A01, expressed in bone marrow
A_72_P086861		1,2998	0,0380	down	BX926795 Sus Scrofa library (scan) Sus scrofa cDNA clone scan0015.c.02 5prim, mRNA sequence
A_72_P489254	NRBF2	1,2994	0,0236	down	Sus scrofa nuclear receptor binding factor 2 (NRBF2), mRNA
A_72_P176621	PSMB8	1,2993	0,0376	down	Sus scrofa proteasome (prosome, macropain) subunit, beta type, 8 (large multifunctional peptidase 7) (PSMB8), mRNA
A_72_P056426	MORF4L1	1,2983	0,0062	down	Sus scrofa mortality factor 4 like 1 (MORF4L1), mRNA
A_72_P058051		1,2979	0,0499	down	BX918751 Sus Scrofa library (scan) Sus scrofa cDNA clone scan0035.d.14 5prim, mRNA sequence
A_72_P774098	LOC100623243	1,2979	0,0494	down	Sus scrofa protein phosphatase inhibitor 2-like (LOC100623243), partial mRNA
A_72_P695489		1,2978	0,0385	down	SusFleck_BF_0089_0092_5_pCNS_J02 SusFleck Backfat Sus scrofa cDNA clone 0089_0092_J02 5', mRNA sequence
A_72_P709972	CDKN3	1,2976	0,0268	down	Sus scrofa cyclin-dependent kinase inhibitor 3 (CDKN3), mRNA
A_72_P443593		1,2968	0,0454	down	Sus scrofa mRNA, clone:THY010001C01, expressed in thymus
A_72_P599943	RNF115	1,2960	0,0070	down	PREDICTED: Sus scrofa ring finger protein 115 (RNF115), mRNA
A_72_P207737		1,2953	0,0178	down	1497871 MARC 3PIG Sus scrofa cDNA 5', mRNA sequence
A_72_P471686	PDK1	1,2951	0,0483	down	Sus scrofa pyruvate dehydrogenase kinase, isozyme 1 (PDK1), mRNA
A_72_P184436	KDM3A	1,2950	0,0416	down	Sus scrofa lysine (K)-specific demethylase 3A (KDM3A), mRNA
A_72_P088561	IL13RA1	1,2948	0,0022	down	Sus scrofa interleukin 13 receptor, alpha 1 (IL13RA1), mRNA
A_72_P413063		1,2939	0,0469	down	1544251 MARC 3PIG Sus scrofa cDNA 5', mRNA sequence
A_72_P564112	GPX1	1,2939	0,0248	down	Sus scrofa glutathione peroxidase 1 (GPX1), mRNA
A_72_P384373	JAK3	1,2936	0,0495	down	Sus scrofa Janus kinase 3 (JAK3), mRNA
A_72_P715808	MTIF3	1,2936	0,0408	down	Sus scrofa mitochondrial translational initiation factor 3 (MTIF3), mRNA
A_72_P122941	MICU3	1,2915	0,0383	down	Sus scrofa mitochondrial calcium uptake family, member 3 (MICU3), mRNA
A_72_P506055	SBNO1	1,2915	0,0006	down	PREDICTED: Sus scrofa strawberry notch homolog 1 (Drosophila) (SBNO1), mRNA
A_72_P044976	FOXP1	1,2914	0,0055	down	Sus scrofa forkhead box P1 (FOXP1), mRNA
A_72_P088511	IL4R	1,2903	0,0198	down	Sus scrofa interleukin 4 receptor (IL4R), mRNA
A_72_P013976	KIAA1328	1,2901	0,0312	down	Sus scrofa KIAA1328 ortholog (KIAA1328), mRNA
A_72_P153056		1,2894	0,0217	down	rpliv0125_b4.y1 liv Sus scrofa cDNA 5', mRNA sequence
A_72_P297509		1,2868	0,0096	down	143793 MARC 1PIG Sus scrofa cDNA 5', mRNA sequence
A_72_P179986		1,2854	0,0462	down	Sus scrofa mRNA, clone: PST010076A06, expressed in prostate
A_72_P002551		1,2846	0,0319	down	1106882 MARC 4PIG Sus scrofa cDNA 5', mRNA sequence
A_72_P387078		1,2845	0,0307	down	MI-P-H5-aee-c-02-1-UM.s1.ab1 MI-P-H5 Sus scrofa cDNA clone MI-P-H5-aee-c-02-1-UM.ab1 3', mRNA sequence
A_72_P245102		1,2841	0,0167	down	Sus scrofa tribbles-like protein 1 mRNA, partial cds
A_72_P125706	CYBRD1	1,2839	0,0445	down	Sus scrofa cytochrome b reductase 1 (CYBRD1), mRNA
A_72_P709552	PPP1R2	1,2832	0,0424	down	PREDICTED: Sus scrofa protein phosphatase 1, regulatory (inhibitor) subunit 2 (PPP1R2), mRNA
A_72_P048986		1,2832	0,0150	down	AJ957852 KN404_2 Sus scrofa cDNA clone C0007751l03 5', mRNA sequence
A_72_P037856	ADAMTS15	1,2829	0,0405	down	Sus scrofa ADAM metallopeptidase with thrombospondin type 1 motif, 15 (ADAMTS15), mRNA
A_72_P153251	PDE4A	1,2814	0,0406	down	Sus scrofa phosphodiesterase 4A, cAMP-specific (PDE4A), mRNA
A_72_P361503	WHAMM	1,2810	0,0356	down	Sus scrofa WAS protein homolog associated with actin, golgi membranes and microtubules (WHAMM), mRNA
A_72_P071241		1,2796	0,0046	down	BP439735 full-length enriched swine cDNA library, adult lung Sus scrofa cDNA clone LNG010067G10 5', mRNA sequence
A_72_P141811	NR2C2	1,2795	0,0441	down	Sus scrofa nuclear receptor subfamily 2, group C, member 2 (NR2C2), mRNA
A_72_P441639	MYOC	1,2789	0,0389	down	Sus scrofa myocilin, trabecular meshwork inducible glucocorticoid response (MYOC), mRNA
A_72_P707028		1,2784	0,0483	down	
A_72_P613562	ANP32B	1,2776	0,0193	down	Sus scrofa acidic (leucine-rich) nuclear phosphoprotein 32 family, member B (ANP32B), mRNA
A_72_P079841	AIFM2	1,2774	0,0188	down	Sus scrofa apoptosis-inducing factor, mitochondrion-associated, 2 (AIFM2), mRNA
A_72_P114836		1,2769	0,0387	down	1550885 MARC 3PIG Sus scrofa cDNA 5', mRNA sequence
A_72_P381778		1,2762	0,0263	down	1143198 MARC 4PIG Sus scrofa cDNA 3', mRNA sequence
A_72_P419849		1,2756	0,0443	down	
A_72_P037936	LTBP2	1,2743	0,0390	down	Sus scrofa latent transforming growth factor beta binding protein 2 (LTBP2), mRNA
A_72_P442821	FFAR1	1,2737	0,0162	down	Sus scrofa free fatty acid receptor 1 (FFAR1), mRNA
A_72_P306154		1,2737	0,0018	down	Sus scrofa mRNA, clone:CLNT10003B02, expressed in colon
A_72_P705820		1,2736	0,0249	down	

A_72_P382613		1,2733	0,0082	down	AJ942500 CSEQRAN17 Sus scrofa cDNA clone C0007305b11 5', mRNA sequence
A_72_P442282	STAT5A	1,2718	0,0376	down	Sus scrofa signal transducer and activator of transcription 5A (STAT5A), mRNA
A_72_P013806	TARS	1,2716	0,0470	down	PREDICTED: Sus scrofa threonyl-tRNA synthetase (TARS), mRNA
A_72_P661674	DNMT3A	1,2705	0,0447	down	Sus scrofa DNA (cytosine-5-)-methyltransferase 3 alpha (DNMT3A), mRNA
A_72_P705849	EIF2B2	1,2701	0,0031	down	Sus scrofa eukaryotic translation initiation factor 2B, subunit 2 beta, 39kDa (EIF2B2), mRNA
A_72_P435029		1,2700	0,0304	down	Sus scrofa mRNA, clone:OVRM10137D04, expressed in ovary
A_72_P070206	GBI1	1,2663	0,0129	down	Sus scrofa GBI1 protein (GBI1), mRNA
A_72_P084201		1,2651	0,0045	down	rfat0113_j3.y1 fat Sus scrofa cDNA 5', mRNA sequence
A_72_P328576	SLC31A2	1,2639	0,0074	down	Sus scrofa solute carrier family 31 (copper transporters), member 2 (SLC31A2), mRNA
A_72_P287029	PPP1R9B	1,2632	0,0396	down	Sus scrofa protein phosphatase 1, regulatory subunit 9B (PPP1R9B), mRNA
A_72_P114076	C2H19orf38	1,2624	0,0216	down	Sus scrofa chromosome 2 open reading frame, human C19orf38 (C2H19orf38), mRNA
A_72_P489024	GGPS1	1,2611	0,0406	down	Sus scrofa geranylgeranyl diphosphate synthase 1 (GGPS1), mRNA
A_72_P085261		1,2608	0,0471	down	sto213_m24.z1 sto Sus scrofa cDNA 5', mRNA sequence
A_72_P536281	IRF1	1,2608	0,0474	down	Sus scrofa interferon regulatory factor 1 (IRF1), mRNA
A_72_P518788	RBMX	1,2608	0,0145	down	Sus scrofa RNA binding motif protein, X-linked (RBMX), mRNA
A_72_P209107		1,2607	0,0441	down	260763 MARC 2PIG Sus scrofa cDNA 5', mRNA sequence
A_72_P383398		1,2607	0,0199	down	Sus scrofa mRNA, clone:TCH010019E09, expressed in trachea
A_72_P440606	C-FLIP	1,2606	0,0199	down	Sus scrofa cellular FLICE-like inhibitory protein (C-FLIP), mRNA
A_72_P303044	GNAQ	1,2591	0,0046	down	Sus scrofa guanine nucleotide binding protein (G protein), q polypeptide (GNAQ), mRNA
A_72_P641109	GRN	1,2589	0,0380	down	Sus scrofa granulin (GRN), mRNA
A_72_P194322	ATF6	1,2587	0,0023	down	Sus scrofa activating transcription factor 6 (ATF6), partial mRNA
A_72_P427449	ATXN2L	1,2578	0,0058	down	Sus scrofa ataxin 2-like (ATXN2L), mRNA
A_72_P177501	TKT	1,2574	0,0408	down	Sus scrofa transketolase (TKT), mRNA
A_72_P063506		1,2564	0,0439	down	HX225919 full-length enriched swine cDNA library, adult hypothalamus Sus scrofa cDNA clone HTMT10141D08, mRNA sequence
A_72_P579028	RNF115	1,2550	0,0316	down	PREDICTED: Sus scrofa ring finger protein 115 (RNF115), mRNA
A_72_P440351	FOXO3	1,2549	0,0404	down	Sus scrofa forkhead box O3 (FOXO3), mRNA
A_72_P262032		1,2547	0,0121	down	1543878 MARC 3PIG Sus scrofa cDNA 5', mRNA sequence
A_72_P539428	HSD17B8	1,2521	0,0185	down	Sus scrofa hydroxysteroid (17-beta) dehydrogenase 8 (HSD17B8), mRNA
A_72_P674666	TNFSF13B	1,2519	0,0196	down	Sus scrofa tumor necrosis factor (ligand) superfamily, member 13b (TNFSF13B), mRNA
A_72_P495140	ATP6V1G1	1,2516	0,0181	down	Sus scrofa ATPase, H+ transporting, lysosomal 13kDa, V1 subunit G1 (ATP6V1G1), mRNA
A_72_P618830	ANAPC16	1,2509	0,0334	down	Sus scrofa anaphase promoting complex subunit 16 (ANAPC16), mRNA
A_72_P340418		1,2507	0,0249	down	908928 MARC 4PIG Sus scrofa cDNA 5', mRNA sequence
A_72_P747855	SLC22A15	1,2502	0,0416	down	Sus scrofa solute carrier family 22, member 15 (SLC22A15), mRNA
A_72_P345928	LOC100624357	1,2502	0,0427	down	Sus scrofa nuclear valosin-containing protein-like (LOC100624357), misc_RNA
A_72_P101616		1,2494	0,0279	down	rnmm32c_i4.y1 nmm Sus scrofa cDNA 5', mRNA sequence
A_72_P424724	UBR5	1,2491	0,0047	down	Sus scrofa ubiquitin protein ligase E3 component n-recognin 5 (UBR5), mRNA
A_72_P080211	CATSPER2	1,2489	0,0312	down	Sus scrofa cation channel, sperm associated 2 (CATSPER2), mRNA
A_72_P382523		1,2478	0,0164	down	907587 MARC 4PIG Sus scrofa cDNA 3', mRNA sequence
A_72_P207462	PHF21A	1,2477	0,0140	down	Sus scrofa PHD finger protein 21A (PHF21A), mRNA
A_72_P419709		1,2471	0,0035	down	Pig DNA sequence from clone CH242-452E5 on chromosome 4, complete sequence
A_72_P432109	C4H1orf85	1,2464	0,0178	down	Sus scrofa chromosome 4 open reading frame, human C1orf85 (C4H1orf85), mRNA
A_72_P358463		1,2461	0,0301	down	Sus scrofa mRNA, clone:OVRM10190F12, expressed in ovary
A_72_P055926	LOC100627731	1,2456	0,0212	down	Sus scrofa zinc finger and BTB domain-containing protein 41-like (LOC100627731), mRNA
A_72_P017441	ARV1	1,2441	0,0014	down	Sus scrofa ARV1 homolog (S. cerevisiae) (ARV1), mRNA
A_72_P381068	NAV2	1,2434	0,0328	down	PREDICTED: Sus scrofa neuron navigator 2 (NAV2), transcript variant X1, mRNA
A_72_P391333	LOC100627422	1,2431	0,0403	down	Sus scrofa ligand-dependent corepressor-like (LOC100627422), mRNA
A_72_P158011	FXR2	1,2429	0,0044	down	Sus scrofa fragile X mental retardation, autosomal homolog 2 (FXR2), mRNA
A_72_P085691	RNF219	1,2429	0,0207	down	Sus scrofa ring finger protein 219 (RNF219), mRNA
A_72_P258557		1,2423	0,0270	down	Sus scrofa mRNA, clone:OVR010070G03, expressed in ovary
A_72_P278734	ZNF398	1,2418	0,0300	down	Sus scrofa zinc finger protein 398 (ZNF398), mRNA
A_72_P084306	LOC100524673	1,2406	0,0059	down	Sus scrofa dnaJ homolog subfamily C member 18-like (LOC100524673), mRNA
A_72_P017616		1,2406	0,0203	down	830262 MARC 3PIG Sus scrofa cDNA 3', mRNA sequence
A_72_P477002	CDKN1A	1,2399	0,0303	down	Sus scrofa cyclin-dependent kinase inhibitor 1A (p21, Cip1) (CDKN1A), mRNA
A_72_P178276		1,2384	0,0496	down	1103934 MARC 4PIG Sus scrofa cDNA 3', mRNA sequence
A_72_P335073	SUPT3H	1,2381	0,0301	down	Sus scrofa suppressor of Ty 3 homolog (S. cerevisiae) (SUPT3H), mRNA
A_72_P358648		1,2375	0,0178	down	Select seq gb|EW492796.2|
A_72_P078761	APP	1,2372	0,0467	down	Sus scrofa amyloid beta (A4) precursor protein (APP), mRNA
A_72_P489169	CSGALNACT2	1,2368	0,0102	down	Sus scrofa chondroitin sulfate N-acetylgalactosaminyltransferase 2 (CSGALNACT2), mRNA
A_72_P443517	LOC100156127	1,2351	0,0440	down	PREDICTED: Sus scrofa histone H2B type 1-M (LOC100156127), mRNA
A_72_P531339	SART3	1,2348	0,0014	down	Sus scrofa squamous cell carcinoma antigen recognized by T cells 3 (SART3), mRNA
A_72_P404833	ARHGEF3	1,2348	0,0474	down	Sus scrofa Rho guanine nucleotide exchange factor (GEF) 3 (ARHGEF3), partial mRNA
A_72_P005156	RNF8	1,2341	0,0229	down	Sus scrofa ring finger protein 8, E3 ubiquitin protein ligase (RNF8), mRNA
A_72_P042071	EEF2K	1,2331	0,0050	down	Sus scrofa eukaryotic elongation factor-2 kinase (EEF2K), mRNA
A_72_P311993		1,2329	0,0048	down	AJ963025 KN404_2 Sus scrofa cDNA clone C0007756b05 5', mRNA sequence
A_72_P477541	EFTUD1	1,2312	0,0441	down	Sus scrofa elongation factor Tu GTP binding domain containing 1 (EFTUD1), mRNA
A_72_P305319	SCAMP1	1,2311	0,0459	down	Sus scrofa secretory carrier membrane protein 1 (SCAMP1), mRNA
A_72_P200477	TRAF3	1,2308	0,0378	down	Sus scrofa TNF receptor-associated factor 3 (TRAF3), mRNA
A_72_P313643	ASS1	1,2301	0,0171	down	Sus scrofa argininosuccinate synthase 1 (ASS1), mRNA
A_72_P213607	DECR1	1,2297	0,0017	down	Sus scrofa 2,4-dienoyl CoA reductase 1, mitochondrial (DECR1), mRNA
A_72_P017466	NR2E3	1,2294	0,0462	down	PREDICTED: Sus scrofa nuclear receptor subfamily 2, group E, member 3 (NR2E3), mRNA
A_72_P244692	SIRT2	1,2273	0,0216	down	Sus scrofa sirtuin 2 (SIRT2), mRNA

A_72_P397378	SNAP29	1,2268	0,0402	down	Sus scrofa synaptosomal-associated protein, 29kDa (SNAP29), mRNA
A_72_P227707	MAP3K2	1,2258	0,0105	down	PREDICTED: Sus scrofa mitogen-activated protein kinase kinase kinase 2 (MAP3K2), transcript variant X1, mRNA
A_72_P442415		1,2240	0,0321	down	Sus scrofa mRNA, clone: PST010043A06, expressed in prostate
A_72_P006596	GNG12	1,2236	0,0033	down	Sus scrofa guanine nucleotide binding protein (G protein), gamma 12 (GNG12), mRNA
A_72_P279819		1,2221	0,0170	down	CJ006204 full-length enriched swine cDNA library, adult liver Sus scrofa cDNA clone LVRM1D080189 5', mRNA sequence
A_72_P593749		1,2219	0,0155	down	Sus scrofa mRNA, clone:OVR010034B01, expressed in ovary
A_72_P093036		1,2214	0,0244	down	BX673967 Sus Scrofa library (scac) Sus scrofa cDNA clone scac0044.l.23 5prim, mRNA sequence
A_72_P430939	SUN2	1,2199	0,0361	down	Sus scrofa Sad1 and UNC84 domain containing 2 (SUN2), mRNA
A_72_P340658	SLC16A7	1,2196	0,0358	down	Sus scrofa solute carrier family 16, member 7 (monocarboxylic acid transporter 2) (SLC16A7), mRNA
A_72_P161131	LPCAT1	1,2189	0,0288	down	Sus scrofa lysophosphatidylcholine acyltransferase 1 (LPCAT1), mRNA
A_72_P211432	ANKFY1	1,2173	0,0033	down	Sus scrofa ankyrin repeat and FYVE domain containing 1 (ANKFY1), mRNA
A_72_P200872		1,2169	0,0052	down	Sus scrofa mRNA, clone:OVRM10001G04, expressed in ovary
A_72_P386773	MCOLN1	1,2166	0,0203	down	Sus scrofa mucolipin 1 (MCOLN1), mRNA
A_72_P275939	MYO9B	1,2163	0,0059	down	Sus scrofa myosin IXB (MYO9B), mRNA
A_72_P305579	MLC1	1,2155	0,0089	down	Sus scrofa megalencephalic leukoencephalopathy with subcortical cysts 1 (MLC1), mRNA
A_72_P606248	PI4KA	1,2152	0,0499	down	Sus scrofa phosphatidylinositol 4-kinase, catalytic, alpha (PI4KA), mRNA
A_72_P471478	TBR1	1,2150	0,0056	down	Sus scrofa T-box, brain, 1 (TBR1), mRNA
A_72_P010806	FBXW4	1,2147	0,0271	down	Sus scrofa F-box and WD repeat domain containing 4 (FBXW4), mRNA
A_72_P626578	ARPC1B	1,2133	0,0035	down	Sus scrofa actin related protein 2/3 complex, subunit 1B, 41kDa (ARPC1B), mRNA
A_72_P747726	TMEM66	1,2129	0,0370	down	Sus scrofa transmembrane protein 66 (TMEM66), mRNA
A_72_P290489	PDE6D	1,2127	0,0130	down	Sus scrofa phosphodiesterase 6D, cGMP-specific, rod, delta (PDE6D), mRNA
A_72_P408258	PRPS2	1,2124	0,0046	down	Sus scrofa phosphoribosyl pyrophosphate synthetase 2 (PRPS2), mRNA
A_72_P246212		1,2117	0,0125	down	Pig DNA sequence from clone CH242-488P24 on chromosome X, complete sequence
A_72_P376858	ZNF750	1,2116	0,0307	down	Sus scrofa zinc finger protein 750 (ZNF750), mRNA
A_72_P072351		1,2111	0,0029	down	rthg22_p14.y1 thg Sus scrofa cDNA 5', mRNA sequence
A_72_P444508	PMAIP1	1,2111	0,0203	down	Sus scrofa phorbol-12-myristate-13-acetate-induced protein 1 (PMAIP1), mRNA
A_72_P363908	LOC102158955	1,2109	0,0199	down	Sus scrofa kinesin-like protein KIF3C-like (LOC102158955), mRNA
A_72_P127566	TBC1D20	1,2104	0,0262	down	Sus scrofa TBC1 domain family, member 20 (TBC1D20), mRNA
A_72_P330073	MAP3K13	1,2104	0,0363	down	Sus scrofa mitogen-activated protein kinase kinase kinase 13 (MAP3K13), mRNA
A_72_P287369	KEAP1	1,2089	0,0039	down	Sus scrofa kelch-like ECH-associated protein 1 (KEAP1), mRNA
A_72_P367918		1,2082	0,0307	down	1496071 MARC 3PIG Sus scrofa cDNA 3', mRNA sequence
A_72_P007406	SUV420H1	1,2079	0,0148	down	Sus scrofa suppressor of variegation 4-20 homolog 1 (Drosophila) (SUV420H1), mRNA
A_72_P201467	TRPS1	1,2069	0,0089	down	Sus scrofa trichorhinophalangeal syndrome I (TRPS1), mRNA
A_72_P166401	LOC106510442	1,2068	0,0108	down	PREDICTED: Sus scrofa ankyrin repeat domain-containing protein 11-like (LOC106510442), mRNA
A_72_P389948	SCAF8	1,2065	0,0378	down	Sus scrofa SR-related CTD-associated factor 8 (SCAF8), mRNA
A_72_P276334		1,2059	0,0065	down	rcly14b_n5.y1 cly Sus scrofa cDNA 5', mRNA sequence
A_72_P632976	KDM2A	1,2053	0,0154	down	Sus scrofa lysine (K)-specific demethylase 2A (KDM2A), mRNA
A_72_P027311	FRMD8	1,2051	0,0079	down	Sus scrofa FERM domain containing 8 (FRMD8), mRNA
A_72_P088786	ST3GAL4	1,2046	0,0349	down	Sus scrofa ST3 beta-galactoside alpha-2,3-sialyltransferase 4 (ST3GAL4), mRNA
A_72_P283429	NMRK1	1,2040	0,0463	down	Sus scrofa nicotinamide riboside kinase 1 (NMRK1), mRNA
A_72_P738383	C17H8orf40	1,2030	0,0193	down	Sus scrofa chromosome 8 open reading frame 40 (C17H8orf40), mRNA
A_72_P325153		1,2030	0,0050	down	rpigcf0_004944.y1 ctl Sus scrofa cDNA 5', mRNA sequence
A_72_P298589	SLCO4C1	1,2025	0,0410	down	Sus scrofa solute carrier organic anion transporter family, member 4C1 (SLCO4C1), mRNA
A_72_P001941	PHLDB2	1,2021	0,0188	down	Sus scrofa pleckstrin homology-like domain, family B, member 2 (PHLDB2), partial mRNA
A_72_P290554	RAD52	1,2016	0,0365	down	Sus scrofa RAD52 homolog (S. cerevisiae) (RAD52), mRNA
A_72_P300064		1,2006	0,0146	down	942373 MARC 4PIG Sus scrofa cDNA 5', mRNA sequence
A_72_P626952	PSMD4	1,1996	0,0064	down	Sus scrofa proteasome (prosome, macropain) 26S subunit, non-ATPase, 4 (PSMD4), mRNA [NM_001044535]
A_72_P116231		1,1995	0,0164	down	Rep: Conserved protein - Propionibacterium acnes, partial (10%) [TC550429]
A_72_P492650		1,1990	0,0474	down	
A_72_P074296	TAB2	1,1985	0,0276	down	TGF-beta activated kinase 1/MAP3K7 binding protein 2 [Source:HGNC Symbol;Acc:17075] [ENSSSCT00000034966]
A_72_P416997		1,1971	0,0358	down	
A_72_P192006		1,1960	0,0371	down	Sus scrofa mRNA, clone: HTMT10014F06, expressed in hypothalamus. [AK391669]
A_72_P442544		1,1952	0,0126	down	Uncharacterized protein [Source:UniProtKB/TrEMBL;Acc:F1RUR6] [ENSSSCT00000012152]
A_72_P535819	PPP2R5A	1,1951	0,0270	down	Sus scrofa protein phosphatase 2, regulatory subunit B', alpha (PPP2R5A), mRNA [NM_001100192]
A_72_P143206		1,1947	0,0272	down	PDUts1060G01 Porcine testis cDNA library I Sus scrofa cDNA clone PDUts1060G01 5' similar to homologue to emb [CV868904]
A_72_P613851	CKMT2	1,1946	0,0419	down	Sus scrofa creatine kinase, mitochondrial 2 (sarcomeric) (CKMT2), mRNA [NM_001044551]
A_72_P068911	LAMP-1	1,1937	0,0412	down	Sus scrofa mRNA, clone:OVR010041H06, expressed in ovary. [AK347488]
A_72_P564219	TPM3	1,1933	0,0457	down	Sus scrofa tropomyosin 3 (TPM3), mRNA [NM_001001632]
A_72_P738682	APP	1,1928	0,0021	down	Sus scrofa amyloid beta (A4) precursor protein (APP), mRNA [NM_214372]
A_72_P375023	ZNF692	1,1927	0,0289	down	zinc finger protein 692 [Source:HGNC Symbol;Acc:26049] [ENSSSCT00000024881]
A_72_P443639	HIST1H2BD	1,1922	0,0180	down	Sus scrofa histone cluster 1, H2bd (HIST1H2BD), mRNA [NM_001243656]
A_72_P748776	C-FLIP	1,1914	0,0052	down	Sus scrofa cellular FLICE-like inhibitory protein (C-FLIP), mRNA [NM_001001628]
A_72_P091001	CHD3	1,1911	0,0435	down	Uncharacterized protein [Source:UniProtKB/TrEMBL;Acc:F1ST12] [ENSSSCT00000019548]
A_72_P639144		1,1910	0,0113	down	CAP-GLY domain containing linker protein 1 [Source:HGNC Symbol;Acc:10461] [ENSSSCT00000010735]
A_72_P606643	GLUL	1,1905	0,0410	down	Sus scrofa glutamate-ammonia ligase (GLUL), mRNA. [Source:RefSeq mRNA;Acc:NM_213909] [ENSSSCT00000016935]
A_72_P144036		1,1902	0,0463	down	Rep: Undifferentiated embryonic cell transcription factor 1 - Homo sapiens (Human), partial (28%) [TC599898]

A_72_P489851	TNKS2	1,1901	0,0102	down	tankyrase, TRF1-interacting ankyrin-related ADP-ribose polymerase 2 [Source:HGNC Symbol;Acc:15677] [ENSSSCT00000011453]
A_72_P091116		1,1882	0,0311	down	
A_72_P506040		1,1880	0,0333	down	coiled-coil serine-rich protein 2 [Source:HGNC Symbol;Acc:29197] [ENSSSCT00000011332]
A_72_P507038	IL17RE	1,1880	0,0103	down	interleukin 17 receptor E [Source:HGNC Symbol;Acc:18439] [ENSSSCT00000032890]
A_72_P025521		1,1879	0,0492	down	Sus scrofa mRNA, clone:SKNB10069F06, expressed in skin. [AK349843]
A_72_P543792	ST3GAL4	1,1875	0,0200	down	Sus scrofa ST3 beta-galactoside alpha-2,3-sialyltransferase 4 (ST3GAL4), mRNA [NM_213757]
DCP_1_2		1,1851	0,0353	down	Unknown
A_72_P364188	APP	1,1849	0,0170	down	Sus scrofa amyloid beta (A4) precursor protein (APP), mRNA [NM_214372]
A_72_P131436		1,1847	0,0491	down	Rep: Extracellular solute-binding protein family 5 precursor - Ochrobactrum anthropi (strain ATCC 49188 / DSM 6882 / NCTC 12168), partial (4%) [TC569637]
A_72_P153526	ACTN1	1,1830	0,0281	down	Sus scrofa actinin, alpha 1 (ACTN1), mRNA [NM_001243061]
A_72_P739076	APP	1,1827	0,0345	down	Sus scrofa amyloid beta (A4) precursor protein (APP), mRNA [NM_214372]
A_72_P165661	RBM38	1,1825	0,0195	down	Sus scrofa RNA binding motif protein 38 (RBM38), mRNA [NM_001105312]
A_72_P223322	LOC780415	1,1810	0,0324	down	Sus scrofa interferon regulatory factor 9 (LOC780415), mRNA [NM_001078670]
A_72_P728808	INTS6	1,1809	0,0046	down	integrator complex subunit 6 [Source:HGNC Symbol;Acc:14879] [ENSSSCT00000010282]
A_72_P254612	YBX3	1,1805	0,0387	down	Y box binding protein 3 [Source:HGNC Symbol;Acc:2428] [ENSSSCT00000000680]
A_72_P431694		1,1800	0,0251	down	947694 MARC 4PIG Sus scrofa cDNA 5', mRNA sequence [CN158867]
A_72_P165271	IKBKG	1,1789	0,0308	down	Sus scrofa inhibitor of kappa light polypeptide gene enhancer in B-cells, kinase gamma (IKBKG), mRNA [NM_001113053]
A_72_P231872	NIT1	1,1769	0,0201	down	nitrilase 1 [Source:HGNC Symbol;Acc:7828] [ENSSSCT00000006974]
A_72_P332563		1,1769	0,0277	down	Uncharacterized protein [Source:UniProtKB/TrEMBL;Acc:F1RRJ6] [ENSSSCT00000001879]
A_72_P479388	TRAF3	1,1768	0,0217	down	Uncharacterized protein [Source:UniProtKB/TrEMBL;Acc:F1SA11] [ENSSSCT00000002804]
A_72_P718358	APP	1,1768	0,0479	down	Sus scrofa amyloid beta (A4) precursor protein (APP), mRNA [NM_214372]
A_72_P253542	EPS15	1,1764	0,0403	down	epidermal growth factor receptor pathway substrate 15 [Source:HGNC Symbol;Acc:3419] [ENSSSCT00000004284]
A_72_P194702	SNAPC5	1,1761	0,0495	down	small nuclear RNA activating complex, polypeptide 5, 19kDa [Source:HGNC Symbol;Acc:15484] [ENSSSCT00000025296]
A_72_P657188	COL5A2	1,1761	0,0149	down	Sus scrofa collagen, type V, alpha 2 (COL5A2), mRNA [NM_001105289]
A_72_P332828		1,1760	0,0485	down	
A_72_P550426	TM2D2	1,1760	0,0316	down	Sus scrofa TM2 domain containing 2 (TM2D2), mRNA [NM_001244627]
A_72_P040086	ZBTB7A	1,1755	0,0387	down	zinc finger and BTB domain containing 7A [Source:HGNC Symbol;Acc:18078] [ENSSSCT00000028851]
A_72_P097051	SH2B3	1,1750	0,0372	down	SH2B adaptor protein 3 [Source:HGNC Symbol;Acc:29605] [ENSSSCT00000034933]
A_72_P345513		1,1714	0,0351	down	Rep: Brain protein I3 - Bos taurus (Bovine), complete [TC534832]
A_72_P251322		1,1714	0,0165	down	E1A binding protein p400 [Source:HGNC Symbol;Acc:11958] [ENSSSCT00000027939]
A_72_P138971		1,1705	0,0394	down	rpfco0135_a20.y1 fco Sus scrofa cDNA 5', mRNA sequence [EW190742]
A_72_P601263	KLF13	1,1704	0,0277	down	Sus scrofa Kruppel-like factor 13 (KLF13) mRNA, complete cds. [GQ274321]
A_72_P224907	PHLPP1	1,1700	0,0433	down	PH domain and leucine rich repeat protein phosphatase 1 [Source:HGNC Symbol;Acc:20610] [ENSSSCT00000005402]
A_72_P304604		1,1690	0,0438	down	Rep: Membrane-associated ring finger (C3HC4) 7 - Mus musculus (Mouse), partial (23%) [TC525466]
A_72_P201857		1,1688	0,0336	down	
A_72_P026581	STK38	1,1686	0,0061	down	serine/threonine kinase 38 [Source:HGNC Symbol;Acc:17847] [ENSSSCT00000001742]
DCP_1_0		1,1685	0,0458	down	Unknown
A_72_P016331		1,1682	0,0028	down	Rep: DNA polymerase III, subunits gamma and tau - Ralstonia pickettii 12D, partial (4%) [TC571510]
A_72_P428649	ARRDC4	1,1679	0,0386	down	arrestin domain containing 4 [Source:HGNC Symbol;Acc:28087] [ENSSSCT00000002508]
A_72_P146756	RIT1	1,1669	0,0119	down	Sus scrofa Ras-like without CAAX 1 (RIT1), mRNA [NM_001077230]
A_72_P495781	GTF3C4	1,1668	0,0312	down	general transcription factor IIIC, polypeptide 4, 90kDa [Source:HGNC Symbol;Acc:4667] [ENSSSCT00000006302]
A_72_P734168	APP	1,1668	0,0069	down	Sus scrofa amyloid beta (A4) precursor protein (APP), mRNA [NM_214372]
DCP_1_1		1,1668	0,0350	down	Unknown
A_72_P602293	CDC5L	1,1644	0,0205	down	cell division cycle 5-like [Source:HGNC Symbol;Acc:1743] [ENSSSCT00000001908]
A_72_P258317	NHLRC2	1,1642	0,0368	down	Sus scrofa NHL repeat containing 2 (NHLRC2), mRNA [NM_001243561]
A_72_P530745		1,1639	0,0246	down	Rep: Guanylyl cyclase-activating protein 2 (GCAP 2) (Guanylate cyclase activator 1B). - Canis familiaris, partial (52%) [TC547899]
A_72_P430569		1,1638	0,0096	down	solute carrier family 8 (sodium/lithium/calcium exchanger), member B1 [Source:HGNC Symbol;Acc:26175] [ENSSSCT00000010829]
A_72_P678715	MESP1	1,1631	0,0429	down	mesoderm posterior 1 homolog (mouse) [Source:HGNC Symbol;Acc:29658] [ENSSSCT00000002070]
A_72_P060581		1,1631	0,0144	down	Uncharacterized protein [Source:UniProtKB/TrEMBL;Acc:I3LD16] [ENSSSCT00000024699]
A_72_P073556	FAM134A	1,1629	0,0470	down	family with sequence similarity 134, member A [Source:HGNC Symbol;Acc:28450] [ENSSSCT00000017648]
A_72_P070851		1,1626	0,0064	down	Rep: Myb protein P42POP - Mus musculus (Mouse), partial (5%) [TC576306]
A_72_P245812	VNN1	1,1617	0,0186	down	Sus scrofa mRNA, clone:OVRM10042A10, expressed in ovary. [AK235021]
A_72_P318799	C-FLIP	1,1605	0,0060	down	Sus scrofa cellular FLICE-like inhibitory protein (C-FLIP), mRNA. [Source:RefSeq mRNA;Acc:NM_001001628] [ENSSSCT00000036156]
A_72_P242707	PPM1F	1,1605	0,0353	down	protein phosphatase, Mg2+/Mn2+ dependent, 1F [Source:HGNC Symbol;Acc:19388] [ENSSSCT00000011040]
A_72_P133976	EPAS1	1,1603	0,0090	down	Sus scrofa endothelial PAS domain protein 1 (EPAS1), mRNA [NM_001097420]
(+)E1A_r60_1		1,1592	0,0364	down	Unknown
A_72_P008921		1,1592	0,0226	down	
A_72_P027471		1,1592	0,0367	down	Rep: procollagen-proline, 2-oxoglutarate 4-dioxygenase (proline 4-hydroxylase), alpha II polypeptide - Mus musculus, partial (12%) [TC552921]

A_72_P307908	PITPNB	1,1586	0,0126	down	phosphatidylinositol transfer protein, beta [Source:HGNC Symbol;Acc:9002] [ENSSSCT00000010923]
A_72_P216607		1,1582	0,0416	down	Rep: PaREP2b - Pyrobaculum aerophilum, partial (6%) [TC539323]
A_72_P538413	ASB6	1,1582	0,0495	down	Sus scrofa ankyrin repeat and SOCS box containing 6 (ASB6), mRNA [NM_001099937]
A_72_P359053	EPS15	1,1580	0,0259	down	epidermal growth factor receptor pathway substrate 15 [Source:HGNC Symbol;Acc:3419] [ENSSSCT00000004284]
A_72_P725743	WDFY2	1,1560	0,0385	down	Sus scrofa mRNA, clone:LVRM10032G05, expressed in liver. [AK232818]
A_72_P178166	CDKN1B	1,1553	0,0300	down	Sus scrofa cyclin-dependent kinase inhibitor 1B (p27, Kip1) (CDKN1B), mRNA [NM_214316]
A_72_P251482		1,1545	0,0495	down	
A_72_P181471		1,1538	0,0139	down	Uncharacterized protein [Source:UniProtKB/TrEMBL;Acc:I3LR82] [ENSSSCT00000031293]
A_72_P397039	NUP133	1,1538	0,0206	down	nucleoporin 133kDa [Source:HGNC Symbol;Acc:18016] [ENSSSCT00000011155]
A_72_P020791	NPHP1	1,1531	0,0293	down	nephronophthisis 1 (juvenile) [Source:HGNC Symbol;Acc:7905] [ENSSSCT00000035691]
A_72_P129521	RUNDC3A	1,1518	0,0117	down	Sus scrofa mRNA, clone:ADR010091D08, expressed in adrenal gland. [AK343555]
A_72_P488244		1,1511	0,0163	down	Uncharacterized protein [Source:UniProtKB/TrEMBL;Acc:F1RKD3] [ENSSSCT00000010814]
A_72_P015826		1,1508	0,0483	down	Rep: eIF2 - Pichia stipitis (Yeast), partial (10%) [TC600391]
A_72_P315788		1,1501	0,0034	down	
A_72_P442582	LOC733646	1,1500	0,0405	down	Sus scrofa lysosomal 9kDa H+ transporting ATPase V0 subunit e (LOC733646), mRNA [NM_001044579]
A_72_P503592	FAM21C	1,1485	0,0442	down	Uncharacterized protein [Source:UniProtKB/TrEMBL;Acc:F1SDV2] [ENSSSCT00000011385]
A_72_P320883		1,1484	0,0387	down	Rep: interferon regulatory factor 2 binding protein 2 isoform A - Homo sapiens, partial (36%) [TC610616]
A_72_P185416		1,1482	0,0476	down	
A_72_P214652	DDX18	1,1455	0,0305	down	Uncharacterized protein [Source:UniProtKB/TrEMBL;Acc:F1RY13] [ENSSSCT00000025125]
A_72_P096591		1,1445	0,0215	down	Uncharacterized protein [Source:UniProtKB/TrEMBL;Acc:F1SGW8] [ENSSSCT00000000878]
A_72_P255157	TMCC2	1,1439	0,0282	down	transmembrane and coiled-coil domain family 2 [Source:HGNC Symbol;Acc:24239] [ENSSSCT00000016658]
A_72_P359363		1,1431	0,0291	down	
A_72_P047026		1,1430	0,0074	down	AJ957172 KN404_2 Sus scrofa cDNA clone C0007753m03 3', mRNA sequence [AJ957172]
A_72_P100816	C6H1orf174	1,1425	0,0219	down	Sus scrofa chromosome 1 open reading frame 174 (C6H1orf174), mRNA [NM_001244820]
A_72_P010796		1,1419	0,0154	down	Sus scrofa mRNA, clone: TES010038G04, expressed in testis. [AK398200]
A_72_P430194	LOC100521274	1,1395	0,0079	down	Sus scrofa mRNA, clone:PTG010092E04, expressed in pituitary gland. [AK349514]
A_72_P430694	ALG5	1,1384	0,0236	down	Sus scrofa mRNA, clone:THY010049D06, expressed in thymus. [AK238941]
A_72_P321508	KLF7	1,1384	0,0222	down	Krueppel-like factor 7 [Source:RefSeq peptide;Acc:NP_001090956] [ENSSSCT00000031970]
A_72_P430219		1,1375	0,0071	down	Rep: TonB family protein - Anaeromyxobacter sp. (strain Fw109-5), partial (9%) [TC553127]
A_72_P097881	KAT6B	1,1338	0,0438	down	K(lysine) acetyltransferase 6B [Source:HGNC Symbol;Acc:17582] [ENSSSCT00000011293]
A_72_P442654	STAT2	1,1336	0,0455	down	Sus scrofa signal transducer and activator of transcription 2, 113kDa (STAT2), mRNA [NM_213889]
A_72_P086121		1,1329	0,0486	down	cytochrome P450, family 20, subfamily A, polypeptide 1 [Source:HGNC Symbol;Acc:20576] [ENSSSCT00000024501]
A_72_P442424	LOC100739444	1,1316	0,0348	down	Sus scrofa mRNA, clone:ITT010040A03, expressed in small intestine. [AK345026]
A_72_P199947		1,1305	0,0485	down	Rep: Phosphatidylinositol 4-kinase type 2-beta (EC 2.7.1.67) (Phosphatidylinositol 4-kinase type II-beta). - Canis familiaris, partial (39%) [TC599400]
A_72_P208917		1,1300	0,0209	down	BX675220 Sus Scrofa library (scac) Sus scrofa cDNA clone scac0030.f.03 3prim, mRNA sequence [BX675220]
A_72_P203497		1,1288	0,0227	down	Rep: Acyl-CoA dehydrogenase family member 10 (ACAD-10). - Bos Taurus, partial (33%) [TC620433]
A_72_P103467		1,1257	0,0150	down	Rep: Undecaprenyl-diphosphatase 1 - Clostridium acetobutylicum, partial (6%) [TC542440]
A_72_P398008		1,1246	0,0247	down	Rep: Precorrin-2 methylase - Thermus thermophilus (strain HB8 / ATCC 27634 / DSM 579), partial (8%) [TC611063]
A_72_P490731	PTPRE	1,1231	0,0317	down	protein tyrosine phosphatase, receptor type, E [Source:HGNC Symbol;Acc:9669] [ENSSSCT00000011767]
A_72_P052386		1,1223	0,0235	down	Rep: FLORICAULA/LEAFY-like protein - Solanum lycopersicum (Tomato) (Lycopersicon esculentum), partial (5%) [TC613065]
A_72_P232152	EFTUD1	1,1222	0,0072	down	Sus scrofa mRNA, clone:PBL010056D05, expressed in peripheral blood mononuclear cells. [AK348895]
A_72_P370408	KLF13	1,1214	0,0440	down	Sus scrofa Kruppel-like factor 13 (KLF13) mRNA, complete cds. [GQ274321]
A_72_P493617	PARP16	1,1204	0,0279	down	poly (ADP-ribose) polymerase family, member 16 [Source:HGNC Symbol;Acc:26040] [ENSSSCT00000005439]
A_72_P341863		1,1194	0,0452	down	Rep: GPI ethanolamine phosphate transferase 1 - Mus musculus (Mouse), partial (4%) [TC547800]
A_72_P234607	USP46	1,1187	0,0436	down	ubiquitin specific peptidase 46 [Source:HGNC Symbol;Acc:20075] [ENSSSCT00000030149]
A_72_P531514		1,1154	0,0174	down	WW domain binding protein 11 [Source:HGNC Symbol;Acc:16461] [ENSSSCT00000000657]
A_72_P657354	IGBP1	1,1139	0,0163	down	PREDICTED: Sus scrofa immunoglobulin (CD79A) binding protein 1, transcript variant 2 (IGBP1), mRNA [XM_001927382]
A_72_P339183	LOC100626218	1,1134	0,0169	down	Uncharacterized protein [Source:UniProtKB/TrEMBL;Acc:I3LPU1] [ENSSSCT00000027588]
A_72_P206617		1,1130	0,0445	down	BP158130 full-length enriched swine cDNA library, adult spleen Sus scrofa cDNA clone SPL010028E04 5', mRNA sequence [BP158130]
A_72_P073526		1,1120	0,0245	down	807308 MARC 3PIG Sus scrofa cDNA 5', mRNA sequence [CF178259]
A_72_P125901		1,1098	0,0230	down	Rep: Uncharacterized protein C19orf28 - Homo sapiens (Human), partial (62%) [TC582869]
A_72_P585246	PCIF1	1,1074	0,0286	down	PDX1 C-terminal inhibiting factor 1 [Source:HGNC Symbol;Acc:16200] [ENSSSCT00000008136]
A_72_P647594	SRSF11	1,1063	0,0488	down	Sus scrofa serine/arginine-rich splicing factor 11 (SRSF11), mRNA [NM_001044587]
A_72_P556495	ASTE1	1,1024	0,0487	down	asteroid homolog 1 (Drosophila) [Source:HGNC Symbol;Acc:25021] [ENSSSCT00000035248]
