# Supplementary material for: Transcriptomic Analysis of Porcine Endometrium during Implantation after In Vitro Stimulation by Adiponectin
Source: Int J Mol Sci. 2019 Mar 16;20(6):1335. doi: 10.3390/ijms20061335 (PMC6470965; doi:10.3390/ijms20061335)
Supplement: Supplementary file 1 [file ijms-20-01335-s001.zip › SF.2..pdf]

Supplementary Table 2

Gene onthology (GO) analysis generated from up-regulated and down-regulated DE-genes

Category				Gene numbers	p-value	Genes
		**GO - Biological Process**				
**GO:0010467**	**Gene expression**			**141**	**6,47E-04**	**MORF4L1, STAT5A, LOC100158121, NAA15, SYNCRIP, RPS6KB1, FOXO3, ENPEP, NR2E3, NFXL1, SART3, CITED2, KDM1B, APP, MDFIC, WWP1, ZNF445, RARB, EIF2B2, SAMD4A, GNL3, PPP1CC, TBR1, HHEX, EP300, EIF2S1, MED17, TFAP2A, TNFAIP3, TDRD7, NFKBIA, KEAP1, MYBL2, CCL4, UHMK1, NR2C2, RPS27, HNRNPK, DDX46, KLKB1, LEO1, HELLS, TRAF3, TAF2, ZNF398, IKZF4, DNMT3A, TAF5, SMAD7, KLF11, MPHOSPH10, LOC100154071, DENR, FXR2, RBMX, FOXP1, ATM, SFRP5, ATF6, RNF8, ETS1, WDR3, PHEX, PABPC1L, GRB7, ABCF1, COPS5, CPEB2, CPEB4, CCNT1, ZNF518A, LOC100627422, DDR2, CDC45, NOS2, MSN, CASP1, KDM5B, FANCA, LOC100621389, CHUK, FTSJ3, LOC100153329, ABCE1, TBL1XR1, SNAPC5, CCNK, ACTA1, ARID5B, LDB1, SAA3, TLE4, EIF4G1, TARS, INHBA, FANCD2, HIPK3, UCHL5, CUX2, SBNO1, MTDH, UBE3A, ZBTB11, SAV1, NR3C1, NEO1, VARS, DTNBP1, ZFP36L1, TFAM, SF3B1, TNFRSF1B, MAP3K2, AGO1, CHD2, MYCBP, KDM3A, PHF20L1, INPP5B, BCL9, CHD3, CEBPA, BRD2, KAT2B, CEBPB, ZNF667, RGS14, SIRT2, SOD2, ATXN1, RPS6KA3, PAPOLA, HBZ, IRF1, NOP58, NOP56, ZBTB1, BMPR1A, RNF111**
*GO:0006351	Transcription, DNA-templated			91	4,36E-05	MORF4L1, COPS5, STAT5A, LOC100158121, CCNT1, NAA15, FOXO3, NR2E3, ZNF518A, NFXL1, LOC100627422, DDR2, CITED2, CDC45, APP, MDFIC, WWP1, ZNF445, RARB, KDM5B, FANCA, LOC100153329, LOC100621389, CHUK, GNL3, SNAPC5, TBL1XR1, CCNK, LDB1, ARID5B, SAA3, TLE4, TBR1, INHBA, HHEX, EP300, FANCD2, HIPK3, MED17, UCHL5, TFAP2A, CUX2, TNFAIP3, MTDH, UBE3A, SAV1, NFKBIA, KEAP1, NR3C1, NEO1, MYBL2, NR2C2, TFAM, HNRNPK, MAP3K2, AGO1, LEO1, KDM3A, HELLS, BCL9, TRAF3, CEBPA, TAF2, IKZF4, DNMT3A, BRD2, KAT2B, CEBPB, SMAD7, TAF5, KLF11, ZNF667, LOC100154071, RBMX, FOXP1, RGS14, SIRT2, SOD2, SFRP5, ATF6, RNF8, ATXN1, RPS6KA3, ETS1, HBZ, IRF1, ZBTB1, RNF111, BMPR1A
	*GO:0006366	Transcription from RNA polymerase II promoter		51	3,77E-03	COPS5, STAT5A, CCNT1, NR2E3, NFXL1, LOC100627422, CITED2, APP, RARB, CHUK, GNL3, SNAPC5, TBL1XR1, CCNK, LDB1, TLE4, TBR1, INHBA, HHEX, EP300, MED17, TFAP2A, MTDH, UBE3A, NFKBIA, NEO1, NR3C1, NR2C2, AGO1, LEO1, KDM3A, BCL9, CEBPA, TAF2, DNMT3A, IKZF4, KAT2B, TAF5, SMAD7, KLF11, RBMX, SIRT2, FOXP1, RNF8, ATF6, RPS6KA3, ETS1, HBZ, IRF1, ZBTB1, BMPR1A
**GO:0034645**	**Cellular macromolecule biosynthetic process**			**131**	**5,09E-04**	**MORF4L1, STAT5A, LOC100158121, NAA15, SYNCRIP, RPS6KB1, FOXO3, NR2E3, NFXL1, CITED2, ST3GAL1, APP, APOA1, MDFIC, ST3GAL4, WWP1, ZNF445, RARB, EIF2B2, SAMD4A, GNL3, POLG, TBR1, HHEX, EP300, EIF2S1, MED17, MCMBP, TFAP2A, TNFAIP3, ENPP1, NFKBIA, KEAP1, MYBL2, NR2C2, UHMK1, RPS27, HNRNPK, LEO1, HELLS, TRAF3, TAF2, ZNF398, IKZF4, DNMT3A, SMAD7, TAF5, KLF11, LOC100154071, DENR, FXR2, RBMX, FOXP1, ATM, SFRP5, ATF6, RNF8, ETS1, PHEX, GRB7, ALG12, ABCF1, CPEB2, COPS5, CPEB4, CCNT1, DPY19L4, ZNF518A, LOC100627422, DDR2, CDC45, KDM5B, TNIP1, FANCA, LOC100621389, CHUK, LOC100153329, ABCE1, TBL1XR1, SNAPC5, CCNK, ARID5B, LDB1, PIGV, SAA3, CHST3, TLE4, EIF4G1, TARS, INHBA, FANCD2, HIPK3, UCHL5, CHSY1, CUX2, SBNO1, MTDH, UBE3A, ZBTB11, SAV1, NR3C1, NEO1, VARS, ZFP36L1, TFAM, MAP3K2, AGO1, CHD2, MYCBP, KDM3A, PHF20L1, BCL9, CHD3, CEBPA, BRD2, KAT2B, CEBPB, ZNF667, RGS14, SIRT2, SOD2, ATXN1, RPS6KA3, HBZ, CSGALNACT2, IRF1, ZBTB1, RNF111, BMPR1A**
*GO:0018130	Heterocycle biosynthetic process			107	1,66E-03	MORF4L1, STAT5A, LOC100158121, NAA15, FOXO3, NR2E3, NFXL1, CITED2, TPK1, APP, MDFIC, WWP1, RARB, ZNF445, ATP8, SAMD4A, GNL3, TBR1, HHEX, EP300, MED17, TFAP2A, TNFAIP3, PRPS2, NFKBIA, KEAP1, MRAP2, MYBL2, CCL4, NR2C2, HNRNPK, LEO1, HELLS, TRAF3, ZNF398, TAF2, IKZF4, DNMT3A, SMAD7, TAF5, KLF11, LOC100154071, RBMX, FOXP1, RNF8, ATF6, SFRP5, ETS1, PHEX, COPS5, CCNT1, ZNF518A, DDR2, LOC100627422, CDC45, ATP5O, FANCA, KDM5B, LOC100153329, CHUK, LOC100621389, TBL1XR1, SNAPC5, CCNK, LDB1, ARID5B, SAA3, TLE4, INHBA, FANCD2, HIPK3, UCHL5, CUX2, SBNO1, MTDH, UBE3A, SAV1, ZBTB11, NR3C1, NEO1, TFAM, MAP3K2, AGO1, CHD2, MYCBP, KDM3A, PHF20L1, CHD3, BCL9, CEBPA, BRD2, KAT2B, CEBPB, ZNF667, AMPD3, SIRT2, RGS14, SOD2, ATXN1, RPS6KA3, HBZ, IRF1, ZBTB1, RNF111, BMPR1A
	*GO:0034654	Nucleobase-containing compound biosynthetic process		106	1,30E-03	MORF4L1, STAT5A, LOC100158121, NAA15, FOXO3, NR2E3, NFXL1, CITED2, APP, MDFIC, WWP1, RARB, ZNF445, ATP8, SAMD4A, GNL3, TBR1, HHEX, EP300, MED17, TFAP2A, TNFAIP3, PRPS2, NFKBIA, KEAP1, MRAP2, MYBL2, CCL4, NR2C2, HNRNPK, LEO1, HELLS, TRAF3, ZNF398, TAF2, IKZF4, DNMT3A, SMAD7, TAF5, KLF11, LOC100154071, RBMX, FOXP1, RNF8, ATF6, SFRP5, ETS1, PHEX, COPS5, CCNT1, ZNF518A, DDR2, LOC100627422, CDC45, ATP5O, FANCA, KDM5B, LOC100153329, CHUK, LOC100621389, TBL1XR1, SNAPC5, CCNK, LDB1, ARID5B, SAA3, TLE4, INHBA, FANCD2, HIPK3, UCHL5, CUX2, SBNO1, MTDH, UBE3A, SAV1, ZBTB11, NR3C1, NEO1, TFAM, MAP3K2, CHD2, AGO1, MYCBP, KDM3A, PHF20L1, CHD3, BCL9, CEBPA, BRD2, KAT2B, CEBPB, ZNF667, AMPD3, SIRT2, RGS14, SOD2, ATXN1, RPS6KA3, HBZ, IRF1, ZBTB1, RNF111, BMPR1A
	**GO:0019438	Aromatic compound biosynthetic process		107	1,82E-03	MORF4L1, STAT5A, LOC100158121, NAA15, FOXO3, NR2E3, NFXL1, CITED2, TPK1, APP, MDFIC, WWP1, RARB, ZNF445, ATP8, SAMD4A, GNL3, TBR1, HHEX, EP300, MED17, TFAP2A, TNFAIP3, PRPS2, NFKBIA, KEAP1, MRAP2, MYBL2, CCL4, NR2C2, HNRNPK, LEO1, HELLS, TRAF3, ZNF398, TAF2, IKZF4, DNMT3A, SMAD7, TAF5, KLF11, LOC100154071, RBMX, FOXP1, RNF8, ATF6, SFRP5, ETS1, PHEX, COPS5, CCNT1, ZNF518A, DDR2, LOC100627422, CDC45, ATP5O, FANCA, KDM5B, LOC100153329, CHUK, LOC100621389, TBL1XR1, SNAPC5, CCNK, LDB1, ARID5B, SAA3, TLE4, INHBA, FANCD2, HIPK3, UCHL5, CUX2, SBNO1, MTDH, UBE3A, SAV1, ZBTB11, NR3C1, NEO1, TFAM, MAP3K2, AGO1, CHD2, MYCBP, KDM3A, PHF20L1, CHD3, BCL9, CEBPA, BRD2, KAT2B, CEBPB, ZNF667, AMPD3, SIRT2, RGS14, SOD2, ATXN1, RPS6KA3, HBZ, IRF1, ZBTB1, RNF111, BMPR1A
**GO:0010468**	**Regulation of gene expression**			**118**	**7,30E-06**	**MORF4L1, STAT5A, LOC100158121, NAA15, SYNCRIP, RPS6KB1, FOXO3, NR2E3, CITED2, KDM1B, APP, MDFIC, WWP1, RARB, EIF2B2, SAMD4A, GNL3, PPP1CC, TBR1, HHEX, EP300, EIF2S1, MED17, TFAP2A, TNFAIP3, TDRD7, NFKBIA, KEAP1, MYBL2, CCL4, NR2C2, UHMK1, DDX46, HNRNPK, LEO1, HELLS, TRAF3, TAF2, ZNF398, IKZF4, DNMT3A, SMAD7, KLF11, LOC100154071, FXR2, RBMX, FOXP1, ATM, RNF8, ATF6, SFRP5, ETS1, PHEX, GRB7, ABCF1, CPEB2, COPS5, CPEB4, CCNT1, LOC100627422, DDR2, CDC45, NOS2, MSN, KDM5B, FANCA, CHUK, LOC100153329, TBL1XR1, CCNK, ACTA1, ARID5B, LDB1, SAA3, TLE4, EIF4G1, INHBA, FANCD2, UCHL5, CUX2, SBNO1, MTDH, UBE3A, ZBTB11, SAV1, NR3C1, NEO1, DTNBP1, ZFP36L1, TFAM, TNFRSF1B, MAP3K2, AGO1, CHD2, MYCBP, KDM3A, PHF20L1, INPP5B, BCL9, CHD3, CEBPA, BRD2, KAT2B, CEBPB, ZNF667, SIRT2, RGS14, SOD2, ATXN1, RPS6KA3, PAPOLA, HBZ, IRF1, ZBTB1, RNF111, BMPR1A**
*GO:0010628	Positive regulation of gene expression			53	7,62E-04	ABCF1, COPS5, STAT5A, CCNT1, NAA15, RPS6KB1, NR2E3, APP, MDFIC, RARB, MSN, KDM5B, SAMD4A, CHUK, GNL3, TBL1XR1, CCNK, ACTA1, LDB1, TBR1, HHEX, INHBA, EP300, MED17, TFAP2A, CUX2, UBE3A, NFKBIA, NR3C1, DTNBP1, NR2C2, UHMK1, TFAM, MAP3K2, AGO1, LEO1, KDM3A, BCL9, CEBPA, TAF2, IKZF4, KAT2B, CEBPB, RBMX, SIRT2, FOXP1, ATF6, RPS6KA3, ETS1, IRF1, RNF111, BMPR1A
*GO:0010629	Negative regulation of gene expression			43	7,43E-03	MORF4L1, MTDH, CPEB2, CPEB4, CCNT1, LOC100158121, SYNCRIP, NR2E3, LOC100627422, CCL4, ZFP36L1, CDC45, MDFIC, WWP1, AGO1, RARB, NOS2, KDM5B, LOC100153329, SAMD4A, HELLS, CEBPA, TBL1XR1, DNMT3A, IKZF4, SMAD7, LDB1, KLF11, LOC100154071, SAA3, TLE4, FXR2, FOXP1, SIRT2, ATXN1, RNF8, EP300, EIF2S1, HBZ, IRF1, TFAP2A, GRB7, ZBTB1
	*GO:0016458	Gene silencing		8	5,06E-02	MORF4L1, DNMT3A, CDC45, LOC100158121, AGO1, LOC100154071, HELLS, LOC100153329
		*GO:0006342	Chromatin silencing	7	1,10E-02	MORF4L1, DNMT3A, CDC45, LOC100158121, LOC100154071, HELLS, LOC100153329
	*GO:0045814	Negative regulation of gene expression,		7	1,45E-02	MORF4L1, DNMT3A, CDC45, LOC100158121, LOC100154071, HELLS, LOC100153329
*GO:0010608	Posttranscriptional regulation of gene expression			18	2,23E-03	ABCF1, CPEB2, TDRD7, CPEB4, SYNCRIP, RPS6KB1, FOXO3, FXR2, UHMK1, EIF4G1, ZFP36L1, TNFRSF1B, APP, EIF2S1, AGO1, EIF2B2, GRB7, SAMD4A
	*GO:0006417	Regulation of translation		16	2,27E-03	ABCF1, CPEB2, CPEB4, SYNCRIP, RPS6KB1, FOXO3, FXR2, UHMK1, EIF4G1, ZFP36L1, APP, EIF2S1, AGO1, EIF2B2, GRB7, SAMD4A
		**GO:0017148	Negative regulation of translation	9	8,77E-03	ZFP36L1, CPEB2, EIF2S1, CPEB4, AGO1, SYNCRIP, FXR2, GRB7, SAMD4A
	*GO:0040029	Regulation of gene expression, epigenetic		9	5,98E-02	MORF4L1, KDM1B, DNMT3A, CDC45, LOC100158121, AGO1, LOC100154071, HELLS, LOC100153329

**GO:0051171**	**Regulation of nitrogen compound metabolic process**							**117**	**1,77E-05**	**MORF4L1, STAT5A, LOC100158121, NAA15, SYNCRIP, RPS6KB1, FOXO3, NR2E3, CITED2, KDM1B, APP, MDFIC, WWP1, RARB, EIF2B2, SAMD4A, GNL3, FBP1, TBR1, HHEX, EP300, EIF2S1, MED17, TFAP2A, TNFAIP3, ME1, ASS1, NFKBIA, KEAP1, MRAP2, MYBL2, CCL4, NR2C2, UHMK1, HNRNPK, LPCAT1, LEO1, HELLS, TRAF3, TAF2, ZNF398, IKZF4, DNMT3A, SMAD7, KLF11, LOC100154071, FXR2, RBMX, FOXP1, RNF8, ATF6, SFRP5, ETS1, PARPBP, PHEX, GRB7, ABCF1, CPEB2, COPS5, CPEB4, CCNT1, DDR2, LOC100627422, CDC45, KDM5B, FANCA, CHUK, LOC100153329, TBL1XR1, CCNK, ARID5B, LDB1, SAA3, TLE4, RAD52, EIF4G1, INHBA, FANCD2, UCHL5, SBNO1, MTDH, UBE3A, SAV1, ZBTB11, NR3C1, NEO1, ZFP36L1, TFAM, MAP3K2, AGO1, CHD2, MYCBP, KDM3A, PHF20L1, ACSL3, TRIP12, BCL9, CHD3, CEBPA, BRD2, KAT2B, CEBPB, ZNF667, SIRT2, RGS14, SOD2, ATXN1, SAMD8, RPS6KA3, PAPOLA, HBZ, IRF1, ZBTB1, RNF111, BMPR1A**
*GO:0019219	Regulation of nucleobase-containing compound metabolic process							101	1,50E-03	MORF4L1, STAT5A, LOC100158121, NAA15, NR2E3, CITED2, KDM1B, APP, MDFIC, WWP1, RARB, SAMD4A, GNL3, FBP1, TBR1, HHEX, EP300, MED17, TFAP2A, TNFAIP3, ME1, NFKBIA, KEAP1, MRAP2, MYBL2, CCL4, NR2C2, UHMK1, HNRNPK, LEO1, HELLS, TRAF3, ZNF398, TAF2, IKZF4, DNMT3A, SMAD7, KLF11, LOC100154071, RBMX, FOXP1, RNF8, ATF6, SFRP5, ETS1, PARPBP, PHEX, COPS5, CCNT1, DDR2, LOC100627422, CDC45, FANCA, KDM5B, LOC100153329, CHUK, TBL1XR1, CCNK, LDB1, ARID5B, SAA3, TLE4, RAD52, INHBA, FANCD2, UCHL5, SBNO1, MTDH, UBE3A, SAV1, ZBTB11, NR3C1, NEO1, TFAM, MAP3K2, CHD2, AGO1, KDM3A, MYCBP, PHF20L1, TRIP12, CHD3, BCL9, CEBPA, BRD2, KAT2B, CEBPB, ZNF667, SIRT2, RGS14, SOD2, ATXN1, RPS6KA3, PAPOLA, HBZ, IRF1, ZBTB1, RNF111, BMPR1A
	*GO:0051252	Regulation of RNA metabolic process						94	6,65E-04	MORF4L1, COPS5, STAT5A, LOC100158121, CCNT1, NAA15, NR2E3, LOC100627422, DDR2, CITED2, CDC45, APP, MDFIC, WWP1, RARB, KDM5B, FANCA, LOC100153329, CHUK, SAMD4A, GNL3, TBL1XR1, CCNK, LDB1, ARID5B, SAA3, TLE4, TBR1, INHBA, HHEX, EP300, FANCD2, MED17, UCHL5, TFAP2A, TNFAIP3, SBNO1, MTDH, UBE3A, ZBTB11, SAV1, NFKBIA, KEAP1, NR3C1, NEO1, MYBL2, CCL4, NR2C2, UHMK1, TFAM, HNRNPK, MAP3K2, AGO1, CHD2, LEO1, MYCBP, KDM3A, PHF20L1, HELLS, BCL9, CHD3, TRAF3, CEBPA, ZNF398, TAF2, IKZF4, DNMT3A, BRD2, KAT2B, CEBPB, SMAD7, KLF11, LOC100154071, ZNF667, RBMX, FOXP1, RGS14, SIRT2, SOD2, SFRP5, ATF6, RNF8, ATXN1, RPS6KA3, PAPOLA, ETS1, HBZ, IRF1, PHEX, ZBTB1, RNF111, BMPR1A
		*GO:2001141	Regulation of RNA biosynthetic process					92	3,29E-04	MORF4L1, COPS5, STAT5A, LOC100158121, CCNT1, NAA15, NR2E3, LOC100627422, DDR2, CITED2, CDC45, APP, MDFIC, WWP1, RARB, KDM5B, FANCA, LOC100153329, CHUK, SAMD4A, GNL3, TBL1XR1, CCNK, LDB1, ARID5B, SAA3, TLE4, TBR1, INHBA, HHEX, EP300, FANCD2, MED17, UCHL5, TFAP2A, TNFAIP3, SBNO1, MTDH, UBE3A, ZBTB11, SAV1, NFKBIA, KEAP1, NR3C1, NEO1, MYBL2, CCL4, NR2C2, TFAM, HNRNPK, MAP3K2, AGO1, CHD2, LEO1, MYCBP, KDM3A, PHF20L1, HELLS, BCL9, CHD3, TRAF3, CEBPA, ZNF398, TAF2, IKZF4, DNMT3A, BRD2, KAT2B, CEBPB, SMAD7, KLF11, ZNF667, LOC100154071, RBMX, FOXP1, RGS14, SIRT2, SOD2, SFRP5, ATF6, RNF8, ATXN1, RPS6KA3, ETS1, HBZ, IRF1, PHEX, ZBTB1, RNF111, BMPR1A
			*GO:1903506					91	4,60E-04	MORF4L1, COPS5, STAT5A, LOC100158121, CCNT1, NAA15, NR2E3, LOC100627422, DDR2, CITED2, CDC45, APP, MDFIC, WWP1, RARB, KDM5B, FANCA, LOC100153329, CHUK, SAMD4A, GNL3, TBL1XR1, CCNK, LDB1, ARID5B, SAA3, TLE4, TBR1, INHBA, HHEX, EP300, FANCD2, MED17, UCHL5, TFAP2A, TNFAIP3, SBNO1, MTDH, UBE3A, ZBTB11, SAV1, NFKBIA, KEAP1, NR3C1, NEO1, MYBL2, NR2C2, TFAM, HNRNPK, MAP3K2, AGO1, CHD2, LEO1, MYCBP, KDM3A, PHF20L1, HELLS, BCL9, CHD3, TRAF3, CEBPA, ZNF398, TAF2, IKZF4, DNMT3A, BRD2, KAT2B, CEBPB, SMAD7, KLF11, ZNF667, LOC100154071, RBMX, FOXP1, RGS14, SIRT2, SOD2, SFRP5, ATF6, RNF8, ATXN1, RPS6KA3, ETS1, HBZ, IRF1, PHEX, ZBTB1, RNF111, BMPR1A
				*GO:0006355	Regulation of transcription, DNA-templated			91	4,36E-04	MORF4L1, COPS5, STAT5A, LOC100158121, CCNT1, NAA15, NR2E3, LOC100627422, DDR2, CITED2, CDC45, APP, MDFIC, WWP1, RARB, KDM5B, FANCA, LOC100153329, CHUK, SAMD4A, GNL3, TBL1XR1, CCNK, LDB1, ARID5B, SAA3, TLE4, TBR1, INHBA, HHEX, EP300, FANCD2, MED17, UCHL5, TFAP2A, TNFAIP3, SBNO1, MTDH, UBE3A, ZBTB11, SAV1, NFKBIA, KEAP1, NR3C1, NEO1, MYBL2, NR2C2, TFAM, HNRNPK, MAP3K2, AGO1, CHD2, LEO1, MYCBP, KDM3A, PHF20L1, HELLS, BCL9, CHD3, TRAF3, CEBPA, ZNF398, TAF2, IKZF4, DNMT3A, BRD2, KAT2B, CEBPB, SMAD7, KLF11, ZNF667, LOC100154071, RBMX, FOXP1, RGS14, SIRT2, SOD2, SFRP5, ATF6, RNF8, ATXN1, RPS6KA3, ETS1, HBZ, IRF1, PHEX, ZBTB1, RNF111, BMPR1A
					*GO:0006357	Regulation of transcription from RNA polymerase II promoter		54	4,19E-03	COPS5, STAT5A, CCNT1, NR2E3, LOC100627422, CITED2, APP, RARB, CHUK, GNL3, TBL1XR1, CCNK, LDB1, ARID5B, TLE4, TBR1, INHBA, HHEX, EP300, MED17, TFAP2A, MTDH, UBE3A, NFKBIA, NEO1, NR3C1, MYBL2, NR2C2, HNRNPK, AGO1, LEO1, KDM3A, BCL9, CEBPA, TAF2, DNMT3A, IKZF4, BRD2, KAT2B, SMAD7, KLF11, ZNF667, RBMX, SIRT2, FOXP1, SOD2, RNF8, ATF6, RPS6KA3, ETS1, HBZ, IRF1, ZBTB1, BMPR1A
					*GO:0051090	Regulation of sequence-specific DNA binding transcription factor activity		15	2,97E-02	SFRP5, MTDH, CEBPB, EP300, FANCD2, SMAD7, SAV1, ARID5B, NFKBIA, KEAP1, TNFAIP3, FANCA, DDR2, TRAF3
**GO:0051173	Positive regulation of nitrogen compound metabolic process							53	3,05E-03	ABCF1, COPS5, STAT5A, CCNT1, NAA15, RPS6KB1, NR2E3, APP, MDFIC, RARB, SAMD4A, CHUK, GNL3, TBL1XR1, CCNK, LDB1, TBR1, HHEX, INHBA, EP300, MED17, TFAP2A, ASS1, UBE3A, NFKBIA, MRAP2, NR3C1, NR2C2, UHMK1, TFAM, MAP3K2, AGO1, LEO1, KDM3A, ACSL3, BCL9, CEBPA, TAF2, IKZF4, KAT2B, CEBPB, RBMX, SIRT2, FOXP1, SOD2, RNF8, ATF6, RPS6KA3, ETS1, IRF1, RNF111, BMPR1A
	*GO:0045935	Positive regulation of nucleobase-containing compound metabolic process						46	2,25E-02	COPS5, UBE3A, STAT5A, CCNT1, NAA15, NFKBIA, MRAP2, NR2E3, NR3C1, NR2C2, TFAM, APP, MDFIC, MAP3K2, AGO1, LEO1, KDM3A, RARB, CHUK, GNL3, BCL9, CEBPA, TAF2, TBL1XR1, IKZF4, CCNK, KAT2B, CEBPB, LDB1, TBR1, RBMX, FOXP1, SIRT2, RNF8, ATF6, HHEX, INHBA, RPS6KA3, EP300, ETS1, MED17, IRF1, TFAP2A, BMPR1A, RNF111
		*GO:0051254	Positive regulation of RNA metabolic process					44	2,98E-03	COPS5, UBE3A, STAT5A, CCNT1, NAA15, NFKBIA, NR2E3, NR3C1, NR2C2, TFAM, APP, MDFIC, MAP3K2, AGO1, LEO1, KDM3A, RARB, CHUK, GNL3, BCL9, CEBPA, TAF2, TBL1XR1, IKZF4, CCNK, KAT2B, CEBPB, LDB1, TBR1, RBMX, FOXP1, SIRT2, ATF6, HHEX, INHBA, RPS6KA3, EP300, ETS1, MED17, IRF1, TFAP2A, BMPR1A, RNF111
			*GO:1902680	Positive regulation of RNA biosynthetic process				44	1,41E-03	COPS5, UBE3A, STAT5A, CCNT1, NAA15, NFKBIA, NR2E3, NR3C1, NR2C2, TFAM, APP, MDFIC, MAP3K2, AGO1, LEO1, KDM3A, RARB, CHUK, GNL3, BCL9, CEBPA, TAF2, TBL1XR1, IKZF4, CCNK, KAT2B, CEBPB, LDB1, TBR1, RBMX, FOXP1, SIRT2, ATF6, HHEX, INHBA, RPS6KA3, EP300, ETS1, MED17, IRF1, TFAP2A, BMPR1A, RNF111
				*GO:1903508	Positive regulation of nucleic acid-templated transcription			43	2,18E-03	COPS5, UBE3A, STAT5A, CCNT1, NAA15, NFKBIA, NR2E3, NR3C1, NR2C2, TFAM, APP, MAP3K2, AGO1, LEO1, KDM3A, RARB, CHUK, GNL3, BCL9, CEBPA, TAF2, IKZF4, TBL1XR1, CCNK, KAT2B, CEBPB, LDB1, TBR1, RBMX, FOXP1, SIRT2, ATF6, HHEX, INHBA, RPS6KA3, EP300, ETS1, MED17, IRF1, TFAP2A, BMPR1A, RNF111
					*GO:0045893	Positive regulation of transcription, DNA-templated		43	2,18E-03	COPS5, UBE3A, STAT5A, CCNT1, NAA15, NFKBIA, NR2E3, NR3C1, NR2C2, TFAM, APP, MAP3K2, AGO1, LEO1, KDM3A, RARB, CHUK, GNL3, BCL9, CEBPA, TAF2, IKZF4, TBL1XR1, CCNK, KAT2B, CEBPB, LDB1, TBR1, RBMX, FOXP1, SIRT2, ATF6, HHEX, INHBA, RPS6KA3, EP300, ETS1, MED17, IRF1, TFAP2A, BMPR1A, RNF111
						*GO:0045944	Positive regulation of transcription from RNA polymerase II promoter	36	4,39E-04	COPS5, UBE3A, STAT5A, CCNT1, NFKBIA, NR2E3, NR3C1, NR2C2, APP, AGO1, LEO1, KDM3A, RARB, CHUK, GNL3, BCL9, TAF2, IKZF4, TBL1XR1, CCNK, KAT2B, LDB1, TBR1, RBMX, FOXP1, SIRT2, ATF6, HHEX, INHBA, RPS6KA3, EP300, ETS1, MED17, IRF1, TFAP2A, BMPR1A
**GO:0051172	Negative regulation of nitrogen compound metabolic process							46	1,85E-03	MORF4L1, MTDH, CPEB2, CPEB4, CCNT1, LOC100158121, SYNCRIP, NR2E3, LOC100627422, CCL4, ZFP36L1, CDC45, LPCAT1, MDFIC, WWP1, AGO1, RARB, KDM5B, LOC100153329, TRIP12, SAMD4A, HELLS, CEBPA, TBL1XR1, DNMT3A, IKZF4, SMAD7, LDB1, KLF11, FBP1, LOC100154071, SAA3, TLE4, FXR2, FOXP1, SIRT2, ATXN1, RNF8, EP300, EIF2S1, PARPBP, HBZ, IRF1, TFAP2A, GRB7, ZBTB1
	*GO:0045934	Negative regulation of nucleobase-containing compound metabolic process						36	4,22E-02	MORF4L1, MTDH, LOC100158121, CCNT1, NR2E3, CCL4, LOC100627422, CDC45, MDFIC, WWP1, RARB, KDM5B, LOC100153329, TRIP12, HELLS, CEBPA, IKZF4, DNMT3A, TBL1XR1, SMAD7, LDB1, KLF11, FBP1, LOC100154071, SAA3, TLE4, FOXP1, SIRT2, ATXN1, RNF8, EP300, PARPBP, HBZ, IRF1, TFAP2A, ZBTB1
		*GO:0051253	Negative regulation of RNA metabolic process					33	4,13E-02	MORF4L1, MTDH, LOC100158121, CCNT1, NR2E3, CCL4, LOC100627422, CDC45, MDFIC, WWP1, RARB, KDM5B, LOC100153329, HELLS, CEBPA, IKZF4, DNMT3A, TBL1XR1, SMAD7, LDB1, KLF11, LOC100154071, SAA3, TLE4, FOXP1, SIRT2, ATXN1, RNF8, EP300, HBZ, IRF1, TFAP2A, ZBTB1
			*GO:1902679	Negative regulation of RNA biosynthetic process				32	3,99E-02	MORF4L1, MTDH, LOC100158121, NR2E3, CCL4, LOC100627422, CDC45, MDFIC, WWP1, RARB, KDM5B, LOC100153329, HELLS, CEBPA, IKZF4, DNMT3A, TBL1XR1, SMAD7, LDB1, KLF11, LOC100154071, SAA3, TLE4, FOXP1, SIRT2, ATXN1, RNF8, EP300, HBZ, IRF1, TFAP2A, ZBTB1
				*GO:1903507	Negative regulation of nucleic acid-templated transcription			31	5,16E-02	MORF4L1, MTDH, LOC100158121, NR2E3, LOC100627422, CDC45, MDFIC, WWP1, RARB, KDM5B, LOC100153329, HELLS, CEBPA, IKZF4, DNMT3A, TBL1XR1, SMAD7, LDB1, KLF11, LOC100154071, SAA3, TLE4, FOXP1, SIRT2, ATXN1, RNF8, EP300, HBZ, IRF1, TFAP2A, ZBTB1
					*GO:0045892	Negative regulation of transcription, DNA-templated		31	4,89E-02	MORF4L1, MTDH, LOC100158121, NR2E3, LOC100627422, CDC45, MDFIC, WWP1, RARB, KDM5B, LOC100153329, HELLS, CEBPA, IKZF4, DNMT3A, TBL1XR1, SMAD7, LDB1, KLF11, LOC100154071, SAA3, TLE4, FOXP1, SIRT2, ATXN1, RNF8, EP300, HBZ, IRF1, TFAP2A, ZBTB1

**GO:0016070**	**RNA metabolic process**			**117**	**2,63E-03**	**MORF4L1, STAT5A, LOC100158121, NAA15, FOXO3, NR2E3, NFXL1, SART3, CITED2, APP, MDFIC, WWP1, RARB, ZNF445, SAMD4A, GNL3, TBR1, HHEX, EP300, MED17, TFAP2A, TNFAIP3, NFKBIA, KEAP1, MYBL2, CCL4, NR2C2, UHMK1, DDX46, HNRNPK, LEO1, HELLS, TRAF3, TAF2, ZNF398, IKZF4, DNMT3A, SMAD7, TAF5, KLF11, MPHOSPH10, LOC100154071, RBMX, FOXP1, ATM, RNF8, ATF6, SFRP5, ETS1, WDR3, PABPC1L, PHEX, COPS5, CCNT1, ZNF518A, LOC100627422, DDR2, CDC45, KDM5B, FANCA, LOC100621389, CHUK, FTSJ3, LOC100153329, TBL1XR1, SNAPC5, CCNK, ARID5B, LDB1, SAA3, TLE4, TARS, INHBA, FANCD2, HIPK3, UCHL5, CUX2, KIAA1429, SBNO1, MTDH, UBE3A, ZBTB11, SAV1, NR3C1, NEO1, VARS, ZFP36L1, TFAM, SF3B1, MAP3K2, AGO1, CHD2, MYCBP, KDM3A, PHF20L1, BCL9, CHD3, CEBPA, BRD2, KAT2B, CEBPB, ZNF667, SIRT2, RGS14, SOD2, ATXN1, RPS6KA3, PAPOLA, HBZ, IRF1, NOP58, NOP56, ZBTB1, RNF111, BMPR1A**
*GO:0032774	RNA biosynthetic process			101	1,04E-04	MORF4L1, STAT5A, LOC100158121, NAA15, FOXO3, NR2E3, NFXL1, CITED2, APP, MDFIC, WWP1, RARB, ZNF445, SAMD4A, GNL3, TBR1, HHEX, EP300, MED17, TFAP2A, TNFAIP3, NFKBIA, KEAP1, MYBL2, CCL4, NR2C2, HNRNPK, LEO1, HELLS, TRAF3, ZNF398, TAF2, IKZF4, DNMT3A, SMAD7, TAF5, KLF11, LOC100154071, RBMX, FOXP1, RNF8, ATF6, SFRP5, ETS1, PHEX, COPS5, CCNT1, ZNF518A, DDR2, LOC100627422, CDC45, FANCA, KDM5B, LOC100153329, CHUK, LOC100621389, TBL1XR1, SNAPC5, CCNK, LDB1, ARID5B, SAA3, TLE4, INHBA, FANCD2, HIPK3, UCHL5, CUX2, SBNO1, MTDH, UBE3A, SAV1, ZBTB11, NR3C1, NEO1, TFAM, MAP3K2, CHD2, AGO1, KDM3A, MYCBP, PHF20L1, CHD3, BCL9, CEBPA, BRD2, KAT2B, CEBPB, ZNF667, SIRT2, RGS14, SOD2, ATXN1, RPS6KA3, HBZ, IRF1, ZBTB1, RNF111, BMPR1A
	*GO:0097659	Nucleic acid-templated transcription		100	1,36E-04	MORF4L1, STAT5A, LOC100158121, NAA15, FOXO3, NR2E3, NFXL1, CITED2, APP, MDFIC, WWP1, RARB, ZNF445, SAMD4A, GNL3, TBR1, HHEX, EP300, MED17, TFAP2A, TNFAIP3, NFKBIA, KEAP1, MYBL2, NR2C2, HNRNPK, LEO1, HELLS, TRAF3, ZNF398, TAF2, IKZF4, DNMT3A, SMAD7, TAF5, KLF11, LOC100154071, RBMX, FOXP1, RNF8, ATF6, SFRP5, ETS1, PHEX, COPS5, CCNT1, ZNF518A, DDR2, LOC100627422, CDC45, FANCA, KDM5B, LOC100153329, CHUK, LOC100621389, TBL1XR1, SNAPC5, CCNK, LDB1, ARID5B, SAA3, TLE4, INHBA, FANCD2, HIPK3, UCHL5, CUX2, SBNO1, MTDH, UBE3A, SAV1, ZBTB11, NR3C1, NEO1, TFAM, MAP3K2, CHD2, AGO1, KDM3A, MYCBP, PHF20L1, CHD3, BCL9, CEBPA, BRD2, CEBPB, KAT2B, ZNF667, SIRT2, RGS14, SOD2, ATXN1, RPS6KA3, HBZ, IRF1, ZBTB1, RNF111, BMPR1A
*GO:0043631	RNA polyadenylation			5	7,77E-03	APP, PAPOLA, CCNT1, LEO1, PABPC1L
	***GO:0006378**	mRNA polyadenylation		5	6,49E-03	APP, PAPOLA, CCNT1, LEO1, PABPC1L
*GO:0031123	RNA 3'-end processing			5	7,76E-02	APP, PAPOLA, CCNT1, LEO1, PABPC1L
	*GO:0031124	mRNA 3'-end processing		5	1,08E-02	APP, PAPOLA, CCNT1, LEO1, PABPC1L
		**GO:0031440	Regulation of mRNA 3'-end processing	3	3,57E-02	PAPOLA, CCNT1, LEO1
**GO:0010556**	**Regulation of macromolecule biosynthetic process**			**110**	**1,67E-05**	**MORF4L1, STAT5A, LOC100158121, NAA15, SYNCRIP, RPS6KB1, FOXO3, NR2E3, CITED2, APP, MDFIC, WWP1, RARB, EIF2B2, SAMD4A, GNL3, TBR1, HHEX, EP300, EIF2S1, MED17, TFAP2A, TNFAIP3, ENPP1, NFKBIA, KEAP1, MYBL2, CCL4, NR2C2, UHMK1, HNRNPK, LEO1, HELLS, TRAF3, TAF2, ZNF398, IKZF4, DNMT3A, SMAD7, KLF11, LOC100154071, FXR2, RBMX, FOXP1, RNF8, ATF6, SFRP5, ETS1, PHEX, GRB7, ABCF1, SEC24A, CPEB2, COPS5, CPEB4, CCNT1, DDR2, LOC100627422, CDC45, FANCA, KDM5B, CHUK, LOC100153329, TBL1XR1, CCNK, ARID5B, LDB1, ELANE, SAA3, TLE4, EIF4G1, INHBA, FANCD2, UCHL5, SBNO1, MTDH, UBE3A, SAV1, ZBTB11, NR3C1, NEO1, ZFP36L1, TFAM, MAP3K2, AGO1, CHD2, MYCBP, KDM3A, PHF20L1, BCL9, CHD3, CEBPA, BRD2, KAT2B, CEBPB, IL9, ZNF667, SIRT2, RGS14, SOD2, ATXN1, SAMD8, RPS6KA3, HBZ, IRF1, ZBTB1, RNF111, BMPR1A**
	**GO:0010557	Positive regulation of macromolecule biosynthetic process		50	1,71E-03	ABCF1, COPS5, STAT5A, CCNT1, NAA15, RPS6KB1, NR2E3, APP, MDFIC, RARB, SAMD4A, CHUK, GNL3, TBL1XR1, CCNK, LDB1, ELANE, TBR1, HHEX, INHBA, EP300, MED17, TFAP2A, UBE3A, NFKBIA, NR3C1, NR2C2, UHMK1, TFAM, MAP3K2, AGO1, LEO1, KDM3A, BCL9, CEBPA, TAF2, IKZF4, CEBPB, KAT2B, IL9, RBMX, SIRT2, FOXP1, ATF6, RPS6KA3, ETS1, IRF1, BMPR1A, RNF111
	**GO:0010558	Negative regulation of macromolecule biosynthetic process		43	2,69E-03	MORF4L1, MTDH, ENPP1, CPEB2, CPEB4, LOC100158121, SYNCRIP, NR2E3, LOC100627422, CCL4, ZFP36L1, CDC45, MDFIC, WWP1, AGO1, RARB, KDM5B, LOC100153329, SAMD4A, HELLS, CEBPA, DNMT3A, TBL1XR1, IKZF4, SMAD7, LDB1, ELANE, KLF11, LOC100154071, SAA3, TLE4, FXR2, FOXP1, SIRT2, ATXN1, RNF8, EP300, EIF2S1, HBZ, IRF1, TFAP2A, GRB7, ZBTB1
*GO:2000112	Regulation of cellular macromolecule biosynthetic process			105	6,94E-05	MORF4L1, STAT5A, LOC100158121, NAA15, SYNCRIP, RPS6KB1, FOXO3, NR2E3, CITED2, APP, MDFIC, WWP1, RARB, EIF2B2, SAMD4A, GNL3, TBR1, HHEX, EP300, EIF2S1, MED17, TFAP2A, TNFAIP3, ENPP1, NFKBIA, KEAP1, MYBL2, NR2C2, UHMK1, HNRNPK, LEO1, HELLS, TRAF3, ZNF398, TAF2, IKZF4, DNMT3A, SMAD7, KLF11, LOC100154071, FXR2, RBMX, FOXP1, RNF8, ATF6, SFRP5, ETS1, PHEX, GRB7, ABCF1, CPEB2, COPS5, CPEB4, CCNT1, DDR2, LOC100627422, CDC45, FANCA, KDM5B, CHUK, LOC100153329, TBL1XR1, CCNK, LDB1, ARID5B, SAA3, TLE4, EIF4G1, INHBA, FANCD2, UCHL5, SBNO1, MTDH, UBE3A, SAV1, ZBTB11, NR3C1, NEO1, ZFP36L1, TFAM, MAP3K2, CHD2, AGO1, MYCBP, KDM3A, PHF20L1, CHD3, BCL9, CEBPA, BRD2, KAT2B, CEBPB, ZNF667, SIRT2, RGS14, SOD2, ATXN1, RPS6KA3, HBZ, IRF1, ZBTB1, RNF111, BMPR1A
	*GO:0031328	Positive regulation of cellular biosynthetic process		55	5,11E-04	ABCF1, COPS5, STAT5A, CCNT1, NAA15, RPS6KB1, NR2E3, APP, APOA1, MDFIC, RARB, SAMD4A, CHUK, GNL3, TBL1XR1, CCNK, LDB1, ELANE, MID1IP1, TBR1, HHEX, INHBA, EP300, MED17, TFAP2A, ASS1, UBE3A, NFKBIA, MRAP2, NR3C1, NR2C2, UHMK1, TFAM, MAP3K2, AGO1, LEO1, KDM3A, BCL9, CEBPA, TAF2, IKZF4, KAT2B, CEBPB, IL9, RBMX, FOXP1, SIRT2, SOD2, ATF6, RPS6KA3, ETS1, IRF1, RNF111, BMPR1A
	*GO:0031327	Negative regulation of cellular biosynthetic process		43	5,95E-03	MORF4L1, MTDH, ENPP1, CPEB2, CPEB4, LOC100158121, SYNCRIP, NR2E3, LOC100627422, CCL4, ZFP36L1, CDC45, MDFIC, WWP1, AGO1, RARB, KDM5B, LOC100153329, SAMD4A, HELLS, CEBPA, DNMT3A, TBL1XR1, IKZF4, SMAD7, LDB1, ELANE, KLF11, LOC100154071, SAA3, TLE4, FXR2, FOXP1, SIRT2, ATXN1, RNF8, EP300, EIF2S1, HBZ, IRF1, TFAP2A, GRB7, ZBTB1
		*GO:2000113	Negative regulation of cellular macromolecule biosynthetic process	41	4,19E-03	MORF4L1, MTDH, ENPP1, CPEB2, CPEB4, LOC100158121, SYNCRIP, NR2E3, LOC100627422, ZFP36L1, CDC45, MDFIC, WWP1, AGO1, RARB, KDM5B, LOC100153329, SAMD4A, HELLS, CEBPA, DNMT3A, TBL1XR1, IKZF4, SMAD7, LDB1, KLF11, LOC100154071, SAA3, TLE4, FXR2, FOXP1, SIRT2, ATXN1, RNF8, EP300, EIF2S1, HBZ, IRF1, TFAP2A, GRB7, ZBTB1
*GO:0009893	Positive regulation of metabolic process			83	7,06E-03	ABCF1, COPS5, STAT5A, CCNT1, NAA15, RPS6KB1, NR2E3, DDR2, TNFRSF11B, APP, APOA1, MDFIC, TGFA, MSN, RARB, KDM5B, SAMD4A, CHUK, GNL3, TBL1XR1, CCNK, ACTA1, ANP32B, LDB1, SOCS1, ELANE, SAA3, SOCS4, MID1IP1, TBR1, INHBA, HHEX, EP300, MED17, TFAP2A, CUX2, TNFAIP3, ASS1, UBE3A, NFKBIA, NR3C1, MRAP2, TNFRSF4, CCL4, DTNBP1, NR2C2, UHMK1, TFAM, TNFRSF1B, ACSL1, LPCAT1, MAP3K2, AGO1, LEO1, PIK3R5, KDM3A, ACSL3, BCL9, CEBPA, TAF2, IKZF4, KAT2B, CEBPB, SMAD7, IL9, CHI3L1, RBMX, FOXP1, SIRT2, SOD2, ATF6, RNF8, EPHA4, RPS6KA3, PRLR, ETS1, IRF1, HSPD1, SH3D19, ABL2, RNF111, BMPR1A
	*GO:0010604	Positive regulation of macromolecule metabolic process		76	1,65E-02	ABCF1, COPS5, STAT5A, CCNT1, NAA15, RPS6KB1, NR2E3, DDR2, TNFRSF11B, APP, MDFIC, TGFA, MSN, RARB, KDM5B, SAMD4A, CHUK, GNL3, TBL1XR1, CCNK, ACTA1, ANP32B, LDB1, SOCS1, ELANE, SAA3, SOCS4, TBR1, INHBA, HHEX, EP300, MED17, TFAP2A, CUX2, TNFAIP3, UBE3A, NFKBIA, NR3C1, TNFRSF4, CCL4, DTNBP1, NR2C2, UHMK1, TFAM, TNFRSF1B, ACSL1, LPCAT1, MAP3K2, AGO1, LEO1, PIK3R5, KDM3A, BCL9, CEBPA, TAF2, IKZF4, KAT2B, CEBPB, SMAD7, IL9, CHI3L1, RBMX, FOXP1, SIRT2, RNF8, ATF6, EPHA4, RPS6KA3, PRLR, ETS1, IRF1, HSPD1, SH3D19, RNF111, BMPR1A
		*GO:0031325	Positive regulation of cellular metabolic process	76	1,65E-02	ABCF1, COPS5, STAT5A, CCNT1, NAA15, RPS6KB1, NR2E3, DDR2, TNFRSF11B, APP, APOA1, MDFIC, TGFA, MSN, RARB, SAMD4A, CHUK, GNL3, TBL1XR1, CCNK, ANP32B, LDB1, SOCS1, ELANE, SAA3, SOCS4, MID1IP1, TBR1, INHBA, HHEX, EP300, MED17, TFAP2A, TNFAIP3, ASS1, UBE3A, NFKBIA, NR3C1, MRAP2, TNFRSF4, CCL4, NR2C2, UHMK1, TFAM, TNFRSF1B, ACSL1, MAP3K2, AGO1, LEO1, PIK3R5, KDM3A, BCL9, CEBPA, TAF2, IKZF4, KAT2B, CEBPB, SMAD7, IL9, CHI3L1, RBMX, FOXP1, SIRT2, SOD2, ATF6, RNF8, EPHA4, RPS6KA3, PRLR, ETS1, IRF1, HSPD1, SH3D19, RNF111, BMPR1A
**GO:0006793**	**Phosphorus metabolic process**			**81**	**7,95E-03**	**NUAK2, COPS5, CCNT1, DDR2, ST3GAL1, TPK1, TNFRSF11B, APP, APOA1, EEF2K, TGFA, ATP5O, TNIP1, ATP8, CLN3, TBL1XR1, CCNK, NUDT12, LDB1, SOCS1, PIGV, FBP1, SAA3, PKN2, OLA1, SOCS4, CDKL5, INHBA, HHEX, CKM, HIPK3, EIF2S1, DLD, TNFAIP3, PRPS2, CROT, PPP2R2A, ME1, PHLPP1, ENPP1, BCCIP, MRAP2, TNFRSF4, CCL4, DTNBP1, NR2C2, UHMK1, TNFRSF1B, ACSL1, LPCAT1, SNRK, DCLK3, PIK3R5, RPIA, INPP5B, MYOC, KAT2B, NRBF2, NCEH1, PCIF1, SMAD7, CBL, CHI3L1, MPHOSPH10, TKT, CDC25C, CDKN3, AMPD3, ATM, RGS14, SIRT2, EPHA4, PPP1R9B, CBLB, PRLR, JAK3, PIP4K2A, PHEX, ABL2, MYLK, BMPR1A**

*GO:0006796	Phosphate-containing compound metabolic process						80	1,06E-02	COPS5, NUAK2, CCNT1, DDR2, ST3GAL1, TPK1, TNFRSF11B, APP, APOA1, EEF2K, TGFA, ATP5O, TNIP1, ATP8, CLN3, TBL1XR1, CCNK, NUDT12, LDB1, SOCS1, PIGV, FBP1, SAA3, PKN2, OLA1, SOCS4, CDKL5, INHBA, HHEX, CKM, HIPK3, EIF2S1, DLD, TNFAIP3, PRPS2, CROT, PPP2R2A, ME1, PHLPP1, ENPP1, BCCIP, MRAP2, TNFRSF4, CCL4, DTNBP1, NR2C2, UHMK1, TNFRSF1B, ACSL1, LPCAT1, SNRK, DCLK3, PIK3R5, RPIA, INPP5B, MYOC, KAT2B, NRBF2, NCEH1, PCIF1, SMAD7, CBL, CHI3L1, MPHOSPH10, TKT, CDC25C, CDKN3, AMPD3, ATM, RGS14, SIRT2, EPHA4, PPP1R9B, CBLB, PRLR, JAK3, PIP4K2A, ABL2, MYLK, BMPR1A
	*GO:0016310	Phosphorylation					57	2,54E-02	COPS5, NUAK2, CCNT1, DDR2, ST3GAL1, APP, TNFRSF11B, APOA1, EEF2K, TGFA, TNIP1, CCNK, LDB1, SOCS1, FBP1, PKN2, SAA3, SOCS4, CDKL5, HHEX, INHBA, CKM, HIPK3, EIF2S1, DLD, TNFAIP3, PHLPP1, ENPP1, BCCIP, TNFRSF4, DTNBP1, CCL4, NR2C2, UHMK1, TNFRSF1B, ACSL1, SNRK, DCLK3, PIK3R5, MYOC, KAT2B, NRBF2, SMAD7, CBL, CHI3L1, ATM, SIRT2, RGS14, PPP1R9B, EPHA4, CBLB, PRLR, JAK3, PIP4K2A, ABL2, MYLK, BMPR1A
		*GO:0006468	Protein amino acid phosphorylation				49	4,29E-02	COPS5, NUAK2, CCNT1, DDR2, ST3GAL1, APP, TNFRSF11B, EEF2K, TGFA, TNIP1, CCNK, SOCS1, PKN2, SAA3, SOCS4, CDKL5, HHEX, INHBA, HIPK3, EIF2S1, TNFAIP3, PHLPP1, ENPP1, BCCIP, DTNBP1, CCL4, TNFRSF4, NR2C2, UHMK1, TNFRSF1B, ACSL1, SNRK, DCLK3, PIK3R5, MYOC, KAT2B, SMAD7, CBL, CHI3L1, SIRT2, RGS14, ATM, EPHA4, CBLB, PRLR, JAK3, ABL2, MYLK, BMPR1A
		*GO:0042325	Regulation of phosphorylation				41	9,26E-02	PHLPP1, ENPP1, COPS5, CCNT1, BCCIP, TNFRSF4, DTNBP1, CCL4, DDR2, TNFRSF11B, APP, TNFRSF1B, ACSL1, APOA1, EEF2K, TGFA, PIK3R5, TNIP1, MYOC, CCNK, KAT2B, NRBF2, SMAD7, LDB1, SOCS1, CBL, FBP1, CHI3L1, SAA3, SOCS4, RGS14, SIRT2, HHEX, INHBA, EPHA4, PPP1R9B, CBLB, PRLR, HIPK3, TNFAIP3, BMPR1A
			*GO:0043549	Regulation of kinase activity			23	5,86E-02	CCNK, KAT2B, NRBF2, LDB1, SOCS1, CCNT1, CBL, CHI3L1, SOCS4, BCCIP, DDR2, DTNBP1, RGS14, EPHA4, HHEX, CBLB, APP, ACSL1, PRLR, HIPK3, TGFA, PIK3R5, TNFAIP3
				*GO:0045859	Regulation of protein kinase activity		21	6,98E-02	CCNK, KAT2B, SOCS1, CCNT1, CBL, CHI3L1, SOCS4, BCCIP, DDR2, DTNBP1, RGS14, EPHA4, HHEX, CBLB, APP, ACSL1, PRLR, HIPK3, TGFA, PIK3R5, TNFAIP3
					*GO:0006469	Negative regulation of protein kinase activity	10	6,94E-02	HHEX, CBLB, KAT2B, HIPK3, SOCS1, CBL, SOCS4, TNFAIP3, DTNBP1, RGS14
				**GO:1904029	Regulation of cyclin-dependent protein kinase activity		6	5,65E-02	HHEX, CCNK, KAT2B, CCNT1, BCCIP, TNFAIP3
					*GO:0000079	Regulation of cyclin-dependent protein kinase activity	6	5,28E-02	HHEX, CCNK, KAT2B, CCNT1, BCCIP, TNFAIP3
			*GO:0061097	Regulation of protein tyrosine kinase activity			5	3,35E-02	APP, CBLB, CBL, TGFA, SOCS4
			*GO:0061099	Negative regulation of protein tyrosine kinase activity			3	9,97E-02	CBLB, CBL, SOCS4
			*GO:0050732	Negative regulation of peptidyl-tyrosine phosphorylation			4	5,71E-02	CBLB, SOCS1, CBL, SOCS4
**GO:0051246**	**Regulation of protein metabolic process**						**79**	5,36E-04	ABCF1, SEC24A, CPEB2, COPS5, CPEB4, STAT5A, CCNT1, SYNCRIP, RPS6KB1, FOXO3, ENPEP, DDR2, TNFRSF11B, APP, APOA1, EEF2K, TGFA, MSN, NOS2, EIF2B2, TNIP1, SAMD4A, GNL3, CCNK, ANP32B, SOCS1, ELANE, SAA3, SOCS4, EIF4G1, INHBA, HHEX, RENBP, EP300, HIPK3, EIF2S1, SERPINB1, TNFAIP3, PHLPP1, RAD23B, SSH1, ENPP1, UBE3A, NFKBIA, AZIN1, BCCIP, TNFRSF4, CCL4, DTNBP1, UHMK1, ZFP36L1, TNFRSF1B, ACSL1, LPCAT1, AGO1, PIK3R5, KDM3A, INPP5B, TRIP12, MYOC, TRAF3, KAT2B, SMAD7, CBL, IL9, CHI3L1, FXR2, RGS14, SIRT2, EPHA4, PPP1R9B, CBLB, PRLR, IRF1, HSPD1, SH3D19, GRB7, RNF111, BMPR1A
*GO:0032268	Regulation of cellular protein metabolic process						68	1,05E-02	ABCF1, CPEB2, COPS5, CPEB4, CCNT1, SYNCRIP, RPS6KB1, FOXO3, DDR2, TNFRSF11B, APP, APOA1, EEF2K, TGFA, MSN, EIF2B2, TNIP1, SAMD4A, GNL3, CCNK, ANP32B, SOCS1, SAA3, SOCS4, EIF4G1, INHBA, HHEX, RENBP, EP300, HIPK3, EIF2S1, SERPINB1, TNFAIP3, PHLPP1, RAD23B, SSH1, ENPP1, UBE3A, NFKBIA, BCCIP, TNFRSF4, DTNBP1, CCL4, UHMK1, ZFP36L1, TNFRSF1B, ACSL1, AGO1, PIK3R5, KDM3A, TRIP12, MYOC, KAT2B, SMAD7, CBL, CHI3L1, FXR2, SIRT2, RGS14, EPHA4, PPP1R9B, CBLB, PRLR, HSPD1, SH3D19, GRB7, RNF111, BMPR1A
**GO:0070887**	**Cellular response to chemical stimulus**						**76**	**2,82E-04**	**CGA, FGF7, CPEB2, COPS5, STAT5A, CPEB4, SYNCRIP, RPS6KB1, FOXO3, CITED2, APOA1, CXCR4, IL4R, MSN, NOS2, CASP1, CHUK, LY96, SOCS1, FBP1, SAA3, SOCS4, UBR1, RAD52, IFNAR1, HHEX, RAB11FIP2, EP300, GNAQ, FANCD2, MED17, RYR1, RAB14, TFAP2A, RAB13, CUX2, TNFAIP3, JAM3, AMCF-II, MTDH, SSH1, ENPP1, UBE3A, DIAPH1, CXCL2, NFKBIA, CXCL8, NR3C1, NEO1, CCL4, CCL28, TNFRSF1B, ACSL1, AGO1, KDM3A, TRAF3, CEBPA, PDK1, DNMT3A, KAT2B, MLC1, NCEH1, SMAD7, CHI3L1, ACACA, RBMX, SIRT2, SOD2, ATF6, PPP1R9B, PRLR, IRF1, JAK3, LHB, ABL2, RNF111**
**GO:0010033	Response to organic substance						76	2,91E-04	CGA, FGF7, CPEB2, COPS5, STAT5A, CPEB4, SYNCRIP, RPS6KB1, FOXO3, AQP3, CITED2, TNFRSF11B, APOA1, CXCR4, IL4R, MSN, NOS2, CASP1, CHUK, LY96, SOCS1, ELANE, SOCS4, UBR1, IFNAR1, HHEX, RAB11FIP2, EP300, GNAQ, MED17, RYR1, RAB14, CUX2, TNFAIP3, PPP2R2A, AMCF-II, ME1, MTDH, SSH1, ENPP1, UBE3A, DIAPH1, FFAR1, GDAP1, CXCL2, NFKBIA, CXCL8, ABHD2, NR3C1, NEO1, TNFRSF4, CCL4, TNFRSF1B, ACSL1, AGO1, KDM3A, TRAF3, CEBPA, DNMT3A, KAT2B, MLC1, SMAD7, CHI3L1, ACACA, RBMX, SIRT2, ATF6, PPP1R9B, RPS6KA3, PRLR, IRF1, HSPD1, JAK3, LHB, ABL2, RNF111
	*GO:0071310	Cellular response to organic substance					64	2,83E-04	CGA, FGF7, COPS5, CPEB2, STAT5A, CPEB4, SYNCRIP, RPS6KB1, FOXO3, CITED2, APOA1, CXCR4, IL4R, MSN, NOS2, CASP1, CHUK, LY96, SOCS1, SOCS4, UBR1, IFNAR1, HHEX, RAB11FIP2, EP300, MED17, RYR1, RAB14, CUX2, TNFAIP3, AMCF-II, MTDH, ENPP1, SSH1, DIAPH1, UBE3A, CXCL2, NFKBIA, CXCL8, NR3C1, NEO1, CCL4, TNFRSF1B, ACSL1, AGO1, KDM3A, TRAF3, CEBPA, DNMT3A, KAT2B, MLC1, SMAD7, ACACA, CHI3L1, RBMX, SIRT2, ATF6, PPP1R9B, PRLR, IRF1, JAK3, LHB, ABL2, RNF111
		*GO:0033993	Response to lipid				27	6,34E-04	AMCF-II, MTDH, UBE3A, CXCL2, FFAR1, GDAP1, CXCL8, NFKBIA, ABHD2, NR3C1, TNFRSF4, AQP3, TNFRSF11B, TNFRSF1B, KDM3A, NOS2, MSN, CASP1, MLC1, LY96, ELANE, IFNAR1, RPS6KA3, EP300, MED17, TNFAIP3, ABL2
			*GO:0071396	Cellular response to lipid			14	9,36E-02	MTDH, MLC1, LY96, UBE3A, NFKBIA, NR3C1, TNFRSF1B, EP300, MED17, KDM3A, NOS2, MSN, TNFAIP3, ABL2
		*GO:0034097	Response to cytokine				22	3,97E-02	AMCF-II, STAT5A, SOCS1, CXCL2, CHI3L1, SYNCRIP, CXCL8, SOCS4, FOXO3, CCL4, RBMX, IFNAR1, APOA1, ACSL1, PRLR, CXCR4, IL4R, IRF1, NOS2, JAK3, CHUK, TRAF3
			*GO:0071345	Cellular response to cytokine stimulus			22	1,06E-02	AMCF-II, STAT5A, SOCS1, CXCL2, CHI3L1, SYNCRIP, CXCL8, SOCS4, FOXO3, CCL4, RBMX, IFNAR1, APOA1, ACSL1, PRLR, CXCR4, IL4R, IRF1, NOS2, JAK3, CHUK, TRAF3
		*GO:0010243	Response to organonitrogen compound				17	7,98E-02	DNMT3A, KAT2B, SSH1, CPEB2, ENPP1, DIAPH1, CPEB4, SOCS1, NFKBIA, RPS6KB1, UBR1, SIRT2, PPP1R9B, RYR1, CASP1, TNFAIP3, PPP2R2A
		*GO:0032870	Cellular response to hormone stimulus				16	9,35E-02	CGA, KAT2B, CPEB2, ENPP1, UBE3A, STAT5A, SOCS1, ACACA, RPS6KB1, NR3C1, ACSL1, EP300, PRLR, MED17, KDM3A, LHB
			**GO:0009755	Hormone-mediated signaling pathway			9	4,55E-02	EP300, ACSL1, PRLR, UBE3A, STAT5A, MED17, KDM3A, NR3C1, LHB
		*GO:0071407	Cellular response to organic cyclic compound				15	4,74E-02	CEBPA, MLC1, SSH1, UBE3A, DIAPH1, RPS6KB1, NR3C1, SIRT2, PPP1R9B, EP300, MED17, AGO1, RYR1, KDM3A, MSN
*GO:1901700	Response to oxygen-containing compound						39	2,04E-03	AMCF-II, MTDH, ENPP1, SSH1, CPEB2, CPEB4, CXCL2, FFAR1, GDAP1, CXCL8, NFKBIA, ABHD2, RPS6KB1, TNFRSF4, AQP3, TNFRSF1B, TNFRSF11B, NOS2, MSN, CASP1, DNMT3A, KAT2B, MLC1, STX2, LY96, ELANE, SOCS1, ACACA, APTX, UBR1, SIRT2, IFNAR1, SOD2, RAB11FIP2, RPS6KA3, GNAQ, TXNRD1, TNFAIP3, ABL2
	*GO:1901701	Cellular response to oxygen-containing compound					23	6,52E-02	DNMT3A, KAT2B, MTDH, MLC1, SSH1, ENPP1, CPEB2, LY96, CPEB4, SOCS1, ACACA, NFKBIA, RPS6KB1, UBR1, SIRT2, SOD2, RAB11FIP2, TNFRSF1B, GNAQ, NOS2, MSN, TNFAIP3, ABL2
*GO:1901699	Cellular response to nitrogen compound						15	7,37E-02	DNMT3A, PPP1R9B, KAT2B, SSH1, GNAQ, ENPP1, CPEB2, DIAPH1, CPEB4, SOCS1, AGO1, RYR1, RPS6KB1, UBR1, SIRT2
	GO:0071417	Cellular response to organonitrogen compound					13	7,06E-02	DNMT3A, PPP1R9B, KAT2B, SSH1, ENPP1, CPEB2, DIAPH1, CPEB4, SOCS1, RYR1, RPS6KB1, UBR1, SIRT2
**GO:0044085**	**Cellular component biogenesis**						**72**	**7,72E-02**	**ABCF1, TLN1, ATL2, ATL3, SART3, WHAMM, TRIM4, CDC45, APP, APOA1, MSN, WDR35, FTSJ3, LOC100621389, GNL3, ABCE1, STX1A, STX2, ACTA1, ANP32B, LDB1, FBP1, PKN2, DECR1, MID1IP1, RAD52, TMEM67, ARPC1B, RAB14, RAB13, CUX2, SNAP29, ME1, CALY, SSH1, DIAPH1, GPM6B, ARPC5, MYBL2, SF3B1, RPS27, ATXN2L, DNAAF1, AGO1, DNAJA4, ACSL3, MYOC, HELLS, HSD17B8, LOC100156127, SELP, BRD2, MLC1, SMAD7, ACACA, MPHOSPH10, ARFIP1, DENR, FOXP1, SIRT2, NCKAP1, SAMD8, PRLR, CABIN1, WDR3, NOP58, DNAJB1, NOP56, PIP4K2A, ZBTB1, GRB7, TBC1D20**
**GO:0009892**	**Negative regulation of metabolic process**						**67**	**5,54E-03**	**MORF4L1, CPEB2, CPEB4, CCNT1, LOC100158121, SYNCRIP, NR2E3, LOC100627422, CDC45, APP, MDFIC, WWP1, NOS2, RARB, KDM5B, TNIP1, LOC100153329, SAMD4A, TBL1XR1, LDB1, SOCS1, ELANE, FBP1, SAA3, TLE4, SOCS4, HHEX, RENBP, EP300, HIPK3, EIF2S1, SERPINB1, TFAP2A, TNFAIP3, MTDH, ENPP1, AZIN1, DTNBP1, CCL4, ZFP36L1, LPCAT1, AGO1, KDM3A, TRIP12, HELLS, CEBPA, DNMT3A, IKZF4, KAT2B, PCIF1, SMAD7, CBL, KLF11, LOC100154071, MPHOSPH10, FXR2, FOXP1, SIRT2, RGS14, RNF8, ATXN1, CBLB, PARPBP, HBZ, IRF1, ZBTB1, GRB7**

*GO:0031324	Negative regulation of cellular metabolic process						64	5,48E-03	MORF4L1, CPEB2, CPEB4, CCNT1, LOC100158121, SYNCRIP, NR2E3, LOC100627422, CDC45, APP, MDFIC, WWP1, RARB, KDM5B, TNIP1, LOC100153329, SAMD4A, TBL1XR1, LDB1, SOCS1, ELANE, FBP1, SAA3, TLE4, SOCS4, HHEX, RENBP, EP300, HIPK3, EIF2S1, SERPINB1, TFAP2A, TNFAIP3, MTDH, ENPP1, DTNBP1, CCL4, ZFP36L1, AGO1, KDM3A, TRIP12, HELLS, CEBPA, DNMT3A, IKZF4, KAT2B, PCIF1, SMAD7, CBL, KLF11, LOC100154071, MPHOSPH10, FXR2, FOXP1, SIRT2, RGS14, RNF8, ATXN1, CBLB, PARPBP, HBZ, IRF1, ZBTB1, GRB7
	*GO:0032269	Negative regulation of cellular protein metabolic process					28	5,78E-02	CPEB2, ENPP1, CPEB4, SYNCRIP, DTNBP1, ZFP36L1, APP, AGO1, KDM3A, TNIP1, SAMD4A, TRIP12, KAT2B, SMAD7, SOCS1, CBL, SOCS4, FXR2, RGS14, SIRT2, HHEX, RENBP, CBLB, HIPK3, EIF2S1, SERPINB1, TNFAIP3, GRB7
*GO:0010605	Negative regulation of macromolecule metabolic process						63	4,06E-03	MORF4L1, CPEB2, CPEB4, CCNT1, LOC100158121, SYNCRIP, NR2E3, LOC100627422, CDC45, APP, MDFIC, WWP1, NOS2, RARB, KDM5B, TNIP1, LOC100153329, SAMD4A, TBL1XR1, LDB1, SOCS1, ELANE, SAA3, TLE4, SOCS4, HHEX, RENBP, EP300, HIPK3, EIF2S1, SERPINB1, TFAP2A, TNFAIP3, MTDH, ENPP1, AZIN1, DTNBP1, CCL4, ZFP36L1, AGO1, KDM3A, TRIP12, HELLS, CEBPA, DNMT3A, IKZF4, KAT2B, SMAD7, CBL, KLF11, LOC100154071, FXR2, FOXP1, SIRT2, RGS14, RNF8, ATXN1, CBLB, PARPBP, HBZ, IRF1, ZBTB1, GRB7
	*GO:0051248	Negative regulation of protein metabolic process					31	2,84E-02	CPEB2, ENPP1, CPEB4, SYNCRIP, AZIN1, DTNBP1, ZFP36L1, APP, AGO1, KDM3A, NOS2, TNIP1, SAMD4A, TRIP12, KAT2B, SMAD7, ELANE, SOCS1, CBL, SOCS4, FXR2, RGS14, SIRT2, HHEX, RENBP, CBLB, HIPK3, EIF2S1, SERPINB1, TNFAIP3, GRB7
**GO:0007166**	**Cell surface receptor signaling pathway**						**64**	**5,65E-02**	**FGF7, STAT5A, RPS6KB1, FOXO3, CITED2, APP, APOA1, CXCR4, IL4R, TGFA, INSR, TBL1XR1, STX1A, LY96, LDB1, ARID5B, SOCS1, TLE4, SOCS4, PTPRU, IFNAR1, HHEX, INHBA, GNAQ, RAB14, CHSY1, CUX2, TNFAIP3, AMCF-II, ITGAL, MTDH, ENPP1, PTCHD1, CD247, CXCL2, NFKBIA, CXCL8, NEO1, GCLM, CCL4, PEA15, IL17D, TNFRSF1B, ACSL1, ITGB7, MYOC, BCL9, TRAF3, CEBPA, NCEH1, SMAD7, CBL, SIRT2, SFRP5, CBLB, RNF115, PRLR, PLN, IRF1, JAK3, CD79A, ABL2, RNF111, BMPR1A**
*GO:0007167	Enzyme linked receptor protein signaling pathway						26	5,33E-02	FGF7, ENPP1, RPS6KB1, NEO1, CITED2, APP, TGFA, INSR, MYOC, NCEH1, SMAD7, ARID5B, SOCS1, CBL, SOCS4, SIRT2, INHBA, HHEX, CBLB, RNF115, PRLR, RAB14, JAK3, ABL2, RNF111, BMPR1A
	*GO:0007169	Transmembrane receptor protein tyrosine kinase signaling pathway					19	2,45E-02	FGF7, ENPP1, ARID5B, SOCS1, CBL, SOCS4, RPS6KB1, SIRT2, HHEX, CBLB, APP, RNF115, PRLR, RAB14, TGFA, JAK3, INSR, ABL2, MYOC
		*GO:0038127	ERBB signaling pathway				7	1,88E-02	APP, CBLB, RNF115, CBL, TGFA, SOCS4, MYOC
			**GO:1901184	Regulation of ERBB signaling pathway			6	3,95E-03	APP, CBLB, RNF115, CBL, TGFA, SOCS4
				*GO:1901185	Negative regulation of ERBB signaling pathway		4	7,48E-03	CBLB, RNF115, CBL, SOCS4
			*GO:0007173	Epidermal growth factor receptor signaling pathway			6	3,40E-02	APP, CBLB, RNF115, CBL, TGFA, SOCS4
				**GO:0042058	Regulation of epidermal growth factor receptor signaling pathway		6	2,81E-03	APP, CBLB, RNF115, CBL, TGFA, SOCS4
					*GO:0042059	Negative regulation of epidermal growth factor receptor signaling pathway	4	7,48E-03	CBLB, RNF115, CBL, SOCS4
	*GO:0019221	Cytokine-mediated signaling pathway					17	2,63E-02	AMCF-II, STAT5A, SOCS1, CXCL2, CXCL8, SOCS4, FOXO3, CCL4, IFNAR1, APOA1, ACSL1, PRLR, CXCR4, IL4R, IRF1, JAK3, TRAF3
		*GO:0070098	Chemokine-mediated signaling pathway				5	7,23E-02	AMCF-II, CXCR4, CXCL2, CXCL8, CCL4
**GO:0043933**	**Macromolecular complex subunit organization**						**63**	**8,96E-03**	**MORF4L1, ATL2, ATL3, LOC100158121, SART3, DDR2, WHAMM, TRIM4, CDC45, APOA1, MSN, LOC100153329, LOC100621389, TBL1XR1, ABCE1, STX1A, ANP32B, STX2, LDB1, FBP1, DECR1, MID1IP1, RAD52, ARPC1B, EP300, KDM2A, SMARCA1, ME1, SNAP29, CALY, DIAPH1, ARPC5, NR3C1, SF3B1, RPS27, ATXN2L, AGO1, LEO1, KDM3A, ACSL3, TRIP12, HELLS, HSD17B8, CHD3, LOC100156127, DNMT3A, SELP, BRD2, KAT2B, MLC1, LOC100154071, ACACA, ARFIP1, DENR, ATM, SIRT2, NCKAP1, RNF8, PRLR, CABIN1, ZBTB1, GRB7, TBC1D20**
*GO:0006325	Chromatin organization						27	2,81E-03	MORF4L1, LOC100158121, NR3C1, CDC45, LEO1, KDM3A, LOC100153329, LOC100621389, HELLS, TRIP12, CHD3, LOC100156127, DNMT3A, TBL1XR1, BRD2, KAT2B, ANP32B, LDB1, LOC100154071, SIRT2, ATM, RNF8, EP300, KDM2A, CABIN1, SMARCA1, ZBTB1
	*GO:0016569	Covalent chromatin modification					15	6,43E-02	MORF4L1, TBL1XR1, DNMT3A, KAT2B, LDB1, NR3C1, SIRT2, ATM, RNF8, EP300, KDM2A, LEO1, KDM3A, HELLS, TRIP12
*GO:0065003	Macromolecular complex assembly						43	4,79E-02	ME1, SNAP29, CALY, ATL2, DIAPH1, ATL3, ARPC5, SART3, WHAMM, TRIM4, SF3B1, RPS27, CDC45, APOA1, ATXN2L, AGO1, MSN, ACSL3, LOC100621389, HELLS, HSD17B8, LOC100156127, SELP, BRD2, STX1A, MLC1, STX2, ANP32B, FBP1, ACACA, DECR1, MID1IP1, ARFIP1, DENR, RAD52, SIRT2, NCKAP1, ARPC1B, PRLR, CABIN1, GRB7, ZBTB1, TBC1D20
	*GO:0051262	Protein tetramerization					6	8,12E-02	ME1, ACACA, FBP1, DECR1, LOC100621389, HSD17B8
**GO:0009605**	**Response to external stimulus**						**58**	**2,53E-02**	**NUAK2, STAT5A, TNFRSF11B, APP, APOA1, CXCR4, IL4R, SLC22A3, NOS2, CASP1, FANCA, TNIP1, TBL1XR1, STX2, LY96, ELANE, SAA3, CHST3, PPP1CC, TBR1, PROC, IFNAR1, GNAQ, FANCD2, CLIC5, EIF2S1, RAB14, RAB13, TNFAIP3, JAM3, AMCF-II, PHLPP1, MTDH, CXCL2, ENPP4, NFKBIA, CXCL8, NEO1, TNFRSF4, CCL28, CCL4, TNFRSF1B, MAP3K2, KLKB1, SERPINB10, TRAF3, SELP, CEBPE, CHI3L1, LGALS8, FXR2, FOXP1, SIRT2, EPHA4, RDH11, RPS6KA3, ETS1, IRF1**
**GO:0009607	Response to biotic stimulus						28	6,31E-02	AMCF-II, MTDH, CXCL2, CXCL8, NFKBIA, TNFRSF4, TNFRSF11B, TNFRSF1B, IL4R, SERPINB10, NOS2, CASP1, TRAF3, CEBPE, LY96, ELANE, LGALS8, CHST3, FXR2, SIRT2, IFNAR1, RPS6KA3, EIF2S1, RAB14, IRF1, HSPD1. TNFAIP3. TMCO1
	*GO:0071216	Cellular response to biotic stimulus					8	7,09E-02	TNFRSF1B, MTDH, LY96, NFKBIA, NOS2, TNFAIP3, TMCO1, SIRT2
		*GO:0009617	Response to bacterium				18	1,63E-02	AMCF-II, MTDH, CEBPE, LY96, ELANE, CXCL2, NFKBIA, CXCL8, TNFRSF4, SIRT2, IFNAR1, TNFRSF11B, TNFRSF1B, RPS6KA3, RAB14, NOS2, CASP1, TNFAIP3
			*GO:0002237	Response to molecule of bacterial origin			16	7,28E-04	AMCF-II, MTDH, LY96, ELANE, CXCL2, NFKBIA, CXCL8, TNFRSF4, SIRT2, IFNAR1, TNFRSF1B, RPS6KA3, TNFRSF11B, NOS2, CASP1, TNFAIP3
				*GO:0071219	Cellular response to molecule of bacterial origin		7	8,03E-02	TNFRSF1B, MTDH, LY96, NFKBIA, NOS2, TNFAIP3, SIRT2
					*GO:0032496	Response to lipopolysaccharide	15	1,17E-03	AMCF-II, MTDH, LY96, ELANE, CXCL2, NFKBIA, CXCL8, TNFRSF4, IFNAR1, TNFRSF1B, RPS6KA3, TNFRSF11B, NOS2, CASP1, TNFAIP3
**GO:0065009**	**Regulation of molecular function**						**57**	**9,58E-02**	**COPS5, CCNT1, DDR2, TBC1D16, APP, APOA1, TGFA, NQO1, FANCA, CCNK, SCN2B, ANP32B, LDB1, ARID5B, SOCS1, SOCS4, MID1IP1, HHEX, RENBP, SGSM1, EP300, GNAQ, FANCD2, HIPK3, SERPINB1, TNFAIP3, MTDH, SAV1, NFKBIA, BCCIP, KEAP1, DTNBP1, GCLM, CCL4, ACSL1, PIK3R5, TRAF3, KAT2B, NRBF2, CEBPB, PCIF1, SMAD7, CBL, MPHOSPH10, CHI3L1, SIRT2, RGS14, SOD2, SFRP5, EPHA4, CBLB, PRLR, PLN, HSPD1, ABL2, CSN2**
*GO:0043086	Negative regulation of catalytic activity						20	7,73E-02	KAT2B, PCIF1, SMAD7, SOCS1, CBL, MPHOSPH10, SOCS4, DTNBP1, RGS14, HHEX, CBLB, APP, RENBP, APOA1, HIPK3, PLN, SERPINB1, TNFAIP3, NQO1, CSN2
	GO:0051348	Negative regulation of transferase activity					11	6,98E-02	HHEX, CBLB, KAT2B, SMAD7, HIPK3, SOCS1, CBL, SOCS4, TNFAIP3, DTNBP1, RGS14
*GO:0010469	Regulation of receptor activity						6	7,24E-02	APP, CBLB, PLN, CBL, TGFA, SOCS4
	*GO:0007176	Regulation of epidermal growth factor-activated receptor activity					5	8,47E-04	APP, CBLB, CBL, TGFA, SOCS4
		*GO:0007175	Negative regulation of epidermal growth factor-activated receptor activity				3	2,01E-02	CBLB, CBL, SOCS4
**GO:1902589**	**Single-organism organelle organization**						**52**	**5,08E-02**	**TLN1, NUAK2, SLC6A1, PDLIM3, WHAMM, APOA1, CAMSAP2, SLC22A3, MCOLN1, WDR35, STX1A, ACTA1, STX2, CHST3, MID1IP1, TACC2, TMEM67, ARPC1B, SPATA18, EIF2S1, MCMBP, JAM3, ANKFY1, SNAP29, SSH1, DIAPH1, GDAP1, EEA1, ARPC5, MYBL2, DTNBP1, TPM1, NR2C2, DNAAF1, SERPINB10, MYOC, IQSEC2, CHD3, PDK1, MAP1A, ARFIP1, DENR, RGS14, ATM, FOXP1, NCKAP1, PPP1R9B, CEP68, SYNM, TBC1D20, ABL2, GFRA2**
**GO:0042592**	**Homeostatic process**						**51**	**8,14E-03**	**SEC24A, STAT5A, FOXO3, AQP3, CITED2, APP, APOA1, SLC22A5, GNL3, CLN3, SIT1, SCN2B, LDB1, INHBA, DCLRE1A, RAB11FIP2, RHCG, TNFSF13B, DLD, RYR1, TXNRD1, TNFAIP3, JAM3, TMCO1, AMCF-II, ENPP1, DIAPH1, TMX4, FFAR1, NEO1, MRAP2, GCLM, CCL28, LPCAT1, SLC31A2, MYOC, ETFA, CEBPA, KLF13, ACACA, AMPD3, ATM, SOD2, ABCG8, ETS1, PYGL, PLN, HBZ, JAK3, TBC1D20, ABL2**
*GO:0048872	Homeostasis of number of cells						16	3,79E-04	AMCF-II, SIT1, KLF13, LDB1, STAT5A, FOXO3, AMPD3, CITED2, INHBA, TNFSF13B, ETS1, HBZ, SLC22A5, JAK3, TNFAIP3, JAM3
	*GO:0002262	Myeloid cell homeostasis					10	3,58E-03	INHBA, ETS1, KLF13, LDB1, STAT5A, HBZ, FOXO3, AMPD3, JAM3, CITED2
		*GO:0034101	Erythrocyte homeostasis				9	4,09E-03	INHBA, ETS1, KLF13, LDB1, STAT5A, HBZ, FOXO3, AMPD3, CITED2
	*GO:0001776	Leukocyte homeostasis					7	9,94E-03	AMCF-II, SIT1, TNFSF13B, STAT5A, JAK3, TNFAIP3, JAM3

		*GO:0002260	Lymphocyte homeostasis						5	5,76E-02	SIT1, TNFSF13B, STAT5A, JAK3, TNFAIP3
**GO:0048584**	**Positive regulation of response to stimulus**								**50**	**7,47E-02**	**MASP1, STAT5A, CITED2, TNFRSF11B, APOA1, IL4R, TGFA, CASP1, TNIP1, STX1A, LY96, SOCS1, ELANE, SAA3, HHEX, INHBA, TNFSF13B, CHSY1, CUX2, TNFAIP3, AMCF-II, MTDH, UBE3A, CXCL2, ENPP4, CD247, NFKBIA, CXCL8, NEO1, CCL4, TNFRSF4, PEA15, TNFRSF1B, AGO1, PIK3R5, MYOC, TRAF3, SELP, CBL, CHI3L1, RNF8, EPHA4, RPS6KA3, ETS1, IRF1, HSPD1, CD79A, ZBTB1, BMPR1A, RNF111**
*GO:0031349	Positive regulation of defense response								11	6,28E-02	RPS6KA3, ETS1, LY96, STAT5A, IRF1, NFKBIA, HSPD1, TNFAIP3, TNIP1, CCL4, TRAF3
**GO:0044248**	**Cellular catabolic process**								**49**	**9,02E-03**	**RAD23B, ENPP1, UBE3A, KEAP1, ABHD2, ENPEP, ALDH1L2, UHMK1, ZFP36L1, DDHD2, CYLD, CUL5, TNFRSF1B, APOA1, WWP1, AGO1, NOS2, MSN, FBXO9, TRIP12, SAMD4A, ETFA, HECTD1, TBL1XR1, NUDT12, SMAD7, CBL, SAA3, SOCS4, DECR1, UBR1, ATM, PSMB8, CLPX, SIRT2, RNF8, CTSK, RENBP, RNF115, PYGM, PYGL, SPATA18, UCHL5, SH3D19, TNFAIP3, CROT, RNF111**
**GO:0009057	Macromolecule catabolic process								42	2,48E-03	RAD23B, UBE3A, KEAP1, AZIN1, UHMK1, ZFP36L1, CYLD, CUL5, TNFRSF1B, LPCAT1, WWP1, AGO1, NOS2, MSN, FBXO9, TRIP12, SAMD4A, HECTD1, TBL1XR1, SMAD7, CBL, CHI3L1, SOCS4, UBR1, PAG6, ATM, PSMB8, CLPX, SIRT2, RNF8, LYVE1, CTSK, RNF115, PYGM, PYGL, SPATA18, UCHL5, SH3D19, TNFAIP3, RNF111
	*GO:0044265	Cellular macromolecule catabolic process							32	3,15E-02	RAD23B, UBE3A, KEAP1, UHMK1, ZFP36L1, CYLD, CUL5, WWP1, AGO1, MSN, FBXO9, SAMD4A, TRIP12, HECTD1, TBL1XR1, SMAD7, CBL, SOCS4, UBR1, PSMB8, CLPX, SIRT2, ATM, RNF8, CTSK, RNF115, SPATA18, UCHL5, TNFAIP3, RNF111
	*GO:0030163	Protein catabolic process							33	6,74E-03	RAD23B, UBE3A, KEAP1, AZIN1, CYLD, TNFRSF1B, CUL5, LPCAT1, WWP1, NOS2, MSN, FBXO9, TRIP12, HECTD1, TBL1XR1, SMAD7, CBL, SOCS4, UBR1, PAG6, PSMB8, CLPX, SIRT2, RNF8, CTSK, RNF115, SPATA18, UCHL5, SH3D19, TNFAIP3, RNF111
		*GO:0044257	Cellular protein catabolic process						27	2,48E-02	RAD23B, UBE3A, KEAP1, CYLD, CUL5, WWP1, MSN, FBXO9, TRIP12, HECTD1, TBL1XR1, SMAD7, CBL, SOCS4, UBR1, PSMB8, CLPX, SIRT2, RNF8, CTSK, RNF115, SPATA18, UCHL5, TNFAIP3, RNF111
		**GO:0006508	Proteolysis						42	6,79E-02	RAD23B, COPS5, UBE3A, KEAP1, ENPEP, CYLD, CUL5, APP, TNFRSF1B, WWP1, KLKB1, CASP1, FBXO9, INPP5B, TRIP12, HECTD1, TBL1XR1, ANP32B, SMAD7, CBL, SOCS4, UBR1, PAG6, RBMX, SIRT2, CLPX, PSMB8, PROC, RNF8, CBLB, CTSK, RENBP, RNF115, DLD, UCHL5, SERPINB1, SH3D19, HSPD1, TNFAIP3, RNF111
			*GO:0051603	Proteolysis involved in cellular protein catabolic process					25	4,35E-02	RAD23B, TBL1XR1, UBE3A, SMAD7, CBL, KEAP1, SOCS4, UBR1, CLPX, PSMB8, SIRT2, RNF8, CYLD, CUL5, CTSK, RNF115, WWP1, UCHL5, TNFAIP3, FBXO9, TRIP12, RNF111, HECTD1
				*GO:0043632	Modification-dependent macromolecule catabolic process				21	7,44E-02	RAD23B, TBL1XR1, SMAD7, UBE3A, CBL, KEAP1, SOCS4, UBR1, SIRT2, RNF8, CYLD, CUL5, RNF115, WWP1, UCHL5, TNFAIP3, FBXO9, TRIP12, HECTD1, RNF111
					*GO:0019941	Modification-dependent protein catabolic process			21	6,52E-02	RAD23B, TBL1XR1, SMAD7, UBE3A, CBL, KEAP1, SOCS4, UBR1, SIRT2, RNF8, CYLD, CUL5, RNF115, WWP1, UCHL5, TNFAIP3, FBXO9, TRIP12, HECTD1, RNF111
						*GO:0006511	Ubiquitin-dependent protein catabolic process		21	5,43E-02	RAD23B, TBL1XR1, SMAD7, UBE3A, CBL, KEAP1, SOCS4, UBR1, SIRT2, RNF8, CYLD, CUL5, RNF115, WWP1, UCHL5, TNFAIP3, FBXO9, TRIP12, HECTD1, RNF111
							*GO:0042787	Protein ubiquitination involved in ubiquitin-dependent protein catabolic process	9	2,41E-02	CUL5, RNF115, UBE3A, WWP1, CBL, KEAP1, TRIP12, RNF111, HECTD1
**GO:0033554**	**Cellular response to stress**								**49**	**6,02E-02**	**MORF4L1, PHLPP1, RAD23B, CPEB2, NUAK2, COPS5, SLC6A1, ASS1, TMX4, BCCIP, FOXO3, DTNBP1, NR2C2, PEA15, CDC45, TNFRSF1B, APOA1, SERPINB10, CHD2, SLC22A3, TRIP12, PDK1, CCNK, NRBF2, POLG, CHST3, APTX, RAD52, ATM, RGS14, SIRT2, SOD2, ATF6, RNF8, EPHA4, DCLRE1A, EP300, KDM2A, FANCD2, HIPK3, SPATA18, EIF2S1, PARPBP, UCHL5, TNFAIP3, ZBTB1, TMCO1, BMPR1A**
**GO:0071495	Cellular response to endogenous stimulus								30	7,42E-02	CGA, FGF7, SSH1, CPEB2, ENPP1, UBE3A, DIAPH1, CPEB4, STAT5A, RPS6KB1, NEO1, NR3C1, CITED2, ACSL1, KDM3A, DNMT3A, KAT2B, SMAD7, SOCS1, ACACA, UBR1, SIRT2, PPP1R9B, EP300, PRLR, MED17, RAB14, RYR1, LHB, RNF111
	*GO:0006974	Cellular response to DNA damage stimulus							25	7,17E-02	MORF4L1, RAD23B, CCNK, POLG, APTX, BCCIP, FOXO3, RAD52, ATM, SOD2, RNF8, PEA15, DCLRE1A, CDC45, TNFRSF1B, EP300, KDM2A, FANCD2, SPATA18, PARPBP, UCHL5, CHD2, ZBTB1, TRIP12
**GO:0006952**	**Defense response**								**48**	**2,11E-03**	**AMCF-II, MASP1, STAT5A, CXCL2, PTGS1, SYNCRIP, NFKBIA, CXCL8, RPS6KB1, TNFRSF4, CCL4, IL17D, TNFRSF1B, TNFRSF11B, APOA1, IL4R, SERPINB10, NOS2, CASP1, FBXO9, FANCA, TNIP1, TRAF3, SELP, CEBPE, LY96, ELANE, SOCS1, SAA3, CHI3L1, CHST3, LGALS8, FXR2, SIRT2, PROC, IFNAR1, EIF4G1, RPS6KA3, ETS1, FANCD2, EIF2S1, IRF1, RAB14, HSPD1, JAK3, TNFAIP3, ABL2, JAM3**
	*GO:0006955	Immune response							40	1,24E-02	AMCF-II, CSF3, ITGAL, MASP1, ENPP1, CXCL2, CD247, CXCL8, NFKBIA, SYNCRIP, TNFRSF4, CCL28, CCL4, SLA-8, TNFRSF11B, TNFRSF1B, APOA1, IL4R, NOS2, FBXO9, TNIP1, TRAF3, LY96, ELANE, SOCS1, IL9, FOXP1, IFNAR1, RPS6KA3, TNFSF13B, IRF1, JAK3, HSPD1, CD79A, TNFAIP3, JAM3, ZBTB1, ABL2, BMPR1A
		*GO:0002764	Immune response-regulating signaling pathway						10	9,54E-02	RPS6KA3, LY96, CD247, IRF1, NFKBIA, CD79A, HSPD1, TNFAIP3, TNIP1, TRAF3
			*GO:0002757	Immune response-activating signal transduction					10	8,03E-02	RPS6KA3, LY96, CD247, IRF1, NFKBIA, CD79A, HSPD1, TNFAIP3, TNIP1, TRAF3
				*GO:0002218	Activation of innate immune response				8	2,07E-02	RPS6KA3, LY96, IRF1, NFKBIA, HSPD1, TNFAIP3, TNIP1, TRAF3
					*GO:0002758	Innate immune response-activating signal transduction			8	1,55E-02	RPS6KA3, LY96, IRF1, NFKBIA, HSPD1, TNFAIP3, TNIP1, TRAF3
	*GO:0009719	Response to endogenous stimulus							36	4,38E-02	ME1, CGA, FGF7, ENPP1, SSH1, CPEB2, DIAPH1, UBE3A, CPEB4, STAT5A, NFKBIA, ABHD2, RPS6KB1, NEO1, NR3C1, CITED2, ACSL1, KDM3A, CASP1, DNMT3A, KAT2B, SMAD7, SOCS1, ACACA, UBR1, SIRT2, PPP1R9B, EP300, PRLR, MED17, RAB14, RYR1, TNFAIP3, LHB, RNF111, PPP2R2A
	*GO:0009628	Response to abiotic stimulus							28	1,35E-02	CPEB2, TRPV2, NFKBIA, DDHD2, APP, MAP3K2, CXCR4, NOS2, CASP1, DNAJA4, PDK1, ELANE, CHI3L1, PPP1CC, SIRT2, ATM, SOD2, RNF8, RDH11, EP300, CKM, GNAQ, FANCD2, EIF2S1, RYR1, IRF1, HSPD1, ZBTB1
		*GO:0006979	Response to oxidative stress						16	3,54E-03	PDK1, STX2, PTGS1, APTX, FOXO3, RAD52, GCLM, SIRT2, SOD2, GPX2, GPX1, APP, FANCD2, TXNRD1, TNFAIP3, NQO1
			*GO:0070482	Response to oxygen levels					9	8,28E-02	PDK1, EP300, CPEB2, CXCR4, RYR1, NOS2, CASP1, SIRT2, SOD2
	*GO:0006954	Inflammatory response							21	3,53E-02	AMCF-II, SELP, STAT5A, ELANE, CXCL2, PTGS1, CHI3L1, SAA3, CXCL8, CCL4, TNFRSF4, PROC, IL17D, TNFRSF11B, TNFRSF1B, APOA1, ETS1, IL4R, NOS2, TNFAIP3, JAM3
**GO:2000026**	**Regulation of multicellular organismal development**								**48**	**3,87E-02**	**PHLPP1, CSF3, MTDH, SSH1, SAV1, TRPV2, STAT5A, NFKBIA, GPM6B, FOXO3, DDR2, NR2C2, CITED2, APP, APOA1, IL4R, LEO1, RARB, GOLGA4, KDM5B, FANCA, LOC100621389, MYOC, KLF13, SMAD7, LDB1, CHI3L1, TBR1, FOXP1, SIRT2, PROC, EIF4G1, SFRP5, HHEX, INHBA, EPHA4, PRLR, ETS1, FANCD2, IRF1, TFAP2A, CUX2, TNFAIP3, ZBTB1, ABL2, BMPR1A, CSN2**
**GO:1903706**	**Regulation of hemopoiesis**								**16**	**4,60E-03**	**CSF3, KLF13, STAT5A, LDB1, NFKBIA, FOXO3, INHBA, ETS1, FANCD2, IL4R, IRF1, LEO1, ZBTB1, FANCA, LOC100621389**
**GO:1903708	Positive regulation of hemopoiesis								8	8,09E-02	CSF3, INHBA, ETS1, IL4R, STAT5A, FOXO3, ZBTB1
**GO:1903707	Negative regulation of hemopoiesis								8	1,67E-02	KLF13, IL4R, LDB1, STAT5A, IRF1, LEO1, NFKBIA, LOC100621389
*GO:0045637	Regulation of myeloid cell differentiation								11	8,80E-03	CSF3, INHBA, ETS1, KLF13, LDB1, STAT5A, LEO1, NFKBIA, FOXO3, LOC100621389
	*GO:0045646	Regulation of erythrocyte differentiation							6	2,81E-03	INHBA, ETS1, KLF13, LDB1, STAT5A, FOXO3
	*GO:0045638	Negative regulation of myeloid cell differentiation							6	2,20E-02	KLF13, LDB1, STAT5A, LEO1, NFKBIA, LOC100621389
		*GO:0045647	Negative regulation of erythrocyte differentiation						3	2,74E-02	KLF13, LDB1, STAT5A
*GO:0045589	Regulation of regulatory T cell differentiation								3	4,47E-02	FANCD2, IRF1, FANCA
**GO:0002682**	**Regulation of immune system process**								**46**	**1,69E-04**	**PHLPP1, AMCF-II, CSF3, MASP1, STAT5A, CXCL2, CD247, NFKBIA, CXCL8, FOXO3, CCL28, CCL4, AQP3, APOA1, IL4R, SERPINB10, LEO1, MSN, FANCA, TNIP1, LOC100621389, TRAF3, SELP, SIT1, LY96, KLF13, LDB1, ELANE, SOCS1, CHST3, FXR2, FOXP1, INHBA, RPS6KA3, TNFSF13B, ETS1, FANCD2, EIF2S1, IRF1, JAK3, HSPD1, CD79A, TNFAIP3, JAM3, ZBTB1**
*GO:0002684	Positive regulation of immune system process								29	4,59E-03	CSF3, AMCF-II, MASP1, STAT5A, CD247, CXCL2, CXCL8, NFKBIA, FOXO3, CCL4, AQP3, IL4R, TNIP1, TRAF3, SELP, LY96, ELANE, FOXP1, INHBA, RPS6KA3, TNFSF13B, ETS1, IRF1, JAK3, HSPD1, CD79A, TNFAIP3, ZBTB1
*GO:0045088	Regulation of innate immune response								9	9,95E-02	RPS6KA3, LY96, SOCS1, IRF1, NFKBIA, HSPD1, TNFAIP3, TNIP1, TRAF3
	*GO:0045089	Positive regulation of innate immune response							8	7,75E-02	RPS6KA3, LY96, IRF1, NFKBIA, HSPD1, TNFAIP3, TNIP1, TRAF3
*GO:0002685	Regulation of leukocyte migration								8	9,16E-02	AMCF-II, SELP, CXCL2, CXCL8, MSN, JAM3, CCL28, CCL4

	*GO:0050901	Leukocyte tethering or rolling			3	9,97E-02	SELP, ITGB7, CCL28
**GO:0045595**	**Regulation of cell differentiation**				**43**	**5,99E-02**	**CSF3, ENPP1, SSH1, SAV1, STAT5A, TRPV2, NFKBIA, FOXO3, DDR2, NR2C2, APP, APOA1, IL4R, LEO1, KDM3A, RARB, GOLGA4, FANCA, LOC100621389, MYOC, CEBPA, ANP32B, KLF13, SMAD7, LDB1, TBR1, FOXP1, SIRT2, PROC, SOD2, EIF4G1, INHBA, EPHA4, PRLR, ETS1, FANCD2, IRF1, TFAP2A, CUX2, ZBTB1, ABL2, BMPR1A**
**GO:0042127**	**Regulation of cell proliferation**				**43**	**3,03E-02**	**AMCF-II, CSF3, SAV1, STAT5A, CXCL2, PTGS1, CXCL8, NFKBIA, FOXO3, NR2E3, TNFRSF4, DDR2, P14ARF, TNFRSF1B, TNFRSF11B, TGFA, RARB, NOS2, KDM5B, FANCA, CEBPA, KAT2B, KLF13, ELANE, KLF11, LOC100154071, PTPRU, FOXP1, SIRT2, SOD2, SFRP5, HHEX, INHBA, PPP1R9B, TNFSF13B, IRF1, TFAP2A, JAK3, SMARCA1, TNFAIP3, ABL2, BMPR1A**
**GO:0080134**	**Regulation of response to stress**				**40**	**4,40E-03**	**PHLPP1, AMCF-II, COPS5, STAT5A, ENPP4, NFKBIA, DTNBP1, CCL4, TNFRSF1B, APOA1, KLKB1, SERPINB10, NOS2, CASP1, FANCA, TNIP1, TRIP12, TRAF3, SELP, MLC1, STX2, LY96, SOCS1, CHST3, RAD52, FXR2, SIRT2, PROC, RNF8, EPHA4, RPS6KA3, ETS1, FANCD2, HIPK3, EIF2S1, PARPBP, IRF1, HSPD1, TNFAIP3, BMPR1A**
**GO:0032101	Regulation of response to external stimulus				30	3,09E-03	AMCF-II, STAT5A, ENPP4, CXCL2, CXCL8, CCL4, TNFRSF1B, APOA1, CXCR4, KLKB1, SERPINB10, NOS2, CASP1, FANCA, TNIP1, TRAF3, SELP, STX2, LY96, CHST3, FXR2, TBR1, SIRT2, PROC, EPHA4, ETS1, FANCD2, EIF2S1, TNFAIP3, JAM3
	*GO:0002831	Regulation of response to biotic stimulus			10	2,76E-02	AMCF-II, LY96, EIF2S1, SERPINB10, CHST3, HSPD1, FXR2, TNFAIP3, SIRT2, TRAF3
**GO:0046907**	**Intracellular transport**				**40**	**7,39E-02**	**ARV1, SEC24A, AP4E1, GDAP1, NFKBIA, KEAP1, ZIC1, DTNBP1, WHAMM, UHMK1, HOOK1, TBC1D16, DDHD2, APP, MDFIC, IL4R, ANKRD50, MSN, GOLGA4, ABCE1, STX1A, MLC1, STX2, ANP32B, ARFIP1, PPP1CC, RGS14, ATXN1, SFRP5, HHEX, CTSK, SGSM1, KIF1B, KPNA7, RAB14, KPNA5, ANKFY1, VPS26A, TBC1D20, BMPR1A**
*GO:0006820	Anion transport				13	4,78E-02	CLN3, SLCO4C1, SLC6A1, SLC12A2, ENPP1, LOC100156775, SLC16A7, CLIC5, SLC16A8, SLC37A2, NOS2, SLC27A2, CROT
*GO:0006869	Lipid transport				12	2,58E-02	ABCG8, INHBA, ARV1, APOA1, ACSL1, NFKBIA, NOS2, ABCA3, SLC27A2, KDM5B. ACSL3. CROT
	*GO:0044539	Long-chain fatty acid import			2	7,57E-02	ACSL1, ACSL3
*GO:0006836	Neurotransmitter transport				8	5,88E-02	SNAP29, STX1A, SYN1, STX2, SLC6A1, SLC22A3, SV2B, DTNBP1
*GO:0046942	Carboxylic acid transport				8	8,79E-02	CLN3, LOC100156775, SLC16A7, SLC6A1, SLC16A8, NOS2, SLC27A2, CROT
*GO:0070634	Transepithelial ammonium transport				2	7,57E-02	RHCG, SLC12A2
**GO:0006629**	**Lipid metabolic process**				**38**	**1,70E-02**	**TM7SF2, ARV1, HSD3B1, STAT5A, PTGS1, ABHD2, ST3GAL1, DDHD2, APP, ACSL1, APOA1, LPCAT1, ST3GAL4, GGPS1, ELOVL7, SDR16C5, PIK3R5, INPP5B, ETFA, HSD17B8, CEBPA, PDK1, TBL1XR1, CLN3, NRBF2, PIGV, ACACA, SAA3, FADS2, DECR1, MID1IP1, ATM, SIRT2, SAMD8, RDH11, PIP4K2A, ALG12, CROT**
*GO:0044255	Cellular lipid metabolic process				29	3,32E-02	ARV1, PTGS1, ABHD2, ST3GAL1, DDHD2, APOA1, LPCAT1, ST3GAL4, GGPS1, ELOVL7, SDR16C5, PIK3R5, INPP5B, HSD17B8, ETFA, PDK1, CLN3, PIGV, ACACA, SAA3, DECR1, MID1IP1, SIRT2, ATM, SAMD8, RDH11, PIP4K2A, ALG12, CROT
**GO:0007010**	**Cytoskeleton organization**				**35**	**6,06E-02**	**TLN1, SSH1, NUAK2, DIAPH1, PDLIM3, GPM6B, ARPC5, MYBL2, DTNBP1, TPM1, WHAMM, APOA1, CAMSAP2, MYOC, CHD3, IQSEC2, ACTA1, MAP1A, MID1IP1, ARFIP1, FOXP1, RGS14, NCKAP1, TACC2, TMEM67, ARPC1B, PPP1R9B, LARP4, CEP68, SYNM, SH3D19, JAM3, ABL2, GFRA2, ARHGAP10**
*GO:0030036	Actin cytoskeleton organization				21	3,36E-02	TLN1, ACTA1, SSH1, NUAK2, DIAPH1, PDLIM3, ARPC5, ARFIP1, TPM1, DTNBP1, FOXP1, WHAMM, NCKAP1, ARPC1B, PPP1R9B, APOA1, ABL2, JAM3, MYOC, GFRA2, IQSEC2
	*GO:0045010	Actin nucleation			5	3,35E-02	ARPC1B, ARPC5, ARFIP1, WHAMM, NCKAP1
*GO:0014902	Myotube differentiation				6	8,12E-02	FAM65B, ACTA1, IL4R, RYR1, PLEKHO1, BCL9
**GO:0016192**	**Vesicle-mediated transport**				**35**	**6,24E-02**	**SNAP29, CALY, SEC24A, AP4E1, COPS5, UBE3A, EEA1, DTNBP1, WHAMM, HOOK1, TBC1D16, DDHD2, APP, IL4R, ANKRD50, GOLPH3L, MSN, GOLGA4, SCAMP1, CLN3, STX1A, MLC1, CEBPE, STX2, ELANE, CBL, RAB11FIP2, SGSM1, ATP9A, RAB14, RAB13, TBC1D20, ANKFY1, VPS26A, ABL2**
**GO:0002520**	**Immune system development**				**34**	**4,79E-03**	**PHLPP1, CSF3, STAT5A, NFKBIA, FOXO3, CITED2, ZFP36L1, IL4R, CHD2, LEO1, FANCA, LOC100621389, CEBPA, CEBPE, KLF13, LDB1, FOXP1, ATM, SOD2, INHBA, HHEX, EP300, ETS1, FANCD2, HBZ, IRF1, JAK3, HSPD1, CD79A, PIP4K2A, TNFAIP3, JAM3, ZBTB1**
*GO:0048534	Hematopoietic or lymphoid organ development				31	1,27E-02	CSF3, STAT5A, NFKBIA, FOXO3, CITED2, ZFP36L1, IL4R, CHD2, LEO1, FANCA, LOC100621389, CEBPA, CEBPE, KLF13, LDB1, FOXP1, ATM, SOD2, INHBA, HHEX, EP300, ETS1, FANCD2, HBZ, IRF1, JAK3, CD79A, PIP4K2A, JAM3, ZBTB1
	*GO:0030097	Hemopoiesis			31	5,33E-03	CSF3, STAT5A, NFKBIA, FOXO3, CITED2, ZFP36L1, IL4R, CHD2, LEO1, FANCA, LOC100621389, CEBPA, CEBPE, KLF13, LDB1, FOXP1, ATM, SOD2, INHBA, HHEX, EP300, ETS1, FANCD2, HBZ, IRF1, JAK3, CD79A, PIP4K2A, JAM3, ZBTB1
		*GO:0030099	Myeloid cell differentiation		18	1,53E-03	CSF3, CEBPA, CEBPE, KLF13, LDB1, STAT5A, NFKBIA, FOXO3, CITED2, INHBA, HHEX, EP300, ETS1, HBZ, LEO1, PIP4K2A, LOC100621389
			*GO:0030218	Erythrocyte differentiation	8	9,59E-03	INHBA, ETS1, KLF13, LDB1, STAT5A, HBZ, FOXO3, CITED2
	**GO:0042113	B cell activation			11	7,48E-02	HHEX, EP300, TNFSF13B, STAT5A, JAK3, CD79A, HSPD1, TNFAIP3, ZBTB1, ATM. FOXP1
		*GO:0030183	B cell differentiation		8	7,41E-02	HHEX, EP300, STAT5A, JAK3, CD79A, ZBTB1, ATM, FOXP1
**GO:0051276**	**Chromosome organization**				**33**	**3,26E-02**	**MORF4L1, RAD23B, LOC100158121, NR3C1, CDC45, LEO1, KDM3A, LOC100153329, TRIP12, LOC100621389, HELLS, GNL3, CHD3, LOC100156127, DNMT3A, TBL1XR1, BRD2, KAT2B, ANP32B, LDB1, LOC100154071, SIRT2, ATM, RNF8, HHEX, DCLRE1A, EP300, KDM2A, FANCD2, MCMBP, CABIN1, SMARCA1, ZBTB1**
**GO:0043603**	**Cellular amide metabolic process**				**32**	**4,67E-02**	**ABCF1, CPEB2, ASS1, CPEB4, GDAP1, SYNCRIP, RPS6KB1, FOXO3, ENPEP, VARS, GCLM, UHMK1, ST3GAL1, ZFP36L1, RPS27, APP, ST3GAL4, AGO1, EIF2B2, SAMD4A, CEBPA, CLN3, ABCE1, DENR, FXR2, ATM, SOD2, SAMD8, EIF4G1, TARS, EIF2S1, GRB7**
*GO:0043604	Amide biosynthetic process				26	5,67E-02	ABCF1, CPEB2, ASS1, CPEB4, SYNCRIP, RPS6KB1, VARS, GCLM, UHMK1, ZFP36L1, ST3GAL1, RPS27, ST3GAL4, AGO1, EIF2B2, SAMD4A, CEBPA, ABCE1, DENR, FXR2, ATM, SAMD8, EIF4G1, TARS, EIF2S1, GRB7
*GO:0034248	Regulation of cellular amide metabolic process				17	2,03E-03	ABCF1, CPEB2, CPEB4, SYNCRIP, RPS6KB1, FOXO3, FXR2, UHMK1, EIF4G1, ZFP36L1, SAMD8, APP, EIF2S1, AGO1, EIF2B2, GRB7, SAMD4A
	*GO:0034249	Negative regulation of cellular amide metabolic process			9	1,36E-02	ZFP36L1, CPEB2, EIF2S1, CPEB4, AGO1, SYNCRIP, FXR2, GRB7, SAMD4A
**GO:0046903**	**Secretion**				**28**	**8,21E-02**	**SNAP29, CGA, SEC24A, COPS5, STAT5A, FFAR1, DTNBP1, APOA1, SYN1, IL4R, GOLPH3L, NOS2, CASP1, ACSL3, KDM5B, SCAMP1, STX1A, STX2, SOCS1, CHI3L1, ARFIP1, FOXP1, INHBA, RAB11FIP2, TNFSF13B, PRLR, RAB13, CSN2**
**GO:0098602**	**Single organism cell adhesion**				**27**	**3,37E-02**	**ITGAL, TLN1, ASS1, STAT5A, CCL28, CITED2, ZFP36L1, APOA1, IL4R, ITGB7, MSN, TNIP1, FANCA, MYOC, SELP, SIT1, SMAD7, PTPRU, FOXP1, TNFSF13B, ETS1, FANCD2, IRF1, JAK3, HSPD1, JAM3, ZBTB1**
*GO:0016337	Single organismal cell-cell adhesion				26	2,53E-02	ITGAL, TLN1, ASS1, STAT5A, CCL28, CITED2, ZFP36L1, APOA1, IL4R, ITGB7, MSN, TNIP1, FANCA, SELP, SIT1, SMAD7, PTPRU, FOXP1, TNFSF13B, ETS1, FANCD2, IRF1, JAK3, HSPD1, JAM3, ZBTB1
	*GO:0007159	Leukocyte cell-cell adhesion			20	2,66E-02	ITGAL, SELP, SIT1, ASS1, STAT5A, CCL28, FOXP1, ZFP36L1, TNFSF13B, ETS1, FANCD2, IL4R, ITGB7, IRF1, HSPD1, MSN, JAK3, TNIP1, FANCA, ZBTB1
**GO:0031347**	**Regulation of defense response**				**25**	**2,09E-03**	**AMCF-II, STAT5A, NFKBIA, CCL4, TNFRSF1B, APOA1, SERPINB10, NOS2, CASP1, TNIP1, FANCA, TRAF3, LY96, SOCS1, CHST3, FXR2, SIRT2, PROC, RPS6KA3, FANCD2, ETS1, EIF2S1, IRF1, HSPD1, TNFAIP3**
	*GO:0050727	Regulation of inflammatory response			12	4,74E-02	TNFRSF1B, APOA1, ETS1, FANCD2, STAT5A, NOS2, TNFAIP3, CASP1, TNIP1, CCL4, FANCA, PROC
**GO:0044703**	**Multi-organism reproductive process**				**24**	**5,39E-02**	**RAD23B, DNMT3A, STX2, UBE3A, STAT5A, ABHD2, RPS6KB1, FOXO3, SIRT2, NR2C2, CITED2, KDM1B, HOOK1, INHBA, APP, FANCD2, DLD, KDM3A, PABPC1L, INPP5B, TBC1D20, LHB, JAM3, KDM5B**
**GO:0032504**	**Multicellular organism reproduction**				**23**	**1,44E-02**	**RAD23B, DNMT3A, UBE3A, STAT5A, ABHD2, RPS6KB1, FOXO3, SIRT2, NR2C2, CITED2, KDM1B, HOOK1, INHBA, APP, FANCD2, DLD, KDM3A, EIF2B2, PABPC1L, INPP5B, TBC1D20, JAM3, LHB**
*GO:0048609	Multicellular organismal reproductive process				23	1,21E-02	RAD23B, DNMT3A, UBE3A, STAT5A, ABHD2, RPS6KB1, FOXO3, SIRT2, NR2C2, CITED2, KDM1B, HOOK1, INHBA, APP, FANCD2, DLD, KDM3A, EIF2B2, PABPC1L, INPP5B, TBC1D20, JAM3, LHB
	*GO:0019953	Sexual reproduction			22	4,76E-02	RAD23B, DNMT3A, STX2, UBE3A, ABHD2, RPS6KB1, FOXO3, SIRT2, NR2C2, KDM1B, HOOK1, INHBA, APP, FANCD2, DLD, KDM3A, PABPC1L, INPP5B, TBC1D20, JAM3, KDM5B, LHB
		*GO:0007276	Gamete generation		18	5,28E-02	RAD23B, DNMT3A, ABHD2, RPS6KB1, FOXO3, SIRT2, NR2C2, KDM1B, HOOK1, INHBA, FANCD2, DLD, KDM3A, PABPC1L, INPP5B, JAM3, LHB, TBC1D20
			*GO:0030728	Ovulation	3	4.47E-02	INHBA. FOXO3. LHB
	*GO:0042698	Ovulation cycle			5	7.23E-02	INHBA. UBE3A. STAT5A. FOXO3. EIF2B2
		*GO:0022602	Ovulation cycle process		5	6.23E-02	INHBA. UBE3A. STAT5A. FOXO3. EIF2B2
**GO:0003006**	**Developmental process involved in reproduction**				**23**	**5,24E-02**	**CEBPA, CGA, UBE3A, ARID5B, STAT5A, ABHD2, RPS6KB1, FOXO3, SIRT2, CITED2, HOOK1, ZFP36L1, INHBA, PRLR, DLD, KDM3A, EIF2B2, PABPC1L, FANCA, TBC1D20, JAM3, KDM5B, BMPR1A**
GO:0007548	Sex differentiation				11	4,82E-02	CGA, INHBA, UBE3A, STAT5A, ARID5B, FOXO3, EIF2B2, FANCA, TBC1D20, CITED2, BMPR1A

	*GO:0045137	Development of primary sexual characteristics				10	1,69E-02	CGA, INHBA, UBE3A, STAT5A, ARID5B, FOXO3, EIF2B2, FANCA, TBC1D20, CITED2
		*GO:0008406	Gonad development			10	1,50E-02	CGA, INHBA, UBE3A, STAT5A, ARID5B, FOXO3, EIF2B2, FANCA, TBC1D20, CITED2
		*GO:0046545	Development of primary female sexual characteristics			7	1,21E-02	INHBA, UBE3A, STAT5A, ARID5B, FOXO3, EIF2B2, FANCA
			*GO:0008585	Female gonad development		7	1,21E-02	INHBA, UBE3A, STAT5A, ARID5B, FOXO3, EIF2B2, FANCA
				*GO:0001541	Ovarian follicle development	4	9,97E-02	INHBA, UBE3A, FOXO3, EIF2B2
	*GO:0046660	Female sex differentiation				7	2,38E-02	INHBA, UBE3A, STAT5A, ARID5B, FOXO3, EIF2B2, FANCA
**GO:0030155**	**Regulation of cell adhesion**					**21**	**3,53E-02**	**SIT1, ASS1, SMAD7, LDB1, STAT5A, GPM6B, CCL28, CITED2, APOA1, TNFSF13B, PRLR, ETS1, FANCD2, IL4R, IRF1, HSPD1, JAK3, FANCA, ZBTB1, ABL2, MYOC**
**GO:0045785	Positive regulation of cell adhesion					13	8,43E-02	SMAD7, STAT5A, LDB1, CCL28, CITED2, APOA1, TNFSF13B, ETS1, IL4R, HSPD1, JAK3, ZBTB1, MYOC
*GO:0022407	Regulation of cell-cell adhesion					16	1,21E-02	SIT1, ASS1, SMAD7, STAT5A, CCL28, CITED2, APOA1, TNFSF13B, ETS1, FANCD2, IL4R, IRF1, HSPD1, JAK3, FANCA, ZBTB1
	**GO:0022409	Positive regulation of cell-cell adhesion				9	9,95E-02	TNFSF13B, ETS1, SMAD7, IL4R, STAT5A, JAK3, HSPD1, ZBTB1, CITED2
	*GO:1903037	Regulation of leukocyte cell-cell adhesion				13	2,30E-02	SIT1, ASS1, STAT5A, CCL28, TNFSF13B, ETS1, FANCD2, IL4R, IRF1, HSPD1, JAK3, ZBTB1, FANCA
**GO:0048589**	**Developmental growth**					**20**	**7,08E-02**	**CGA, TBL1XR1, UBE3A, ARID5B, STAT5A, TRPV2, SAV1, FOXO3, RAPH1, DDR2, FOXP1, ZFP36L1, HHEX, APP, PRLR, RARB, GOLGA4, KDM5B, BMPR1A, BCL9**
*GO:0035265	Organ growth					7	8,03E-02	CGA, PRLR, UBE3A, SAV1, RARB, DDR2, FOXP1
*GO:0043900	Regulation of multi-organism process					18	2,63E-02	AMCF-II, CCNK, LY96, ELANE, PKN2, CHST3, FXR2, CCL4, SIRT2, INHBA, EP300, MDFIC, EIF2S1, SERPINB10, NOS2, TNFAIP3, TBC1D20, TRAF3
*GO:0043903	Regulation of symbiosis, encompassing mutualism through parasitism					13	6,64E-02	AMCF-II, CCNK, EP300, MDFIC, EIF2S1, ELANE, SERPINB10, PKN2, CHST3, FXR2, CCL4, TBC1D20, TRAF3
	*GO:0051702	Interaction with symbiont				6	1,64E-02	AMCF-II, CCNK, EP300, ELANE, CCL4, TBC1D20
*GO:0035821	Modification of morphology or physiology of other organism					8	4,49E-03	AMCF-II, CCNK, EP300, ELANE, NOS2, TNIP1, CCL4, TBC1D20
	*GO:0051817	Modification of morphology or physiology of other organism involved in symbiotic interaction				7	1,21E-02	AMCF-II, CCNK, EP300, ELANE, TNIP1, CCL4, TBC1D20
	*GO:0044364	Disruption of cells of other organism				3	7,60E-02	AMCF-II, ELANE, NOS2
		*GO:0031640	Killing of cells of other organism			3	7,60E-02	AMCF-II, ELANE, NOS2
	*GO:0051851	Modification by host of symbiont morphology or physiology				6	1,47E-02	AMCF-II, CCNK, EP300, ELANE, CCL4, TBC1D20
*GO:0043902	Positive regulation of multi-organism process					8	6,47E-02	INHBA, EP300, LY96, MDFIC, PKN2, NOS2, TBC1D20, SIRT2
**GO:0048732**	**Gland development**					**17**	**1,20E-02**	**CEBPA, CGA, UBE3A, ARID5B, STAT5A, SOD2, CITED2, HHEX, APOA1, PRLR, TGFA, MSN, TNFAIP3, ZBTB1, KDM5B, BMPR1A, CSN2**
*GO:0001889	Liver development					6	2,20E-02	CEBPA, HHEX, TGFA, TNFAIP3, CITED2, SOD2
*GO:0030325	Adrenal gland development					3	6,49E-02	APOA1, ARID5B, CITED2
**GO:0006914**	**Autophagy**					**17**	**7,09E-02**	**SNAP29, PDK1, NRBF2, MTDH, SLC6A1, LGALS8, CHST3, FXR2, SIRT2, NR2C2, CTSK, SPATA18, EIF2S1, SERPINB10, SLC22A3, MCOLN1, PIP4K2A**
**GO:0010876**	**Lipid localization**					**14**	**1,88E-02**	**ARV1, ENPP1, STAT5A, NFKBIA, ABCA3, ABCG8, INHBA, APOA1, ACSL1, NOS2, ACSL3, KDM5B, SLC27A2, CROT**
**GO:0007517**	**Muscle organ development**					**13**	**3,58E-02**	**FAM65B, EP300, ACTA1, SMAD7, SAV1, ARID5B, CHD2, RYR1, SIRT2, FOXP1, MYOC, CITED2, BCL9**
*GO:0060537	Muscle tissue development					13	9,95E-02	FAM65B, EP300, ACTA1, SMAD7, PLN, SAV1, RYR1, RARB, FOXP1, SIRT2, MYOC, CITED2, BCL9
	*GO:0014706	Striated muscle tissue development				13	7,50E-02	FAM65B, EP300, ACTA1, SMAD7, PLN, SAV1, RYR1, RARB, FOXP1, SIRT2, MYOC, CITED2, BCL9
**GO:0045444**	**Fat cell differentiation**					**12**	**7,04E-03**	**CEBPA, TBL1XR1, EP300, ENPP1, SAV1, ARID5B, LRRC8C, SOCS1, FBXO9, PSMB8, SIRT2, SOD2**
**GO:0031589**	**Cell-substrate adhesion**					**12**	**5,67E-02**	**ITGAL, APOA1, TMEM8B, SNED1, ITGB7, LDB1, GPM6B, JAM3, CCL28, WHAMM, MYOC, MUC4**
*GO:0007160	Cell-matrix adhesion					11	6,35E-03	ITGAL, TMEM8B, SNED1, ITGB7, LDB1, GPM6B, JAM3, CCL28, WHAMM, MYOC, MUC4
**GO:0050878**	**Regulation of body fluid levels**					**12**	**6,94E-02**	**SELP, TLN1, STX2, PRLR, STAT5A, KLKB1, ENPP4, ADRA2B, DTNBP1, AQP3, CSN2, PROC**
**GO:0006790**	**Sulfur compound metabolic process**					**11**	**9,09E-02**	**PDK1, TPK1, ENPP1, CSGALNACT2, GDAP1, ACACA, CHST3, MAT2B, CHSY1, GCLM, SOD2**
*GO:0044272	Sulfur compound biosynthetic process					6	9,54E-02	PDK1, CSGALNACT2, CHST3, MAT2B, CHSY1, GCLM
	*GO:0050650	Chondroitin sulfate proteoglycan biosynthetic process				3	8,76E-02	CSGALNACT2, CHST3, CHSY1
**GO:0048511**	**Rhythmic process**					**10**	**7,47E-02**	**PHLPP1, INHBA, EP300, GNAQ, UBE3A, STAT5A, FOXO3, NOS2, PPP1CC, EIF2B2**
**GO:0048469**	**Cell maturation**					**9**	**4,33E-02**	**CEBPA, APP, DLD, HBZ, ABHD2, FOXO3, PABPC1L, SIRT2, MYOC**
**GO:0046822**	**Regulation of nucleocytoplasmic transport**					**9**	**8,28E-02**	**SFRP5, ANP32B, MDFIC, NFKBIA, KEAP1, PPP1CC, ZIC1, UHMK1, BMPR1A**
**GO:0002221**	**Pattern recognition receptor signaling pathway**					**8**	**1,33E-02**	**RPS6KA3, LY96, IRF1, NFKBIA, HSPD1, TNFAIP3, TNIP1, TRAF3**
*GO:0002224	Toll-like receptor signaling pathway					8	4,04E-03	RPS6KA3, LY96, IRF1, NFKBIA, HSPD1, TNFAIP3, TNIP1, TRAF3
*GO:0070427	Nucleotide-binding oligomerization domain containing 1 signaling pathway					2	7,57E-02	NFKBIA, TNFAIP3
**GO:0043491**	**Protein kinase B signaling**					**8**	**4,56E-02**	**PHLPP1, SFRP5, MTDH, CHI3L1, PIK3R5, RPS6KB1, SIRT2, MYOC**
**GO:0034329**	**Cell junction assembly**					**8**	**5,60E-02**	**TLN1, SMAD7, LDB1, PKN2, GPM6B, RAB13, WHAMM, MYOC**
*GO:0007044	Cell-substrate junction assembly					5	5,31E-02	TLN1, LDB1, GPM6B, WHAMM, MYOC
**GO:0001101**	**Response to acid chemical**					**8**	**8,09E-02**	**DNMT3A, CPEB4, SOCS1, FFAR1, GDAP1, UBR1, ABL2, AQP3**
**GO:0006575**	**Cellular modified amino acid metabolic process**					**7**	**6,92E-02**	**CGA, CKM, ASS1, GDAP1, ALDH1L2, GCLM, SOD2**
*GO:0006474	N-terminal protein amino acid acetylation					3	8,76E-02	KAT2B, EP300, NAA15
**GO:0048017**	**Inositol lipid-mediated signaling**					**7**	**8,03E-02**	**SELP, UBE3A, CBL, PI4KA, PIK3R5, SIRT2, MYOC**
*GO:0048015	Phosphatidylinositol-mediated signaling					7	7,28E-02	SELP, UBE3A, CBL, PI4KA, PIK3R5, SIRT2, MYOC
	*GO:0014065	Phosphatidylinositol 3-kinase signaling				6	6,82E-02	SELP, UBE3A, CBL, PIK3R5, SIRT2, MYOC
**GO:0007050**	**Cell cycle arrest**					**7**	**7,28E-02**	**INHBA, CCNK, CDKN1A, IRF1, CDKN3, P14ARF, UHMK1**
**GO:0098727**	**Maintenance of cell number**					**7**	**8,43E-02**	**FANCD2, LDB1, MED17, LEO1, FOXO3, BCL9, BMPR1A**
*GO:0019827	Stem cell population maintenance					7	7,65E-02	FANCD2, LDB1, MED17, LEO1, FOXO3, BCL9, BMPR1A
**GO:0061008**	**Hepaticobiliary system development**					**6**	**2,88E-02**	**CEBPA, HHEX, TGFA, TNFAIP3, CITED2, SOD2**
**GO:0006413**	**Translational initiation**					**6**	**7,24E-02**	**ABCF1, ABCE1, EIF2S1, RPS6KB1, DENR, UHMK1**
**GO:0006081**	**Cellular aldehyde metabolic process**					**5**	**2,71E-02**	**RDH11, TKT, SDR16C5, KDM3A, RPIA**
**GO:0051781**	**Positive regulation of cell division**					**5**	**5,76E-02**	**FGF7, PKN2, TGFA, IGF2, SIRT2**
**GO:0051899**	**Membrane depolarization**					**5**	**6,72E-02**	**SCN2B, CATSPER2, CASP1, GCLM, MYOC**
**GO:1900181**	**Negative regulation of protein localization to nucleus**					**5**	**9,46E-02**	**SFRP5, MDFIC, DCLK3, NFKBIA, KEAP1**
**GO:0048048**	**Embryonic eye morphogenesis**					**4**	**4,54E-02**	**TFAP2A, MFAP2, RARB, CITED2**
***GO:0002089**	**Lens morphogenesis in camera-type eye**					**3**	**5,45E-02**	**TDRD7, TBC1D20, CITED2**
**GO:2000348**	**Regulation of CD40 signaling pathway**					**3**	**1,38E-02**	**FANCD2, TNFAIP3, FANCA**
**GO:0042026**	**Protein refolding**					**3**	**2,74E-02**	**DNAJB1, HSPD1, DNAJA4**
**GO:0006656**	**Phosphatidylcholine biosynthetic process**					**3**	**6,49E-02**	**APOA1, LPCAT1, ACSL3**
*GO:2001245	Regulation of phosphatidylcholine biosynthetic process					2	7,57E-02	LPCAT1, ACSL3
**GO:0031629**	**Synaptic vesicle fusion to presynaptic active zone membrane**					**3**	**9,97E-02**	**SNAP29, STX1A, STX2**

*Subset **Part of

Category		Gene numbers	p-value	Genes
	**GO - Cellular Component**			
GO:0044421	Extracellular region part	109	7,48E-02	MASP1, LTBP2, PTGS1, ENPEP, ALDH1L2, ST3GAL1, APP, APOA1, ST3GAL4, WWP1, ZNF445, NQO1, GNL3, POLG, FBP1, OLA1, ARPC1B, RENBP, RHCG, EIF2S1, SLC37A2, RAB14, RYR1, MFAP2, RAB13, TNFAIP3, ANKFY1, VPS26A, PRPS2, AMCF-II, ITGAL, ASS1, ENPP1, ARPC5, CCL4, IL17D, ITGB7, KLKB1, SLCO4C1, PCIF1, ACACA, LOC100154071, CHI3L1, LGALS8, TKT, FXR2, RBMX, NCKAP1, LYVE1, DNAJB1, HSPD1, PABPC1L, LHB, TLN1, DDR2, GPX2, CXCR4, TGFA, SLC22A5, MSN, GOLGA4, LOC100153329, LOC100621389, SIT1, STX2, ANP32B, AIFM2, ACTA1, ELANE, SAA3, PI4KA, DECR1, PROC, TARS, INHBA, CTSK, CKM, CLIC5, GRN, CYBRD1, SERPINB1, TXNRD1, CUX2, JAM3, CSF3, CXCL2, ADAMTS15, CXCL8, EEA1, ATP6V1G1, CCL28, APLP2, SERPINB10, CHD2, MYOC, ETFA, SELP, SLC12A2, IL9, IGF2, KLK1, PAG6, PYGM, SNED1, PYGL, HBZ, MAT2B, CSN2

GO:0031988	Membrane-bounded vesicle	95	4,84E-02	TLN1, SEC24A, LTBP2, COPS5, PTGS1, ENPEP, ALDH1L2, DDR2, ST3GAL1, GPX2, APP, APOA1, CXCR4, ST3GAL4, WWP1, SV2B, ZNF445, SLC22A5, MSN, NQO1, GOLGA4, LOC100153329, LOC100621389, SCAMP1, CLN3, STX1A, SIT1, STX2, ACTA1, ANP32B, POLG, ELANE, FBP1, PI4KA, OLA1, DECR1, TARS, ARPC1B, RENBP, KIF1B, RHCG, CLIC5, EIF2S1, GRN, SLC37A2, DLD, RAB14, RYR1, CYBRD1, TXNRD1, RAB13, CUX2, TNFAIP3, ANKFY1, VPS26A, PRPS2, ITGAL, ASS1, EEA1, ABHD2, ATP6V1G1, ARPC5, CCL28, DTNBP1, APLP2, DDHD2, SYN1, KLKB1, ITGB7, CHD2, MYOC, ETFA, SELP, SLCO4C1, SLC12A2, OPRL1, CHI3L1, ACACA, LOC100154071, LGALS8, TKT, KLK1, FXR2, RBMX, ATM, NCKAP1, LYVE1, SNED1, PYGM, PYGL, HBZ, MAT2B, DNAJB1, HSPD1, PABPC1L
GO:0070062	Extracellular exosome	80	5,05E-02	TLN1, LTBP2, PTGS1, ENPEP, ALDH1L2, DDR2, ST3GAL1, GPX2, APP, APOA1, CXCR4, ST3GAL4, WWP1, ZNF445, MSN, SLC22A5, NQO1, GOLGA4, LOC100153329, LOC100621389, SIT1, STX2, ACTA1, ANP32B, POLG, ELANE, FBP1, PI4KA, OLA1, DECR1, TARS, ARPC1B, RENBP, RHCG, CLIC5, EIF2S1, GRN, SLC37A2, RAB14, CYBRD1, RYR1, TXNRD1, RAB13, CUX2, TNFAIP3, VPS26A, ANKFY1, PRPS2, ITGAL, ASS1, EEA1, ATP6V1G1, ARPC5, CCL28, APLP2, KLKB1, ITGB7, CHD2, MYOC, ETFA, SLCO4C1, SLC12A2, CHI3L1, LOC100154071, ACACA, LGALS8, TKT, KLK1, FXR2, RBMX, NCKAP1, LYVE1, SNED1, PYGM, PYGL, HBZ, MAT2B, DNAJB1, HSPD1, PABPC1L
GO:1903561	Extracellular vesicle	80	5,90E-02	TLN1, LTBP2, PTGS1, ENPEP, ALDH1L2, DDR2, ST3GAL1, GPX2, APP, APOA1, CXCR4, ST3GAL4, WWP1, ZNF445, MSN, SLC22A5, NQO1, GOLGA4, LOC100153329, LOC100621389, SIT1, STX2, ACTA1, ANP32B, POLG, ELANE, FBP1, PI4KA, OLA1, DECR1, TARS, ARPC1B, RENBP, RHCG, CLIC5, EIF2S1, GRN, SLC37A2, RAB14, CYBRD1, RYR1, TXNRD1, RAB13, CUX2, TNFAIP3, VPS26A, ANKFY1, PRPS2, ITGAL, ASS1, EEA1, ATP6V1G1, ARPC5, CCL28, APLP2, KLKB1, ITGB7, CHD2, MYOC, ETFA, SLCO4C1, SLC12A2, CHI3L1, LOC100154071, ACACA, LGALS8, TKT, KLK1, FXR2, RBMX, NCKAP1, LYVE1, SNED1, PYGM, PYGL, HBZ, MAT2B, DNAJB1, HSPD1, PABPC1L
GO:0043230	Extracellular organelle	80	5,97E-02	TLN1, LTBP2, PTGS1, ENPEP, ALDH1L2, DDR2, ST3GAL1, GPX2, APP, APOA1, CXCR4, ST3GAL4, WWP1, ZNF445, MSN, SLC22A5, NQO1, GOLGA4, LOC100153329, LOC100621389, SIT1, STX2, ACTA1, ANP32B, POLG, ELANE, FBP1, PI4KA, OLA1, DECR1, TARS, ARPC1B, RENBP, RHCG, CLIC5, EIF2S1, GRN, SLC37A2, RAB14, CYBRD1, RYR1, TXNRD1, RAB13, CUX2, TNFAIP3, VPS26A, ANKFY1, PRPS2, ITGAL, ASS1, EEA1, ATP6V1G1, ARPC5, CCL28, APLP2, KLKB1, ITGB7, CHD2, MYOC, ETFA, SLCO4C1, SLC12A2, CHI3L1, LOC100154071, ACACA, LGALS8, TKT, KLK1, FXR2, RBMX, NCKAP1, LYVE1, SNED1, PYGM, PYGL, HBZ, MAT2B, DNAJB1, HSPD1, PABPC1L
GO:0005654	Nucleoplasm	74	1,06E-02	MORF4L1, ABCF1, COPS5, CCNT1, RPS6KB1, FOXO3, ZIC1, SART3, CDC45, SMAP2, RARB, TNIP1, FANCA, GOLGA4, CHUK, GNL3, SCAMP1, TBL1XR1, CCNK, PKN2, RAI14, APTX, DECR1, PPP1CC, CLPX, CDKL5, RAB11FIP2, EP300, HIPK3, MED17, DLD, MCMBP, KPNA7, FBXL4, TFAP2A, KPNA5, KIAA1429, RAD23B, MTDH, SSH1, ZBTB11, HCFC2, NR3C1, MYBL2, NR2C2, SF3B1, ATXN2L, MAP3K2, CHD2, LEO1, KDM3A, TRIP12, CHD3, TAF2, DNMT3A, IKZF4, SELP, KAT2B, CEBPE, PCIF1, KANSL1, SMAD7, TAF5, TKT, RGS14, ATXN1, CBLB, PAPOLA, PPIG, ETS1, PARPBP, NOP58, SH3D19, ZBTB1
GO:0097458	Neuron part	32	3,40E-02	SNAP29, CPEB2, SLC6A1, COPS5, CPEB4, TRPV2, PTGS1, EEA1, DTNBP1, UHMK1, MBP, APP, TMEM57, SYN1, SV2B, MYOC, SCAMP1, CLN3, STX1A, STX2, OPRL1, PDYN, RGS14, SIRT2, EPHA4, RDH11, PPP1R9B, KIF1B, GNAQ, CLIC5, SYNM, RAB13
GO:0043005	Neuron projection	22	6,81E-02	CLN3, STX1A, CPEB2, SLC6A1, OPRL1, CPEB4, TRPV2, EEA1, PDYN, DTNBP1, RGS14, UHMK1, MBP, EPHA4, PPP1R9B, APP, KIF1B, TMEM57, SYN1, SV2B, RAB13, MYOC
GO:0044454	Nuclear chromosome part	20	5,06E-02	MORF4L1, LOC100156127, DNMT3A, LDB1, LOC100158121, LOC100154071, APTX, PPP1CC, RBMX, CITED2, DCLRE1A, CDC45, SYN1, IRF1, MCMBP, UCHL5, SMARCA1, LOC100153329, LOC100621389
GO:0000228	Nuclear chromosome	20	9,12E-02	MORF4L1, LOC100156127, DNMT3A, LDB1, LOC100158121, LOC100154071, APTX, PPP1CC, RBMX, CITED2, DCLRE1A, CDC45, SYN1, IRF1, MCMBP, UCHL5, SMARCA1, LOC100153329, LOC100621389
GO:0000785	Chromatin	19	5,15E-02	MORF4L1, LOC100156127, DNMT3A, LDB1, LOC100158121, LOC100154071, APTX, RBMX, CITED2, PARPBP, IRF1, MCMBP, UCHL5, KDM3A, SMARCA1, LOC100153329, HELLS, LOC100621389
GO:0044431	Golgi apparatus part	19	6,56E-02	SCAMP1, CLN3, SLC35A5, CHST3, ARFIP1, WHAMM, SLC35A3, ST3GAL1, SAMD8, SLC35D1, ST3GAL4, IGF2R, ATP9A, CSGALNACT2, RAB14, CHSY1, RAB13, TBC1D20, TMCO1
GO:0000790	Nuclear chromatin	15	1,94E-02	MORF4L1, LOC100156127, DNMT3A, LDB1, LOC100158121, LOC100154071, APTX, RBMX, CITED2, IRF1, MCMBP, UCHL5, SMARCA1, LOC100153329
GO:0000139	Golgi membrane	14	3,30E-02	SLC35A5, CHST3, ARFIP1, WHAMM, SLC35A3, SAMD8, ST3GAL1, SLC35D1, ST3GAL4, CSGALNACT2, RAB14, CHSY1, TBC1D20, TMCO1
GO:0030424	Axon	13	2,42E-02	EPHA4, APP, KIF1B, TMEM57, SYN1, SLC6A1, TRPV2, EEA1, PDYN, DTNBP1, MYOC, UHMK1, MBP
GO:0005769	Early endosome	12	7,50E-03	TBC1D16, EPHA4, CLN3, MLC1, CXCR4, RUSC1, ATP9A, RAB14, EEA1, HSPD1, ANKFY1, VPS26A
GO:0030133	Transport vesicle	11	5,62E-02	SCAMP1, DDHD2, CLN3, APP, STX1A, SEC24A, SYN1, STX2, COPS5, SV2B, DTNBP1
GO:0031984	Organelle subcompartment	11	5,62E-02	SCAMP1, ST3GAL1, CLN3, ARV1, IGF2R, ATP9A, CSGALNACT2, RAB14, CHSY1, ARFIP1, RAB13
GO:0098791	Golgi subcompartment	10	8,43E-02	SCAMP1, ST3GAL1, CLN3, IGF2R, ATP9A, CSGALNACT2, RAB14, CHSY1, ARFIP1, RAB13
GO:0043209	Myelin sheath	10	8,75E-02	SYN1, DLD, ATP5O, TKT, MSN, HSPD1, JAM3, SIRT2, MBP, SOD2
GO:0008021	Synaptic vesicle	8	1,21E-02	SCAMP1, CLN3, STX1A, SYN1, STX2, COPS5, SV2B, DTNBP1
GO:0070382	Exocytic vesicle	8	3,23E-02	SCAMP1, CLN3, STX1A, SYN1, STX2, COPS5, SV2B, DTNBP1
GO:0033267	Axon part	7	4,41E-02	KIF1B, TRPV2, EEA1, PDYN, DTNBP1, MYOC, MBP
GO:0030658	Transport vesicle membrane	6	4,53E-02	SCAMP1, STX1A, SEC24A, SYN1, SV2B, DTNBP1
GO:0099572	Postsynaptic specialization	5	2,17E-02	PPP1R9B, SYN1, CPEB4, DTNBP1, RGS14
GO:0014069	Postsynaptic density	5	2,17E-02	PPP1R9B, SYN1, CPEB4, DTNBP1, RGS14
GO:0030672	Synaptic vesicle membrane	5	2,78E-02	SCAMP1, STX1A, SYN1, SV2B, DTNBP1
GO:0099501	Exocytic vesicle membrane	5	2,78E-02	SCAMP1, STX1A, SYN1, SV2B, DTNBP1
GO:0060076	Excitatory synapse	5	4,29E-02	PPP1R9B, SYN1, CPEB4, DTNBP1, RGS14
GO:0000307	Cyclin-dependent protein kinase holoenzyme complex	4	6,20E-02	CCNK, CCNT1, BCCIP, HSPD1
GO:0044309	Neuron spine	4	6,20E-02	APP, EEA1, DTNBP1, RGS14
GO:0043218	Compact myelin	3	5,17E-02	JAM3, SIRT2, MBP
GO:0019908	Nuclear cyclin-dependent protein kinase holoenzyme complex	3	5,17E-02	CCNT1, BCCIP, HSPD1
**Category**		**Gene numbers**	**p-value**	**Genes**
	**GO - Molecular Process**			
GO:0005524	ATP binding	55	6,70E-03	ABCF1, NUAK2, RPS6KB1, DDR2, TPK1, CSNK2A2, EEF2K, EIF2B2, INSR, CHUK, ABCE1, ACTA1, PKN2, OLA1, CCT6A, CLPX, CDKL5, TARS, KIF1B, CKM, NAV2, HIPK3, ATP9A, LOC102162987, SMARCA1, ENPP1, ASS1, VARS, ABCA3, UHMK1, CHD8, DDX46, SYN1, SNRK, MAP3K2, DCLK3, CHD2, KIF21A, LOC780419, DNAJA4, HELLS, CHD3, ACACA, ATM, ABCG8, EPHA4, RPS6KA3, PYGL, HSPD1, JAK3, PIP4K2A, ABL2, MYLK, GFRA2, BMPR1A
GO:0003677	DNA binding	28	2,76E-02	ZFAND5, SSH1, STAT5A, LOC100158121, NR3C1, LOC100627422, KDM1B, CHD8, APP, HNRNPK, RARB, KDM5B, LOC100153329, LOC100621389, CHD3, LOC100156127, DNMT3A, CEBPB, AIFM2, POLG, LOC100154071, APTX, RAD52, ATM, KDM2A, SMARCA1, TNFAIP3
GO:0003682	Chromatin binding	20	1,83E-03	MORF4L1, TAF2, DNMT3A, BRD2, KAT2B, POLG, LDB1, CCNT1, TLE4, APTX, RBMX, FOXP1, SIRT2, CITED2, RNF8, TFAM, HHEX, CDC45, MCMBP, HELLS
GO:0004674	Protein serine/threonine kinase activity	13	4,63E-02	NUAK2, PKN2, RPS6KB1, UHMK1, ATM, CSNK2A2, RPS6KA3, SNRK, HIPK3, EEF2K, DCLK3, GFRA2, MYLK
GO:0004842	Ubiquitin-protein transferase activity	11	1,82E-02	RNF8, CBLB, UBE3A, UBR5, WWP1, FBXL4, KEAP1, TNFAIP3, TRIP12, TRAF3, HECTD1
GO:0003713	Transcription coactivator activity	10	1,29E-02	CEBPA, KAT2B, EP300, MTDH, UBE3A, ARID5B, MED17, TFAP2A, MYCBP, MYOC
GO:0016887	ATPase activity	9	7,75E-03	ABCG8, ABCF1, ABCE1, KIF1B, OLA1, KIF21A, SMARCA1, ABCA3, ATP8
GO:0044212	Transcription regulatory region DNA binding	9	2,63E-02	TAF2, IKZF4, TBL1XR1, SMAD7, TAF5, ARID5B, KLF11, CCNT1, MYBL2
GO:0016874	Ligase activity	7	5,30E-03	RNF8, CBLB, UBE3A, UBR5, WWP1, TRIP12, HECTD1
GO:0043022	Ribosome binding	6	8,34E-03	ABCF1, CPEB2, EIF2S1, CPEB4, NAA15, OLA1
GO:0008083	Growth factor activity	6	4,37E-02	CSF3, INHBA, FGF7, TGFA, IGF2

GO:0050660	Flavin adenine dinucleotide binding	6	6,96E-02	KDM1B, AIFM2, DLD, TXNRD1, NOS2, ETFA
GO:0001047	Core promoter binding	5	4,54E-02	KANSL1, AGO1, KDM3A, FOXO3, RBMX
GO:0003725	Double-stranded RNA binding	5	6,83E-02	MTDH, AGO1, APTX, MSN, HSPD1
GO:0000030	Mannosyltransferase activity	3	3,61E-02	PIGV, DPY19L4, ALG12
GO:0004467	Long-chain fatty acid-CoA ligase activity	3	6,56E-02	ACSL1, SLC27A2, ACSL3
GO:0008307	Structural constituent of muscle	3	8,85E-02	PDLIM3, SYNM, TPM1
GO:0008184	Glycogen phosphorylase activity	2	7,62E-02	PYGM, PYGL
GO:0005130	Granulocyte colony-stimulating factor receptor binding	2	7,62E-02	CSF3
