# Supplementary material for: Transcriptomic Analysis of Porcine Endometrium during Implantation after In Vitro Stimulation by Adiponectin
Source: Int J Mol Sci. 2019 Mar 16;20(6):1335. doi: 10.3390/ijms20061335 (PMC6470965; doi:10.3390/ijms20061335)
Supplement: Supplementary file 1 [file ijms-20-01335-s001.zip › SF.3..pdf]

Supplementary Table 3

The most significantly enriched gene ontologies (biological process)

GO - Biological Process

						Count	p-value	Altered genes
		Up-regulated				Count	p-value	Altered genes
GO:0006396	RNA processing					12	1,72E-02	SF3B1, PAPOLA, PPIG, TRMT11, TRDMT1, MPHOSPH10, SYNCRIP, WDR3, NOP58, NOP56, ALG11, KIAA1429
	*GO:0034470	ncRNA processing				7	9,69E-03	TRMT11, TRDMT1, MPHOSPH10, WDR3, NOP58, NOP56, ALG11
		*GO:0006364	rRNA processing			5	1,22E-02	MPHOSPH10, WDR3, NOP58, NOP56, ALG11
GO:0022403	Cell cycle phase					11	6,96E-03	TAF2, CCDC99, DCLRE1A, CUL5, PRC1, DBF4, ALG11, FANCA, SMC2, ATM, HELLS
	*GO:0000279	M phase				8	4,09E-02	CCDC99, DCLRE1A, PRC1, ALG11, FANCA, SMC2, ATM, HELLS
GO:0022402	Cell cycle process					11	4,77E-02	TAF2, CCDC99, DCLRE1A, CUL5, PRC1, DBF4, ALG11, FANCA, SMC2, ATM, HELLS
GO:0003006	Developmental process involved in reproduction					9	3,97E-03	HOOK1, PLA2G4A, FGF7, TDRD7, DLD, RQCD1, RPS6KB1, FANCA, BMPR1A
GO:0019953	Sexual reproduction					9	7,68E-02	HOOK1, PLA2G4A, TDRD7, DLD, MYCBP, RPS6KB1, SEPP1, ALG11, ATM
GO:0034660	ncRNA metabolic process					8	7,05E-03	TRMT11, TRDMT1, MPHOSPH10, WDR3, NOP58, NOP56, ALG11, VARS
	*GO:0016072	rRNA metabolic process				5	1,41E-02	MPHOSPH10, WDR3, NOP58, NOP56, ALG11
*GO:0007276	Gamete generation					8	8,92E-02	HOOK1, PLA2G4A, TDRD7, DLD, MYCBP, RPS6KB1, ALG11, ATM
	*GO:0007281	Germ cell development				4	7,47E-02	HOOK1, TDRD7, DLD, RPS6KB1
GO:0000278	Mitotic cell cycle					8	6,82E-02	TAF2, CCDC99, DCLRE1A, CUL5, PRC1, DBF4, SMC2, HELLS
GO:0022613	Ribonucleoprotein complex biogenesis					6	3,07E-02	MPHOSPH10, WDR3, NOP58, NOP56, ALG11, BMS1
	*GO:0042254	Ribosome biogenesis				6	6,63E-03	MPHOSPH10, WDR3, NOP58, NOP56, ALG11, BMS1
GO:0006457	Protein folding					6	2,88E-02	PPIG, FKBP3, CCT6A, HSPD1, UGGT1, CLPX
GO:0014070	Response to organic cyclic substance					5	3,00E-02	TAF2, RYR1, ACACA, RPS6KB1, PPP2R2A
GO:0009141	Nucleoside triphosphate metabolic process					5	3,84E-02	ENPP1, ATP9A, OLA1, ATP5O, ATP8
	*GO:0009143	Nucleoside triphosphate catabolic process				3	2,23E-02	ENPP1, OLA1, ATP5O
GO:0007548	Sex differentiation					5	5,90E-02	PLA2G4A, FGF7, RQCD1, FANCA, BMPR1A
GO:0006352	Transcription initiation					4	4,66E-02	TAF2, SNAPC5, TAF5, MED23
GO:0008654	Phospholipid biosynthetic process					4	7,65E-02	SAMD8, PLA2G4A, FGF7, MBOAT1
GO:0009166	Nucleotide catabolic					3	8,73E-02	ENPP1, OLA1, ATP5O
	*GO:0046034	ATP metabolic process				4	8,18E-02	ATP9A, OLA1, ATP5O, ATP8
GO:0070167	Regulation of biomineral formation					3	3,82E-02	PLA2G4A, ENPP1, BMPR1A
	GO:0030500	Regulation of bone mineralization				3	3,39E-02	PLA2G4A, ENPP1, BMPR1A
GO:0050679	Positive regulation of epithelial cell proliferation					3	5,97E-02	FGF7, TGFA, BMPR1A
GO:0015780	Nucleotide-sugar transport					2	5,67E-02	SLC35A5, SLC35A3
GO:0045022	Early endosome to late endosome transport					2	9,28E-02	HOOK1, EEA1
GO:0048875	Chemical homeostasis within a tissue					2	9,28E-02	PLA2G4A, FGF7
	*GO:0043129	Surfactant homeostasis				2	9,28E-02	PLA2G4A, FGF7
		Down-regulated				Count	p-value	Altered genes
GO:0006793	Phosphorus metabolic process					45	1,43E-04	SSH1, NUAK2, STAT5A, CCNT1, SSH2, DDR2, UHMK1, CSNK2A2, APP, SNRK, CXCR4, MAP3K2, MDFIC, PTPLA, DCLK3, EEF2K, IL1B, DYRK3, ADRA2B, MTMR9, INSR, PDK1, PAN3, NCEH1, SMAD7, PI4KA, PKN2, PTPRU, CDKN3, CDC25C, PPP1CC, CDKL5, DAPK1, SACM1L, EPHA4, RPS6KA3, MTMR14, PRLR, HIPK1, HIPK3, CDC42BPA, JAK3, PIP4K2A, MAP3K13, MYLK
*GO:0006796	Phosphate-containing compound metabolic process					45	1,43E-04	SSH1, NUAK2, STAT5A, CCNT1, SSH2, DDR2, UHMK1, CSNK2A2, APP, SNRK, CXCR4, MAP3K2, MDFIC, PTPLA, DCLK3, EEF2K, IL1B, DYRK3, ADRA2B, MTMR9, INSR, PDK1, PAN3, NCEH1, SMAD7, PI4KA, PKN2, PTPRU, CDKN3, CDC25C, PPP1CC, CDKL5, DAPK1, SACM1L, EPHA4, RPS6KA3, MTMR14, PRLR, HIPK1, HIPK3, CDC42BPA, JAK3, PIP4K2A, MAP3K13, MYLK
	*GO:0016310	Phosphorylation				34	4,33E-03	NUAK2, STAT5A, CCNT1, DDR2, UHMK1, CSNK2A2, APP, SNRK, CXCR4, MAP3K2, MDFIC, EEF2K, DCLK3, IL1B, DYRK3, ADRA2B, INSR, PDK1, PAN3, SMAD7, PI4KA, PKN2, CDKL5, DAPK1, EPHA4, RPS6KA3, PRLR, HIPK1, HIPK3, CDC42BPA, JAK3, PIP4K2A, MAP3K13, MYLK
		*GO:0006468	Protein amino acid phosphorylation			32	9,01E-04	NUAK2, STAT5A, CCNT1, DDR2, UHMK1, CSNK2A2, APP, SNRK, CXCR4, MAP3K2, MDFIC, DCLK3, EEF2K, IL1B, DYRK3, ADRA2B, INSR, PDK1, PAN3, SMAD7, PKN2, CDKL5, DAPK1, EPHA4, RPS6KA3, PRLR, HIPK1, HIPK3, CDC42BPA, JAK3, MAP3K13, MYLK
			*GO:0001932	Regulation of protein amino acid phosphorylation		11	1,33E-02	PPP1R9B, APOA1, MDFIC, MAP3K2, SMAD7, HIPK3, SOCS1, IL1B, BDKRB1, IGF2, INSR
				*GO:0001933	Negative regulation of protein amino acid phosphorylation	4	4,70E-02	SMAD7, SOCS1, BDKRB1, INSR
	*GO:0051174	Regulation of phosphorus metabolic process				29	4,92E-05	CCNT1, BDKRB1, BCCIP, TNFRSF4, APLP2, APP, APOA1, CXCR4, MDFIC, MAP3K2, IL1B, ADRA2B, INSR, CCNK, SMAD7, SOCS1, ELANE, IGF2, CDC25C, CDKN3, SIRT2, ATXN1, PPP1R9B, INHBA, CDKN1A, PPP1R2, PRLR, HIPK3, MAP3K13
		*GO:0019220	Regulation of phosphate metabolic process			29	4,92E-05	CCNT1, BDKRB1, BCCIP, TNFRSF4, APLP2, APP, APOA1, CXCR4, MDFIC, MAP3K2, IL1B, ADRA2B, INSR, CCNK, SMAD7, SOCS1, ELANE, IGF2, CDC25C, CDKN3, SIRT2, ATXN1, PPP1R9B, INHBA, CDKN1A, PPP1R2, PRLR, HIPK3, MAP3K13
			*GO:0042325	Regulation of phosphorylation		28	6,30E-05	CCNT1, BDKRB1, BCCIP, TNFRSF4, APLP2, APP, APOA1, CXCR4, MDFIC, MAP3K2, IL1B, ADRA2B, INSR, CCNK, SMAD7, SOCS1, ELANE, IGF2, CDC25C, CDKN3, SIRT2, ATXN1, INHBA, PPP1R9B, CDKN1A, PRLR, HIPK3, MAP3K13

				*GO:0043549	Regulation of kinase activity		21	8,63E-04	CCNK, CCNT1, ELANE, BCCIP, IGF2, BDKRB1, CDC25C, CDKN3, TNFRSF4, APLP2, APP, CDKN1A, PRLR, CXCR4, MDFIC, MAP3K2, HIPK3, IL1B, ADRA2B, INSR, MAP3K13
					*GO:0045859	Regulation of protein kinase activity	21	5,59E-04	CCNK, CCNT1, ELANE, BCCIP, IGF2, BDKRB1, CDC25C, CDKN3, TNFRSF4, APLP2, APP, CDKN1A, PRLR, CXCR4, MDFIC, MAP3K2, HIPK3, IL1B, ADRA2B, INSR, MAP3K13
					**GO:0000079	Regulation of cyclin-dependent protein kinase activity	6	1,19E-02	CDKN1A, CCNK, CCNT1, BCCIP, CDKN3, CDC25C
				*GO:0042326	Negative regulation of phosphorylation		7	9,27E-04	ATXN1, INHBA, CDKN1A, SMAD7, SOCS1, BDKRB1, INSR
			*GO:0010563	Negative regulation of phosphorus metabolic process			7	1,31E-03	ATXN1, INHBA, CDKN1A, SMAD7, SOCS1, BDKRB1, INSR
				*GO:0045936	Negative regulation of phosphate metabolic process		7	1,31E-03	ATXN1, INHBA, CDKN1A, SMAD7, SOCS1, BDKRB1, INSR
	*GO:0016311	Dephosphorylation					11	6,13E-03	MTMR14, NCEH1, SSH1, SSH2, PTPLA, PTPRU, MTMR9, PPP1CC, CDKN3, CDC25C, SACM1L
**GO:0007242**	**Intracellular signaling cascade**						**44**	**2,79E-02**	**STAT5A, RASGEF1B, NFKBIA, RRAD, TP63, MYO9B, NR3C1, GNG12, APOA1, MCTP2, CXCR4, MAP3K2, MDFIC, RHOBTB1, IL1B, KDM3A, NOS2, ADRA2B, INSR, PDK1, ARHGEF3, IL8, OPRL1, LY96, SMAD7, SOCS1, PI4KA, IGF2, IFNAR1, DAPK1, CBLB, RPS6KA3, PRLR, GNAQ, HIPK1, NCOA4, MED17, UBR5, RAB14, CDC42BPA, JAK3, RAB13, MAP3K13, PHLDB2**
*GO:0030522	Intracellular receptor-mediated signaling pathway						6	4,20E-02	NCOA4, MED17, UBR5, KDM3A, NOS2, NR3C1
*GO:0016055	Wnt receptor signaling pathway						10	7,03E-03	CSNK2A2, SFRP5, HHEX, TBL1XR1, CHD8, LDB1, FBXW4, TLE3, TLE4, PTPRU
*GO:0051897	Positive regulation of protein kinase B signaling cascade						3	3,05E-02	GPX1, IGF2, INSR
**GO:0042592**	**Homeostatic process**						**40**	**1,85E-05**	**STAT5A, TMX4, FFAR1, BDKRB1, FOXO3, CCL28, GCLM, APLP2, MBP, GPX2, GSR, GPX1, APP, APOA1, CXCR4, IL1B, DYRK3, SLC22A5, EIF2B2, INSR, CLN3, SLC12A2, OPRL1, SMAD7, ELANE, IGF2, SOD2, ABCG8, ATXN1, INHBA, CTSK, EP300, TNFSF13B, RHCG, PYGM, GNAQ, PYGL, PLN, HBZ, TXNRD1**
*GO:0048878	Chemical homeostasis						26	1,47E-03	FFAR1, BDKRB1, FOXO3, GCLM, CCL28, APLP2, MBP, APP, APOA1, CXCR4, IL1B, EIF2B2, INSR, CLN3, SMAD7, OPRL1, ELANE, IGF2, SOD2, ATXN1, ABCG8, RHCG, GNAQ, PYGM, PYGL, PLN
	*GO:0050801	Ion homeostasis					19	1,79E-02	CLN3, OPRL1, SMAD7, ELANE, BDKRB1, CCL28, GCLM, APLP2, SOD2, MBP, ATXN1, APP, GNAQ, RHCG, PYGM, CXCR4, PLN, IL1B, EIF2B2
	*GO:0033500	Carbohydrate homeostasis					5	3,55E-02	PYGL, FFAR1, IGF2, FOXO3, INSR
		*GO:0042593	Glucose homeostasis				5	3,55E-02	PYGL, FFAR1, IGF2, FOXO3, INSR
*GO:0019725	Cellular homeostasis						24	1,96E-03	CLN3, SLC12A2, OPRL1, SMAD7, TMX4, ELANE, BDKRB1, GCLM, CCL28, APLP2, SOD2, MBP, ATXN1, GPX1, GSR, APP, GNAQ, RHCG, PYGM, CXCR4, PLN, IL1B, TXNRD1, EIF2B2
	*GO:0055082	Cellular chemical homeostasis					19	9,00E-03	CLN3, OPRL1, SMAD7, ELANE, BDKRB1, CCL28, GCLM, APLP2, SOD2, MBP, ATXN1, APP, GNAQ, RHCG, PYGM, CXCR4, PLN, IL1B, EIF2B2
	*GO:0006873	Cellular ion homeostasis					19	7,57E-03	CLN3, OPRL1, SMAD7, ELANE, BDKRB1, CCL28, GCLM, APLP2, SOD2, MBP, ATXN1, APP, GNAQ, RHCG, PYGM, CXCR4, PLN, IL1B, EIF2B2
**GO:0010604**	**Positive regulation of macromolecule metabolic process**						**40**	**3,10E-04**	**STAT5A, NFKBIA, TP63, FOXO3, NR2E3, CITED2, APP, CHD8, MDFIC, KLKB1, IL1B, KDM3A, RARB, NFATC3, INSR, SAMD4A, CEBPA, TBL1XR1, CEBPB, SMAD7, KLF13, ELANE, IL9, IGF2, TBR1, PSMB8, ATF6, RNF8, ATXN1, INHBA, HHEX, CBLB, EP300, TNFSF13B, ETS1, NCOA4, MED17, IRF1, SH3D19, JAK3**
***GO:0009891**	Positive regulation of biosynthetic process						34	4,39E-04	STAT5A, NFKBIA, TP63, FOXO3, NR2E3, CITED2, CHD8, APP, MDFIC, IL1B, KDM3A, RARB, INSR, NFATC3, SAMD4A, CEBPA, TBL1XR1, CEBPB, KLF13, ELANE, IL9, IGF2, TBR1, SOD2, ATXN1, ATF6, INHBA, HHEX, EP300, ETS1, NCOA4, MED17, IRF1, JAK3
	*GO:0031328	Positive regulation of cellular biosynthetic process					34	3,38E-04	STAT5A, NFKBIA, TP63, FOXO3, NR2E3, CITED2, CHD8, APP, MDFIC, IL1B, KDM3A, RARB, INSR, NFATC3, SAMD4A, CEBPA, TBL1XR1, CEBPB, KLF13, ELANE, IL9, IGF2, TBR1, SOD2, ATXN1, ATF6, INHBA, HHEX, EP300, ETS1, NCOA4, MED17, IRF1, JAK3
	*GO:0010557	Positive regulation of macromolecule biosynthetic process					33	3,18E-04	STAT5A, NFKBIA, TP63, FOXO3, NR2E3, CITED2, CHD8, APP, MDFIC, IL1B, KDM3A, RARB, INSR, NFATC3, SAMD4A, CEBPA, TBL1XR1, CEBPB, KLF13, ELANE, IL9, IGF2, TBR1, ATXN1, ATF6, INHBA, HHEX, EP300, ETS1, NCOA4, MED17, IRF1, JAK3
*GO:0010628	Positive regulation of gene expression						28	1,94E-03	STAT5A, NFKBIA, TP63, FOXO3, NR2E3, CITED2, APP, CHD8, MDFIC, KDM3A, RARB, NFATC3, CEBPA, TBL1XR1, CEBPB, KLF13, TBR1, ATF6, ATXN1, HHEX, INHBA, EP300, TNFSF13B, NCOA4, ETS1, MED17, IRF1, JAK3
	*GO:0045941	Positive regulation of transcription					27	2,65E-03	STAT5A, NFKBIA, TP63, FOXO3, NR2E3, CITED2, APP, CHD8, MDFIC, KDM3A, RARB, NFATC3, CEBPA, TBL1XR1, CEBPB, KLF13, TBR1, ATF6, ATXN1, HHEX, INHBA, EP300, NCOA4, ETS1, MED17, IRF1, JAK3
		*GO:0045893	Positive regulation of transcription, DNA-dependent				26	5,30E-04	STAT5A, TP63, NFKBIA, FOXO3, NR2E3, CITED2, APP, CHD8, MDFIC, RARB, NFATC3, CEBPA, TBL1XR1, CEBPB, KLF13, TBR1, ATF6, ATXN1, HHEX, INHBA, EP300, NCOA4, ETS1, MED17, IRF1, JAK3
	*GO:0051254	Positive regulation of RNA metabolic process					26	6,15E-04	STAT5A, TP63, NFKBIA, FOXO3, NR2E3, CITED2, APP, CHD8, MDFIC, RARB, NFATC3, CEBPA, TBL1XR1, CEBPB, KLF13, TBR1, ATF6, ATXN1, HHEX, INHBA, EP300, NCOA4, ETS1, MED17, IRF1, JAK3

**GO:0010941**	**Regulation of cell death**						**36**	**1,73E-03**	**NUAK2, STAT5A, NFKBIA, TP63, PMAIP1, NR3C1, FOXO3, TNFRSF4, GCLM, CITED2, PEA15, GPX1, CHD8, APP, IL1B, RARB, CASP1, NQO1, TRAF3, CLN3, ARHGEF3, CEBPB, AIFM2, IGF2, SOD2, PROC, DAPK1, INHBA, CDKN1A, TNFSF13B, PRLR, HIPK1, ETS1, IGF2R, HIPK3, TNFAIP3**
*GO:0043067	Regulation of programmed cell death						36	1,63E-03	NUAK2, STAT5A, NFKBIA, TP63, PMAIP1, NR3C1, FOXO3, TNFRSF4, GCLM, CITED2, PEA15, GPX1, CHD8, APP, IL1B, RARB, CASP1, NQO1, TRAF3, CLN3, ARHGEF3, CEBPB, AIFM2, IGF2, SOD2, PROC, DAPK1, INHBA, CDKN1A, TNFSF13B, PRLR, HIPK1, ETS1, IGF2R, HIPK3, TNFAIP3
		*GO:0042981	Regulation of apoptosis				36	1,38E-03	NUAK2, STAT5A, NFKBIA, TP63, PMAIP1, NR3C1, FOXO3, TNFRSF4, GCLM, CITED2, PEA15, GPX1, CHD8, APP, IL1B, RARB, CASP1, NQO1, TRAF3, CLN3, ARHGEF3, CEBPB, AIFM2, IGF2, SOD2, PROC, DAPK1, INHBA, CDKN1A, TNFSF13B, PRLR, HIPK1, ETS1, IGF2R, HIPK3, TNFAIP3
			*GO:0043065	Positive regulation of apoptosis			19	2,75E-02	ARHGEF3, CEBPB, AIFM2, TP63, NR3C1, PMAIP1, FOXO3, DAPK1, INHBA, GPX1, CDKN1A, APP, HIPK1, ETS1, IL1B, RARB, NQO1, CASP1, TRAF3
				*GO:0012502	Induction of programmed cell death		15	3,46E-02	ARHGEF3, CEBPB, AIFM2, TP63, PMAIP1, FOXO3, DAPK1, INHBA, GPX1, APP, CDKN1A, HIPK1, ETS1, CASP1, TRAF3
				*GO:0006917	Induction of apoptosis		15	3,50E-02	ARHGEF3, CEBPB, AIFM2, TP63, PMAIP1, FOXO3, DAPK1, INHBA, GPX1, APP, CDKN1A, HIPK1, ETS1, CASP1, TRAF3
			*GO:0060548	Negative regulation of cell death			21	9,21E-04	CLN3, CEBPB, NUAK2, STAT5A, NFKBIA, TP63, IGF2, GCLM, PROC, SOD2, DAPK1, CITED2, GPX1, PEA15, CDKN1A, CHD8, TNFSF13B, PRLR, HIPK3, IL1B, TNFAIP3
				*GO:0043069	Negative regulation of programmed cell death		21	9,48E-04	CLN3, CEBPB, NUAK2, STAT5A, NFKBIA, TP63, IGF2, GCLM, PROC, SOD2, DAPK1, CITED2, GPX1, PEA15, CDKN1A, CHD8, TNFSF13B, PRLR, HIPK3, IL1B, TNFAIP3
					*GO:0043066	Negative regulation of apoptosis	21	7,74E-04	CLN3, CEBPB, NUAK2, STAT5A, NFKBIA, TP63, IGF2, GCLM, PROC, SOD2, DAPK1, CITED2, GPX1, PEA15, CDKN1A, CHD8, TNFSF13B, PRLR, HIPK3, IL1B, TNFAIP3
**GO:0010033**	**Response to organic substance**						**35**	**4,04E-04**	**ME1, CGA, SLC6A1, STAT5A, PTGS1, NFKBIA, BDKRB1, PMAIP1, NR3C1, GNG12, TNFRSF11B, ACSL1, PLIN2, IL1B, KDM3A, CASP1, EIF2B2, INSR, SELP, APCS, KAT2B, ACTA1, LY96, ATP4B, SOCS1, FBP1, IGF2, PTPRU, APPL1, ATF6, CDKN1A, EP300, PYGM, DNAJB1, HSPB3**
*GO:0009719	Response to endogenous stimulus						21	3,77E-03	ME1, CGA, KAT2B, SLC6A1, ACTA1, ATP4B, STAT5A, SOCS1, PTGS1, FBP1, IGF2, GNG12, PTPRU, APPL1, TNFRSF11B, CDKN1A, EP300, IL1B, KDM3A, EIF2B2, INSR
	*GO:0009725	Response to hormone stimulus					20	2,76E-03	ME1, CGA, KAT2B, SLC6A1, ACTA1, STAT5A, PTGS1, SOCS1, FBP1, IGF2, GNG12, PTPRU, APPL1, TNFRSF11B, CDKN1A, EP300, IL1B, KDM3A, EIF2B2, INSR
		*GO:0032870	Cellular response to hormone stimulus				9	2,06E-02	CGA, KAT2B, STAT5A, FBP1, KDM3A, IGF2, GNG12, APPL1, INSR
*GO:0032101	Regulation of response to external stimulus						11	7,63E-03	GPX2, SELP, CLN3, GPX1, MASP1, IL8, STAT5A, KLKB1, ELANE, IGF2, PROC
	*GO:0009617	Response to bacterium					12	1,07E-02	CFP, GPX2, SELP, GPX1, CEBPE, ATP4B, LY96, SOCS1, NFKBIA, IL1B, BDKRB1, NOS2
		*GO:0032496	Response to lipopolysaccharide				7	1,37E-02	SELP, ATP4B, LY96, SOCS1, NFKBIA, IL1B, BDKRB1
**GO:0006357**	**Regulation of transcription from RNA polymerase II promoter**						**34**	**9,46E-04**	**SUPT3H, STAT5A, NFKBIA, TP63, FOXO3, NR2E3, CITED2, CHD8, APP, RARB, NFATC3, CHD3, CEBPA, DNMT3A, TBL1XR1, CEBPB, SMAD7, KLF13, KLF11, TBR1, FOXP1, SOD2, ATXN1, ATF6, INHBA, HHEX, EP300, ETS1, MED17, HBZ, IRF1, TFAP2A, PHF21A, JAK3**
*GO:0045944	Positive regulation of transcription from RNA polymerase II promoter						24	7,66E-05	CEBPA, TBL1XR1, CEBPB, KLF13, STAT5A, NFKBIA, TP63, NR2E3, FOXO3, TBR1, CITED2, ATXN1, ATF6, INHBA, HHEX, CHD8, APP, EP300, ETS1, MED17, IRF1, RARB, JAK3, NFATC3
***GO:0000122**	**Negative regulation of transcription from RNA polymerase II promoter**						**13**	**3,97E-02**	**CEBPA, DNMT3A, TBL1XR1, SMAD7, KLF11, TP63, NR2E3, FOXP1, CITED2, HHEX, HBZ, PHF21A, RARB**
**GO:0010605**	**Negative regulation of macromolecule metabolic process**						**33**	**2,11E-03**	**EIF2C1, MASP1, TP63, BDKRB1, NR2E3, TNFRSF4, CITED2, WWP1, SERPINB10, RARB, KDM5B, INSR, CEBPA, DNMT3A, CLN3, TBL1XR1, SMAD7, ARID5B, LDB1, ELANE, KLF11, SOCS1, IGF2, PTPRU, FOXP1, PSMB8, SIRT2, ATXN1, INHBA, HHEX, TRPS1, HBZ, PHF21A**
*GO:0010558	Negative regulation of macromolecule biosynthetic process						25	6,70E-03	EIF2C1, TP63, NR2E3, TNFRSF4, CITED2, WWP1, RARB, KDM5B, CEBPA, DNMT3A, TBL1XR1, SMAD7, LDB1, ARID5B, KLF11, ELANE, PTPRU, SIRT2, FOXP1, ATXN1, HHEX, INHBA, TRPS1, HBZ, PHF21A
**GO:0009611**	**Response to wounding**						**33**	**5,54E-06**	**ABCF1, ITGAL, PLDN, MASP1, F13A1, CXCL2, BDKRB1, ABHD2, TNFRSF4, DTNBP1, CCL4, TPM1, CFP, GPX1, IL17D, CXCR4, IL10RB, KLKB1, IL1B, NFATC3, SELP, CEBPB, APCS, IL8, LY96, IL9, STXBP1, CHST3, IGF2, SOD2, PROC, LYVE1, GNAQ**
*GO:0006954	Inflammatory response						21	2,62E-04	ABCF1, SELP, ITGAL, APCS, CEBPB, MASP1, IL8, LY96, CXCL2, IL9, IGF2, BDKRB1, TNFRSF4, CCL4, CFP, IL17D, CXCR4, IL10RB, KLKB1, IL1B, NFATC3
	*GO:0050727	Regulation of inflammatory response					7	1,29E-02	GPX2, GPX1, MASP1, STAT5A, KLKB1, ELANE, IGF2
		*GO:0050728	Negative regulation of inflammatory response				4	4,37E-02	GPX2, GPX1, ELANE, IGF2
GO:0030888	Regulation of B cell proliferation						4	3,34E-02	CDKN1A, TNFSF13B, IL13RA1, TNFRSF4
	*GO:0030890	Positive regulation of B cell proliferation					4	1,35E-02	CDKN1A, TNFSF13B, IL13RA1, TNFRSF4
**GO:0051173**	**Positive regulation of nitrogen compound metabolic process**						**32**	**5,21E-04**	**STAT5A, NFKBIA, TP63, FOXO3, NR2E3, CITED2, CHD8, APP, MDFIC, IL1B, KDM3A, RARB, INSR, NFATC3, CEBPA, TBL1XR1, CEBPB, KLF13, IGF2, TBR1, SOD2, RNF8, ATF6, ATXN1, INHBA, HHEX, EP300, ETS1, NCOA4, MED17, IRF1, JAK3**
*GO:0045935	Positive regulation of nucleobase, nucleoside, nucleotide and nucleic acid metabolic process						30	1,35E-03	STAT5A, NFKBIA, TP63, FOXO3, NR2E3, CITED2, APP, CHD8, MDFIC, KDM3A, RARB, INSR, NFATC3, CEBPA, TBL1XR1, CEBPB, KLF13, IGF2, TBR1, ATF6, RNF8, ATXN1, HHEX, INHBA, EP300, NCOA4, ETS1, MED17, IRF1, JAK3

**GO:0016265**	**Death**				**29**	**1,82E-02**	**PHLPP1, NUAK2, NFKBIA, TP63, FOXO3, PMAIP1, GPX1, PEA15, TNFRSF11B, APP, CXCR4, IL1B, CASP1, TRAF3, CLN3, ARHGEF3, AIFM2, KLF11, APTX, IGF2, IL24, SOD2, DAPK1, ATXN1, SFRP5, EP300, HIPK3, TNFAIP3, DRAM1**
*GO:0008219	Cell death				29	1,70E-02	PHLPP1, NUAK2, NFKBIA, TP63, FOXO3, PMAIP1, GPX1, PEA15, TNFRSF11B, APP, CXCR4, IL1B, CASP1, TRAF3, CLN3, ARHGEF3, AIFM2, KLF11, APTX, IGF2, IL24, SOD2, DAPK1, ATXN1, SFRP5, EP300, HIPK3, TNFAIP3, DRAM1
	*GO:0012501	Programmed cell death			25	2,39E-02	PHLPP1, NUAK2, TP63, NFKBIA, FOXO3, PMAIP1, GPX1, PEA15, TNFRSF11B, APP, CXCR4, IL1B, CASP1, TRAF3, ARHGEF3, AIFM2, KLF11, IL24, SOD2, DAPK1, SFRP5, EP300, HIPK3, TNFAIP3, DRAM1
	*GO:0006915	Apoptosis			25	2,05E-02	PHLPP1, NUAK2, TP63, NFKBIA, FOXO3, PMAIP1, GPX1, PEA15, TNFRSF11B, APP, CXCR4, IL1B, CASP1, TRAF3, ARHGEF3, AIFM2, KLF11, IL24, SOD2, DAPK1, SFRP5, EP300, HIPK3, TNFAIP3, DRAM1
		*GO:0008637	Apoptotic mitochondrial changes		4	4,33E-02	GPX1, AIFM2, PMAIP1, SOD2
**GO:0032268**	**Regulation of cellular protein metabolic process**				**27**	**2,10E-04**	**EIF2C1, MASP1, NFKBIA, BDKRB1, MTIF3, ZFP36L1, APP, APOA1, MDFIC, MAP3K2, KLKB1, SERPINB10, IL1B, EIF2B2, INSR, SAMD4A, CLN3, SMAD7, SOCS1, IGF2, PSMB8, EIF4G1, PPP1R9B, EIF4G3, EP300, HIPK3, SH3D19**
*GO:0051248	Negative regulation of protein metabolic process				10	4,97E-02	CLN3, EIF2C1, MASP1, SMAD7, SOCS1, SERPINB10, BDKRB1, IGF2, INSR, PSMB8
	*GO:0032269	Negative regulation of cellular protein metabolic process			10	4,07E-02	CLN3, EIF2C1, MASP1, SMAD7, SOCS1, SERPINB10, BDKRB1, IGF2, INSR, PSMB8
*GO:0030162	Regulation of proteolysis				9	5,37E-05	CLN3, EP300, MASP1, SMAD7, KLKB1, SERPINB10, IL1B, IGF2, SH3D19
	*GO:0045862	Positive regulation of proteolysis			5	3,44E-03	EP300, SMAD7, KLKB1, IL1B, SH3D19
	*GO:0045861	Negative regulation of proteolysis			4	1,75E-02	CLN3, MASP1, SERPINB10, IGF2
**GO:0009890**	**Negative regulation of biosynthetic process**				**26**	**4,86E-03**	**EIF2C1, TP63, NR2E3, TNFRSF4, CITED2, WWP1, RARB, KDM5B, CEBPA, DNMT3A, TBL1XR1, SMAD7, LDB1, ARID5B, KLF11, ELANE, IGF2, PTPRU, SIRT2, FOXP1, ATXN1, HHEX, INHBA, TRPS1, HBZ, PHF21A**
*GO:0031327	Negative regulation of cellular biosynthetic process				26	6,15E-03	EIF2C1, TP63, NR2E3, TNFRSF4, CITED2, WWP1, RARB, KDM5B, CEBPA, DNMT3A, TBL1XR1, SMAD7, LDB1, ARID5B, KLF11, ELANE, IGF2, PTPRU, SIRT2, FOXP1, ATXN1, HHEX, INHBA, TRPS1, HBZ, PHF21A
**GO:0010629**	**Negative regulation of gene expression**				**24**	**4,98E-03**	**CEBPA, DNMT3A, TBL1XR1, EIF2C1, SMAD7, ARID5B, LDB1, KLF11, TP63, NR2E3, PTPRU, TNFRSF4, FOXP1, SIRT2, CITED2, ATXN1, HHEX, WWP1, TRPS1, HBZ, PHF21A, RARB, INSR, KDM5B**
*GO:0016481	Negative regulation of transcription				22	7,05E-03	CEBPA, DNMT3A, TBL1XR1, SMAD7, ARID5B, LDB1, KLF11, TP63, NR2E3, PTPRU, TNFRSF4, SIRT2, FOXP1, CITED2, ATXN1, HHEX, WWP1, TRPS1, HBZ, PHF21A, RARB, KDM5B
	*GO:0045892	Negative regulation of transcription, DNA-dependent			19	4,48E-03	CEBPA, DNMT3A, TBL1XR1, SMAD7, ARID5B, LDB1, KLF11, TP63, NR2E3, TNFRSF4, SIRT2, FOXP1, CITED2, HHEX, TRPS1, HBZ, PHF21A, RARB, KDM5B
**GO:0033554**	**Cellular response to stress**				**24**	**1,90E-02**	**MORF4L1, RAD23B, CLN3, NUAK2, POLG, CHST3, APTX, TP63, BCCIP, FOXO3, RAD52, TPM1, SOD2, RNF8, ATF6, GPX1, CDKN1A, HIPK1, MDFIC, MAP3K2, FANCD2, UBR5, MYOF, MAP3K13**
*GO:0006979	Response to oxidative stress				11	9,21E-03	ANGPTL7, GPX2, GPX1, EP300, PTGS1, APTX, CA3, NQO1, TPM1, GCLM, SOD2
*GO:0006952	Defense response				25	2,56E-02	ABCF1, ITGAL, MASP1, CXCL2, BDKRB1, TNFRSF4, CCL4, CFP, IL17D, IL10RB, CXCR4, KLKB1, IL1B, NOS2, TNIP1, NFATC3, SELP, APCS, CEBPB, IL8, CEBPE, LY96, IL9, IGF2, INHBA
**GO:0051172**	**Negative regulation of nitrogen compound metabolic process**				**22**	**2,55E-02**	**CEBPA, DNMT3A, TBL1XR1, SMAD7, ARID5B, LDB1, KLF11, TP63, NR2E3, PTPRU, TNFRSF4, SIRT2, FOXP1, CITED2, ATXN1, HHEX, WWP1, TRPS1, HBZ, PHF21A, RARB, KDM5B**
*GO:0045934	Negative regulation of nucleobase, nucleoside, nucleotide and nucleic acid metabolic process				22	2,15E-02	CEBPA, DNMT3A, TBL1XR1, SMAD7, ARID5B, LDB1, KLF11, TP63, NR2E3, PTPRU, TNFRSF4, SIRT2, FOXP1, CITED2, ATXN1, HHEX, WWP1, TRPS1, HBZ, PHF21A, RARB, KDM5B
	*GO:0051253	Negative regulation of RNA metabolic process			19	5,29E-03	CEBPA, DNMT3A, TBL1XR1, SMAD7, ARID5B, LDB1, KLF11, TP63, NR2E3, TNFRSF4, SIRT2, FOXP1, CITED2, HHEX, TRPS1, HBZ, PHF21A, RARB, KDM5B
**GO:0051338**	**Regulation of transferase activity**				**22**	**5,87E-04**	**CCNK, CCNT1, ELANE, BCCIP, IGF2, BDKRB1, CDC25C, CDKN3, TNFRSF4, APLP2, APP, CDKN1A, APOA1, PRLR, CXCR4, MDFIC, MAP3K2, HIPK3, IL1B, ADRA2B, INSR, MAP3K13**
**GO:0006928**	**Cell motion**				**21**	**1,95E-02**	**SELP, ITGAL, ZFAND5, TLN1, IL8, ARID5B, ELANE, ARPC5, CCL4, TPM1, TBR1, ARPC1B, GPX1, EPHA4, LYVE1, APP, APOA1, CXCR4, ETS1, IL1B, MSN**
*GO:0051270	Regulation of cell motion				13	3,89E-03	SELP, SMAD7, CHST3, IGF2, BDKRB1, ABHD2, PTPRU, TPM1, CITED2, ETS1, CLIC4, CXCR4, INSR
	*GO:0030334	Regulation of cell migration			11	1,12E-02	SELP, CXCR4, SMAD7, CLIC4, ABHD2, BDKRB1, IGF2, PTPRU, INSR, TPM1, CITED2
		*GO:0030336	Negative regulation of cell migration		6	1,47E-02	SMAD7, CLIC4, ABHD2, PTPRU, TPM1, CITED2
		**GO:0051271	Negative regulation of cell motion		6	2,18E-02	SMAD7, CLIC4, ABHD2, PTPRU, TPM1, CITED2
**GO:0051276**	**Chromosome organization**				**20**	**4,15E-02**	**MORF4L1, SUPT3H, DNMT3A, TBL1XR1, DPF3, KAT2B, AIFM2, NR3C1, SIRT2, RNF8, CHD8, EP300, KDM2A, FANCD2, CABIN1, PHF21A, KDM3A, KDM5B, CHD3, SUV420H1**
*GO:0006325	Chromatin organization				18	1,69E-02	MORF4L1, SUPT3H, DNMT3A, TBL1XR1, DPF3, KAT2B, NR3C1, SIRT2, RNF8, CHD8, EP300, KDM2A, CABIN1, PHF21A, KDM3A, KDM5B, CHD3, SUV420H1
	*GO:0016568	Chromatin modification			18	6,57E-04	MORF4L1, SUPT3H, DNMT3A, TBL1XR1, DPF3, KAT2B, NR3C1, SIRT2, RNF8, CHD8, EP300, KDM2A, CABIN1, PHF21A, KDM3A, KDM5B, CHD3, SUV420H1
		*GO:0016569	Covalent chromatin modification		9	1,51E-02	RNF8, MORF4L1, SUPT3H, DNMT3A, EP300, KAT2B, KDM3A, SIRT2, SUV420H1
			*GO:0016570	Histone modification	8	3,62E-02	RNF8, MORF4L1, SUPT3H, EP300, KAT2B, KDM3A, SIRT2, SUV420H1
**GO:0002684**	**Positive regulation of immune system process**				**19**	**3,97E-05**	**PLDN, MASP1, STAT5A, CD247, NFKBIA, BDKRB1, TNFRSF4, AQP3, FOXP1, CFP, CBLB, CDKN1A, TNFSF13B, IL4R, IL1B, CD79A, NOS2, IL13RA1, CD226**

*GO:0002697	Regulation of immune effector process			**10**	**1,09E-03**	**APOA1, MASP1, STAT5A, IL1B, IGF2, NOS2, IL13RA1, TNFRSF4, CD226, FOXP1**
	*GO:0002699	Positive regulation of immune effector process		**6**	**5,03E-03**	**STAT5A, IL1B, NOS2, IL13RA1, CD226, FOXP1**
**GO:0032774**	**RNA biosynthetic process**			**17**	**3,81E-03**	**BATF3, CEBPA, SUPT3H, CCNK, CEBPB, KLF13, CCNT1, KLF11, NR3C1, NR2E3, PRDM4, ETS1, MED17, TRPS1, IRF1, GTF3C1, NFATC3**
*GO:0006351	Transcription, DNA-dependent			17	3,33E-03	BATF3, CEBPA, SUPT3H, CCNK, CEBPB, KLF13, CCNT1, KLF11, NR3C1, NR2E3, PRDM4, ETS1, MED17, TRPS1, IRF1, GTF3C1, NFATC3
	*GO:0006366	Transcription from RNA polymerase II promoter		16	9,69E-04	BATF3, CEBPA, SUPT3H, CCNK, CEBPB, KLF13, CCNT1, KLF11, NR2E3, NR3C1, PRDM4, ETS1, MED17, TRPS1, IRF1, NFATC3
**GO:0051726**	**Regulation of cell cycle**			**18**	**4,99E-03**	**CCNK, STAT5A, CCNT1, BCCIP, IGF2, CDC25C, CDKN3, SIRT2, CITED2, CSNK2A2, PPP1R9B, INHBA, APP, CDKN1A, PRDM4, ETS1, IL1B, INSR**
*GO:0045787	Positive regulation of cell cycle			6	4,95E-02	APP, STAT5A, TGFA, IGF2, INSR, CITED2
**GO:0040008**	**Regulation of growth**			**16**	**2,82E-02**	**MORF4L1, CGA, STAT5A, SOCS1, TP63, BDKRB1, TKT, IGF2, DDR2, PPP1R9B, INHBA, CDKN1A, APP, EP300, PRDM4, INSR**
**GO:0046903**	**Secretion**			**14**	**4,35E-02**	**SNAP29, SCAMP1, CGA, PLDN, LTBP2, STX2, FFAR1, STXBP1, AQP3, ABCG8, TNFSF13B, SYN1, SCFD2, RAB14**
*GO:0032940	Secretion by cell			12	1,70E-02	SCAMP1, SNAP29, CGA, PLDN, TNFSF13B, STX2, SYN1, LTBP2, SCFD2, FFAR1, STXBP1, RAB14
	*GO:0050707	Regulation of cytokine secretion		4	3,99E-02	APOA1, IGF2, CASP1, TNFRSF4
**GO:0040012**	**Regulation of locomotion**			**13**	**3,73E-03**	**SELP, IL8, CXCR4, SMAD7, CLIC4, ELANE, ABHD2, BDKRB1, IGF2, PTPRU, INSR, TPM1, CITED2**
*GO:0040013	Negative regulation of locomotion			7	4,49E-03	SMAD7, CLIC4, ELANE, ABHD2, PTPRU, TPM1, CITED2
**GO:0006916**	**Anti-apoptosis**			**14**	**2,44E-03**	**CEBPB, STAT5A, TP63, NFKBIA, DAPK1, CITED2, SOD2, PEA15, GPX1, TNFSF13B, PRLR, HIPK3, IL1B, TNFAIP3**
**GO:0007507**	**Heart development**			**13**	**8,99E-03**	**SMAD7, PDLIM3, TPM1, FOXP1, SOD2, CITED2, HHEX, EP300, GNAQ, PLN, RARB, NFATC3, INSR**
**GO:0003006**	**Developmental process involved in reproduction**			**13**	**3,60E-02**	**CGA, DZIP1, STAT5A, TP63, FOXO3, NR2C2, CITED2, INHBA, CXCR4, NCOA4, EIF2B2, LHB, INSR**
*GO:0048608	Reproductive structure development			8	4,19E-02	CGA, INHBA, NCOA4, STAT5A, TP63, FOXO3, EIF2B2, LHB
	*GO:0045137	Development of primary sexual characteristics		8	4,34E-02	CGA, INHBA, NCOA4, STAT5A, TP63, FOXO3, EIF2B2, LHB
**GO:0050865**	**Regulation of cell activation**			13	**1,78E-03**	**SELP, INHBA, CDKN1A, CBLB, PLDN, SIT1, TNFSF13B, STAT5A, IL4R, IL1B, IL13RA1, TNFRSF4, CD226**
*GO:0002694	Regulation of leukocyte activation			12	3,55E-03	INHBA, CDKN1A, CBLB, PLDN, SIT1, TNFSF13B, STAT5A, IL4R, IL1B, IL13RA1, TNFRSF4, CD226
	**GO:0051249	Regulation of lymphocyte activation		11	4,65E-03	INHBA, CDKN1A, CBLB, PLDN, SIT1, TNFSF13B, STAT5A, IL4R, IL1B, IL13RA1, TNFRSF4
		*GO:0050864	Regulation of B cell activation	6	9,31E-03	INHBA, CDKN1A, TNFSF13B, STAT5A, IL13RA1, TNFRSF4
GO:0007517	Muscle organ development			12	1,93E-02	FAM65B, ZFAND5, APP, EP300, ACTA1, SMAD7, PLN, ARID5B, TP63, RARB, TPM1, FOXP1
***GO:0060537**	**Muscle tissue development**			**10**	**4,62E-03**	**ZFAND5, APP, EP300, ACTA1, SMAD7, PLN, TP63, RARB, TPM1, FOXP1**
	*GO:0014706	Striated muscle tissue development		8	3,22E-02	APP, EP300, ACTA1, SMAD7, PLN, RARB, TPM1, FOXP1
	*GO:0048745	Smooth muscle tissue development		3	2,06E-02	ZFAND5, TP63, FOXP1
**GO:0009991**	**Response to extracellular stimulus**			**12**	**2,53E-02**	**PPP1R9B, CLN3, CDKN1A, TNFRSF11B, ACSL1, ACTA1, NUAK2, GDAP1, SLC22A3, INSR, AQP3, SOD2**
**GO:0030029**	**Actin filament-based process**			**12**	**4,44E-02**	**PPP1R9B, TLN1, SSH1, ACTA1, NUAK2, DIAPH1, SSH2, CDC42BPA, PDLIM3, MYO9B, ARPC5, TPM1**
*GO:0030036	Actin cytoskeleton organization			12	3,00E-02	PPP1R9B, TLN1, SSH1, ACTA1, NUAK2, DIAPH1, SSH2, CDC42BPA, PDLIM3, MYO9B, ARPC5, TPM1
**GO:0030097**	**Hemopoiesis**			**12**	**3,94E-02**	**CEBPA, CSF3, INHBA, SNRK, CEBPE, STAT5A, HBZ, IRF1, DYRK3, CD79A, FOXP1, SOD2**
**GO:0032844**	**Regulation of homeostatic process**			**10**	**2,49E-03**	**INHBA, TNFRSF11B, ETS1, SMAD7, LDB1, STAT5A, BDKRB1, FOXO3, SLC22A5, GCLM**
*GO:0001817	Regulation of cytokine production			11	1,75E-02	INHBA, CEBPB, APOA1, STAT5A, IL9, ELANE, IRF1, IGF2, CASP1, TNFRSF4, IFNAR1
**GO:0010035**	**Response to inorganic substance**			**11**	**3,70E-02**	**GPX1, CDKN1A, TNFRSF11B, EP300, SLC6A1, CYBRD1, APTX, INSR, TPM1, AQP3, SOD2**
**GO:0010608**	**Posttranscriptional regulation of gene expression**			**11**	**4,36E-02**	**ZFP36L1, EIF4G1, EIF4G3, APP, APOA1, EIF2C1, SMAD7, APTX, EIF2B2, MTIF3, SAMD4A**
**GO:0005976**	**Polysaccharide metabolic process**			**10**	**2,08E-03**	**LYVE1, PPP1R2, SLC35D1, PYGM, PYGL, CSGALNACT2, CHI3L1, CHST3, MAT2B, PPP1CC**
*GO:0044264	Cellular polysaccharide metabolic process			6	1,09E-02	PPP1R2, PYGM, PYGL, CSGALNACT2, MAT2B, PPP1CC
*GO:0000272	Polysaccharide catabolic process			4	3,34E-02	LYVE1, PYGM, PYGL, CHI3L1
**GO:0006790**	**Sulfur metabolic process**			**10**	**2,65E-03**	**TPK1, GSR, GPX1, SLC35D1, STAT5A, CSGALNACT2, CHST3, MAT2B, GCLM, SOD2**
*GO:0044272	Sulfur compound biosynthetic process			6	8,57E-03	TPK1, SLC35D1, CSGALNACT2, CHST3, MAT2B, GCLM
	*GO:0050650	Chondroitin sulfate proteoglycan biosynthetic process		3	3,05E-02	SLC35D1, CSGALNACT2, CHST3
*GO:0006749	Glutathione metabolic process			4	3,34E-02	GSR, GPX1, GCLM, SOD2
GO:0003013	Circulatory system process			10	4,84E-02	GPX1, RENBP, PLN, PTGS1, BDKRB1, NOS2, MYOF, TPM1, GCLM, SOD2
*GO:0008015	Blood circulation			10	4,84E-02	GPX1, RENBP, PLN, PTGS1, BDKRB1, NOS2, MYOF, TPM1, GCLM, SOD2
**GO:0050867**	**Positive regulation of cell activation**			**9**	**7,36E-03**	**SELP, CDKN1A, PLDN, TNFSF13B, STAT5A, IL4R, IL13RA1, TNFRSF4, CD226**
*GO:0002696	Positive regulation of leukocyte activation			8	1,85E-02	CDKN1A, PLDN, TNFSF13B, STAT5A, IL4R, IL13RA1, TNFRSF4, CD226
	*GO:0051251	Positive regulation of lymphocyte		7	3,72E-02	CDKN1A, PLDN, TNFSF13B, STAT5A, IL4R, IL13RA1, TNFRSF4
		*GO:0042110	T cell activation	9	1,51E-02	ITGAL, CBLB, PRLR, CXCR4, STAT5A, IRF1, IGF2, TNFRSF4, IFNAR1
		*GO:0050871	Positive regulation of B cell activation	5	9,40E-03	CDKN1A, TNFSF13B, STAT5A, IL13RA1, TNFRSF4

**GO:0002683**	**Negative regulation of immune system process**		**7**	**1,90E-02**	**GPX2, INHBA, GPX1, CBLB, MASP1, IL4R, IGF2**
**GO:0030099**	**Myeloid cell differentiation**		**7**	**3,11E-02**	**CEBPA, CSF3, INHBA, SNRK, CEBPE, HBZ, DYRK3**
*GO:0045637	Regulation of myeloid cell differentiation		6	3,26E-02	INHBA, ETS1, LDB1, STAT5A, NFKBIA, FOXO3
	*GO:0045638	Negative regulation of myeloid cell differentiation	4	3,99E-02	INHBA, LDB1, STAT5A, NFKBIA
**GO:0048585**	**Negative regulation of response to stimulus**		**7**	**4,21E-02**	**GPX2, CLN3, GPX1, MASP1, SOCS1, ELANE, IGF2**
*GO:0032102	Negative regulation of response to external stimulus		5	3,79E-02	GPX2, CLN3, GPX1, ELANE, IGF2
*GO:0050777	Negative regulation of immune response		4	5,61E-02	GPX2, GPX1, MASP1, IGF2
**GO:0050817**	**Coagulation**		**7**	**4,57E-02**	**PLDN, GNAQ, KLKB1, F13A1, STXBP1, DTNBP1, PROC**
*GO:0007596	Blood coagulation		7	4,57E-02	PLDN, GNAQ, KLKB1, F13A1, STXBP1, DTNBP1, PROC
**GO:0007050**	**Cell cycle arrest**		**7**	**4,75E-02**	**PPP1R9B, INHBA, CDKN1A, KAT2B, IL8, CDKN3, UHMK1**
**GO:0042035**	**Regulation of cytokine biosynthetic process**		**6**	**4,00E-02**	**INHBA, CEBPB, STAT5A, IL9, ELANE, IRF1**
**GO:0045646**	**Regulation of erythrocyte differentiation**		**5**	**9,48E-04**	**INHBA, ETS1, LDB1, STAT5A, FOXO3**
*GO:0045648	Positive regulation of erythrocyte differentiation		3	3,05E-02	INHBA, ETS1, FOXO3
**GO:0018212**	**Peptidyl-tyrosine modification**		**5**	**3,33E-02**	**PRLR, STAT5A, JAK3, INSR, DDR2**
*GO:0018108	Peptidyl-tyrosine phosphorylation		5	2,90E-02	PRLR, STAT5A, JAK3, INSR, DDR2
**GO:0009896**	**Positive regulation of catabolic process**		**5**	**3,55E-02**	**CBLB, SMAD7, IGF2, SH3D19, INSR**
**GO:0045444**	**Fat cell differentiation**		**5**	**4,54E-02**	**CEBPA, GPX1, CEBPB, SOCS1, INSR**
**GO:0001889**	**Liver development**		**5**	**4,54E-02**	**CEBPA, HHEX, EP300, SOD2, CITED2**
**GO:0045682**	**Regulation of epidermis development**		**4**	**1,75E-02**	**INHBA, TP63, KEAP1, AQP3**
**GO:0015695**	**Organic cation transport**		**4**	**4,55E-02**	**RHCG, SLC12A2, SLC22A3, SLC22A5**
**GO:0000272**	**Polysaccharide catabolic process**		**4**	**3,34E-02**	**LYVE1, PYGM, PYGL, CHI3L1**
**GO:0030856**	**Regulation of epithelial cell differentiation**		**4**	**3,66E-02**	**PRLR, STAT5A, TP63, AQP3**
**GO:0022406**	**Membrane docking**		**4**	**4,33E-02**	**PLDN, SCFD2, STXBP1, MSN**
**GO:0030325**	**Adrenal gland development**		**3**	**2,54E-02**	**APOA1, NR3C1, CITED2**
**GO:0044058**	**Regulation of digestive system process**		**3**	**3,05E-02**	**ABCG8, APOA1, SLC22A5**
**GO:0045604**	**Regulation of epidermal cell differentiation**		**3**	**4,80E-02**	**TP63, KEAP1, AQP3**

*Subset **Part of
