# Supplementary material for: Transcriptomic Analysis of Porcine Endometrium during Implantation after In Vitro Stimulation by Adiponectin
Source: Int J Mol Sci. 2019 Mar 16;20(6):1335. doi: 10.3390/ijms20061335 (PMC6470965; doi:10.3390/ijms20061335)
Supplement: Supplementary file 1 [file ijms-20-01335-s001.zip › SF.4..pdf]

Supplementary Table 4. The most significantly enriched gene ontologies (biological process)

GO - Biological Process						Count	p-value	Altered genes
		**Up-regulated**						
**GO:0010467**	**Gene expression**					**47**	**4,67E-04**	**MTDH, COPS5, CPEB2, TDRD7, UBE3A, ZBTB11, NAA15, SYNCRIP, RPS6KB1, ENPEP, NEO1, ZNF518A, MYBL2, VARS, NFXL1, KDM1B, SF3B1, TFAM, CDC45, CHD2, LEO1, MYCBP, ZNF445, FANCA, HELLS, CHUK, GNL3, BCL9, TAF2, ABCE1, SNAPC5, TAF5, MPHOSPH10, ZNF667, DENR, ATM, PAPOLA, EIF2S1, UCHL5, WDR3, NOP58, NOP56, CUX2, PHEX, ZBTB1, BMPR1A**
*GO:0006351	Transcription, DNA-templated					26	1,53E-02	MTDH, COPS5, UBE3A, NAA15, NEO1, NFXL1, MYBL2, ZNF518A, TFAM, CDC45, LEO1, ZNF445, FANCA, CHUK, HELLS, BCL9, GNL3, TAF2, SNAPC5, TAF5, ZNF667, UCHL5, CUX2, ZBTB1, BMPR1A
**GO:0016070**	**RNA metabolic process**					**39**	**2,18E-03**	**MTDH, COPS5, UBE3A, ZBTB11, NAA15, NEO1, ZNF518A, MYBL2, VARS, NFXL1, SF3B1, TFAM, CDC45, CHD2, LEO1, MYCBP, ZNF445, FANCA, HELLS, CHUK, GNL3, BCL9, TAF2, SNAPC5, TAF5, MPHOSPH10, ZNF667, ATM, PAPOLA, UCHL5, WDR3, NOP58, NOP56, CUX2, PHEX, ZBTB1, BMPR1A, KIAA1429**
*GO:0032774	RNA biosynthetic process					30	9,41E-03	MTDH, COPS5, UBE3A, ZBTB11, NAA15, NEO1, MYBL2, NFXL1, ZNF518A, TFAM, CDC45, CHD2, LEO1, MYCBP, ZNF445, FANCA, CHUK, HELLS, GNL3, BCL9, TAF2, SNAPC5, TAF5, ZNF667, UCHL5,
	*GO:0097659	Nucleic acid-templated transcription				30	8,73E-03	MTDH, COPS5, UBE3A, ZBTB11, NAA15, NEO1, MYBL2, NFXL1, ZNF518A, TFAM, CDC45, CHD2, LEO1, MYCBP, ZNF445, FANCA, CHUK, HELLS, GNL3, BCL9, TAF2, SNAPC5, TAF5, ZNF667, UCHL5,
**GO:0010468**	**Regulation of gene expression**					**33**	**1,05E-02**	**MTDH, CPEB2, COPS5, UBE3A, TDRD7, ZBTB11, NAA15, SYNCRIP, RPS6KB1, NEO1, MYBL2, KDM1B, TFAM, CDC45, CHD2, LEO1, MYCBP, FANCA, CHUK, HELLS, GNL3, BCL9, TAF2, ZNF667, ATM, PAPOLA, EIF2S1, UCHL5, CUX2, PHEX, ZBTB1, BMPR1A**
*GO:0006355	Regulation of transcription, DNA-templated					24	8,44E-02	TAF2, MTDH, COPS5, UBE3A, ZBTB11, ZNF667, NAA15, NEO1, MYBL2, TFAM, CDC45, UCHL5, CHD2, LEO1, MYCBP, PHEX, FANCA, ZBTB1, HELLS, CHUK, BMPR1A, GNL3, BCL9
	GO:1902895	Positive regulation of pri-miRNA transcription from RNA polymerase II promoter				2	9,85E-02	BMPR1A, GNL3
**GO:0051171**	**Regulation of nitrogen compound metabolic process**					**33**	**1,18E-02**	**MTDH, CPEB2, COPS5, UBE3A, ZBTB11, NAA15, SYNCRIP, RPS6KB1, NEO1, MYBL2, KDM1B, TFAM, CDC45, CHD2, LEO1, MYCBP, ACSL3, FANCA, CHUK, HELLS, GNL3, BCL9, TAF2, ZNF667, SAMD8, PAPOLA, EIF2S1, PARPBP, UCHL5, PHEX, ZBTB1, BMPR1A**
*GO:0019219	Regulation of nucleobase-containing compound metabolic process					27	9,97E-02	MTDH, COPS5, UBE3A, ZBTB11, NAA15, NEO1, MYBL2, KDM1B, TFAM, CDC45, CHD2, LEO1, MYCBP, FANCA, HELLS, CHUK, BCL9, GNL3, TAF2, ZNF667, PAPOLA, PARPBP, UCHL5, PHEX, ZBTB1, BMPR1A
	*GO:0051252	Regulation of RNA metabolic process				25	8,59E-02	TAF2, MTDH, COPS5, UBE3A, ZBTB11, ZNF667, NAA15, NEO1, MYBL2, TFAM, CDC45, PAPOLA, UCHL5, CHD2, LEO1, MYCBP, PHEX, FANCA, ZBTB1, HELLS, CHUK, BMPR1A, GNL3, BCL9
		*GO:2001141	Regulation of RNA biosynthetic process			24	8,84E-02	TAF2, MTDH, COPS5, UBE3A, ZBTB11, ZNF667, NAA15, NEO1, MYBL2, TFAM, CDC45, UCHL5, CHD2, LEO1, MYCBP, PHEX, FANCA, ZBTB1, HELLS, CHUK, BMPR1A, GNL3, BCL9
			*GO:1903506	Regulation of nucleic acid-templated transcription		24	8,62E-02	TAF2, MTDH, COPS5, UBE3A, ZBTB11, ZNF667, NAA15, NEO1, MYBL2, TFAM, CDC45, UCHL5, CHD2, LEO1, MYCBP, PHEX, FANCA, ZBTB1, HELLS, CHUK, [illegible]
**GO:0010556**	**Regulation of macromolecule biosynthetic process**					**31**	**1,23E-02**	**MTDH, SEC24A, ENPP1, CPEB2, COPS5, UBE3A, ZBTB11, NAA15, SYNCRIP, RPS6KB1, NEO1, MYBL2, TFAM, CDC45, CHD2, LEO1, MYCBP, FANCA, CHUK, HELLS, GNL3, BCL9, TAF2, ZNF667, SAMD8, EIF2S1, UCHL5, PHEX, ZBTB1, BMPR1A**
*GO:2000112	Regulation of cellular macromolecule biosynthetic process					29	2,69E-02	MTDH, CPEB2, COPS5, ENPP1, UBE3A, ZBTB11, NAA15, SYNCRIP, RPS6KB1, NEO1, MYBL2, TFAM, CDC45, CHD2, LEO1, MYCBP, FANCA, CHUK, HELLS, GNL3, BCL9, TAF2, ZNF667, EIF2S1, UCHL5, PHEX, ZBTB1, BMPR1A
**GO:0044248**	**Cellular catabolic process**					**15**	**6,80E-02**	**NUDT12, ENPP1, UBE3A, SOCS4, UBR1, ENPEP, CLPX, ATM, DDHD2, CUL5, UCHL5, CROT, ETFA, HECTD1**
**GO:0016192**	**Vesicle-mediated transport**					**14**	**2,05E-02**	**STX1A, SEC24A, COPS5, AP4E1, UBE3A, EEA1, HOOK1, DDHD2, SGSM1, ANKRD50, ATP9A, VPS26A, ABL2, GOLGA4**
**GO:0006259**	**DNA metabolic process**					**12**	**2,25E-02**	**KDM1B, CDC45, DCLRE1A, PARPBP, MCMBP, UCHL5, HSPD1, ZBTB1, HELLS, ATM, GNL3**
*GO:0006281	DNA repair					7	8,22E-02	CDC45, DCLRE1A, PARPBP, UCHL5, ZBTB1, ATM
*GO:0006310	DNA recombination					5	6,27E-02	CDC45, PARPBP, UCHL5, HSPD1
**GO:0070647**	**Protein modification by small protein conjugation or removal**					**10**	5,23E-02	**CUL5, COPS5, UBE3A, RUSC1, UCHL5, LEO1, SOCS4, GNL3, HECTD1**
**GO:0009628**	**Response to abiotic stimulus**					**9**	8,85E-02	**RDH11, DDHD2, CPEB2, EIF2S1, TRPV2, RYR1, HSPD1, ZBTB1, ATM**
*GO:0071482	Cellular response to light stimulus					3	8,13E-02	RDH11, EIF2S1, ZBTB1
**GO:0022613**	**Ribonucleoprotein complex biogenesis**					**8**	**3,70E-02**	**SF3B1, ABCE1, MPHOSPH10, NOP58, WDR3, DENR, NOP56, GNL3**
*GO:0042254	Ribosome biogenesis					6	5,38E-02	ABCE1, MPHOSPH10, NOP58, WDR3, NOP56, GNL3
**GO:0009116**	**Nucleoside metabolic process**					**6**	**7,35E-02**	**ENPP1, DLD, OLA1, ATP5O, ATP8, CROT**
	GO:0042278	Purine nucleoside metabolic process				6	4,74E-02	ENPP1, DLD, OLA1, ATP5O, ATP8, CROT
	GO:0046128	Purine ribonucleoside metabolic process				6	4,57E-02	ENPP1, DLD, OLA1, ATP5O, ATP8, CROT
**GO:1901657**	**Glycosyl compound metabolic process**					**6**	**9,16E-02**	**ENPP1, DLD, OLA1, ATP5O, ATP8, CROT**
**GO:0009141**	**Nucleoside triphosphate metabolic process**					**5**	**9,23E-02**	**ENPP1, DLD, OLA1, ATP5O, ATP8**
**GO:0031346**	**Positive regulation of cell projection organization**					**5**	**4,74E-02**	**TRPV2, CUX2, GOLGA4, ABL2, NCKAP1**
*GO:0010976	Positive regulation of neuron projection development					4	6,75E-02	TRPV2, CUX2, GOLGA4, ABL2
**GO:0007160**	**Cell-matrix adhesion**					**4**	**9,02E-02**	**TMEM8B, SNED1, GPM6B, MUC4**
**GO:0006413**	**Translational initiation**					**4**	**2,18E-02**	**ABCE1, EIF2S1, RPS6KB1, DENR**
**GO:0031440**	**Regulation of mRNA 3'-end processing**					**2**	**7,96E-02**	**PAPOLA, LEO1**

			Down-regulated		Count	p-value	Altered genes
**GO:0010468**	**Regulation of gene expression**				**85**	**2,63E-04**	**MORF4L1, ABCF1, CPEB4, STAT5A, LOC100158121, CCNT1, FOXO3, NR2E3, LOC100627422, DDR2, CITED2, APP, MDFIC, WWP1, MSN, NOS2, RARB, EIF2B2, KDM5B, LOC100153329, SAMD4A, TBL1XR1, CCNK, ACTA1, LDB1, ARID5B, SAA3, TLE4, PPP1CC, TBR1, EIF4G1, INHBA, HHEX, EP300, FANCD2, MED17, TFAP2A, TNFAIP3, SBNO1, SAV1, NFKBIA, KEAP1, NR3C1, CCL4, DTNBP1, NR2C2, UHMK1, ZFP36L1, TNFRSF1B, HNRNPK, DDX46, MAP3K2, AGO1, KDM3A, PHF20L1, INPP5B, CHD3, TRAF3, CEBPA, ZNF398, IKZF4, DNMT3A, BRD2, KAT2B, CEBPB, SMAD7, KLF11, LOC100154071, FXR2, RBMX, FOXP1, RGS14, SIRT2, SOD2, SFRP5, ATF6, RNF8, ATXN1, RPS6KA3, ETS1, HBZ, IRF1, GRB7, RNF111**
*GO:0010628	Positive regulation of gene expression				41	1,14E-03	ABCF1, STAT5A, CCNT1, NFKBIA, NR2E3, NR3C1, DTNBP1, UHMK1, NR2C2, APP, MAP3K2, MDFIC, AGO1, KDM3A, RARB, MSN, KDM5B, SAMD4A, CEBPA, IKZF4, TBL1XR1, CCNK, KAT2B, CEBPB, ACTA1, LDB1, TBR1, RBMX, FOXP1, SIRT2, ATF6, HHEX, INHBA, RPS6KA3, EP300, ETS1, MED17, IRF1, TFAP2A, RNF111
*GO:0010629	Negative regulation of gene expression				36	1,77E-03	MORF4L1, CPEB4, LOC100158121, CCNT1, NR2E3, CCL4, LOC100627422, ZFP36L1, MDFIC, WWP1, AGO1, RARB, NOS2, KDM5B, LOC100153329, SAMD4A, CEBPA, DNMT3A, TBL1XR1, IKZF4, SMAD7, LDB1, KLF11, LOC100154071, SAA3, TLE4, FXR2, FOXP1, SIRT2, ATXN1, RNF8, EP300, HBZ, IRF1, TFAP2A, GRB7
	*GO:0016458	Gene silencing			6	9,91E-02	MORF4L1, DNMT3A, LOC100158121, AGO1, LOC100154071, LOC100153329
**GO:0009892**	**Negative regulation of metabolic process**				**56**	**5,83E-04**	**MORF4L1, CPEB4, CCNT1, LOC100158121, NR2E3, LOC100627422, APP, MDFIC, WWP1, NOS2, RARB, TNIP1, KDM5B, LOC100153329, SAMD4A, TBL1XR1, LDB1, SOCS1, ELANE, FBP1, SAA3, TLE4, HHEX, RENBP, EP300, HIPK3, SERPINB1, TFAP2A, TNFAIP3, AZIN1, DTNBP1, CCL4, ZFP36L1, LPCAT1, AGO1, KDM3A, TRIP12, CEBPA, DNMT3A, IKZF4, KAT2B, PCIF1, SMAD7, CBL, KLF11, LOC100154071, FXR2, SIRT2, RGS14, FOXP1, RNF8, ATXN1, CBLB, HBZ, IRF1, GRB7**
*GO:0031324	Negative regulation of cellular metabolic process				53	8,51E-04	MORF4L1, CPEB4, CCNT1, LOC100158121, NR2E3, LOC100627422, APP, MDFIC, WWP1, RARB, TNIP1, KDM5B, LOC100153329, SAMD4A, TBL1XR1, LDB1, SOCS1, ELANE, SAA3, FBP1, TLE4, HHEX, RENBP, EP300, HIPK3, SERPINB1, TFAP2A, TNFAIP3, DTNBP1, CCL4, ZFP36L1, AGO1, KDM3A, TRIP12, CEBPA, DNMT3A, IKZF4, KAT2B, PCIF1, SMAD7, CBL, KLF11, LOC100154071, FXR2, SIRT2, RGS14, FOXP1, RNF8, ATXN1, CBLB, HBZ, IRF1, GRB7
	*GO:0032269	Negative regulation of cellular protein metabolic process			23	3,08E-02	KAT2B, SMAD7, CPEB4, SOCS1, CBL, FXR2, DTNBP1, RGS14, SIRT2, ZFP36L1, HHEX, CBLB, RENBP, APP, HIPK3, AGO1, SERPINB1, KDM3A, TNFAIP3, TNIP1, GRB7, SAMD4A, TRIP12
**GO:0002682**	**Regulation of immune system process**				**41**	**4,64E-06**	**PHLPP1, AMCF-II, CSF3, MASP1, STAT5A, CXCL2, CD247, CXCL8, NFKBIA, FOXO3, CCL4, CCL28, AQP3, APOA1, IL4R, SERPINB10, MSN, TNIP1, LOC100621389, TRAF3, SELP, SIT1, LY96, KLF13, LDB1, ELANE, SOCS1, CHST3, FXR2, FOXP1, INHBA, RPS6KA3, TNFSF13B, ETS1, FANCD2, IRF1, JAK3, CD79A, TNFAIP3, JAM3**
*GO:0002684	Positive regulation of immune system process				27	2,00E-04	CSF3, AMCF-II, MASP1, STAT5A, CD247, CXCL2, CXCL8, NFKBIA, FOXO3, CCL4, AQP3, IL4R, TNIP1, TRAF3, SELP, LY96, ELANE, FOXP1, INHBA, RPS6KA3, TNFSF13B, ETS1, IRF1, JAK3, CD79A, TNFAIP3
*GO:0050776	Regulation of immune response				15	7,08E-02	MASP1, LY96, ELANE, CD247, SOCS1, NFKBIA, RPS6KA3, APOA1, TNFSF13B, IL4R, IRF1, CD79A, TNFAIP3, TNIP1, TRAF3
	*GO:0050778	Positive regulation of immune response			13	4,98E-02	RPS6KA3, TNFSF13B, MASP1, LY96, IL4R, ELANE, CD247, IRF1, NFKBIA, CD79A, TNFAIP3, TNIP1, TRAF3
		*GO:0002253	Activation of immune response		10	4,41E-02	RPS6KA3, MASP1, LY96, CD247, IRF1, NFKBIA, CD79A, TNFAIP3, TNIP1, TRAF3
*GO:1903706	Regulation of hemopoiesis				13	5,45E-03	CSF3, INHBA, ETS1, KLF13, FANCD2, IL4R, LDB1, STAT5A, IRF1, NFKBIA, FOXO3, LOC100621389
	*GO:0045637	Regulation of myeloid cell differentiation			10	3,42E-03	CSF3, INHBA, ETS1, KLF13, LDB1, STAT5A, NFKBIA, FOXO3, LOC100621389
		*GO:0045646	Regulation of erythrocyte differentiation		6	6,98E-04	INHBA, ETS1, KLF13, LDB1, STAT5A, FOXO3
			*GO:0045647	gulation of erythrocyte differentiation	3	1,52E-02	KLF13, LDB1, STAT5A
		*GO:0045639	Positive regulation of myeloid cell differentiation		5	5,84E-02	CSF3, INHBA, ETS1, FOXO3
		*GO:0045638	Negative regulation of myeloid cell differentiation		5	3,00E-02	KLF13, LDB1, STAT5A, NFKBIA, LOC100621389
	*GO:1903708	Positive regulation of hemopoiesis			7	5,62E-02	CSF3, INHBA, ETS1, IL4R, STAT5A, FOXO3
	*GO:1903707	Negative regulation of hemopoiesis			7	1,30E-02	KLF13, IL4R, LDB1, STAT5A, IRF1, NFKBIA, LOC100621389
*GO:0045088	Regulation of innate immune response				8	5,71E-02	RPS6KA3, LY96, SOCS1, IRF1, NFKBIA, TNFAIP3, TNIP1, TRAF3
	*GO:0045089	Positive regulation of innate immune response			7	5,39E-02	RPS6KA3, LY96, IRF1, NFKBIA, TNFAIP3, TNIP1, TRAF3
*GO:0002685	Regulation of leukocyte migration				8	2,28E-02	AMCF-II, SELP, CXCL2, CXCL8, MSN, JAM3, CCL28, CCL4
	*GO:0050901	Leukocyte tethering or rolling			3	5,77E-02	SELP, ITGB7, CCL28
	*GO:1902622	Regulation of neutrophil migration			3	9,02E-02	CXCL2, CXCL8, JAM3
		*GO:0090022	Regulation of neutrophil chemotaxis		3	7,34E-02	CXCL2, CXCL8, JAM3
**GO:0042127**	**Regulation of cell proliferation**				**38**	**2,44E-03**	**AMCF-II, CSF3, STAT5A, SAV1, CXCL2, PTGS1, CXCL8, NFKBIA, FOXO3, NR2E3, TNFRSF4, DDR2, P14ARF, TNFRSF11B, TNFRSF1B, RARB, NOS2, KDM5B, CEBPA, KAT2B, KLF13, ELANE, KLF11, LOC100154071, PTPRU, FOXP1, SIRT2, SOD2, SFRP5, HHEX, INHBA, PPP1R9B, TNFSF13B, IRF1, TFAP2A, JAK3, TNFAIP3**
*GO:0008285	Negative regulation of cell proliferation				17	2,19E-02	CEBPA, KAT2B, KLF13, SAV1, KLF11, LOC100154071, NR2E3, PTPRU, P14ARF, SIRT2, SOD2, SFRP5, INHBA, IRF1, TFAP2A, RARB,
**GO:0008283**	**Cell proliferation**				**36**	**4,26E-02**	**MORF4L1, CSF3, STAT5A, SAV1, FOXO3, NR2E3, DDR2, P14ARF, ZFP36L1, APOA1, SDR16C5, RARB, KDM5B, CEBPA, PDK1, KAT2B, KLF13, ELANE, KLF11, LOC100154071, PTPRU, FOXP1, SIRT2, SOD2, TACC2, SFRP5, HHEX, INHBA, TNFSF13B, IRF1, TFAP2A, TXNRD1, JAK3, CD79A, TNFAIP3**
**GO:0012501**	**Programmed cell death**				**35**	**6,62E-02**	**PHLPP1, NUAK2, SAV1, STAT5A, FOXO3, GCLM, TNFRSF4, P14ARF, CITED2, ZFP36L1, PEA15, APP, TNFRSF1B, TNFRSF11B, RARB, CASP1, TRAF3, PDK1, CLN3, ANP32B, AIFM2, CBL, KLF11, CHI3L1, SIRT2, PROC, SOD2, INHBA, EP300, PYGL, HIPK3, IRF1, TFAP2A, TNFAIP3, ARHGAP10**
**GO:0022610**	**Biological adhesion**				**29**	**9,07E-02**	**ITGAL, TLN1, ASS1, STAT5A, CCL28, WHAMM, CITED2, ZFP36L1, APP, APOA1, IL4R, ITGB7, MSN, TNIP1, MYOC, SELP, SIT1, ATP4B, SMAD7, LDB1, PTPRU, FOXP1, LYVE1, TNFSF13B, ETS1, FANCD2, IRF1, JAK3, JAM3**
*GO:0007155	Cell adhesion				29	8,77E-02	ITGAL, TLN1, ASS1, STAT5A, CCL28, WHAMM, CITED2, ZFP36L1, APP, APOA1, IL4R, ITGB7, MSN, TNIP1, MYOC, SELP, SIT1, ATP4B, SMAD7, LDB1, PTPRU, FOXP1, LYVE1, TNFSF13B, ETS1, FANCD2, IRF1, JAK3, JAM3

	*GO:0098602	Single organism cell adhesion			24	5,81E-03	ITGAL, SELP, TLN1, SIT1, ASS1, SMAD7, STAT5A, PTPRU, CCL28, FOXP1, CITED2, ZFP36L1, APOA1, TNFSF13B, ETS1, FANCD2, IL4R, ITGB7, IRF1, MSN, JAK3, TNIP1, JAM3, MYOC
		*GO:0016337	Single organismal cell-cell adhesion		23	4,83E-03	ITGAL, SELP, TLN1, SIT1, ASS1, SMAD7, STAT5A, PTPRU, CCL28, FOXP1, CITED2, ZFP36L1, APOA1, TNFSF13B, ETS1, FANCD2, IL4R, ITGB7, IRF1, MSN, JAK3, TNIP1, JAM3
	*GO:0098609	Cell-cell adhesion			23	3,38E-02	ITGAL, SELP, TLN1, SIT1, ASS1, SMAD7, STAT5A, PTPRU, CCL28, FOXP1, CITED2, ZFP36L1, APOA1, TNFSF13B, ETS1, FANCD2, IL4R, ITGB7, IRF1, MSN, JAK3, TNIP1, JAM3
		*GO:0007159	Leukocyte cell-cell adhesion		17	1,24E-02	ITGAL, SELP, SIT1, ASS1, STAT5A, CCL28, FOXP1, ZFP36L1, TNFSF13B, ETS1, FANCD2, IL4R, ITGB7, IRF1, MSN, JAK3, TNIP1
			*GO:0061756	Leukocyte adhesion to vascular endothelial cell	4	9,48E-03	SELP, ETS1, ITGB7, CCL28
	*GO:0007160	Cell-matrix adhesion			7	7,09E-02	ITGAL, ITGB7, LDB1, JAM3, CCL28, WHAMM, MYOC
**GO:0002520**	**Immune system development**				**28**	**2,26E-03**	**PHLPP1, CSF3, STAT5A, NFKBIA, FOXO3, CITED2, ZFP36L1, IL4R, LOC100621389, CEBPA, CEBPE, KLF13, LDB1, FOXP1, SOD2, INHBA, HHEX, EP300, ETS1, FANCD2, HBZ, IRF1, CD79A, JAK3, PIP4K2A, TNFAIP3, JAM3**
*GO:0048534	Hematopoietic or lymphoid organ development				26	4,61E-03	CSF3, STAT5A, NFKBIA, FOXO3, CITED2, ZFP36L1, IL4R, LOC100621389, CEBPA, CEBPE, KLF13, LDB1, FOXP1, SOD2, INHBA, HHEX, EP300, ETS1, FANCD2, HBZ, IRF1, CD79A, JAK3, PIP4K2A, JAM3
	*GO:0030097	Hemopoiesis			26	1,99E-03	CSF3, STAT5A, NFKBIA, FOXO3, CITED2, ZFP36L1, IL4R, LOC100621389, CEBPA, CEBPE, KLF13, LDB1, FOXP1, SOD2, INHBA, HHEX, EP300, ETS1, FANCD2, HBZ, IRF1, CD79A, JAK3, PIP4K2A, JAM3
		*GO:0030099	Myeloid cell differentiation		17	1,35E-04	CSF3, CEBPA, CEBPE, KLF13, STAT5A, LDB1, NFKBIA, FOXO3, CITED2, INHBA, HHEX, EP300, ETS1, HBZ, PIP4K2A, LOC100621389
			*GO:0030218	Erythrocyte differentiation	8	1,73E-03	INHBA, ETS1, KLF13, LDB1, STAT5A, HBZ, FOXO3, CITED2
			*GO:0061515	Myeloid cell development	4	5,15E-02	EP300, HBZ, PIP4K2A, CITED2
**GO:0032504**	**Multicellular organism reproduction**				**18**	**1,83E-02**	**RAD23B, DNMT3A, STAT5A, ABHD2, FOXO3, SIRT2, NR2C2, CITED2, INHBA, APP, FANCD2, KDM3A, EIF2B2, PABPC1L, INPP5B, JAM3, LHB, TBC1D20**
*GO:0048609	Multicellular organismal reproductive process				18	1,57E-02	RAD23B, DNMT3A, STAT5A, ABHD2, FOXO3, SIRT2, NR2C2, CITED2, INHBA, APP, FANCD2, KDM3A, EIF2B2, PABPC1L, INPP5B, JAM3, LHB, TBC1D20
	*GO:0019953	Sexual reproduction			17	5,73E-02	RAD23B, DNMT3A, STX2, ABHD2, FOXO3, SIRT2, NR2C2, INHBA, APP, FANCD2, KDM3A, PABPC1L, INPP5B, JAM3, KDM5B, LHB, TBC1D20
		*GO:0007276	Gamete generation		14	6,32E-02	RAD23B, DNMT3A, ABHD2, FOXO3, SIRT2, NR2C2, INHBA, FANCD2, KDM3A, PABPC1L, INPP5B, JAM3, LHB, TBC1D20
			*GO:0030728	Ovulation	3	2,50E-02	INHBA, FOXO3, LHB
		*GO:0022602	Ovulation cycle process		4	9,66E-02	INHBA, STAT5A, FOXO3, EIF2B2
**GO:0003006**	**Developmental process involved in reproduction**				**17**	**9,05E-02**	**CEBPA, CGA, ARID5B, STAT5A, ABHD2, FOXO3, SIRT2, CITED2, ZFP36L1, INHBA, PRLR, KDM3A, EIF2B2, PABPC1L, JAM3, KDM5B, TBC1D20**
*GO:0045137	Development of primary sexual characteristics				8	2,51E-02	CGA, INHBA, STAT5A, ARID5B, FOXO3, EIF2B2, TBC1D20, CITED2
	*GO:0046545	Development of primary female sexual characteristics			8	2,28E-02	CGA, INHBA, STAT5A, ARID5B, FOXO3, EIF2B2, TBC1D20, CITED2
*GO:0046660	Female sex differentiation				5	8,05E-02	INHBA, STAT5A, ARID5B, FOXO3, EIF2B2
**GO:0001816**	**Cytokine production**				**16**	**4,42E-02**	**AMCF-II, CEBPE, LY96, STAT5A, ELANE, SOCS1, IL9, CHI3L1, FOXP1, APOA1, IL4R, IRF1, NOS2, CASP1, TNFAIP3, TRAF3**
**GO:0042107	Cytokine metabolic process				5	6,54E-02	CEBPE, STAT5A, ELANE, IL9, IRF1
	*GO:0042089	Cytokine biosynthetic process			5	6,19E-02	CEBPE, STAT5A, ELANE, IL9, IRF1
**GO:0030155**	**Regulation of cell adhesion**				**16**	**5,26E-02**	**SIT1, ASS1, SMAD7, LDB1, STAT5A, CCL28, CITED2, APOA1, PRLR, TNFSF13B, ETS1, FANCD2, IL4R, IRF1, JAK3, MYOC**
**GO:0045785	Positive regulation of cell adhesion				11	5,56E-02	APOA1, TNFSF13B, ETS1, SMAD7, IL4R, LDB1, STAT5A, JAK3, CCL28, MYOC, CITED2
*GO:0022407	Regulation of cell-cell adhesion				13	1,25E-02	SIT1, ASS1, SMAD7, STAT5A, CCL28, CITED2, APOA1, TNFSF13B, ETS1, FANCD2, IL4R, IRF1, JAK3
	*GO:1903037	Regulation of leukocyte cell-cell adhesion			10	4,00E-02	SIT1, TNFSF13B, ASS1, ETS1, FANCD2, IL4R, STAT5A, IRF1, JAK3, CCL28
**GO:0061458**	**Reproductive system development**				**12**	**8,88E-02**	**ZFP36L1, CEBPA, CGA, INHBA, PRLR, STAT5A, ARID5B, FOXO3, EIF2B2, KDM5B, TBC1D20, CITED2**
*GO:0048608	Reproductive structure development				12	8,48E-02	ZFP36L1, CEBPA, CGA, INHBA, PRLR, STAT5A, ARID5B, FOXO3, EIF2B2, KDM5B, TBC1D20, CITED2
	*GO:0008406	Gonad development			8	2,28E-02	CGA, INHBA, STAT5A, ARID5B, FOXO3, EIF2B2, TBC1D20, CITED2
		*GO:0008585	Female gonad development		5	5,19E-02	INHBA, STAT5A, ARID5B, FOXO3, EIF2B2
**GO:0045786**	**Negative regulation of cell cycle**				**12**	**6,45E-02**	**CEBPA, TMEM67, INHBA, HHEX, CCNK, CDKN1A, KAT2B, IRF1, TNFAIP3, CDKN3, P14ARF, UHMK1**
**GO:1900046**	**Regulation of hemostasis**				**5**	**4,88E-02**	**SELP, STX2, KLKB1, ENPP4, PROC**
**GO:0042108**	**Positive regulation of cytokine biosynthetic process**				**4**	**7,00E-02**	**STAT5A, ELANE, IL9, IRF1**

*Subset **Part of
