# Supplementary material for: Transcriptomic Analysis of Porcine Endometrium during Implantation after In Vitro Stimulation by Adiponectin
Source: Int J Mol Sci. 2019 Mar 16;20(6):1335. doi: 10.3390/ijms20061335 (PMC6470965; doi:10.3390/ijms20061335)
Supplement: Supplementary file 1 [file ijms-20-01335-s001.zip › SF.5..pdf]

Supplementary Table 5

Complete list of gene interactions

Gene 1	Gene 2	Weight	Network type	Networks
PRLR	LHB	0,0203	Co-expression	Mallon-McKay-2013
HSD3B1	LHB	0,0119	Co-expression	Mallon-McKay-2013
HSD3B1	PRLR	0,0148	Co-expression	Mallon-McKay-2013
CGA	LHB	0,0189	Co-expression	Mallon-McKay-2013
CGA	PRLR	0,0205	Co-expression	Mallon-McKay-2013
CGA	HSD3B1	0,0131	Co-expression	Mallon-McKay-2013
CGB3	LHB	0,0273	Co-expression	Mallon-McKay-2013
CGB3	CGA	0,0216	Co-expression	Mallon-McKay-2013
FSHB	LHCGR	0,0139	Co-expression	Mallon-McKay-2013
HSD3B2	LHB	0,0165	Co-expression	Mallon-McKay-2013
HSD3B2	HSD3B1	0,0113	Co-expression	Mallon-McKay-2013
ICAM5	ESM1	0,0131	Co-expression	Mallon-McKay-2013
TGFA	MUC4	0,0063	Co-expression	Wang-Maris-2006
PRLR	TGFA	0,0072	Co-expression	Wang-Maris-2006
IL1B	CXCL8	0,0205	Co-expression	Wang-Maris-2006
ICAM5	TGFA	0,0047	Co-expression	Wang-Maris-2006
ICAM5	PTGS1	0,0103	Co-expression	Wang-Maris-2006
PRL	PRLR	0,0115	Co-expression	Wang-Maris-2006
LHB	MUC4	0,0045	Co-expression	Innocenti-Brown-2011
IL1B	FGF7	0,0133	Co-expression	Innocenti-Brown-2011
IL1B	CXCL8	0,0094	Co-expression	Innocenti-Brown-2011
TSHB	LHB	0,0038	Co-expression	Innocenti-Brown-2011
IL1B	CXCL8	0,0269	Co-expression	den Boon-Ahlquist-2015
CSRP2	HSD17B8	0,0124	Co-expression	den Boon-Ahlquist-2015
ICAM5	CGA	0,0125	Co-expression	den Boon-Ahlquist-2015
TSHR	LHCGR	0,0142	Co-expression	den Boon-Ahlquist-2015
IL1B	PTGS1	0,0200	Co-expression	Alizadeh-Staudt-2000
ENPP1	INSR	0,0225	Co-expression	Alizadeh-Staudt-2000
CXCL8	TGFA	0,0212	Co-expression	Wang-Cheung-2015
IL1B	TGFA	0,0116	Co-expression	Dobbin-Giordano-2005
IL1B	CXCL8	0,0111	Co-expression	Dobbin-Giordano-2005
ICAM5	HSD3B1	0,0129	Co-expression	Dobbin-Giordano-2005
ICAM5	FSHB	0,0184	Co-expression	Dobbin-Giordano-2005
PRL	PRLR	0,0166	Co-expression	Dobbin-Giordano-2005
INHA	HSD3B2	0,0035	Co-expression	Dobbin-Giordano-2005
IL1B	CXCL8	0,0224	Co-expression	Bild-Nevins-2006 B
LHCGR	FGF7	0,0272	Co-expression	Bild-Nevins-2006 B
FSHB	MUC4	0,0072	Co-expression	Bild-Nevins-2006 B
HSD3B2	HSD3B1	0,0129	Co-expression	Bild-Nevins-2006 B
ICAM5	TGFA	0,0111	Co-expression	Bild-Nevins-2006 B
PRL	ICAM5	0,0057	Co-expression	Bild-Nevins-2006 B
INHA	MUC4	0,0219	Co-expression	Bild-Nevins-2006 B
PRLR	TGFA	0,0105	Co-expression	Ramaswamy-Golub-2001
IL1B	CXCL8	0,0096	Co-expression	Ramaswamy-Golub-2001
FSHB	TGFA	0,0076	Co-expression	Ramaswamy-Golub-2001
CSRP2	INSR	0,0210	Co-expression	Ramaswamy-Golub-2001
TSHR	FSHB	0,0085	Co-expression	Ramaswamy-Golub-2001
IL1B	CXCL8	0,0175	Co-expression	Bonome-Birrer-2008
MAP3K3	INSR	0,0206	Co-expression	Bonome-Birrer-2008
TSHB	TGFA	0,0067	Co-expression	Bonome-Birrer-2008
HSD3B2	TSHB	0,0051	Co-expression	Bonome-Birrer-2008
ENPP1	LHCGR	0,0148	Co-expression	Bonome-Birrer-2008
PRL	CGA	0,0020	Co-expression	Bonome-Birrer-2008
IL1B	CXCL8	0,0114	Co-expression	Boldrick-Relman-2002
IL1B	CXCL8	0,0092	Co-expression	Noble-Diehl-2008
HSD3B2	HSD3B1	0,0335	Co-expression	Noble-Diehl-2008
INHA	CGA	0,0172	Co-expression	Noble-Diehl-2008
PRLR	LHB	0,0177	Co-localization	Schadt-Shoemaker-2004
HSD3B1	LHB	0,0219	Co-localization	Schadt-Shoemaker-2004
HSD3B1	PRLR	0,0208	Co-localization	Schadt-Shoemaker-2004
INSR	LHB	0,0158	Co-localization	Schadt-Shoemaker-2004

INSR	PRLR	0,0243	Co-localization	Schadt-Shoemaker-2004
INSR	HSD3B1	0,0149	Co-localization	Schadt-Shoemaker-2004
CGA	LHB	0,0220	Co-localization	Schadt-Shoemaker-2004
IL1B	CXCL8	0,0187	Co-localization	Schadt-Shoemaker-2004
PTGIS	PTGS1	0,0119	Co-localization	Schadt-Shoemaker-2004
CSRP2	LHB	0,0344	Co-localization	Schadt-Shoemaker-2004
CSRP2	PRLR	0,0216	Co-localization	Schadt-Shoemaker-2004
CSRP2	HSD3B1	0,0231	Co-localization	Schadt-Shoemaker-2004
CSRP2	INSR	0,0215	Co-localization	Schadt-Shoemaker-2004
HSD3B2	LHB	0,0143	Co-localization	Schadt-Shoemaker-2004
HSD3B2	PRLR	0,0183	Co-localization	Schadt-Shoemaker-2004
HSD3B2	HSD3B1	0,0337	Co-localization	Schadt-Shoemaker-2004
HSD3B2	LHCGR	0,0206	Co-localization	Schadt-Shoemaker-2004
HSD3B2	CSRP2	0,0157	Co-localization	Schadt-Shoemaker-2004
CDKN1C	LHB	0,0200	Co-localization	Schadt-Shoemaker-2004
CDKN1C	PRLR	0,0220	Co-localization	Schadt-Shoemaker-2004
CDKN1C	HSD3B1	0,0234	Co-localization	Schadt-Shoemaker-2004
CDKN1C	INSR	0,0205	Co-localization	Schadt-Shoemaker-2004
CDKN1C	CSRP2	0,0246	Co-localization	Schadt-Shoemaker-2004
CDKN1C	HSD3B2	0,0184	Co-localization	Schadt-Shoemaker-2004
PRL	LHB	0,0248	Co-localization	Schadt-Shoemaker-2004
PRLR	LHB	0,0095	Co-localization	Johnson-Shoemaker-2003
CGA	LHB	0,0046	Co-localization	Johnson-Shoemaker-2003
CGA	PRLR	0,0096	Co-localization	Johnson-Shoemaker-2003
IL1B	CXCL8	0,0141	Co-localization	Johnson-Shoemaker-2003
PTGIS	FGF7	0,0108	Co-localization	Johnson-Shoemaker-2003
PTGIS	PTGS1	0,0201	Co-localization	Johnson-Shoemaker-2003
HSD3B2	HSD3B1	0,01	Co-localization	Johnson-Shoemaker-2003
HSD3B2	LHCGR	0,01	Co-localization	Johnson-Shoemaker-2003
ENPP1	INSR	0,02	Co-localization	Johnson-Shoemaker-2003
CDKN1C	LHB	0,00	Co-localization	Johnson-Shoemaker-2003
CDKN1C	PRLR	0,02	Co-localization	Johnson-Shoemaker-2003
CDKN1C	HSD3B1	0,01	Co-localization	Johnson-Shoemaker-2003
CDKN1C	CGA	0,00	Co-localization	Johnson-Shoemaker-2003
INHA	LHCGR	0,02	Co-localization	Johnson-Shoemaker-2003
INHA	HSD3B2	0,01	Co-localization	Johnson-Shoemaker-2003
INHA	ICAM5	0,02	Co-localization	Johnson-Shoemaker-2003
CGA	LHB	0,20	Pathway	REACTOME
LHCGR	LHB	0,67	Pathway	REACTOME
LHCGR	CGA	0,20	Pathway	REACTOME
CGB3	CGA	0,41	Pathway	REACTOME
MAP3K3	IL1B	0,61	Pathway	REACTOME
TSHB	CGA	0,20	Pathway	REACTOME
FSHB	CGA	0,14	Pathway	REACTOME
SQSTM1	IL1B	0,32	Pathway	REACTOME
SQSTM1	MAP3K3	0,3192	Pathway	REACTOME
INHA	CGA	0,2148	Pathway	REACTOME
INHA	FSHB	0,4981	Pathway	REACTOME
TSHR	CGA	0,2012	Pathway	REACTOME
TSHR	TSHB	0,6667	Pathway	REACTOME
CGA	LHB	0,0670	Pathway	Wu-Stein-2010
LHCGR	LHB	0,0986	Pathway	Wu-Stein-2010
LHCGR	CGA	0,0562	Pathway	Wu-Stein-2010
TSHB	CGA	0,0677	Pathway	Wu-Stein-2010
FSHB	CGA	0,0699	Pathway	Wu-Stein-2010
PRL	PRLR	0,2205	Pathway	Wu-Stein-2010
TSHR	CGA	0,0691	Pathway	Wu-Stein-2010
TSHR	TSHB	0,1225	Pathway	Wu-Stein-2010
CGA	LHB	0,2685	Physical Interactions	IREF-SMALL-SCALE-STUDIES
GALNT11	MUC4	0,9501	Physical Interactions	IREF-SMALL-SCALE-STUDIES
LHCGR	LHB	0,1999	Physical Interactions	IREF-SMALL-SCALE-STUDIES
LHCGR	CGA	0,1059	Physical Interactions	IREF-SMALL-SCALE-STUDIES
CGB3	CGA	0,2539	Physical Interactions	IREF-SMALL-SCALE-STUDIES
CGB3	LHCGR	0,1890	Physical Interactions	IREF-SMALL-SCALE-STUDIES
TSHB	CGA	0,4054	Physical Interactions	IREF-SMALL-SCALE-STUDIES

RHBDF1	TGFA	0,3434	Physical Interactions	IREF-SMALL-SCALE-STUDIES
FSHB	CGA	0,2613	Physical Interactions	IREF-SMALL-SCALE-STUDIES
FSHB	CGB3	0,4662	Physical Interactions	IREF-SMALL-SCALE-STUDIES
PTGIS	PTGS1	0,5032	Physical Interactions	IREF-SMALL-SCALE-STUDIES
SQSTM1	MAP3K3	0,0103	Physical Interactions	IREF-SMALL-SCALE-STUDIES
NUCB1	PTGS1	0,2234	Physical Interactions	IREF-SMALL-SCALE-STUDIES
NKD2	TGFA	0,1518	Physical Interactions	IREF-SMALL-SCALE-STUDIES
ESM1	ITGAL	0,4143	Physical Interactions	IREF-SMALL-SCALE-STUDIES
ENPP1	INSR	0,2397	Physical Interactions	IREF-SMALL-SCALE-STUDIES
ICAM5	ITGAL	0,2410	Physical Interactions	IREF-SMALL-SCALE-STUDIES
PRL	PRLR	0,1281	Physical Interactions	IREF-SMALL-SCALE-STUDIES
TSHR	LHCGR	0,0623	Physical Interactions	IREF-SMALL-SCALE-STUDIES
TSHR	TSHB	0,2385	Physical Interactions	IREF-SMALL-SCALE-STUDIES
CGA	LHB	0,2457	Physical Interactions	IREF-HPRD
GALNT11	MUC4	0,9335	Physical Interactions	IREF-HPRD
LHCGR	LHB	0,2058	Physical Interactions	IREF-HPRD
LHCGR	CGA	0,1065	Physical Interactions	IREF-HPRD
CGB3	CGA	0,2403	Physical Interactions	IREF-HPRD
CGB3	LHCGR	0,2013	Physical Interactions	IREF-HPRD
TSHB	CGA	0,3867	Physical Interactions	IREF-HPRD
RHBDF1	TGFA	0,4550	Physical Interactions	IREF-HPRD
FSHB	CGA	0,2468	Physical Interactions	IREF-HPRD
FSHB	CGB3	0,4664	Physical Interactions	IREF-HPRD
NUCB1	PTGS1	0,5983	Physical Interactions	IREF-HPRD
NKD2	TGFA	0,4550	Physical Interactions	IREF-HPRD
ENPP1	INSR	0,2547	Physical Interactions	IREF-HPRD
ICAM5	ITGAL	0,4619	Physical Interactions	IREF-HPRD
PRL	PRLR	0,2462	Physical Interactions	IREF-HPRD
TSHR	LHCGR	0,0694	Physical Interactions	IREF-HPRD
TSHR	TSHB	0,2519	Physical Interactions	IREF-HPRD
TGFA	MUC4	0,0116	Shared protein domains	INTERPRO
PTGS1	MUC4	0,0133	Shared protein domains	INTERPRO
PTGS1	TGFA	0,0113	Shared protein domains	INTERPRO
CGA	LHB	0,0133	Shared protein domains	INTERPRO
IL1B	FGF7	0,0231	Shared protein domains	INTERPRO
CGB3	LHB	0,0587	Shared protein domains	INTERPRO
CGB3	CGA	0,0131	Shared protein domains	INTERPRO
TSHB	LHB	0,0596	Shared protein domains	INTERPRO
TSHB	CGA	0,0133	Shared protein domains	INTERPRO
TSHB	CGB3	0,0585	Shared protein domains	INTERPRO
FSHB	LHB	0,0583	Shared protein domains	INTERPRO
FSHB	CGA	0,0130	Shared protein domains	INTERPRO
FSHB	CGB3	0,0572	Shared protein domains	INTERPRO
FSHB	TSHB	0,0580	Shared protein domains	INTERPRO
SQSTM1	MAP3K3	0,0152	Shared protein domains	INTERPRO
HSD3B2	HSD3B1	0,0349	Shared protein domains	INTERPRO
NKD2	NUCB1	0,0050	Shared protein domains	INTERPRO
INHA	CGA	0,0105	Shared protein domains	INTERPRO
TSHR	LHCGR	0,0627	Shared protein domains	INTERPRO
