# Supplementary figures and images for: Transcriptomic Analysis of Porcine Endometrium during Implantation after In Vitro Stimulation by Adiponectin
Source: Int J Mol Sci. 2019 Mar 16;20(6):1335. doi: 10.3390/ijms20061335 (PMC6470965; doi:10.3390/ijms20061335)

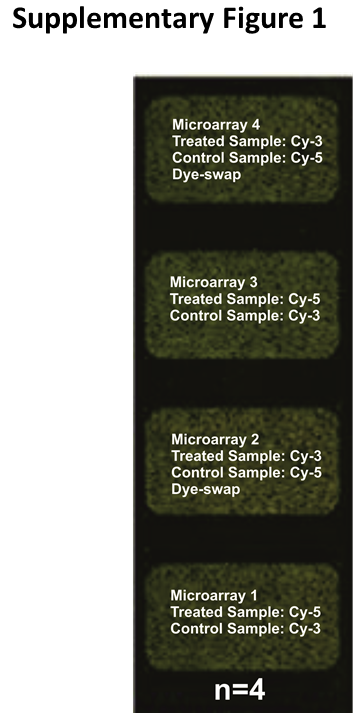

Supplement: Supplementary file 1 [file ijms-20-01335-s001.zip › SF.6..tif]
